# Engineered *Bacillus subtilis* Systems for the Characterization and Detection of Peptide-Based Quorum Sensing in Gram-Positive Bacteria

**DOI:** 10.1021/acs.biochem.6c00065

**Published:** 2026-04-15

**Authors:** Emma L. Eisenbraun, Natalie L. Beebe, Alexandra E. Nelson, Brendan N. Prosser, Troy D. Vulpis, Amanda E. Appel, Helen E. Blackwell

**Affiliations:** † Department of Chemistry, University of Wisconsin−Madison, 1101 University Ave., Madison, Wisconsin 53706, United States; ‡ Microbiology Doctoral Training Program, University of Wisconsin−Madison, 1550 Linden Dr., Madison, Wisconsin 53706, United States

## Abstract

Many questions remain about the biochemical mechanisms of quorum sensing (QS), particularly in Gram-positive bacterial pathogens that pose serious threats to human health such as *Staphylococcus aureus*. Methods to isolate and study the molecular components of these signaling systems *in vitro* are not straightforward. To date, most mechanistic investigations into QS systems have been accomplished in cells and, to a lesser extent, using chemical inhibitors. Herein, we report the development and characterization of a set of engineered *Bacillus subtilis* strains that can be used to study the accessory gene regulator (*agr*) QS circuits of a variety of Gram-positive bacteria. We cloned the *S. aureus* *agr* QS machinery into *B. subtilis* as proof-of-concept to generate “sender” cells capable of autoinducing peptide (AIP) signal biosynthesis, “receiver” cells capable of signal transduction, and “full” cells containing the full *agr* QS system. We verified that the *B. subtilis* sender cells could produce the native *S. aureus* AIP and that the receiver and full cells responded to both the AIP signal and a panel of known chemical *agr* modulators. The approach was readily transferable to study the *agr* QS systems of *Staphylococcus epidermidis* and *Listeria monocytogenes*, revealing interesting differences that could indicate underlying divergences in native QS mechanisms. We also demonstrate the ability of these *B. subtilis* strains to function as biosensors to detect the native AIPs of bacteria and produce a targeted antibiotic in response. These engineered systems should find utility for the study of QS in a range of fundamental and applied contexts.

## Graphical abstract

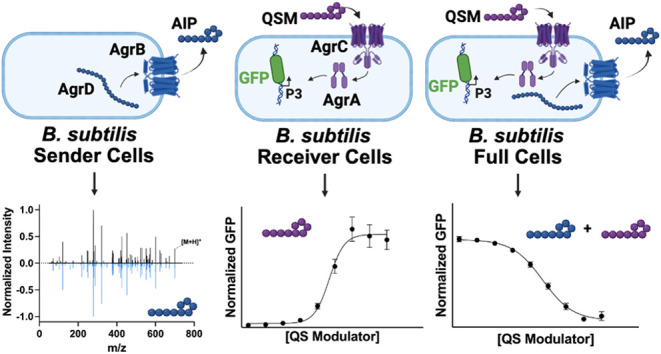

## Introduction

Quorum sensing (QS) is a cell-to-cell communication pathway that allows bacteria to regulate gene expression according to cell density. These genes primarily control group behaviors, such as virulence factor production, biofilm formation, motility, and sporulation.^1,2^ Many of these behaviors play prominent roles in both acute and chronic bacterial infections. Because of this regulatory connection, targeting QS pathways has attracted considerable attention as a means of controlling and reducing bacterial virulence without affecting growth, offering a potential antivirulence strategy that contrasts with traditional antibiotics.^3^

Many common Gram-positive bacterial pathogens that pose threats to human and animal health regulate virulence at least in part using QS. One such bacterium is *Staphylococcus aureus*, the leading cause of endocarditis, bacteremia, osteomyelitis, and skin and soft tissue infections in humans.^4^ The prevalence of methicillin-resistant *S. aureus* (MRSA) in these infections poses an increasingly serious threat and medical burden.^5^ Another prominent Gram-positive bacterium that uses QS is *Staphylococcus epidermidis*, an opportunistic pathogen with a propensity to form biofilm infections on abiotic surfaces such as indwelling medical devices.^6^ These biofilms promote antibiotic resistance and elicit host immune responses, resulting in *S. epidermidis* emerging as a common cause of hospital-acquired infections.^7^ *Listeria monocytogenes* represents a third well-known and QS-active Gram-positive pathogen that is responsible for the foodborne illness listeriosis and accounts for 19% of all foodborne illness-related deaths in the United States.^8^ Further characterization of the QS systems in these three species could provide new pathways toward strategies to mitigate human infection and associated suffering.

One of the best-characterized QS systems in Gram-positive bacteria is the accessory gene regulator (*agr*) system, which is utilized by the three pathogens introduced above to initiate a broad range of virulent behaviors. The *agr* QS system is best understood in *S. aureus* (Figure 1A) and is comprised primarily of four proteinaceous components: two proteins involved in QS signal biosynthesis (AgrB and AgrD) and two proteins involved in signal transduction (AgrC and AgrA).^9^ Briefly, the propeptide AgrD is processed by the transmembrane protease AgrB, which cleaves the AgrD C-terminal domain and facilitates macrocycle formation, creating the pro-autoinducing peptide (AIP).^10^ The N-terminal amphipathic leader sequence of the pro-AIP is then cleaved by a membrane protease (identified as MroQ in some subspecies [i.e., specificity groups] of *S. aureus*),^11^ to yield the resulting AIP signal. The AIP, which consists of a five-membered thiolactone macrocycle and a short N-terminal tail,^12^ is exported out of the cell by an unknown mechanism. Once cellular density has sufficiently increased, a threshold concentration of AIP is achieved in the local environment allowing for productive AIP binding to the extracellular sensor domain of its target receptor, the transmembrane histidine kinase AgrC. AIP:AgrC binding causes autophosphorylation of AgrC, which subsequently phosphorylates its corresponding cytoplasmic response regulator, AgrA.^13,14^ Upon phosphorylation, AgrA dimerizes and binds at the divergent P2 and P3 promoters.^15,16^ Binding at the P2 promoter upregulates the production of the *agr* machinery, resulting in a positive feedback cycle (or “autoinduction”) that is a hallmark of QS systems. In turn, AgrA binding at the P3 promoter activates the production of the effector molecule RNAIII, which is responsible for the direct and indirect regulation of an arsenal of virulence factors.^17^ For *S. aureus* and other closely related species, several divergent *agr* specificity groups exist (in *S. aureus*, groups I–IV), each with unique AgrB, AgrD, and AgrC components and distinct AIP signals, but with highly similar AgrA response regulators. These specificity groups exhibit varying degrees of *agr* interference (i.e., inhibition of nonself groups) via their AIP signals.^18,19^

**Figure 1:**
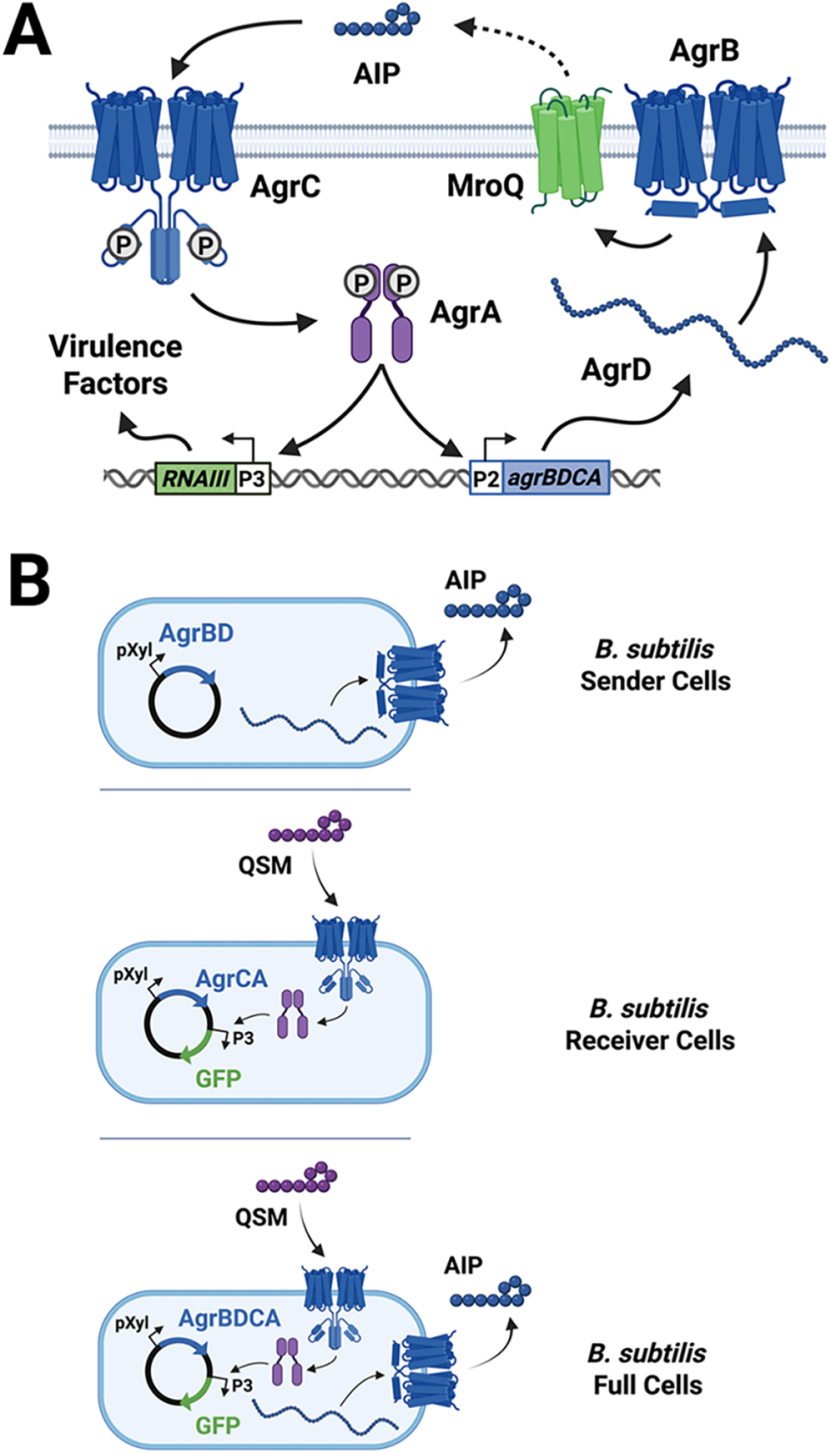
Schematic of the *agr* QS system and three types of *Bacillus subtilis* systems developed in this study. (A) The *agr* system in *S. aureus*. AIP is biosynthesized through the processing of the propeptide AgrD via the membrane protease AgrB and (in certain groups) MroQ. AIPs bind to the AgrC receptor, which activates the response regulator AgrA, upregulating the expression of the *agr* operon and the RNAIII effector molecule, responsible for regulating a variety of virulence factors. (B) *Top:* The *B. subtilis* sender cells contain xylose-inducible AgrBD machinery, capable of AIP biosynthesis. *Middle;* The *B. subtilis* receiver cells contain xylose-inducible AgrCA machinery, capable of receiving QS signals and reading out activity using GFP. *Bottom:* The *B. subtilis* full cells contain both biosynthesis and signal transduction machinery, capable of AIP generation and QS signal detection with fluorescence readout upon induction with xylose.

Since the discovery of the native AIP signals and their interference, there has been considerable interest in the development of chemical ligands capable of inhibiting *agr* QS systems and thereby associated bacterial virulence.^20–22^ A significant number of peptides, synthetic small molecules, and natural products have been discovered that are reported to inhibit various components of the *S. aureus* *agr* QS system.^23^ In *S. epidermidis*, a small number of non-native peptide *agr* activators and inhibitors have been developed,^24–26^ and in *L. monocytogenes*, an even more limited set of peptide and small molecule *agr* inhibitors have been explored.^27–29^ Representative structures are shown in Figure 2. While progress continues in the design of potent and efficacious QS modulators (QSMs), the mechanisms of the majority of these compounds are underexplored, including identifying their specific *agr* targets. Nevertheless, QSMs, of both natural and synthetic origins, represent valuable tools to investigate *agr* as a target for infection control in a range of cell-based and *in vivo* infection models^20,21,23,30,31^ and can complement genetic approaches in such investigations.

**Figure 2:**
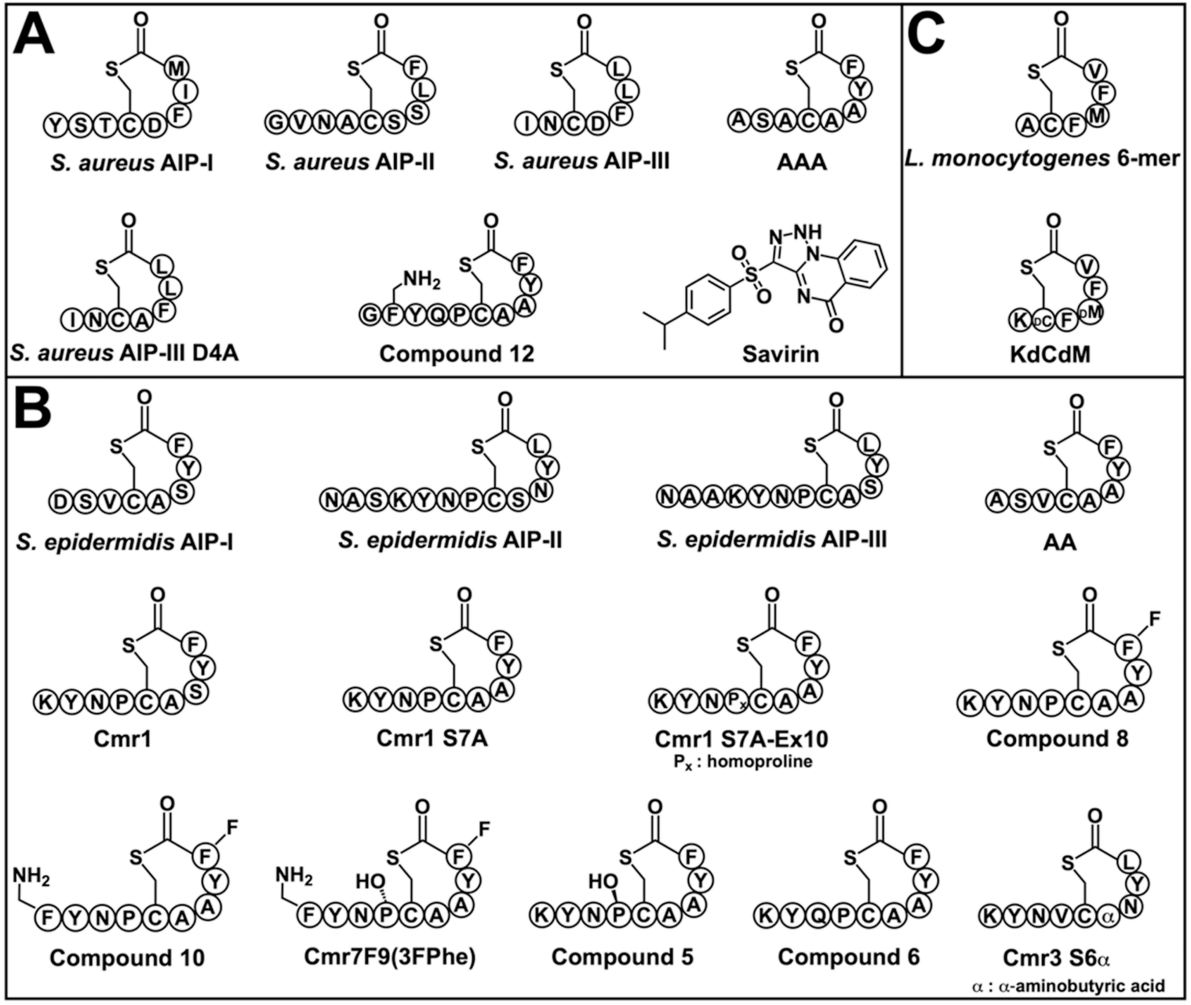
Panel of native and non-native compounds used in this study to characterize the *B. subtilis* constructs. Compounds were previously identified to modulate *agr* in (A) *S. aureus*, (B) *S. epidermidis*, and/or (C) *L. monocytogenes*. Letters in circles indicate amino acids with standard abbreviations. See text for citations to each compound.

Heterologous or engineered QS systems, in which the signaling machinery can be studied outside of the native host cell environment, can provide multiple advantages for mechanistic studies of QSMs, including the separation of the QS system from pathways that intersect with it in the host background, the enhanced genetic tractability of many heterologous organisms, and the manipulation of (typically) a BSL1 organism. Much of the past work on heterologous QS systems has focused extensively on LuxI/LuxR-type QS pathways in Gram-negative bacteria.^32^ Far less has been reported on the development of heterologous Gram-positive QS systems, due in part to the lack of mechanistic information about many of these QS pathways and challenges associated with the genetic manipulation of Gram-positive bacteria. In 2013, the Collins laboratory reported one of the first synthetic QS systems based on that of a Gram-positive bacterium. In a series of studies, the authors were able to engineer *Escherichia coli* and *Priestia megaterium* that expressed components of the *S. aureus* *agr* system.^33,34^ The Collins team created “receiver cells” that expressed AgrC and AgrA, along with “sender cells” that expressed AgrB and AgrD from group-I *S. aureus*. They also developed a “full cell” system where the full *agr* operon was placed in *P. megaterium* and showed that monocultures were capable of significant activation.^33,34^ The goal of the Collins lab was not to explore the mechanisms of *agr* QS with these engineered strains, but rather to demonstrate proof-of-concept that the engineered strains could be used as tools for synthetic biology. Because of this focus, the authors only tested the ability of the sender or full cell supernatants to activate the corresponding receiver cells and did not characterize or quantitate the amount of AIP product generated.

Since these initial reports, several other groups have introduced the *S. aureus* *agr* QS machinery into heterologous strains, including *B. subtilis*,^35,36^ *S. epidermidis*,^37^ *Lactobacillus reuteri*,^38^ *Lactobacillus plantarum*,^39^ *Clostridium sporogenes*,^40^ and *E. coli*,^41^ for the purposes of creating biosensors, exploring delivery mechanisms of select agents, or modulating the induction of protein expression. These past reports demonstrate important advancements, including the fine-tuning of autoinduction, promoter strength, fluorescent readout, and temporal dynamics of the systems.^36,40^ One notable advance involved expressing *S. aureus* *agr* components (in *L. plantarum* and *S. epidermidis*) to drive production of the antibacterial enzyme lysostaphin in response to AIP-I detection, demonstrating targeted killing of *S. aureus*.^37,39^ Overall, these past studies were largely similar to those of the Collins group: most focused solely on *S. aureus* *agr* group-I and not on mechanistic questions about *agrBDCA* function. None of these studies examined QSMs outside of native signaling molecules, and apart from the work in *L. reuteri*, *L. plantarum*, and *C. sporogenes*, these studies neither confirmed the structure nor quantified the amount of AIP product generated in the heterologous systems.^35,37,39,40^

Herein, we report the development and characterization of a new suite of synthetic *agr* QS systems in *B. subtilis*. We first introduced the *S. aureus* *agr* machinery to this platform to establish our methods and generate sender, receiver, and full cells (Figure 1B). AIP production by the sender cells was verified and quantified using supernatant assays and mass spectrometry (MS) analysis. The receiver and full cells were used to evaluate a library of known synthetic *S. aureus* QSMs, which demonstrated activity profiles in these heterologous systems that helped to illuminate their mechanisms of *agr* modulation in wild-type *S. aureus*. The engineered platform was readily adapted to produce *agr* components from both *S. epidermidis* and *L. monocytogenes*, and similar characterizations of AIP production and response to QSMs were conducted. To our knowledge, this work represents the first report of the study of *S. epidermidis* and *L. monocytogenes* *agr* QS components outside of their native host. Lastly, we developed a biosensor system in *B. subtilis* using the *S. aureus* *agr* machinery that can detect three of the four native *S. aureus* AIPs and trigger the production of the antibacterial enzyme lysostaphin in response. This *B. subtilis* platform system offers a straightforward approach to explore both *agr* QS mechanisms and the activities of QSMs in Gram-positive species, and to detect and respond to a broad set of AIP signals, and thereby, pathogenic bacteria.

## Experimental Section

### Materials

All standard chemical and biological reagents and solvents were purchased from commercial suppliers and used according to the instructions. Water (18 MΩ) was purified using an Arium Pro ultrapure water system (Sartorius). All solid-phase extraction solvents (water and acetonitrile (CH_3_CN)) were HPLC grade (Sigma-Aldrich). All native AIPs, analogues, and small molecules previously reported by our laboratory were acquired from in-house stocks and synthesized according toreported methods.^22,24,25,27^ *S. aureus* AIP-III + Ala (used as an internal standard in MS studies) was synthesized according to our previous methods;^22^ HPLC and MS characterization data are in the SI. Savirin was purchased from AK Scientific.

### Bacterial Strains and Growth Methods

All bacterial strains used in this study are listed in Table 1. *B. subtilis* containing either *S. aureus* or *L. monocytogenes* *agr* circuitry and *L. monocytogenes* were grown in Luria–Bertani medium (LB, Research Products International). *B. subtilis* containing *S. epidermidis* *agr* circuitry and *S. epidermidis* were grown in Tryptic Soy Broth (TSB NutriSelect Basic, Sigma-Aldrich). *S. aureus* was grown in Brain Heart Infusion (BHI, Teknova). *B. subtilis* strains carrying a resistance marker were selected using erythromycin (1 μg/mL) combined with lincomycin (25 μg/mL), and *S. aureus* reporter strains (AH1677 and AH430) were selected using chloramphenicol (10 μg/mL). All bacterial cultures were grown at 37 °C with shaking at 200 rpm unless otherwise noted. A 10% w/v xylose (Sigma-Aldrich) stock was made in either LB or TSB medium and diluted to induce gene expression.

**Table 1:** Bacterial Strains and Plasmids Used in This Study

name	description	ref or source
Strains		
*S. aureus* USA300 LAC	Clinical isolate, *agr* group-I	Otto^42^
*S. aureus* USA300 LAC Δ*agr*	USA300 LAC, *agrBDCA* knockout	Lee et al.^43^
*S. aureus* RN6390B	Wild-type *S. aureus* lab strain, *agr* group-I	Ji et al.^18^
*S. aureus* RN6925	Wild-type *S. aureus* clinical isolate, *agr* group-II	Ji et al.^18^
*S. aureus* MW2	Wild-type *S. aureus* USA400, *agr* group-III	King et al.^44^
*S. aureus* RN4850	Wild-type *S. aureus* lab strain, *agr* group-IV	Lyon et al.^45^
*S. aureus* AH1677	USA300 LAC/pDB59 (Cam^r^)	Yarwood et al.^46^ and Kirchdoerfer et al.^47^
*S. aureus* AH430	SA502A/pDB59 (Cam^r^)	Yarwood et al.^46^, Malone et al.^48^ and Kirchdoerfer et al.^47^
*S. epidermidis* RP62A	Clinical isolate, *agr* group-I	Gill et al.^49^
*S. epidermidis* 1457	Wild-type *S. epidermidis*, *agr* group-II	Mack et al.^50^
*S. epidermidis* 8247	Wild-type *S. epidermidis*, *agr* group-III	Olson et al.^51^
*L. monocytogenes* EGD-e	Wild-type *L. monocytogenes* lab strain (serovar 1/2a)	Glaser et al.^52^
*E. coli* DH5α	Cloning strain	NEB^a^
*B. subtilis* 168	trpC2 lab strain	BGSC^b^
Plasmids		
pBS0EXylRP_xylA(V2)_	Empty vector, shuttle vector for cloning in *E. coli* (AmpR), xylose-inducible expression in *B. subtilis* (Mls^r^)	Popp et al.^53^ BGSC^b^
pXyl-Sa-agrBD	Derivative of pBS0EXylRPxylA(V2) with *S. aureus* AgrBD added for xylose-inducible expression (Mls^r^)	This report
pXyl-Sa-agrCA-GFP	Derivative of pBS0EXylRPxylA(V2) with *S. aureus* AgrCA added for xylose-inducible expression and GFP added under P3 expression (Mls^r^)	This report
pXyl-Sa-agrCA-LysT	Derivative of pBS0EXylRPxylA(V2) with *S. aureus* AgrCA added for xylose-inducible expression and lysostaphin added under P3 expression (Mls^r^)	This report
pXyl-Sa-agrBDCA-GFP	Derivative of pBS0EXylRPxylA(V2) with *S. aureus* AgrBDCA added for xylose-inducible expression and GFP added under P3 expression (Mls^r^)	This report
pXyl-Se-agrBD	Derivative of pBS0EXylRPxylA(V2) with *S. epidermidis* AgrBD added for xylose-inducible expression (Mls^r^)	This report
pXyl-Se-agrCA-GFP	Derivative of pBS0EXylRPxylA(V2) with *S. epidermidis* AgrCA added for xylose-inducible expression and GFP added under P3 expression (Mls^r^)	This report
pXyl-Se-agrBDCA-GFP	Derivative of pBS0EXylRPxylA(V2) with *S. epidermidis* AgrBDCA added for xylose-inducible expression and GFP added under P3 expression (Mls^r^)	This report
pSa-control-GFP	Derivative of pXyl-Sa-agrBDCA-GFP with the AgrBDCA gene removed (Mls^r^)	This report
pSe-control-GFP	Derivative of pXyl-Se-agrBDCA-GFP with the AgrBDCA gene removed (Mls^r^)	This report
pLm-control-GFP	Derivative of pXyl-Lm-agrBDCA-GFP with the AgrBDCA gene removed (Mls^r^)	This report
pXyl-Lm-agrBD	Derivative of pBS0EXylRPxylA(V2) with *L. monocytogenes* AgrBD added for xylose-inducible expression (Mls^r^)	This report
pXyl-Lm-agrCA-GFP	Derivative of pBS0EXylRPxylA(V2) with *L. monocytogenes* AgrCA added for xylose-inducible expression and GFP added under P3 expression (Mls^r^)	This report
pXyl-Lm-agrBDCA-GFP	Derivative of pBS0EXylRPxylA(V2) with *L. monocytogenes* AgrBDCA added for xylose-inducible expression and GFP added under P3 expression (Mls^r^)	This report

a New England Biolabs.

b Bacillus Genetic Stock Center.

### Plasmid Construction

All plasmids used and created in this report are listed in Table 1. Primers used in plasmid construction were ordered from Integrated DNA Technologies and are listed in Table S1. Vectors in this study were generated using standard cloning techniques, including a combination of Gibson Assemblies using Phusion polymerase chain reaction (PCR) and Q5 site-directed mutagenesis methods.^54^ Full details of plasmid construction, including primer and vector sequences, are included in the Supporting Information (Figure S1, Tables S1, and S2). Briefly, *agr* machinery was cloned from *S. aureus*, *S. epidermidis*, and *L. monocytogenes* genomic DNA (extracted from RN6390B, RP62A, and EGD-e, respectively). These genes were added under control of a xylose-inducible promoter to the vector pBS0EXylRP_xylA(V2)_, along with a *B. subtilis* ribosomal binding site (RBS).^53^ In the receiver cells and the full cells, a *B. subtilis*-optimized GFP gene was added under the control of the *S. aureus*, *S. epidermidis*, or *L. monocytogenes* P3 promoter. For the biosensor system, lysostaphin fused to an AmyE secretion tag replaced GFP downstream of the *S. aureus* P3 promoter. Constructs that isolate components of the *agr* operon or remove the *agr* operon were created for all three *agr* systems to serve as negative controls. All vectors were characterized using Plasmidsaurus sequencing (see SI for full vector sequences). Plasmids were transformed into *B. subtilis* 168 cells using methods published by Bennallack et al.,^55^ generating the final sender cells (containing pXyl-agrBD), receiver cells (containing pXyl-agrCA-GFP), full cells (containing pXyl-agrBDCA-GFP), and biosensor cells (containing pXyl-Sa-agrCA-LysT), along with the controls (containing pXyl-control-GFP, pXyl-agrA-GFP, and pXyl-agrC-GFP). Growth curves for all engineered strains can be found in the SI (Figure S2).

### *B. subtilis* Supernatant Assays

To isolate supernatants, overnight cultures of *B. subtilis* sender cells or wild-type (WT) *B. subtilis* 168 were diluted 1:100 in LB or TSB medium supplemented with 1% xylose and grown at 37 °C for 8 h at 200 rpm. Cells were then pelleted by centrifugation at 4 °C for 15 min at 4000 rpm, and supernatant was filtered using Steriflip filter units (Millipore, 0.22 μm). Supernatant was frozen with liquid nitrogen and lyophilized for 36 h. Lyophilized supernatants were resuspended in sterile water at either 1/10 or 1/25 of their original volume.

To test the 10-fold or 25-fold concentrated *B. subtilis* sender cell supernatants, they were serially diluted in WT *B. subtilis* 168 supernatant. Aliquots (20 μL) of the diluted supernatant were added to each of the wells of a black 96-well polystyrene microtiter plate (Costar). The diluted supernatant samples from *B. subtilis* sender cells based on *S. aureus* and *L. monocytogenes* *agr* were tested in technical and biological triplicate, and supernatant samples isolated from *B. subtilis* sender cells based on *S. epidermidis* *agr* were tested in technical duplicate and biological triplicate. A 20 μL-aliquot of WT *B. subtilis* supernatant was included as a vehicle control. Overnight cultures of *S. aureus* fluorescent reporter strains (AH1677 and AH430) were grown for 20 h and diluted 50-fold with fresh BHI medium. Aliquots (180 μL) of the diluted reporter cultures were added to each of the wells of the microtiter plate containing supernatant. Plates were then incubated at 37 °C for 24 h at 750 rpm on a Stuart SI505 microtiter plate shaker incubator, and the fluorescence (Excitation 500 nm/Emission 540 nm) and OD_600_ of each well were measured using a Biotek Synergy 2 plate reader running Gen5 3.12 software. Data were normalized to the vehicle (100%) and media (0%) controls and then analyzed using GraphPad Prism software (v. 10.3.1) to generate sigmoidal curve fits ([inhibitor] vs response, four-parameters).

### *B. subtilis* Fluorescent Reporter Assays

QSM agonism and antagonism assays in *B. subtilis* receiver and full cells were performed using previously established protocols with slight changes.^25^ Compound stock solutions in DMSO (1–10 mM) were serially diluted 3-fold with DMSO or left undiluted for point concentration experiments. Aliquots (2 μL) of the compound solutions were added to each of the wells of a black 96-well polystyrene microtiter plate (Costar). Each compound was tested in technical and biological triplicate unless otherwise noted, and 2 μL of DMSO was included as a vehicle control. For the *B. subtilis* receiver and full cells containing the *S. aureus* or *L. monocytogenes* *agr* systems, overnight cultures were grown for 20 h and diluted 50-fold with fresh LB medium, supplemented with 1% w/v xylose, and 198 μL aliquots of the diluted culture were added to each of the wells of the microtiter plate containing compound. For the *B. subtilis* receiver and full cells containing the *S. epidermidis* *agr* systems, overnight cultures were grown for 20 h and diluted 50-fold with fresh TSB medium, supplemented with 0.01% w/v xylose, and 198 μL aliquots of the diluted culture were added to each of the wells of the microtiter plate containing compound. Plates were then incubated at 37 °C for 24 h at 750 rpm on a Stuart SI505 microtiter plate shaker incubator, and the fluorescence (Excitation 500 nm/Emission 540 nm) and OD_600_ of each well were measured using a Biotek Synergy 2 plate reader running Gen5 3.12 software. Data were normalized to growth and media controls, and some full cell data was normalized to the vehicle (100%) and media (0%) controls. Data was analyzed using GraphPad Prism software (v. 10.3.1) to generate sigmoidal curve fits ([compound] vs response, four-parameters). Significance testing was performed using one-way or two-way ANOVA with Šídák’s multiple comparisons test or an unpaired *t* test. Differences with **, ***, and **** were deemed significant with *p*_adj_ < 0.01, *p*_adj_ < 0.001, and *p*_adj_ < 0.0001, respectively. Control *B. subtilis* cells that contain the xylose-inducible vector with the *agr* gene removed, but with the P3-GFP readout component present (pSa-control-GFP, pSe-control-GFP, pLm-control-GFP), were tested and did not show significant GFP production (Figure S3).

### Mass Spectrometric Analysis of Supernatants for AIP Detection

*B. subtilis* supernatant samples were isolated as described above. Following centrifugation and filtration, samples were acidified with trifluoroacetic acid (TFA) to 0.1% and spiked with an internal standard (*S. aureus* AIP-III + N-terminal alanine; sequence AIN-(CDFLL)) to 5 μM before solid-phase extraction (SPE) of native AIPs. SPE of AIP was conducted as described previously with minor modifications.^11^ Briefly, C18 SPE columns (SiliCycle SiliaPrep SPE Cartridges—C18 (17%) poly) were activated with 3 × 3 mL of a 10%/90%/0.1% mixture of H_2_O/CH_3_CN/TFA. Columns were then equilibrated with 2 × 3 mL of 0.1% (v/v) TFA in water. Supernatant samples were loaded onto the column and washed with 5 mL of a 91%/9%/0.1% mixture of H_2_O/CH_3_CN/TFA. Peptides were eluted with 5 mL of a 10%/90%/0.1% mixture of H_2_O/CH_3_CN/TFA and lyophilized overnight prior to LC-MS analysis.

All mass spectrometry (MS) analyses were conducted using a Q-Exactive Focus ESI-Q-Orbitrap Mass Spectrometer (Thermo Scientific) equipped with an UltiMate 3000 HPLC (Thermo Scientific) and diode array detector. Dried SPE eluent was resuspended in 50% HPLC grade CH_3_CN in water such that the final eluted sample was concentrated 10× (relative to initial supernatant sample) and filtered using a 0.45 μM syringe filter (PerkinElmer Red PTFE (Hydrophilic) Syringe Filter). Concentrated samples (5 μL) were injected onto an Agilent PLRP-S 300A (50 mm × 2.1 mm) column using a 10–60% gradient of CH_3_CN in water with 0.1% formic acid (FA) over 8 min. The masses of native AIPs and internal standard were added to the inclusion list as precursor ions for parallel reaction monitoring (PRM). Extracted ion chromatograms (MS^1^) of *S. aureus* AIP-I ([M + H]^+^ = 961.3794), *L. monocytogenes* 6-mer ([M + H]^+^ = 699.3000), and the internal standard ([M + H]^+^ = 890.4440) were integrated. Peaks were integrated within a 5-ppm window around the calculated mass and normalized to the internal standard peak area. *S. aureus* AIP-I and *L. monocytogenes* 6-mer were quantified using a standard curve generated from authentic samples of synthetic AIP (Figure S4). Standard curve data were fitted to a simple linear regression model. All peptide masses and identities were confirmed by matching parent *m*/*z*, MS^2^ (assisted by PRM), and retention time to synthetic standards. *S. aureus* AIP-I produced by *B. subtilis* showed relatively poor fragmentation under these conditions; only four fragment ions were used to confirm its identity.

### *B. subtilis* Biosensor Zone of Inhibition Assays

Overnight cultures of WT *B. subtilis* 168 and the lysostaphin *B. subtilis* construct (*Sa*RC-LysT) were grown in LB medium and supplemented with no antibiotic or erythromycin (1 μg/mL) + lincomycin (25 μg/mL), respectively. Overnight cultures of *S. aureus* (LAC, LAC Δ*agr*, RN6925, MW2, and RN4850) or *S. epidermidis* (RP62A, 1457, and 8247) were grown in TSB medium. The overnight cultures were then diluted 1:500, and a 200-μL aliquot of culture was spread evenly with a sterile swab on tryptic soy agar plates (100 × 15 mm) supplemented with 0.1% xylose and allowed to dry. The cultures of *B. subtilis* 168 and *Sa*RC-LysT were pelleted via centrifugation (13,200 rpm for 1 min) and washed twice in 1 mL of PBS to remove antibiotics from the supernatant. Aliquots (5 μL) of washed *B. subtilis* 168 and *Sa*RC-LysT cells were spotted onto the lawn and allowed to dry. Plates were incubated statically at 37 °C for 24 h, after which they were imaged (using an iPhone 14 camera), and zones were measured using ImageJ. Significance testing was performed analogously to that described for the *B. subtilis* fluorescent reporter assays.

### *B. subtilis* Biosensor MIC Supernatant Assays

Overnight cultures of *S. aureus* LAC were grown in TSB medium at 37 °C for 20 h at 200 rpm. *B. subtilis* constructs (*Sa*RC-LysT and controls) were grown in LB medium supplemented with various amounts of xylose in either the presence or absence of 1 μM *S. aureus* AIP-I at 37 °C for 20 h at 200 rpm. An additional culture of WT *B. subtilis* 168 was grown in LB medium under the same conditions. All *B. subtilis* cultures were pelleted via centrifugation for 2 min at 13,200 rpm. Supernatants were collected and filtered through a 0.22 μm syringe filter. *S. aureus* LAC overnight cultures were then diluted 1:1000 in fresh TSB medium, and 50 μL aliquots were transferred to the wells of a clear 96-well microtiter plate (Costar) along with 50 μL of *B. subtilis* supernatant. Plates were incubated statically at 37 °C for 24 h before the OD_600_ of each well was measured on a Biotek Synergy 2 plate reader running Gen5 3.12 software. Supernatants were tested in five biological replicates in technical duplicate. Measurements were background corrected and normalized to a 100 μL *S. aureus* LAC control (grown in the absence of *B. subtilis* supernatant). Data were analyzed and plotted using GraphPad Prism software (v. 10.4.2). Significance testing was performed analogously to that described for the *B. subtilis* fluorescent reporter assays.

## Results and Discussion

### Development of a *S. aureus* *agr* “Sender” System in *B. subtilis*

We began by cloning *S. aureus* *agrBD* into *B. subtilis* to yield “sender" cells (referred to hereafter as *Sa* sender cells for brevity) capable of biosynthesizing AIP. To start, the *S. aureus* *agrBD* gene was amplified using PCR from extracted genomic *S. aureus* DNA (group-I, strain RN6390B), added to the backbone vector (pBS0ExylRP_xylA(V2)_), and placed under control of a xylose-inducible promoter, using Gibson Assembly methods.^53^ A *B. subtilis* ribosomal binding site was added using primers directly preceding the *agr* genes, generating the final pXyl-Sa-agrBD vector. Sequence-confirmed vectors were then transformed into *B. subtilis* 168 cells (see Experimental section for full details of methods and plasmid construction).^55^ Work from the Collins group showed that expression of the *agrBD* genes in both *E. coli* and *P. megaterium* yielded supernatants (assumed to contain native AIP-I) capable of activating “receiver” cells containing the *agrCA* genes, suggesting that the final AIP N-terminal processing steps (performed by MroQ in *S. aureus* group-I) are likely performed by either an analogous protease or one(s) with similar specificity in these heterologous hosts.^33,34^ At the outset, we could not discount the possibility that AIP products with various tail lengths would be generated, and that such signals could cause a response, as *S. aureus* AgrC-I has been shown to be activated by AIP-I analogs with varied N-terminal tails.^56^

To test if the *Sa* sender cells were producing AIP-I, supernatant from sender cells induced with xylose and grown for 6–8 h was isolated, concentrated, serially diluted, and subsequently added to cultures of a *S. aureus* group-II *agr* reporter strain, AH430, which reads out *agr* activity as fluorescence (i.e., production of YFP is under control of the *agr*-regulated P3 promoter). AIP-I is known to strongly inhibit group-II *agr*.^18^ Thus, if *S. aureus* AIP-I was being produced at sufficient levels by the *Sa* sender cells, the supernatant should inhibit *agr* in this reporter strain. As anticipated, we observed a dose-dependent inhibition of group-II *agr* upon the addition of the *Sa* sender cell supernatant (Figure 3A), reaching 96% inhibition at a supernatant concentration of 250% (unconcentrated supernatant set to 100%).

**Figure 3:**
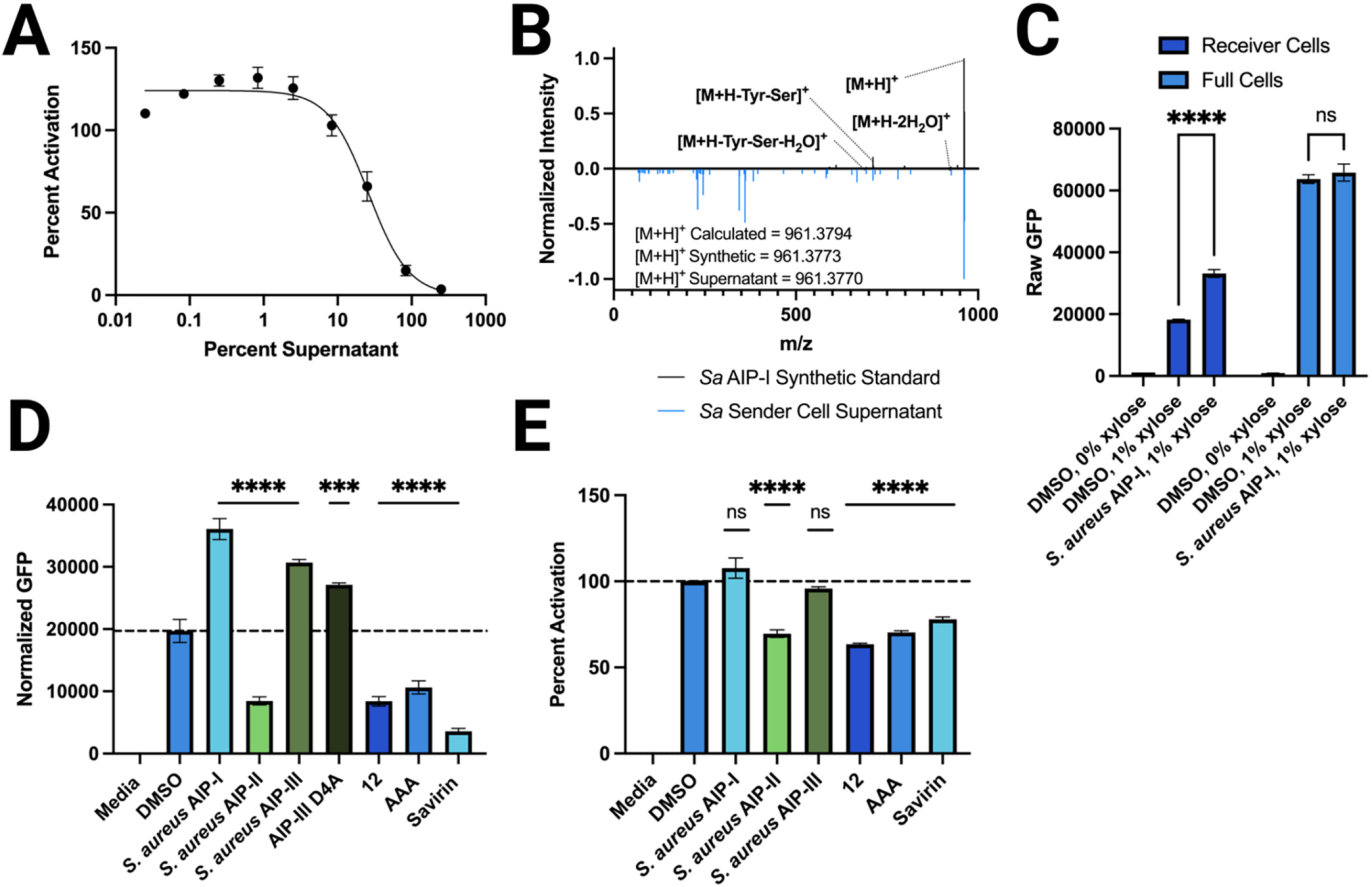
Characterization of the engineered *B. subtilis* strains containing the *S. aureus* *agr* QS system. (A) Dose–response of *Sa* sender cell supernatant in a *S. aureus* group-II *agr* fluorescent reporter strain (AH430). Data normalized to media (0% activity) and WT *B. subtilis* 168 supernatant (100% activity) controls. (B) Mirror plot of MS^2^ spectra comparing *Sa* sender cell supernatant (blue, RT = 6.30 min) compared to a synthetic *S. aureus* AIP-I standard (black, RT = 6.30 min). Labeled mass identities represent peaks identified in both samples. (C) Fluorescence readout of the *Sa* receiver and *Sa* full cells with and without xylose induction, and upon the addition of 10 μM *S. aureus* AIP-I. (D) Activity data for a panel of QS inhibitors (and native agonist AIP-I) in *Sa* receiver cells. All concentrations are 10 μM. Dotted line represents vehicle fluorescence for ease of comparison. Fluorescence normalized to growth and media controls. Activity not reported as percent activation in panels (C) or (D) due to the lack of basal AIP production in the receiver cells that is usually standardized as “100% activity.” (E) Activity data for a panel of QS inhibitors (and native agonist AIP-I) in *Sa* full cells. All concentrations are 10 μM. Dotted line represents vehicle fluorescence for ease of comparison. Fluorescence normalized to media (0% activity) and DMSO (100% activity) controls. Error bars in each plot indicate the standard error of the mean (SEM) of at least three biological replicates, each tested in technical triplicate (*n* = 3). Significance values represent comparisons to DMSO control. See Experimental section for full details of methods.

For further verification of native AIP-I production, we analyzed *Sa* sender cell supernatants using a liquid chromatography-tandem mass spectrometry (LC-MS/MS) method and compared the results to a *S. aureus* AIP-I synthetic standard. We observed that the major peak in the *Sa* sender cell supernatant matched the retention time, molecular ion mass to charge ratio (*m*/*z*), and MS/MS fragmentation patterns of the AIP-I standard (Figure 3B, and S5). A standard curve of synthetic *S. aureus* AIP-I (Figure S4) was used to quantify the amount of AIP in sender cell supernatant (at 8 h), and the sender cells were found to be producing approximately 11 nM AIP-I. While the major peak in the *Sa* sender cell supernatant corresponded to AIP-I, an additional peak was also observed corresponding to the *m*/*z* of the AIP plus an extra alanine residue at the N-terminus (sequence AYST-(CDFIM), [M + H]^+^ = 1032.4165), indicating a mis-cleaved product. [It is possible that the activity of this minor mis-cleavage product is contributing to the overall activity profile of our *Sa* constructs; however, similar N-terminal extensions on AIP-I as observed in this byproduct have been shown to activate AgrC-I to similar levels as the native AIP.^56^ Therefore, we do not anticipate the production of this minor byproduct to confound interpretation of QSM activity in this *Sa* system.] Masses of AIP-I and its mis-cleaved byproduct were only observed in cells containing the *agrBD* construct and not in the *B. subtilis* 168 background control, suggesting these masses correspond to peptide products of AgrD processing and not endogenous *B. subtilis* metabolites.

For comparison of sender cell vs native AIP production, supernatants of *S. aureus* USA300 LAC (group-I) cells grown for 6–8 h were similarly analyzed by LC-MS/MS. The *S. aureus* cells were found to produce significantly more AIP-I (347 nM) than the *Sa* sender cells (∼30-fold). Additionally, no major mis-cleavage products were observed in the *S. aureus* supernatant. This latter result suggests that while there is a protease in the *B. subtilis* sender cells capable of performing the N-terminal cleavage of the pro-AIP like MroQ in *S. aureus*, it may not operate with the same precision. We note that this cleavage step in the sender cells could be performed by the putative MroQ homologue in *B. subtilis*, YdiL,^57^ a different protease, or multiple proteases.

### Development of a *S. aureus* *agr* “Receiver” System in *B. subtilis*

We next generated *S. aureus* “receiver" cells (*Sa* receiver cells for brevity) in *B. subtilis*, using similar cloning methods to those described above yet containing the *S. aureus* *agrCA* signal transduction system genes instead of the *agrBD* genes. Within the same vector, a *B. subtilis*-optimized GFP gene was added under control of the *S. aureus* P3 promoter using a Gibson Assembly to provide a means for measuring AgrCA activity (see Experimental section). We first examined fluorescence in the *Sa* receiver cells in the presence and absence of xylose, and with the native signal AIP-I (Figure 3C). As expected, minimal fluorescence was observed in the absence of xylose. Upon the addition of xylose, a significant increase (16-fold) in fluorescence was observed, primarily due to basal levels of AgrA binding to the P3 promoter (as determined by examination of a receiver cell control containing only *agrA*, Figure S6). Upon the addition of 10 μM AIP-I, fluorescence was further increased approximately an additional 2-fold, indicating activation of the AgrCA system (Figure 3C).

A dose–response experiment was performed in the *Sa* receiver cells, showing a dose-dependent increase in fluorescence upon addition of increasing concentrations of AIP-I (Figure S7). Interestingly, the curve was flatter than typical sigmoidal dose–response curves in *S. aureus* *agr* reporter systems (with a Hill slope of 0.57), suggestive that AgrC receptor cooperativity could be different in the non-native background. The only reported analysis of *S. aureus* AgrC cooperativity was, to our knowledge, performed by the Muir lab in 2014, where they examined the response of AgrC-I isolated in nanoscale lipid discs (“nanodiscs”) to various ligands.^14^ In these prior *in vitro* experiments, AgrC-I was found to lack cooperativity upon activation by AIP-I. We note that the flatter dose–response curve observed in our *B. subtilis* receiver cells relative to *S. aureus* could occur for several reasons other than cooperativity differences, including the different expression levels of AgrC or AgrA, as overproduction of protein could result in a shift in the target to inhibitor ratio, and/or protein structure differences in the heterologous background (see SI for additional data (Figure S8) and discussion).

We next tested a panel of known *S. aureus* group-I *agr* inhibitors in the *Sa* receiver cells to ascertain whether the compounds maintained their activity profiles in the non-native background, including the native peptide inhibitors AIP-II and AIP-III,^18^ the non-native peptide inhibitors **AIP-III D4A** (an inhibitor based on the native AIP-III scaffold, **D4A** for brevity),^22^ **12**, and **AAA** (two derivatives of *S. epidermidis* native AIPs that are potent and efficacious pan-group inhibitors in both *S. epidermidis* and *S. aureus*),^25^ and the small molecule inhibitor savirin^21^ (see Figure 2 for structures and Table S3 for compound activity in a WT *S. aureus* *agr* group-I reporter). These peptidic compounds are all believed to act on AgrC, while savirin has been shown to act on AgrA. We observed that the majority of these compounds decreased fluorescence in the *Sa* receiver cells, as expected (Figure 3D). However, the resulting fluorescent signals were below basal (DMSO vehicle) levels, suggesting that these inhibitors have inverse agonist activity profiles in this *B. subtilis* system. This inverse agonist profile for *S. aureus* AIP-II has been observed before by the Muir lab in their *in vitro* studies of AgrC-I.^14^ Interestingly, two of the inhibitors, the native AIP-III and **D4A**, increased fluorescence *above* vehicle levels, albeit less so than the native agonist AIP-I (AIP-III and **D4A** with 1.55-fold and 1.37-fold activation, respectively, vs AIP-I with 1.8-fold activation above basal DMSO activity). There is some precedence for this unexpected agonistic behavior for these two compounds, again in the context of *in vitro* experiments. Again, the Muir lab reported activation of AgrC-I *in vitro* upon the addition of the inhibitors AIP-III and AIP-I D5A (an inhibitor based on the AIP-I scaffold, but with a similar alanine substitution as in **D4A**).^58^ This 2015 result is congruent with our findings here in the *Sa* receiver system and, along with the observed inverse agonism profile for at least AIP-II, suggests that certain *agr* modulators are interacting with the AgrC-I receptor similarly in the non-native *B. subtilis* and *in vitro* nanodisc systems, yet differently relative to the native environment of the *S. aureus* *agr* reporters. We note that the native *S. aureus* membrane is more negatively charged and composed of different ratios of charged lipids and other membrane components relative to that of *B. subtilis*,^14,59^ potentially contributing to the observed differences in compound activity.

### Development of a *S. aureus* *agr* “Full” System in *B. subtilis*

Building on the results above, *B. subtilis* producing the “full” *S. aureus* *agr* system was developed and characterized (i.e., *Sa* full cells). These *B. subtilis* cells contained the entire *S. aureus* *agrBDCA* operon and the P3-controlled GFP readout system within the same vector (pXyl-Sa-agrBDCA-GFP), and were generated using similar cloning methods to the *Sa* sender and receiver cells. It is worth noting that these cells should mimic *S. aureus* *agr* reporter cells, with the ability to biosynthesize the native signal, sense QSMs, and read out their *agr* activity via fluorescence. The primary difference between the *agr* circuits in the *B. subtilis* full cells and the native *S. aureus* background is the use of xylose to induce *agr* expression in *B. subtilis*, as opposed to the cell density-induced positive QS feedback loop via the P2 promoter present in *S. aureus*. Thus, the QS dynamics and *agr* machinery production differ between the engineered *B. subtilis* strain and *S. aureus* as cell density changes.

Control experiments were performed to test the activity of the *Sa* full cells with and without the addition of xylose and with *S. aureus* AIP-I. As observed for the receiver cells, minimal fluorescence was observed for uninduced cells (Figure 3C). Upon addition of xylose, a large increase in fluorescence was observed after 24 h, approximately double of that observed in the *Sa* receiver cells with added AIP-I. This result indicates that the full cells are both producing AIP and capable of receiving the native signal. Interestingly, a significant increase in fluorescence was not observed upon the addition of exogenous AIP-I (up to 10 μM) after 24 h, potentially indicating that all AgrC binding sites are already saturated with AIP-I produced endogenously by the full cells at that time point.

The same panel of *agr* inhibitors that was tested in the *Sa* receiver cells was also tested in the *Sa* full cells (Figure 3E). Again, inhibition was generally observed across the board, with all AgrC inhibitors (aside from AIP-III) and the AgrA inhibitor savirin demonstrating statistically significant decreases in activity below the vehicle control (i.e., inverse agonist behavior). Select inhibitors were subjected to dose–response studies in the *Sa* full cells and found to block fluorescence in a concentration-dependent manner (Figure S7). No inhibition was observed for AIP-III, as observed in the *Sa* receiver cells, yet no activation was observed either (fluorescence similar to vehicle). At the 1% xylose condition tested, unexpected growth effects were observed for **D4A** in the *Sa* full cells that were not observed for any other compound. When the cells were induced at a lower xylose concentration (0.1%), these growth effects were no longer observed for **D4A**, but reduced inhibition levels were observed for all compounds (except for AIP-III (which was inactive) and savirin, Figure S9). Overall, the results so far demonstrated that these engineered *B. subtilis* strains can produce *S. aureus* AIP-I (via the sender and full strains) and respond to both native AIP-I and known *agr* inhibitors (via the receiver and full strains) and served to validate our design of heterologous *S. aureus* *agr* systems in *B. subtilis*.

### Application of the Engineered *B. subtilis* System to the *S. epidermidis* *agr* Circuit

We were interested in whether this *B. subtilis* system could be applied to other *agr* circuits and turned our attention to *S. epidermidis*. The analogous combination of sender, receiver, and full cells were generated as for *S. aureus*, but now containing the group-I *S. epidermidis* *agrBD*, *agrCA*, and *agrBDCA* genes on the plasmids pXyl-Se-agrBD, pXyl-Se-agrCA-GFP, and pXyl-Se-agrBDCA-GFP (Table 1), respectively, using the same cloning methods as described above. In addition, the *S. epidermidis* P3 promoter was used to control GFP production in the receiver and full cells, as opposed to the *S. aureus* P3 promoter.

First, the cells containing the *S. epidermidis* *agrBD* genes were tested (i.e., the *Se* sender cells). In a similar manner to the *Sa* sender cells, the supernatant from the induced *Se* sender cells was isolated after 6–8 h, concentrated, and added to the *S. aureus* group-I *agr* fluorescence reporter, AH1677.^46,47^ *S. epidermidis* AIP-I is known to inhibit *S. aureus* group-I *agr* (to 92.3%, IC_50_ = 74.4 nM),^25^ and thus if AIP-I was being successfully produced by the *Se* sender cells, its supernatant should inhibit *agr* in this reporter strain. While inhibition was observed, the level of inhibition was significantly less than that observed for the engineered *Sa* sender cells (Figure 4A), only reaching 34% inhibition at the highest concentration of supernatant tested. Furthermore, neither *S. epidermidis* AIP-I nor AIP miscleavage products could be detected in *Se* sender cell supernatant using analogous LC-MS/MS methods as described above for the *Sa* sender system. These results suggest that the *Se* sender cells may produce AIP-I (and/or miscleavage products) but at significantly lower levels than the AIP-I signal produced by the *Sa* sender cells. This result could be for a number of reasons. For example, AgrB/AgrD could be expressed at different levels in the *Sa* vs *Se* sender cells, or *S. epidermidis* AgrB/AgrD are less functional in the non-native membrane environment relative to *S. aureus* AgrB/AgrD. Alignment of the AgrB sequence from *S. aureus* and *S. epidermidis* used in the *Bacillus* systems shows 76% sequence similarity and 53% sequence identity, suggestive of some potential differences (Figure S10). Alignment of the two AgrD sequences shows 65% sequence similarity and 53% identity, with much of the variability existing in the N-terminal and AIP sequence regions (Figure S10). This variation in AgrD could cause the N-terminal leader sequence of *S. epidermidis* AgrD to associate differently with the *B. subtilis* membrane relative to *S. aureus* AgrD, leading to less cleavage and AIP production. Another reason for low AIP production could be because the (unknown) protease(s) responsible for the final N-terminal cleavage in AIP biosynthesis in the *B. subtilis* background are less facile at processing the *S. epidermidis* pro-AIP compared to the *S. aureus* pro-AIP.

**Figure 4:**
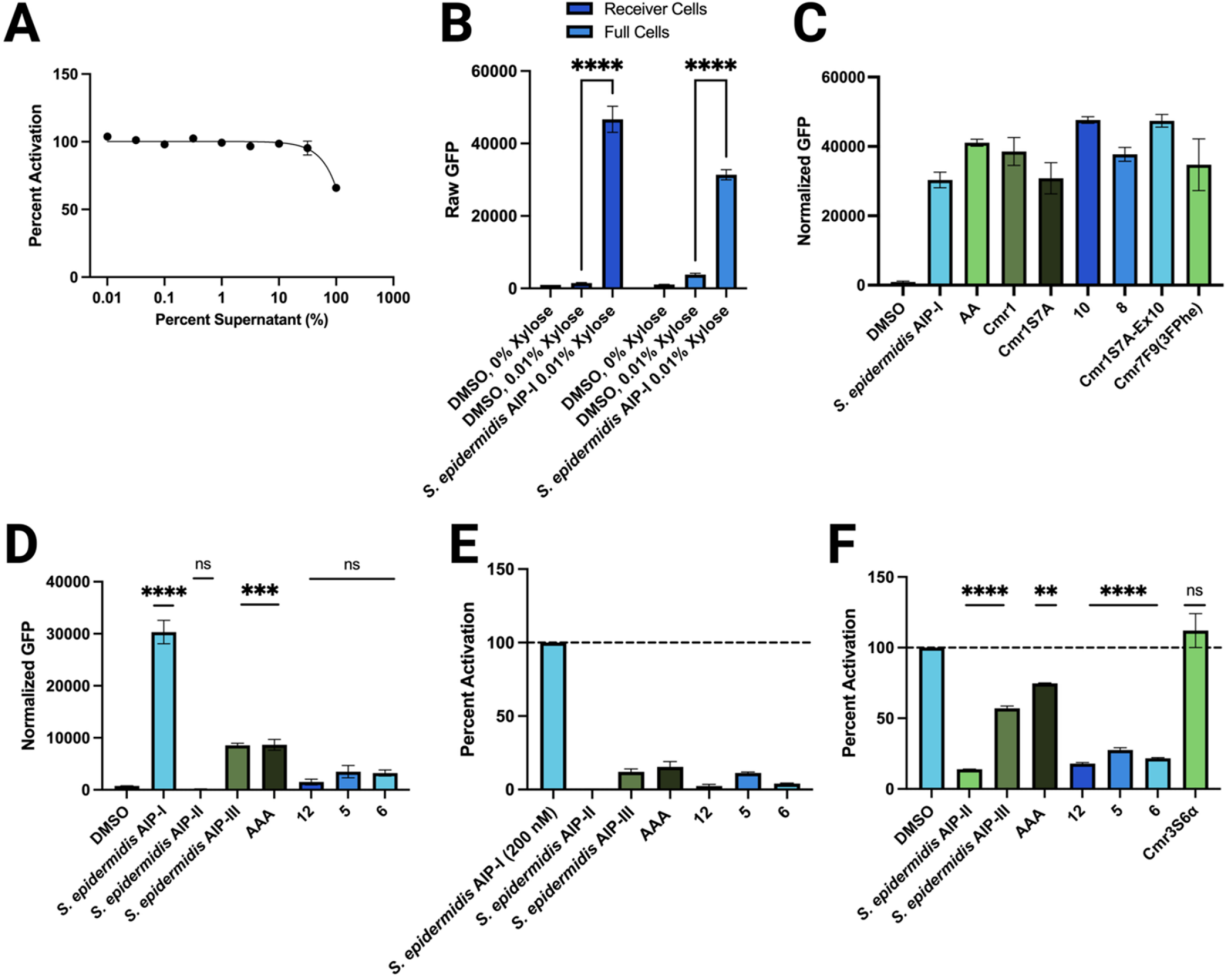
Characterization of the engineered *B. subtilis* strains containing the *S. epidermidis* *agr* QS system. (A) Dose–response of *Se* sender cell supernatant in a *S. aureus* group-I *agr* fluorescent reporter (AH1677). Data normalized to media (0% activity) and WT *B. subtilis* 168 supernatant (100% activity) controls. (B) Fluorescence readout of the *Se* receiver and full cells with and without xylose induction, and upon the addition of 1 μM *S. epidermidis* AIP-I. (C) Activity data for a panel of QS activators in *Se* receiver cells. All concentrations are 1 μM. For panels (C–F), fluorescence was normalized to growth and media controls. (D) Activity data for a panel of QS inhibitors (and native agonist AIP-I) in *Se* receiver cells. All concentrations are 1 μM. Activity not reported as percent activation in panels (B–D) due to the lack of basal AIP production in the receiver cells that is usually standardized as “100% activity”. (E) Activity data for a panel of QS inhibitors competed against 200 nM AIP-I in *Se* receiver cells. All inhibitor concentrations are 1 μM. Data normalized to media (0% activity) and 200 nM AIP-I (100% activity). (F) Activity data for a panel of QS inhibitors and the inactive compound Cmr3S6α in *Se* full cells. All concentrations are 1 μM. Dotted line represents vehicle fluorescence for ease of comparison. Error bars in each plot indicate the standard error of the mean (SEM) of at least three biological replicates, each tested in technical triplicate (*n* = 3). Significance values represent comparisons to DMSO control. See Experimental section for full details of methods.

Next, we examined the receiver cells in *B. subtilis* containing the *S. epidermidis* AgrCA machinery and the fluorescent readout system under control of the P3 promoter (i.e., *Se* receiver cells). The receiver cells were first tested with and without xylose induction and with the native *S. epidermidis* AIP-I (Figure 4B). As observed for the *Sa* receiver system, minimal fluorescence was observed in the absence of xylose. In contrast to the *Sa* system, however, very little background fluorescence was observed upon xylose induction, potentially indicating that the basal level of AgrA binding to the P3 promoter is much lower in this *Se* receiver construct relative to the *Sa* system (as supported by examination of a receiver cell control containing only *S. epidermidis* *agrA*, Figure S6). Upon the addition of 1 μM *S. epidermidis* AIP-I to the *Se* receiver cells, a significant increase (over 30-fold) in fluorescence was observed, congruent with the AgrC and AgrA proteins being present and functional.

The *Se* receiver cells next were tested against a panel of known peptidic *S. epidermidis* *agr* agonists, all of which are believed to act on AgrC. Unlike *S. aureus*, a significant number of non-native peptide-based *agr* agonists have been developed for *S. epidermidis* (e.g., **AA**, **Cmr1**, **Cmr1S7A**, **10**, **8**, **Cmr1S7A-Ex10**, and **Cmr7F9­(3FPhe)**, structures in Figure 2),^24,25^ and we selected agonists for study capable of a wide range of *agr* activation levels (from 40 to 182%, as demonstrated in *S. epidermidis* *agr* reporters; see Table S3). Significant activation was observed in the *Se* receiver cells for all agonists tested at 1 μM (Figure 4C). Only minimal activity differences were observed between these compounds at this concentration; however, full dose–response curves of select compounds demonstrated clear discrimination between the compounds by the receiver cells (Figure S7C). These differences in activation levels followed trends similar to those observed in the *S. epidermidis* fluorescent reporter system (Table S3 and Figure S7C).^25,26^

We also tested a panel of known peptidic *S. epidermidis* *agr* inhibitors (*S. epidermidis* AIP-II and AIP-III, **AAA**, **12**, **5**, and **6**; structures in Figure 2) in the *Se* receiver cells with high inhibitory efficacies in *S. epidermidis* *agr* reporters (87–97%) (Table S3).^25^ As expected, the majority of these compounds failed to show a statistically significant difference from the DMSO control and demonstrated significantly less activation than the native activator AIP-I, even at up to 1 μM. In addition, and similar to our results in the *Sa* receiver system, certain native AIPs or very close analogs, such as AIP-III and **AAA** (an inhibitor based on the *S. epidermidis* native AIP-I with threealanine substitutions) significantly *activated* above the DMSO control (Figure 4D). We sought to further probe whether these compounds were acting as inhibitors, as baseline activity in this receiver system could also simply indicate inactivity. We performed the same inhibition assay in the *Se* receiver cells but competed all the compounds against 200 nM AIP-I (a concentration well above that necessary for full activation) and, in contrast to the nonactivated *Se* receiver cell data, observed significant decreases in activity for all the compounds compared to 200 nM AIP-I alone (Figure 4E). Thus, significant activation of the system is needed to observe inhibitory activity of compounds, mimicking the native *S. epidermidis* systems in which the compounds were originally characterized.^24–26^ Overall, these results indicate that the panel of *S. epidermidis* *agr* inhibitors are capable of significant antagonism in the *Se* receiver cells.

We proceeded to generate and characterize a *B. subtilis* strain that contained the full *S. epidermidis* *agrBDCA* operon and the P3-GFP reporter plasmid (i.e., *Se* full cells). Control experiments were performed testing these cells with and without the addition of xylose and with *S. epidermidis* AIP-I (Figure 4B). As expected, and analogous to the *Se* receiver cells, minimal fluorescence was observed for uninduced cells. Upon addition of xylose, a significant increase (over 3-fold) in fluorescence was observed, indicative of a functional *agr* system. The fluorescence signal increased approximately 8-fold upon addition of 1 μM *S. epidermidis* AIP-I. This result was markedly different than that observed for the *Sa* full cells (Figure 3C), which exhibited no significant difference in fluorescence upon addition of *S. aureus* AIP-I, and is likely explained by the significantly lower AIP production observed in the *Se* sender cells compared to their *Sa* counterparts. These results could be congruent with the observed ability of *S. epidermidis* fluorescent *agr* reporters to activate over basal activation (set as 100%),^25^ a trend that is not observed in analogous *S. aureus* *agr* reporters.^60^

Lastly, the same set of *S. epidermidis* *agr* inhibitors tested in the *Se* receiver cells were tested in the *Se* full cells, along with peptide **Cmr3S6α**, which we have shown to have no measurable activity in *S. epidermidis* *agr* reporter strains and thus served as a valuable control.^24^ Every compound other than **Cmr3S6α** showed significant inhibition below vehicle levels of fluorescence in the full cells (Figure 4F). The trends in relative levels of inhibition in the *Se* full cells were identical with those observed in the *Se* receiver cells with added AIP-I (Figure 4E), with the strongest inhibition observed for *S. epidermidis* AIP-II and the least inhibition observed for **AAA**. Dose–response curves were collected on select inhibitors in the full cell system, and the trends in relative potencies matched those previously reported in *S. epidermidis* fluorescent *agr* reporters, although greater differences in efficacies were observed in the *Se* full cells (Figure S7).^25^

Overall, the results for the engineered *B. subtilis* *Se* cells illuminated some significant differences between how the *S. aureus* and *S. epidermidis agr* components behave in this heterologous background, particularly basal AIP production levels (low in *S. epidermidis* vs *S. aureus*) and background activity of the xylose-induced system prior to compound addition (low in *S. epidermidis* vs *S. aureus*). These observed differences between the two systems can be interpreted as potential intrinsic differences in the native *agr* signal networks in these closely related species; we return to this below. Nevertheless, the receiver and full cells for both *S. aureus* and *S. epidermidis* provide robust responses to both *agr* activators and inhibitors, further validating the design of these heterologous *agr* systems and underscoring their potential utility.

### Application of the Engineered *B. subtilis* System to the *L. monocytogenes* *agr* Circuit

We were interested to study *agr* circuits beyond Staphylococcal species and expanded our approach to *L. monocytogenes*, a pathogen that poses a major threat to human health but has an underexplored *agr* QS system relative to *S. aureus* and *S. epidermidis*. *B. subtilis* sender, receiver, and full cells were constructed containing the AgrBD, AgrCA, and AgrBDCA machinery from *L. monocytogenes* (using plasmids pXyl-Lm-agrBD, pXyl-Lm-agrCA-GFP, and pXyl-Lm-agrBDCA-GFP, Table 1), respectively, and analogous cloning techniques as described above. As with previous constructs, the *L. monocytogenes* P3 promoter was added in front of the GFP gene for fluorescence readout of *agr* activity.

Similar to the two systems above, *L. monocytogenes* sender cells were created by adding the *L. monocytogenes* *agrBD* AIP biosynthesis genes to *B. subtilis* under control of a xylose-inducible promoter (*Lm* sender cells). Sender cell supernatant was isolated, concentrated, and added to a *S. aureus* group-I fluorescence reporter strain, AH1677 (Figure 5A), to gauge AIP production. Based on prior studies of the native *L. monocytogenes* 6-mer AIP in *S. aureus*, if this signal was successfully produced in *B. subtilis*, the supernatant should inhibit *agr* in *S. aureus* group-I cells.^27^ Significant inhibition was indeed observed in the *S. aureus* group-I *agr* reporter, reaching up to 75% inhibition at the highest concentration of supernatant tested. LC-MS/MS analyses of supernatant were performed to verify AIP biosynthesis. The major peak in the *Lm* sender cell supernatant matched the retention time, molecular ion mass to charge ratio (*m*/*z*), and MS/MS fragmentation patterns of an authentic 6-mer AIP standard (Figures 5B and S5). The fragmentation pattern also matched that of supernatant from a wild type *L. monocytogenes* strain in a previous report.^27^ There have been differing reports on the structure of the native *L. monocytogenes* AIP,^27,61–63^ and our results here are congruent with the 6-mer AIP serving as a native signal. A standard curve of synthetic *L. monocytogenes* 6-mer (Figure S4) was used to quantify the amount of AIP in sender cell supernatant (at 8 h), and the sender cells were found to be producing approximately 12 nM AIP. WT *L. monocytogenes* was found to produce only a ∼3-fold higher level of AIP (34 nM) relative to the *Lm* sender cells (at 8 h), demonstrating that the AIP production level in the *Lm* construct was more similar to the native host than observed for the *Sa* or *Se* constructs. These results indicate that the *Lm* sender cells are capable of robust native AIP biosynthesis.

**Figure 5:**
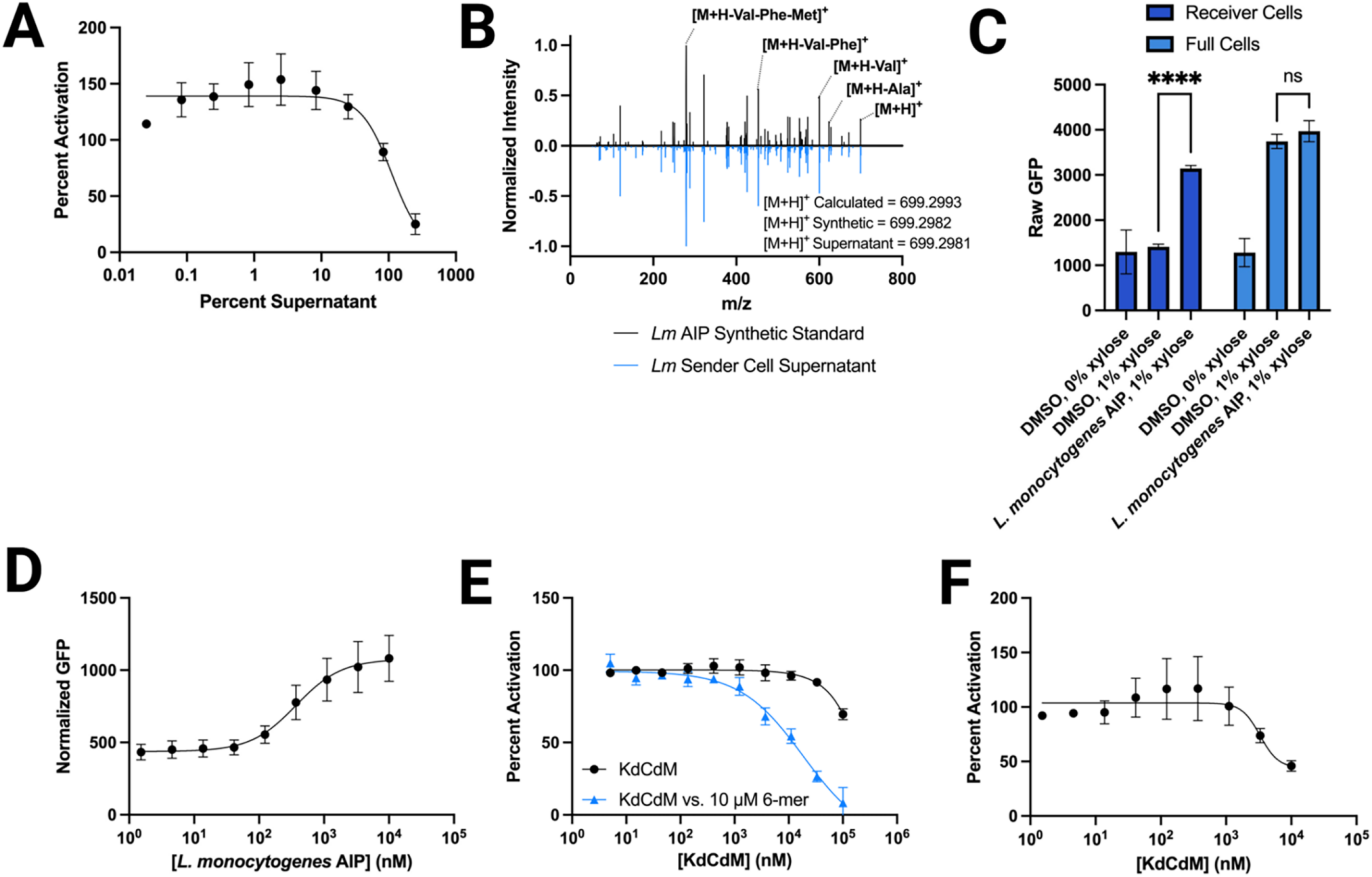
Characterization of the engineered *B. subtilis* strains containing the *L. monocytogenes* *agr* QS system. (A) Dose–response of *Lm* sender cell supernatant in a *S. aureus* group-I *agr* fluorescent reporter (AH1677). Data normalized to media (0% activity) and WT *B. subtilis* 168 supernatant (100% activity) controls. (B) Mirror plot of MS^2^ spectra comparing *Lm* sender cell supernatant (blue, RT = 7.18 min) compared to a synthetic *L. monocytogenes* 6-mer AIP standard (black, RT = 7.18 min). Labeled mass identities represent peaks identified in both samples. (C) Fluorescence readout of the *Lm* receiver and full cells with and without xylose induction, and upon the addition of 10 μM 6-mer AIP. One biological replicate, consisting of three technical replicates, was conducted for these experiments. (D) Dose–response of 6-mer AIP in *Lm* receiver cells, showing a dose-dependent increase in fluorescence upon addition of compound. Fluorescence normalized to growth and media controls. Activity not reported as percent activation in panel (C) or (D) due to the lack of basal AIP production in the receiver cells that is usually standardized as “100% activity.” (E) Dose–response of inhibitor KdCdM in *Lm* receiver cells competed against 10 μM 6-mer AIP (blue curve) or alone (black curve). Fluorescence normalized to media (0% activity) and 10 μM 6-mer (100% activity for blue curve) or DMSO (100% activity for black curve). (F) Dose–response of inhibitor KdCdM in *Lm* full cells, showing a dose-dependent decrease in fluorescence as compound concentration increases. Data normalized to media (0% activity) and DMSO (100% activity) controls. Error bars for each plot indicate the standard error of the mean (SEM) of at least three biological replicates tested in technical triplicate (*n* = 3), unless indicated otherwise. See Experimental section for full details of methods.

Receiver cells containing the *L. monocytogenes* *agrCA* genes and the *L. monocytogenes* P3 promoter controlling fluorescence readout were generated and characterized (i.e., *Lm* receiver cells), again using similar approaches as introduced above. Control experiments testing the *Lm* receiver cells with and without xylose were performed, and no significant difference was observed in fluorescence upon addition of xylose (Figure 5C). However, when the native *L. monocytogenes* 6-mer AIP was added, a dose-dependent increase in fluorescence was observed (Figure 5C,5D). Notably, the normalized fluorescence levels for the *Lm* receiver cells were significantly lower than those observed for *Sa* and *Se* receiver cells, by over an order of magnitude. The origins for these lower levels are unclear; nevertheless, activation by the native AIP caused a significant change in fluorescence relative to the vehicle control. As expected, the known *L. monocytogenes* peptide inhibitor KdCdM (structure in Figure 2)^27^ showed minimal inhibitory activity alone in the *Lm* receiver cells, but demonstrated dose-dependent inhibition when the system was pre-activated with exogenous native AIP (Figure 5E).

*L. monocytogenes* full cells were next developed containing the entire *L. monocytogenes* *agrBDCA* operon and P3-controlled fluorescence readout system (i.e., *Lm* full cells). In control experiments testing these full cells with and without xylose, a significant increase in fluorescence was observed upon the addition of xylose, indicating that native AIP was being produced and activating the full cells (Figure 5C). Again, overall fluorescence levels were significantly lower than those observed for the other *B. subtilis* full systems reported in this study. Addition of the *L. monocytogenes* 6-mer AIP to the *Lm* full cells did not result in a significant difference from the vehicle (Figure 5C), indicating that this system could not be further activated, like the *Sa* full cells and unlike the *Se* full cells above. However, addition of the inhibitor KdCdM resulted in a dose-dependent decrease in fluorescence, resulting in 54% inhibition at 10 μM (Figure 5F). These results demonstrate that the family of *Lm* sender, receiver, and full cells are functional and provide evidence that this *B. subtilis* *agr* platform can be applied to Gram-positive species outside of the *Staphylococci*.

### Application of an Engineered *B. subtilis* Strain as a *S. aureus* Biosensor

Since the receiver cell systems above were all capable of sensing native AIPs, we were curious whether this system could be reconfigured to become a biosensor for pathogens producing AIPs. We selected the community-associated methicillin-resistant group-I *S. aureus* strain, LAC, as the target for biosensor development due to its high clinical relevance.^5,42^ Our goal was to design a system that could sense *S. aureus* AIP-I and in response produce a product capable of killing *S. aureus*. For this purpose, we selected lysostaphin, a bacteriolytic enzyme that targets and degrades the cell wall of Staphylococcal species (and is highly effective against MRSA),^64–66^ in view of prior work using this product in biosensor design (as introduced above).^35,37^ Construction of the biosensor strain in *B. subtilis* (termed *Sa*RC-LysT) was completed using Gibson Assembly and Phusion polymerase chain reaction. The lysostaphin gene was fused to an AmyE secretion tag (IDT) as previously reported by the Voigt lab^35^ and inserted in place of the GFP gene in the *Sa* receiver cells, resulting in a cell line that should be capable of producing and secreting lysostaphin under *S. aureus* group-I *agr* control (see Experimental section for details of plasmid construction).

To characterize the activity of the *Sa*RC-LysT construct, zone of inhibition (ZOI) and supernatant assays were conducted. A lawn of *S. aureus* LAC was grown on agar plates containing 0.1% xylose with 5 μL aliquots of *Sa*RC-LysT or WT *B. subtilis* (control) spotted onto the agar. To ensure that observed effects of *Sa*RC-LysT were due to lysostaphin expression and not plasmid-selection antibiotics, cultures of *B. subtilis* 168 and *Sa*RC-LysT were pelleted and then washed with and resuspended in PBS to remove residual antibiotics (Figure S11). The construct expressing lysostaphin produced a substantially larger ZOI when grown on *S. aureus* compared to a *S. aureus* *agr* mutant (LAC Δ*agr*; Figure 6A). This reduction in activity with the mutant supports an AIP-dependent mechanism of activation in the biosensor cells, with residual inhibition likely reflecting basal expression of lysostaphin. *S. aureus* groups-II, -III, and -IV (RN6925, MW2, and RN4850, respectively) were also plated and examined in this assay with *Sa*RC-LysT, and yielded ZOIs mirroring results from the *Sa* receiver cells, where the presence of AIP-I and AIP-III activate our system (Figures 6B and 3D; ZOI quantification in Figure S12). We note that there is a slight ZOI when *S. aureus* LAC is grown in the presence of the WT *B. subtilis* strain (Figure 6A,B). This effect is most likely attributed to *B. subtilis* production of bacilysin, a membrane associated antibiotic with weak activity against *S. aureus*.^67^

**Figure 6:**
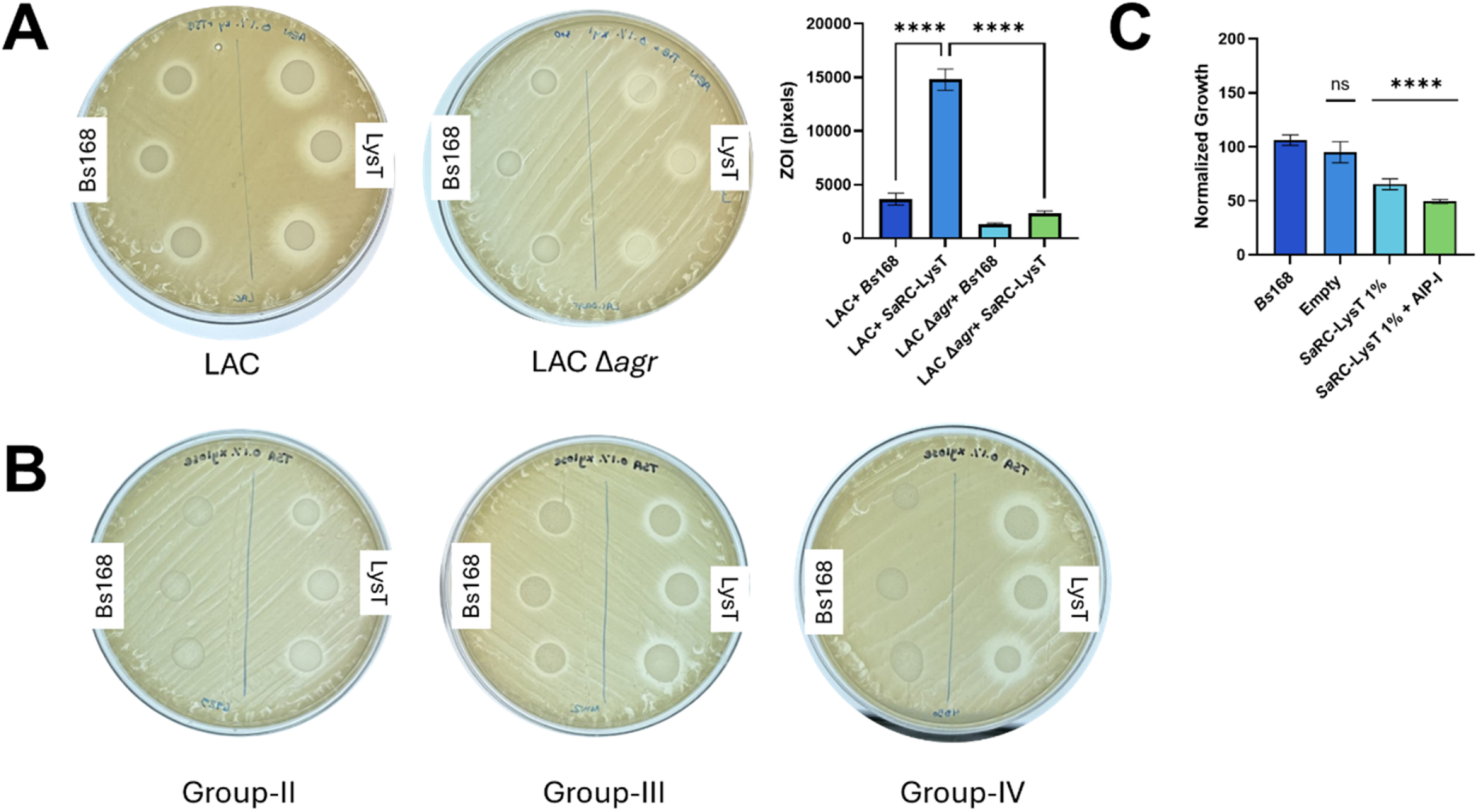
Characterization of the *Sa* receiver cell lysostaphin (LysT) construct. (A) Zone of inhibition (ZOI) assays to determine susceptibility. (*Left*) Lawn of *S. aureus* LAC grown in the presence of *Sa* receiver cell LysT or *B. subtilis* 168 (right half and left half, respectively). (*Middle)* Lawn of *S. aureus* LAC Δ*agr* grown in the presence of *Sa* receiver cell LysT construct or *B. subtilis* 168 (right half and left half, respectively). (*Right*) ImageJ quantification and analysis of ZOI data at left and middle. Error bars represent the standard error of the mean (SEM) of three biological replicates (*n* = 3). (B) *From left to right*: Lawns of *S. aureus* RN6925 (group-II), MW2 (group-III), and RN4850 (group-IV) were grown and subjected to ZOI assays as described in part A. (C) *S. aureus* LAC growth data at 20 h when supplemented with various *Bacillus* supernatants (listed on *x*-axis). Percentages refer to xylose composition in the overnight *Bacillus* cultures. Empty refers to the empty vector control. Error bars indicate the standard error of the mean (SEM) of five biological replicates, each performed in technical duplicate (*n* = 5).

Since lysostaphin is also active against *S. epidermidis*, albeit at higher concentrations,^65^ we tested *Sa*RC-LysT against *S. epidermidis* groups-I, -II, and -III. *S. epidermidis* is a normal part of the human microbiome, and an ideal biosensor strain would not respond to other natural flora. The native AIPs from *S. epidermidis* are actually known to inhibit the group-I *S. aureus* *agr* system,^25^ and ZOI assays showed that the *Sa*RC-LysT construct was largely inactive against *S. epidermidis* (Figure S13), as expected. These results are significant as they demonstrate a biosensor cell line that can sense and respond to the AIPs of multiple *S. aureus* specificity groups yet is nonresponsive to a closely related species.

To further study the effects of the product(s) secreted by the *Sa*RC-LysT, we used a supernatant-based assay in which filtered supernatants from overnight *B. subtilis* 168 or *Sa*RC-LysT were added to *S. aureus* LAC cultures and growth was monitored by measuring OD_600_ after 24 h. Only supernatants from *Sa*RC-LysT strains significantly reduced *S. aureus* LAC growth, with estimated lysostaphin concentrations of 0.63 and 1.25 μg/mL, for strains induced with 1% xylose or 1% xylose + AIP-I, respectively (lysostaphin quantified by reference to a standard curve; Figures 6C and S14). We reasoned that the growth inhibition seen for *S. aureus* LAC treated with the supernatant from *Sa*RC-LysT induced with 1% xylose alone (lightest blue bar in Figure 6C) could stem from background expression of *agr* components. Supporting this hypothesis, further ZOI analyses at various xylose concentrations showed zones of inhibition for *S. aureus* LAC treated with *Sa*RC-LysT supernatant at 0% xylose and for *S. aureus* LAC Δ*agr* treated with *Sa*RC-LysT supernatant induced with 1% xylose, indicating some leaky expression in this engineered system (Figure S15).^68^

## Summary and Conclusions

This study describes the development of a suite of engineered *B. subtilis* strains to sense and study the *agr* QS systems of Gram-positive bacteria. We generated (1) sender cells that contain the AIP biosynthesis components (i.e., the transmembrane peptidase AgrB and propeptide AgrD) and (2) receiver cells that contain the signal transduction components (i.e., the transmembrane receptor histidine kinase AgrC and response regulator AgrA) and a fluorescence reporter for AgrA activity. We first established the *S. aureus* *agr* system in *B. subtilis* as a proof-of-concept and then expanded the approach to the *agr* systems in a closely related species, *S. epidermidis*, and a more distant species, *L. monocytogenes*. We demonstrated that the *Sa* and *Lm* sender cells produced their expected AIPs (*Sa* AIP-I and 6-mer AIP, respectively) using both *agr* reporter assays on supernatant samples and LC-MS detection methods. While *Se* sender cell supernatants also were capable of *agr* inhibition in a reporter system, the *Se* AIP-I and/or miscleavage products were produced at insufficient levels for LC-MS detection. Full cell systems containing both AgrB/D and AgrC/A were then created for all three species and found to self-activate upon induction with xylose, indicating that the three *agr* operons were each functional in this heterologous host.

This study represents, to our knowledge, the first time the *S. epidermidis* and *L. monocytogenes* *agr* QS circuits were examined outside of their native hosts, and the first time the signal generation and signal transduction components of the *S. aureus* *agr* circuit were studied in isolation and together systematically in *B. subtilis*. To further extend the scope of this study, we also reconfigured the *Sa* receiver system into a biosensor capable of sensing *S. aureus* AIP-I and producing the antibacterial enzyme lysostaphin upon detection at sufficient levels to kill *S. aureus* on agar plates and in liquid culture. This biosensor was able to detect three groups of *S. aureus* (-I, -III, and -IV) yet was insensitive to *S. aureus* group-II and *S. epidermidis*, highlighting its selectivity and potential utility.

This study yielded several important outcomes. A first outcome is our demonstration that the engineered receiver and full cells are responsive to not only their native signals, but also a range of synthetic QSMs, with activity profiles that generally match those in wild-type hosts. This work demonstrates important proof-of-concept for the application of heterologous *agr* systems for the evaluation of exogenous compounds. The ability to isolate the *agr* system from other regulatory networks in the native host organism—both known and unknown to intersect with *agr*—is advantageous for mechanistic studies. Further, as *B. subtilis* is nonpathogenic and a BSL1 organism, the manipulation of these strains has obvious practical benefits relative to working with the host pathogens with regards to biosafety. We believe this system could be particularly useful for the study of putative *agr* components from other, less-studied Gram-positive bacteria to begin to characterize their QS circuits.

Second, the *B. subtilis* heterologous strains provided insights into the underlying mechanisms of *agr* QS systems and of QSM activity, along with highlighting potential interesting differences between species. For example, the low level of AIP signal produced by *Se* sender cells relative to *Sa* and *Lm* sender cells indicates potential differences in AgrD processing when expressed in *B. subtilis*, which may also reflect variance in AIP biosynthesis between *S. aureus*, *S. epidermidis*, and *L. monocytogenes*. In turn, analysis of the AIP generated in *Lm* sender cells provided additional evidence for the 6-mer being a native AIP signal used by *L. monocytogenes*, a question of recent intrigue.^27^ When testing QSMs in the receiver constructs, unusual activation activity was observed for the inhibitors *Sa* AIP-III and **D4A** in the *Sa* receiver cells, and for the inhibitors *Se* AIP-III and **AAA** in the *Se* receiver cells. This activity in *S. aureus* is congruent with the results of a prior study by the Muir lab that showed unexpected activation for similar peptidic inhibitors in an *in vitro* AgrC-I activity assay.^58^ In our *B. subtilis* system, some inhibitory activity can be recovered for certain inhibitors when tested in competition with the native AIP, as demonstrated in the *Se* receiver and full cells. These discrepancies in activity could be attributed to partial agonism profiles in the *B. subtilis* system that are not observed in the native system. At least in the *Se* systems, compounds may also interact differently with AgrC when native AIP is present, a phenomenon that we have observed before for certain *S. epidermidis* agonists in the native host.^25^ Further experiments are necessary to investigate these differences, including *in vitro* experiments involving the *S. epidermidis* AgrC receptor and investigation of the inverse agonism trends observed with certain inhibitors in the *S. aureus* system.

As a third outcome, we demonstrate that the *B. subtilis* receiver system can be readily redesigned to provide a *S. aureus* biosensor strain that is responsive to multiple native AIPs and generates the bacteriolytic agent lysostaphin at sufficient levels for killing of *S. aureus*. Although these results represent meaningful advances over previous *agr* biosensor work, our characterization of the system is still at an early stage. Additional experimentation to reduce leaky expression and determine sufficient levels of AIP needed for activation should further our understanding of the utility of this sensor system. Nevertheless, we note that a range of genes could be inserted after the P3 promoter in this receiver system, allowing for the AIP-dependent production of a wide variety of functional gene products depending on desired outcome. In addition, this biosensing system could be readily generalized to sense other *agr*-active bacteria beyond *S. aureus*. Overall, this suite of *B. subtilis* systems represent valuable biological tools that complement the growing arsenal of chemical modulators of QS and may offer new pathways for the study of cell–cell communication networks in Gram-positive bacteria.

## Supplementary Material



## Notes

Additional experimental methods; full details of primers, genetic components, and vector sequences; and additional *B. subtilis* strain characterization data (PDF)
