# Supplementary material for: Engineered Bacillus subtilis Systems for the Characterization and Detection of Peptide-Based Quorum Sensing in Gram-Positive Bacteria
Source: Biochemistry. 2026 Apr 15;65(9):1561–77. doi: 10.1021/acs.biochem.6c00065 (PMC13151043; doi:10.1021/acs.biochem.6c00065)
Supplement: Supplementary file 1 [file bi6c00065_si_001.pdf]

SUPPORTING INFORMATION

Engineered *Bacillus subtilis* Systems for the Characterization and Detection of Peptide-based Quorum Sensing in Gram-Positive Bacteria

Emma L. Eisenbraun,[1] Natalie L. Beebe,[1] Alexandra E. Nelson,[1] Brendan N. Prosser,[1] Troy D. Vulpis,[1] Amanda E. Appel,[2] and Helen E. Blackwell[1,*]

[1]*Department of Chemistry, University of Wisconsin–Madison, 1101 University Ave., Madison, WI 53706, USA;* [2]*Microbiology Doctoral Training Program, University of Wisconsin–Madison, 1550 Linden Dr., Madison, WI 53706, USA; Email:* blackwell@chem.wisc.edu

CONTENTS

- Plasmid design and construction methods
- **Figure S1.** Scheme of iterative plasmid design
- **Table S1.** Primers used in this study
- **Table S2.** Genetic components used in this study
- Plasmid sequences
- Characterization data for *S. aureus* AIP-III + N-terminal alanine standard
- **Figure S2.** Growth curves for *S. aureus* and *S. epidermidis* sender, receiver, and full cells
- **Figure S3.** Fluorescence data for GFP-only control *B. subtilis* cells
- **Figure S4.** Standard curves for AIP quantification in sender cell supernatants
- **Figure S5.** Extracted ion chromatograms (EICs) from MS^1 analyses of *S. aureus* and *L. monocytogenes* sender cell supernatants
- **Figure S6.** Fluorescence data for AgrA- and AgrC-only control *B. subtilis* cells
- **Figure S7.** Representative dose-response data for certain QS modulators in *S. aureus* and *S. epidermidis* receiver and full cells
- Method for AgrC labeling experiments
- **Figure S8.** Examination of relative AgrC-I expression levels via fluorescent labeling
- **Table S3.** QS modulator activity data in *S. aureus* and *S. epidermidis agr* reporter strains
- **Figure S9.** QS inhibitor activity in *S. aureus* full cells induced with 0.1% xylose
- **Figure S10.** NCBI BLAST protein alignment of *S. aureus* and *S. epidermidis* AgrB and AgrD sequences used in *B. subtilis* sender and full cells
- **Figure S11.** *S. aureus* receiver cell LysT zone of inhibition assay data for supernatant controls
- **Figure S12.** *S. aureus* receiver cell LysT versus *S. aureus* agr groups I-IV zone of inhibition quantification
- **Figure S13.** *S. aureus* receiver cell LysT versus *S. epidermidis agr* groups I–III zone of inhibition assay data

- MIC measurement method for lysostaphin in *S. aureus* LAC
- **Figure S14.** Growth inhibition of *S. aureus* LAC upon treatment with lysostaphin and MIC determination
- Method to estimate lysostaphin concentration produced by *S. aureus* receiver cell LysT construct
- **Figure S15.** Effect of xylose percentage on zone of inhibition assay analysis
- References

Plasmid design and construction methods.

The plasmids in this study were made in an iterative fashion as shown schematically in **Figure S1**. All primers used in this study are listed in **Table S1**, and all genetic components are listed in **Table S2**. First, the *S. aureus* and *S. epidermidis agr* regions (AgrBD, AgrCA, and AgrBDCA) were cloned into the *Bacillus* BioBrick vector pBS0ExylRP$_{xylA(V2)}$ (**Figure S1A**) using Gibson Assembly and Phusion PCR methods (primers #1-24 in **Table S1**).[1, 2] The *agr* regions were amplified from *S. aureus* RN3690 and *S. epidermidis* RP62A genomic DNA.

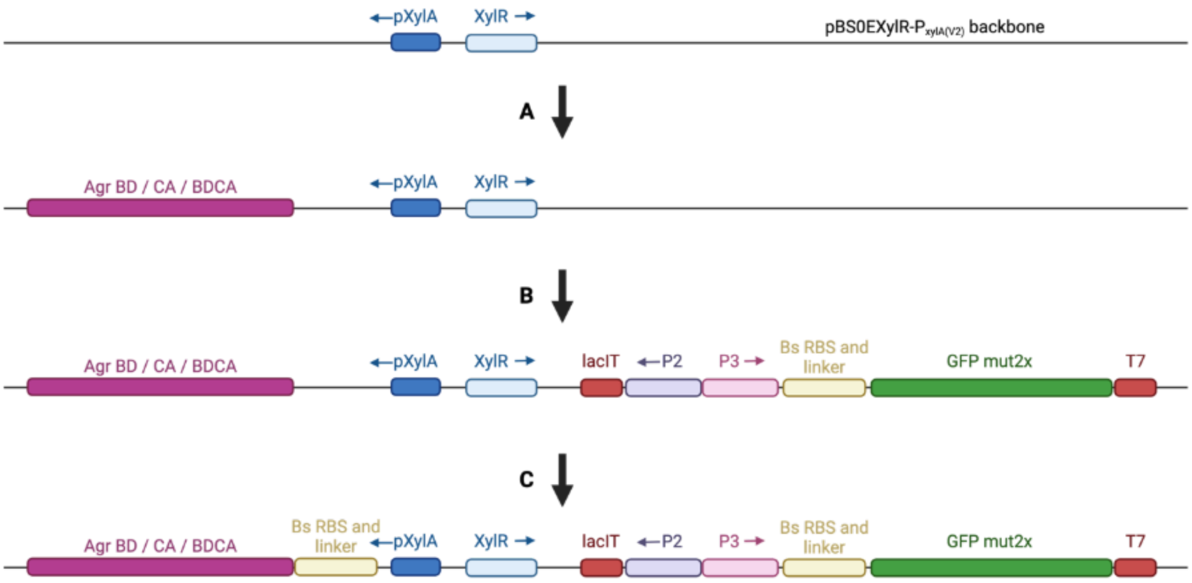

Figure S1. Scheme of iterative plasmid design. (A) *agrBD*, *agrCA*, or *agrBDCA* were added under control of a xylose-inducible promoter in the pBS0EXylR-P$_{xylA(V2)}$ vector. (B) *gfp* was added under P3 control to allow for *agr*-regulated fluorescence readout. Terminators were also added after the P2 promoter and GFP gene. C) A *B. subtilis*-optimized RBS was added in front of the *agr* genes, resulting in the final vectors.

Subsequently, for the plasmids requiring a GFP readout system (i.e., the *S. aureus* and *S. epidermidis* AgrCA and AgrBDCA-containing constructs), the P3 promoter was added in front of *Bacillus*-optimized GFP (**Figure S1B**) using Gibson Assembly and Phusion PCR methods (primers #25-36 in **Table S1**). The full divergent *S. aureus* and *S. epidermidis* P2 and P3 promoters were selected to better replicate native DNA binding conditions and were amplified from *S. aureus* RN3690 and *S. epidermidis* RP62A genomic DNA. The lacIT terminator was added using primers after the P2 promoter to prevent any P2 transcription. A gBlock containing the sequence of a *B. subtilis* RBS, spacer, *Bacillus*-optimized GFP, and the T7 terminator taken from the pNM58 plasmid published by Gonzalez *et al.* were ordered from Integrated DNA Technologies (IDT).[3] The plasmids were assembled using a three-component Gibson Assembly (the *agr*-containing vector backbone, the P3 promoter, and the GFP gene).

The final step in assembling the *S. aureus* and *S. epidermidis* plasmids was to add a *Bacillus* RBS and spacer in front of the *agr* proteins (AgrBD, AgrCA, or AgrBDCA) using Q5 site-directed mutagenesis methods (**Figure S1C**). The RBS and spacer sequence were added using primers (primers #37-44 in **Table S1**). These cloning methods failed for the *S. epidermidis* AgrBD and

AgrBDCA constructs; thus, to assemble those constructs, the *S. epidermidis* AgrBD and AgrBDCA genes were amplified using Phusion PCR methods from previously constructed plasmids and added into the plasmids that successfully had the RBS and spacer added, replacing the *agr* proteins already present, using Gibson Assembly techniques (primers #45-52 in **Table S1**). This process resulted in the final *Bacillus* sender cell plasmids (pXyl-Sa-agrBD and pXyl-Se-agrBD), receiver cell plasmids (pXyl-Sa-agrCA-GFP and pXyl-Se-agrCA-GFP), and full cell plasmids (pXyl-Sa-agrBDCA-GFP and pXyl-Se-agrBDCA-GFP).

Lastly, *Bacillus* plasmids containing the *L. monocytogenes agr* machinery and P3 promoter were created using Gibson Assembly and Phusion PCR methods (primers #55-74 in **Table S1**). The *L. monocytogenes* P3 promoter, *agrBD*, *agrCA*, and *agrBDCA* were amplified from *L. monocytogenes* EGD-e genomic DNA and added into the *S. aureus* sender, receiver, and full cell vector backbones (pXyl-Sa-agrBD, pXyl-Sa-agrCA-GFP, pXyl-Sa-agrBDCA-GFP), respectively, replacing the *S. aureus agr* machinery and promoters. The plasmids were assembled using a four-component Gibson Assembly for the receiver and full cells (two vector backbone portions, the *L. monocytogenes* AgrCA and AgrBDCA genes, and the P3 promoter) and a two-component Gibson Assembly for the sender cells (the vector backbone and the *L. monocytogenes* AgrBD gene). This process resulted in the final *Bacillus* sender, receiver, and full cell plasmids pXyl-Lm-agrBD, pXyl-Lm-agrCA-GFP, and pXyl-Lm-agrBDCA-GFP.

Control plasmids containing only the P3-GFP readout system and lacking the *agr* machinery were created for all three constructs using Q5 site-directed mutagenesis (primers #53, 54, 75 and 76 in **Table S1**). Specifically, the AgrBDCA gene was removed from the full cell plasmids (pXyl-Sa-agrBDCA-GFP, pXyl-Se-agrBDCA-GFP and pXyl-Lm-agrBDCA-GFP) to generate the control plasmids pSa-control-GFP, pSe-control-GFP and pLm-control-GFP.

Single gene control plasmids containing the P3-GFP readout system and either *agrA* or *agrC* under control of the xylose inducible promoter were created for all three constructs using Gibson Assembly and Phusion PCR methods (primers #77-100 in **Table S1**). The backbone for each of these plasmids was amplified from the corresponding full cell constructs (pXyl-Sa-agrBDCA-GFP, pXyl-Se-agrBDCA-GFP, and pXyl-Lm-agrBDCA-GFP) without the AgrBDCA gene. The individual AgrA or AgrC gene was also amplified from the full cell constructs and inserted into the backbone via Gibson Assembly to generate AgrA and AgrC only control *Bacillus* plasmids (pXyl-Sa-agrA-GFP, pXyl-Sa-agrC-GFP, pXyl-Se-agrA-GFP, pXyl-Se-agrC-GFP, pXyl-Lm-agrA-GFP and pXyl-Lm-agrC-GFP).

Table S1. Primers used in this study. All primers were created for this study and ordered from Integrated DNA Technologies. The portions of the primers used to add the lacIT terminator in front of the P3 promoter are underlined, and the portions of the primers used to add the RBS and spacer in front of the *agr* genes are bolded.

Number	Name	Sequence (5' – 3')
1	Sa-BD-I-f	CCATGATTACGCCAAGCTTGCATGCCTGCATTATTCGTGTAATTGTGTTAATTCTTTTGG
2	Sa-BD-I-r	TTACTATATGAAATAAAATGCATCTGTAGAATGAATTATTTTGATAATAAAATTGACCAG
3	Sa-BD-V-f	CTGGTCAATTTTATTATCAAAATAATTCATTCTACAGATGCATTTTATTTCATATAGTAAGTACATCAC
4	Sa-BD-V-r	CCAAAAGAATTAACACAATTACACGAATAATGCAGGCATGCAAGCTTGGCGTAATCATGG
5	Se-BD-I-f	CCATGATTACGCCAAGCTTGCATGCCTGCATTACTCGTATAGTTTAGTCAGTTCTTCTGG
6	Se-BD-I-r	TTACTATATGAAATAAAATGCATCTGTAGAATGAAAATCATCGATAAAAAAATTGAGCAA
7	Se-BD-V-f	TTGCTCAATTTTTTTATCGATGATTTTCATTCTACAGATGCATTTTATTTCATATAGTAAGTACATCAC
8	Se-BD-V-r	CCAGAAGAACTGACTAAACTATACGAGTAATGCAGGCATGCAAGCTTGGCGTAATCATGG
9	Sa-CA-I-f	CCATGATTACGCCAAGCTTGCATGCCTGCATTATATTTTTTTAACGTTTCTCACCGATGC
10	Sa-CA-I-r	TTACTATATGAAATAAAATGCATCTGTAGAATGGAATTATTAAATAGTTATAATTTTGTT
11	Sa-CA-V-f	AACAAAATTATAACTATTTAATAATTCCATTCTACAGATGCATTTTATTTCATATAGTAAGTACATCAC
12	Sa-CA-V-r	GCATCGGTGAGAAACGTTAAAAAAATATAATGCAGGCATGCAAGCTTGGCGTAATCATGG
13	Se-CA-I-f	CCATGATTACGCCAAGCTTGCATGCCTGCATTATATTTTTTTAACATTACGTACTGAAGC
14	Se-CA-I-r	TTACTATATGAAATAAAATGCATCTGTAGAATGGATGATATTAATCTATTTCCGTTTGCA
15	Se-CA-V-f	TGCAAACGGAAATAGATTAATATCATCCATTCTACAGATGCATTTTATTTCATATAGTAAGTACATCAC
16	Se-CA-V-r	GCTTCAGTACGTAATGTTAAAAAAATATAATGCAGGCATGCAAGCTTGGCGTAATCATGG
17	Sa-BDCA-I-f	CCATGATTACGCCAAGCTTGCATGCCTGCATTATATTTTTTTAACGTTTCTCACCGATGC
18	Sa-BDCA-I-r	TTACTATATGAAATAAAATGCATCTGTAGAATGAATTATTTTGATAATAAAATTGACCAG
19	Sa-BDCA-V-f	CTGGTCAATTTTATTATCAAAATAATTCATTCTACAGATGCATTTTATTTCATATAGTAAGTACATCAC
20	Sa-BDCA-V-r	GCATCGGTGAGAAACGTTAAAAAAATATAATGCAGGCATGCAAGCTTGGCGTAATCATGG
21	Se-BDCA-I-f	CCATGATTACGCCAAGCTTGCATGCCTGCATTATATTTTTTTAACATTACGTACTGAAGC
22	Se-BDCA-I-r	TTACTATATGAAATAAAATGCATCTGTAGAATGAAAATCATCGATAAAAAAATTGAGCAA
23	Se-BDCA-V-f	TTGCTCAATTTTTTTATCGATGATTTTCATTCTACAGATGCATTTTATTTCATATAGTAAGTACATCAC
24	Se-BDCA-V-r	GCTTCAGTACGTAATGTTAAAAAAATATAATGCAGGCATGCAAGCTTGGCGTAATCATGG
25	Sa-BackboneV-f	TGACAGTGAAGCCAGGCATTAACCGGGCAGGCCATGTCTGCCCGTATTTCGGTAAAACGACGGCCAGTGAAT
26	Sa-BackboneV-r	GCCTCTAAACGGGTCTTGAGGGGTTTTTTGAACGTCGTGACTGGGAAAACCCT
27	Sa-GFP-f	TAACGCCAGGGTTTTCCCAGTCACGACGTTCAAAAAACCCCTCAAGACCCGTTTAGAGGC
28	Sa-GFP-r	AAATGCTTTTAGCATGTTTTAATATAACTAGATCACAGAGATGTGGTCAGTCAGTCTTAA
29	Sa-P3-f	TTAAGACTGACTGACCACATCTCTGTGATCTAGTTATATTAAAACATGCTAAAAGCATTTATTT
30	Sa-P3-r	TCGTTTTACCGAAATACGGGCAGACATGGCCTGCCCGGTTAATGCCTGGCTTCACTGTCATTATACGATTTAG
31	Se-BackboneV-f	GACACTTGAAGCCAGGCATTAACCGGGCAGGCCATGTCTGCCCGTATTTCGGTAAAACGACGGCCAGTGAAT
32	Se-BackboneV-r	GCCTCTAAACGGGTCTTGAGGGGTTTTTTGAACGTCGTGACTGGGAAAACCCT

33	Se-GFP-f	TAACGCCAGGGTTTTCCCAGTCACGACGTTCAAAAAACCCCTCAAGACCCGTTTAGAGGC
34	Se-GFP-r	AAATGGAAAAATCATGTTTTAATAGACTCAGATCACAGAGATGTGGTCAGTCAGTCTTAA
35	Se-P3-f	TTAAGACTGACTGACCACATCTCTGTGATCTGAGTCTATTAAAACATGATTTTTCCATTTAA AGATTAAAAT
36	Se-P3-r	TCGTTTTACCGAAATACGGGCAGACATGGCCTGCCCGGTTAATGCCTGGCTTCAAGTGTCAT TATACAATTTTGC
37	Sa-BD-RBS-f	**GAACTACT**ATGAATTATTTTGATAATAAAATTGACC
38	Sa-BD-RBS-r	**CTCCTTA**TCTACAGATGCATTTTATTTCATATAG
39	Sa-CA-RBS-f	**GAACTACT**ATGGAATTATTAAATAGTTATAATTTTGTTTTATTC
40	Sa-CA-RBS-r	**CTCCTTA**TCTACAGATGCATTTTATTTCATATAG
41	Sa-BDCA-RBS-f	**CCTCCTTA**TCTACAGATGCATTTTATTTCATATAG
42	Sa-BDCA-RBS-r	**AACTACT**ATGAATTATTTTGATAATAAAATTGACC
43	Se-CA-RBS-f	**CCTCCTTA**TCTACAGATGCATTTTATTTCATATAG
44	Se-CA-RBS-r	**AACTACT**ATGGATGATATTAATCTATTTCCG
45	Se-BD-RBSV-f	TTGCTCAATTTTTTTATCGATGATTTTCATAGTAGTTCCTCCTTATCTACAGATGCATTT
46	Se-BD-RBSV-r	CCAGAAGAACTGACTAAACTATACGAGTAATGCAGGCATGCAAGCTTGGCGTAATCATGG
47	Se-BD-RBSI-f	CCATGATTACGCCAAGCTTGCATGCCTGCATTACTCGTATAGTTTAGTCAGTTCTTCTGG
48	Se-BD-RBSI-r	AAATGCATCTGTAGATAAGGAGGAACTACTATGAAAATCATCGATAAAAAAATTGAGCAA
49	Se-BDCA-RBSV-f	TTGCTCAATTTTTTTATCGATGATTTTCATAGTAGTTCCTCCTTATCTACAGATGCATTT
50	Se-BDCA-RBSV-r	GCTTCAGTACGTAATGTTAAAAAAATATAATGCAGGCATGCAAGCTTGGCGTAATCATGG
51	Se-BDCA-RBSI-f	CCATGATTACGCCAAGCTTGCATGCCTGCATTATATTTTTTTAACATTACGTACTGAAGC
52	Se-BDCA-RBSI-r	AAATGCATCTGTAGATAAGGAGGAACTACTATGAAAATCATCGATAAAAAAATTGAGCAA
53	Control-GFP-f	TGCAGGCATGCAAGCTTG
54	Control-GFP-r	TCTACAGATGCATTTTATTTCATATAGTAAGTACATC
55	Lm-BD-V-f	CAAAGGGACTTTTGCAGTAAAATTACTCAAAGTAGTTCCTCCTTATCTACAGATGCATTT
56	Lm-BD-V-r	AAAATGCAAGAAAAAAACGAAAATAAATAATGCAGGCATGCAAGCTTGGCGTAATCATGG
57	Lm-BD-Agr-f	CCATGATTACGCCAAGCTTGCATGCCTGCATTATTTATTTTCGTTTTTTTCTTGCATTTT
58	Lm-BD-Agr-r	AAATGCATCTGTAGATAAGGAGGAACTACTTTGAGTAATTTTACTGCAAAAGTCCCTTTG
59	Lm-CA-V1-f	TGGGTAAATTCGTTGTAAAATATTAGTGGAGGTGAATTAGGATCACAGAGATGTGGTCAGTC AGTCTTAATTAACCAAGG
60	Lm-CA-V1-r	TTATGCATCATCGAAATTAATTAAAAGCTTGAGTTTATAATGCAGGCATGCAAGCTTGGCGT AATCATGGCCATAGCTGT
61	Lm-CA-Agr-f	ACAGCTATGGCCATGATTACGCCAAGCTTGCATGCCTGCATTATAAACTCAAGCTTTTAATT AATTTCGATGATGCATAA
62	Lm-CA-Agr-r	ATATGAAATAAAATGCATCTGTAGATAAGGAGGAACTACTATGTTTAGTATTTTGATGGCAA TTATACAGATAACGGGTA
63	Lm-CA-V2-f	TACCCGTTATCTGTATAATTGCCATCAAAATACTAAACATAGTAGTTCCTCCTTATCTACAG ATGCATTTTATTTCATAT
64	Lm-CA-V2-r	TGGCTTTTCCATTTTATTAATCGTCTTGTTCTGTTTGAAGAGCCAGGCATTAACCGGGCAGG CCATGTCTGCCCGTATTT
65	Lm-CA-P3-f	AAATACGGGCAGACATGGCCTGCCCGGTTAATGCCTGGCTCTTCAAACAGAACAAGACGATT AATAAAATGGAAAAGCCA
66	Lm-CA-P3-r	CCTTGGTTAATTAAGACTGACTGACCACATCTCTGTGATCCTAATTCACCTCCACTAATATT TTACAACGAATTTACCCA

67	Lm-BDCA-V1-f	TGGGTAAATTCGTTGTAAAATATTAGTGGAGGTGAATTAGGATCACAGAGATGTGGTCAGTCAGTCTTAATTAACCAAGG
68	Lm-BDCA-V1-r	TTATGCATCATCGAAATTAATTAAAAGCTTGAGTTTATAATGCAGGCATGCAAGCTTGGCGTAATCATGGCCATAGCTGT
69	Lm-BDCA-Agr-f	ACAGCTATGGCCATGATTACGCCAAGCTTGCATGCCTGCATTATAAACTCAAGCTTTTAATTAATTTCGATGATGCATAA
70	Lm-BDCA-Agr-r	ATATGAAATAAAATGCATCTGTAGATAAGGAGGAACTACTTTGAGTAATTTTACTGCAAAAGTCCCTTTGTCAGAAAGAA
71	Lm-BDCA-V2-f	TTCTTTCTGACAAAGGGACTTTTGCAGTAAAATTACTCAAAGTAGTTCCTCCTTATCTACAGATGCATTTTATTTCATAT
72	Lm-BDCA-V2-r	TGGCTTTTCCATTTTATTAATCGTCTTGTTCTGTTTGAAGAGCCAGGCATTAACCGGGCAGGCCATGTCTGCCCGTATTT
73	Lm-BDCA-P3-f	AAATACGGGCAGACATGGCCTGCCCGGTTAATGCCTGGCTCTTCAAACAGAACAAGACGATTAATAAAATGGAAAAGCCA
74	Lm-BDCA-P3-r	CCTTGGTTAATTAAGACTGACTGACCACATCTCTGTGATCCTAATTCACCTCCACTAATATTTTACAACGAATTTACCCA
75	Lm-Control-GFP-f	ATGCAGGCATGCAAG
76	Lm-Control-GFP-r	ATCTACAGATGCATTTTATTTCATATAG
77	Sa-AgrA-V-f	TCGCAAATGAAAATTTTCATAGTAGTTCCTCCTTATCTACAGATGCATTTT
78	Sa-AgrA-V-r	GAAACGTTAAAAAAATATAATGCAGGCATGCAAGCTTGGC
79	Sa-AgrA-I-f	GCCAAGCTTGCATGCCTGCATTATATTTTTTTAACGTTTCTCACCG
80	Sa-AgrA-I-r	GTAGATAAGGAGGAACTACTATGAAAATTTTCATTTGCGAAGA
81	Sa-AgrC-V-f	TAACTATTTAATAATTCCACAGTAGTTCCTCCTTATCTACAGATGCATTTT
82	Sa-AgrC-V-r	TTGAAATTATTAACAACTAGTGCAGGCATGCAAGCTTGGC
83	Sa-AgrC-I-f	GCCAAGCTTGCATGCCTGCACTAGTTGTTAATAATTTCAACTTTTTGAA
84	Se-AgrC-I-r	TGTAGATAAGGAGGAACTACTGTGGAATTATTAAATAGTTATAATTTTGTTTTAT
85	Se-AgrA-V-f	TCACAAACAAAAATTTTCATAGTAGTTCCTCCTTATCTACAGATGCATTTT
86	Se-AgrA-V-r	GTAATGTTAAAAAAATATAATGCAGGCATGCAAGCTTGGC
87	Se-AgrA-I-f	GCCAAGCTTGCATGCCTGCATTATATTTTTTTAACATTACGTACTGAAGC
88	Se-AgrA-I-r	GTAGATAAGGAGGAACTACTATGAAAATTTTTGTTTGTGAAGATG
89	Se-AgrC-V-f	AATAGATTAATATCATCCATAGTAGTTCCTCCTTATCTACAGATGCATTTT
90	Se-AgrC-V-r	TAAATAATAAGGAATCATAATGCAGGCATGCAAGCTTGGC
91	Se-AgrC-I-f	GCCAAGCTTGCATGCCTGCATTATGATTCCTTATTATTTATTTCTACTTTTTG
92	Se-AgrC-I-r	GTAGATAAGGAGGAACTACTATGGATGATATTAATCTATTTCCGTT
93	Lm-AgrA-V-f	CAAATAAAAACCGGTAGCATAGTAGTTCCTCCTTATCTACAGATGCATTTT
94	Lm-AgrA-V-r	TTAAAAGCTTGAGTTTATAATGCAGGCATGCAAGCTTGGC
95	Lm-AgrA-I-f	GCCAAGCTTGCATGCCTGCATTATAAACTCAAGCTTTTAATTAATTTCG
96	Lm-AgrA-I-r	GTAGATAAGGAGGAACTACTATGCTACCGGTTTTTATTTGT
97	Lm-AgrC-V-f	GCCATCAAAATACTAAACATAGTAGTTCCTCCTTATCTACAGATGCATTTT
98	Lm-AgrC-V-r	AAGAATTAGAAATTATGTAGTGCAGGCATGCAAGCTTGGC
99	Lm-AgrC-I-f	GCCAAGCTTGCATGCCTGCACTACATAATTTCTAATTCTTGAATAACTTC
100	Lm-AgrC-I-r	GTAGATAAGGAGGAACTACTATGTTTAGTATTTTGATGGCAATTAT
101	Sa-LysT-V-f	ATAACGGCAGTAAAGAGGTTTTGAATCGTTTTGCAAACATTGTACATTTCACCTCCTTGGTTAATTAAGACTGACTGACC
102	Sa-LysT-V-r	TACTAATACTTTAGGTGTTCTTTGGGGAACTATAAAGTAATAACCTAGGCTGCTGCCACCGCTGAGCAATAACTAGCATA

103	Sa-LysT-I-f	TATGCTAGTTATTGCTCAGCGGTGGCAGCAGCCTAGGTTATTACTTATAGTTCCCCAAAGAA CACCTAAAGTATTAGTA
104	Sa-LysT-I-r	GGTCAGTCAGTCTTAATTAACCAAGGAGGTGAAATGTACAATGTTTCGAAAACGATTCAAAA CCTCTTTACTGCCGTTAT

Table S2. Genetic components used in this study.

Name	Type	DNA Sequence	Ref.
S. aureus P3	Promoter	TCACTGTCATTATACGATTTAGTACAATCTTTTATCATTATATT<br>GCCTAACTGTAGGAAATAAATACTTAACTGTTAAATGTAATTTG<br>TATTTAATATTTTAACATAAAAAAATTTACAGTTAAGAATAAAA<br>AACGACTAGTTAAGAAAAATTGGAAAATAAATGCTTTTAGCATG<br>TTTTAATATAACTA	This work
S. epidermidis P3	Promoter	CCAAGTGTCATTATACAATTTTGCGCAACATTTTTTAGAAAGCA<br>TGCCTAACTGTTAAAAAAATATACCTAAGTGTTTTAATTAAGTA<br>CTATTAGATATTTTACCATATTTAGTTTTACAGTTGAGTACTAA<br>ATATTGCTATTTACGAAATTTTAATCTTTAAATGGAAAAATCAT<br>GTTTTAATAGACTCA	This work
L. monocytogenes P3	Promoter	CTTCAAACAGAACAAGACGATTAATAAAATGGAAAAGCCAACTG<br>TGAACATTCTTTTGCAGTTGGTTTTTTTTATGAATAAACTCACT<br>TAAATAAAATATTAATATGGGTTTTTAAGCCTATTATACAGTCC<br>TTTTTACACGTTCGAATATTCTGAAATGTATCCAAAAAATCGGC<br>AATTCTGCTCTTTTTTGGTCGTTCATGACAAGAATCGGACATTT<br>CATTACATTTTTGGTTATTATGGGTAAATTCGTTGTAAAATATT<br>AGTGGAGGTGAATTAG	This work
bsc	RBS	AGGAGG	Popp _et al._, 2017[1]; González et al., 2020[3]
gfpmut2	CDS	ATGAAAGGAGAAGAACTTTTCACTGGAGTTGTCCCAATTCTTGT<br>TGAATTAGATGGTGATGTTAATGGGCACAAATTTTCTGTCAGTG<br>GAGAGGGTGAAGGTGATGCAACATACGGAAAACTTACCCTTAAA<br>TTTATTTGCACTACTGGAAAACTACCTGTTCCATGGCCAACACT<br>TGTCACTACTTTCGCGTATGGTCTTCAATGCTTTGCGAGATACC<br>CAGATCATATGAAACAGCATGACTTTTTCAAGAGTGCCATGCCC<br>GAAGGTTATGTACAGGAAAGAACTATATTTTTCAAAGATGACGG<br>GAACTACAAGACACGTGCTGAAGTCAAGTTTGAAGGTGATACCC<br>TTGTTAATAGAATCGAGTTAAAAGGTATTGATTTTAAAGAAGAT<br>GGAAACATTCTTGGACACAAATTGGAATACAACTATAACTCACA<br>CAATGTATACATCATGGCAGACAAACAAAAGAATGGAATCAAAG<br>TTAACTTCAAAATTAGACACAACATTGAAGATGGAAGCGTTCAA<br>CTAGCAGACCATTATCAACAAAATACTCCAATTGGCGATGGCCC<br>TGTCCTTTTACCAGACAACCATTACCTGTCCACACAATCTAAGC<br>TTTCGAAAGATCCCAACGAAAAGAGAGACCACATGGTCCTTCTT<br>GAGTTTGTAACAGCTGCTGGGATTACACATGGCATGGATGAACT<br>ATACAAA	González _et al._, 2020[3]
S. aureus agrBD	CDS	ATGAATTATTTTGATAATAAAATTGACCAGTTTGCCACGTATCT<br>TCAAAAGAGAAATAACTTAGATCATATTCAATTTTTGCAAGTAC<br>GATTAGGGATGCAGGTCTTAGCTAAAAATATAGGTAAATTAATT<br>GTTATGTATACTATTGCCTATATTTTAAACATTTTTCTGTTTAC<br>GTTAATTACGAATTTAACATTTTATTTAATAAGAAGACATGCAC<br>ATGGTGCACATGCACCTTCTTCTTTTTGGTGTTATGTAGAAAGT<br>ATTATACTATTTATACTTTTACCTTTAGTAATAGTAAATTTTCA<br>TATTAACTTTTTAATTATGATTATTTTAACAGTTATTTCTTTAG<br>GTGTAATCTCAGTATATGCTCCTGCAGCAACTAAAAAGAAGCCC<br>ATTCCTGTGCGACTTATTAAACGAAAAAAATATTATGCGATTAT<br>TGTTAGTTTAACCCTTTTCATTATCACACTTATCATCAAAGAGC<br>CATTTGCCCAATTCATTCAATTAGGCATCATAATAGAAGCTATT<br>ACATTATTACCTATTTTCTTTATTAAGGAGGACTTAAAATGAAT<br>ACATTATTTAACTTATTTTTTGATTTTATTACTGGGATTTTAAA<br>AAACATTGGTAACATCGCAGCTTATAGTACTTGTGACTTCATAA<br>TGGATGAAGTTGAAGTACCAAAAGAATTAACACAATTACACGAA<br>TAA	This work
S. aureus agrCA	CDS	ATGGAATTATTAAATAGTTATAATTTTGTTTTATTCGTATTAAC<br>TCAAATGATATTAATGTTTACAATACCAGCTATAATTAGTGGTA<br>TTAAGTACAGTAAACTTGATTATTTTTTCATCATAGTAATTTCG	This work

		ACATTATCGTTATTTCTATTTAAAATGTTTGATAGCGCGTCCTT<br>AATCATATTAACTTCATTTATTATTATAATGTATTTTGTCAAAA<br>TCAAATGGTATTCTATTTTGTTGATTATGACTTCGCAGATTATT<br>CTATACTGTGCTAACTACATGTATATAGTTATATATGCATATAT<br>CACCAAAATTTCTGATAGTATATTTGTAATATTCCCTAGCTTTT<br>TTGTAGTTTATGTGACTATTAGTATACTATTCTCATATATAATA<br>AATAGAGTTCTCAAAAAAATTAGCACACCATATCTAATACTAAA<br>CAAAGGATTTTTAATAGTTATTTCGACTATCTTACTGCTTACTT<br>TTTCATTATTTTTCTTTTATTCACAAATAAACTCGGATGAAGCT<br>AAAGTAATAAGGCAGTATTCTTTTATTTTTATTGGTATCACTAT<br>ATTTTTAAGTATATTAACATTTGTTATTTCTCAATTTCTCCTTA<br>AAGAGATGAAATATAAACGTAATCAAGAAGAAATTGAAACCTAT<br>TATGAATATACATTGAAGATTGAAGCTATCAACAACGAAATGCG<br>CAAGTTCCGTCATGATTATGTCAATATCTTAACGACACTTTCAG<br>AATACATTCGAGAAGATGACATGCCTGGCCTACGTGATTATTTC<br>AATAAAAATATTGTACCTATGAAAGACAATTTACAAATGAATGC<br>TATAAAATTAAATGGTATCGAGAATCTTAAAGTACGTGAAATTA<br>AAGGCTTAATTACTGCGAAAATTTTACGTGCACAAGAAATGAAT<br>ATTCCGATTAGTATCGAAATACCCGATGAAGTAAGTAGCATTAA<br>CTTGAATATGATCGATTTAAGTCGCAGTATTGGTATTATTCTTG<br>ATAATGCAATTGAGGCATCAACTGAAATTGATGACCCTATCATT<br>CGCGTTGCATTTATTGAAAGTGAAAATTCAGTAACGTTTATTGT<br>TATGAATAAATGCGCTGATGATATACCACGCATTCATGAATTGT<br>TCCAAGAAAGTTTTTCTACTAAAGGTGAAGGTCGTGGTTTAGGT<br>CTATCAACTTTAAAAGAAATTGCTGATAATGCAGACAATGTCTT<br>ATTAGATACAATTATCGAAAATGGTTTCTTTATTCAAAAAGTTG<br>AAATTATTAACAACTAGCCATAAGGATGTGAATGTATGAAAATT<br>TTCATTTGCGAAGACGATCCAAAACAAAGAGAAAACATGGTTAC<br>CATTATTAAAAATTATATAATGATAGAAGAAAAGCCTATGGAAA<br>TTGCCCTCGCAACTGATAATCCTTATGAGGTGCTTGAGCAAGCT<br>AAAAATATGAATGACATAGGCTGTTACTTTTTAGATATTCAACT<br>TTCAACTGATATTAATGGTATCAAATTAGGCAGTGAAATTCGTA<br>AGCATGACCCAGTTGGTAACATTATTTTCGTTACGAGTCACAGT<br>GAACTTACCTATTTAACATTTGTCTACAAAGTTGCAGCGATGGA<br>TTTTATTTTTAAAGATGATCCAGCTGAATTAAGAACTCGAATTA<br>TAGACTGTTTAGAAACTGCACATACACGCTTACAATTGTTGTCT<br>AAAGATAATAGCGTTGAAACGATTGAATTAAAACGTGGCAGTAA<br>TTCAGTGTATGTTCAATATGATGATATTATGTTTTTTGAATCAT<br>CAACAAAATCTCACAGACTCATTGCCCATTTAGATAACCGTCAA<br>ATTGAATTTTATGGTAATTTAAAAGAACTGAGTCAATTAGATGA<br>TCGTTTCTTTAGATGTCATAATAGCTTTGTCGTCAATCGCCATA<br>ATATTGAATCTATAGATTCGAAAGAGCGAATTGTCTATTTTAAA<br>AATAAAGAACACTGCTATGCATCGGTGAGAAACGTTAAAAAAAT<br>ATAA	
S. aureus agrC	CDS	CTAGTTGTTAATAATTTCAACTTTTTGAATAAAGAAACCATTTT<br>CGATAATTGTATCTAATAAGACATTGTCTGCATTATCAGCAATT<br>TCTTTTAAAGTTGATAGACCTAAACCACGACCTTCACCTTTAGT<br>AGAAAAACTTTCTTGGAACAATTCATGAATGCGTGGTATATCAT<br>CAGCGCATTTATTCATAACAATAAACGTTACTGAATTTTCACTT<br>TCAATAAATGCAACGCGAATGATAGGGTCATCAATTTCAGTTGA<br>TGCCTCAATTGCATTATCAAGAATAATACCAATACTGCGACTTA<br>AATCGATCATATTCAAGTTAATGCTACTTACTTCATCGGGTATT<br>TCGATACTAATCGGAATATTCATTTCTTGTGCACGTAAAATTTT<br>CGCAGTAATTAAGCCTTTAATTTCACGTACTTTAAGATTCTCGA<br>TACCATTTAATTTTATAGCATTCATTTGTAAATTGTCTTTCATA<br>GGTACAATATTTTTATTGAAATAATCACGTAGGCCAGGCATGTC<br>ATCTTCTCGAATGTATTCTGAAAGTGTCGTTAAGATATTGACAT<br>AATCATGACGGAACTTGCGCATTTCGTTGTTGATAGCTTCAATC<br>TTCAATGTATATTCATAATAGGTTTCAATTTCTTCTTGATTACG<br>TTTATATTTCATCTCTTTAAGGAGAAATTGAGAAATAACAAATG<br>TTAATATACTTAAAAATATAGTGATACCAATAAAAATAAAAGAA<br>TACTGCCTTATTACTTTAGCTTCATCCGAGTTTATTTGTGAATA<br>AAAGAAAAATAATGAAAAAGTAAGCAGTAAGATAGTCGAAATAA	This work

		CTATTAAAAATCCTTTGTTTAGTATTAGATATGGTGTGCTAATT<br>TTTTTGAGAACTCTATTTATTATATATGAGAATAGTATACTAAT<br>AGTCACATAAACTACAAAAAAGCTAGGGAATATTACAAATATAC<br>TATCAGAAATTTTGGTGATATATGCATATATAACTATATACATG<br>TAGTTAGCACAGTATAGAATAATCTGCGAAGTCATAATCAACAA<br>AATAGAATACCATTTGATTTTGACAAAATACATTATAATAATAA<br>ATGAAGTTAATATGATTAAGGACGCGCTATCAAACATTTTAAAT<br>AGAAATAACGATAATGTCGAAATTACTATGATGAAAAAATAATC<br>AAGTTTACTGTACTTAATACCACTAATTATAGCTGGTATTGTAA<br>ACATTAATATCATTTGAGTTAATACGAATAAAACAAAATTATAA<br>CTATTTAATAATTCCAC	
S. aureus agrA	CDS	TTATATTTTTTTAACGTTTCTCACCGATGCATAGCAGTGTTCTT<br>TATTTTTAAAATAGACAATTCGCTCTTTCGAATCTATAGATTCA<br>ATATTATGGCGATTGACGACAAAGCTATTATGACATCTAAAGAA<br>ACGATCATCTAATTGACTCAGTTCTTTTAAATTACCATAAAATT<br>CAATTTGACGGTTATCTAAATGGGCAATGAGTCTGTGAGATTTT<br>GTTGATGATTCAAAAAACATAATATCATCATATTGAACATACAC<br>TGAATTACTGCCACGTTTTAATTCAATCGTTTCAACGCTATTAT<br>CTTTAGACAACAATTGTAAGCGTGTATGTGCAGTTTCTAAACAG<br>TCTATAATTCGAGTTCTTAATTCAGCTGGATCATCTTTAAAAAT<br>AAAATCCATCGCTGCAACTTTGTAGACAAATGTTAAATAGGTAA<br>GTTCACTGTGACTCGTAACGAAAATAATGTTACCAACTGGGTCA<br>TGCTTACGAATTTCACTGCCTAATTTGATACCATTAATATCAGT<br>TGAAAGTTGAATATCTAAAAAGTAACAGCCTATGTCATTCATAT<br>TTTTAGCTTGCTCAAGCACCTCATAAGGATTATCAGTTGCGAGG<br>GCAATTTCCATAGGCTTTTCTTCTATCATTATATAATTTTTAAT<br>AATGGTAACCATGTTTTCTCTTTGTTTTGGATCGTCTTCGCAAA<br>TGAAAATTTTCAT	This work
S. aureus agrBDCA	CDS	ATGAATTATTTTGATAATAAAATTGACCAGTTTGCCACGTATCT<br>TCAAAAGAGAAATAACTTAGATCATATTCAATTTTTGCAAGTAC<br>GATTAGGGATGCAGGTCTTAGCTAAAAATATAGGTAAATTAATT<br>GTTATGTATACTATTGCCTATATTTTAAACATTTTTCTGTTTAC<br>GTTAATTACGAATTTAACATTTTATTTAATAAGAAGACATGCAC<br>ATGGTGCACATGCACCTTCTTCTTTTTGGTGTTATGTAGAAAGT<br>ATTATACTATTTATACTTTTACCTTTAGTAATAGTAAATTTTCA<br>TATTAACTTTTTAATTATGATTATTTTAACAGTTATTTCTTTAG<br>GTGTAATCTCAGTATATGCTCCTGCAGCAACTAAAAAGAAGCCC<br>ATTCCTGTGCGACTTATTAAACGAAAAAAATATTATGCGATTAT<br>TGTTAGTTTAACCCTTTTCATTATCACACTTATCATCAAAGAGC<br>CATTTGCCCAATTCATTCAATTAGGCATCATAATAGAAGCTATT<br>ACATTATTACCTATTTTCTTTATTAAGGAGGACTTAAAATGAAT<br>ACATTATTTAACTTATTTTTTGATTTTATTACTGGGATTTTAAA<br>AAACATTGGTAACATCGCAGCTTATAGTACTTGTGACTTCATAA<br>TGGATGAAGTTGAAGTACCAAAAGAATTAACACAATTACACGAA<br>TAATTTAAATAGAGAGTGTGATAGTAGGTGGAATTATTAAATAG<br>TTATAATTTTGTTTTATTCGTATTAACTCAAATGATATTAATGT<br>TTACAATACCAGCTATAATTAGTGGTATTAAGTACAGTAAACTT<br>GATTATTTTTTCATCATAGTAATTTCGACATTATCGTTATTTCT<br>ATTTAAAATGTTTGATAGCGCGTCCTTAATCATATTAACTTCAT<br>TTATTATTATAATGTATTTTGTCAAAATCAAATGGTATTCTATT<br>TTGTTGATTATGACTTCGCAGATTATTCTATACTGTGCTAACTA<br>CATGTATATAGTTATATATGCATATATCACCAAAATTTCTGATA<br>GTATATTTGTAATATTCCCTAGCTTTTTTGTAGTTTATGTGACT<br>ATTAGTATACTATTCTCATATATAATAAATAGAGTTCTCAAAAA<br>AATTAGCACACCATATCTAATACTAAACAAAGGATTTTTAATAG<br>TTATTTCGACTATCTTACTGCTTACTTTTTCATTATTTTTCTTT<br>TATTCACAAATAAACTCGGATGAAGCTAAAGTAATAAGGCAGTA<br>TTCTTTTATTTTTATTGGTATCACTATATTTTTAAGTATATTAA<br>CATTTGTTATTTCTCAATTTCTCCTTAAAGAGATGAAATATAAA<br>CGTAATCAAGAAGAAATTGAAACCTATTATGAATATACATTGAA<br>GATTGAAGCTATCAACAACGAAATGCGCAAGTTCCGTCATGATT	This work

		ATGTCAATATCTTAACGACACTTTCAGAATACATTCGAGAAGAT<br>GACATGCCTGGCCTACGTGATTATTTCAATAAAAATATTGTACC<br>TATGAAAGACAATTTACAAATGAATGCTATAAAATTAAATGGTA<br>TCGAGAATCTTAAAGTACGTGAAATTAAAGGCTTAATTACTGCG<br>AAAATTTTACGTGCACAAGAAATGAATATTCCGATTAGTATCGA<br>AATACCCGATGAAGTAAGTAGCATTAACTTGAATATGATCGATT<br>TAAGTCGCAGTATTGGTATTATTCTTGATAATGCAATTGAGGCA<br>TCAACTGAAATTGATGACCCTATCATTCGCGTTGCATTTATTGA<br>AAGTGAAAATTCAGTAACGTTTATTGTTATGAATAAATGCGCTG<br>ATGATATACCACGCATTCATGAATTGTTCCAAGAAAGTTTTTCT<br>ACTAAAGGTGAAGGTCGTGGTTTAGGTCTATCAACTTTAAAAGA<br>AATTGCTGATAATGCAGACAATGTCTTATTAGATACAATTATCG<br>AAAATGGTTTCTTTATTCAAAAAGTTGAAATTATTAACAACTAG<br>CCATAAGGATGTGAATGTATGAAAATTTTCATTTGCGAAGACGA<br>TCCAAAACAAAGAGAAAACATGGTTACCATTATTAAAAATTATA<br>TAATGATAGAAGAAAAGCCTATGGAAATTGCCCTCGCAACTGAT<br>AATCCTTATGAGGTGCTTGAGCAAGCTAAAAATATGAATGACAT<br>AGGCTGTTACTTTTTAGATATTCAACTTTCAACTGATATTAATG<br>GTATCAAATTAGGCAGTGAAATTCGTAAGCATGACCCAGTTGGT<br>AACATTATTTTCGTTACGAGTCACAGTGAACTTACCTATTTAAC<br>ATTTGTCTACAAAGTTGCAGCGATGGATTTTATTTTTAAAGATG<br>ATCCAGCTGAATTAAGAACTCGAATTATAGACTGTTTAGAAACT<br>GCACATACACGCTTACAATTGTTGTCTAAAGATAATAGCGTTGA<br>AACGATTGAATTAAAACGTGGCAGTAATTCAGTGTATGTTCAAT<br>ATGATGATATTATGTTTTTTGAATCATCAACAAAATCTCACAGA<br>CTCATTGCCCATTTAGATAACCGTCAAATTGAATTTTATGGTAA<br>TTTAAAAGAACTGAGTCAATTAGATGATCGTTTCTTTAGATGTC<br>ATAATAGCTTTGTCGTCAATCGCCATAATATTGAATCTATAGAT<br>TCGAAAGAGCGAATTGTCTATTTTAAAAATAAAGAACACTGCTA<br>TGCATCGGTGAGAAACGTTAAAAAAATATAA	
S. epidermidis agrBD	CDS	ATGAAAATCATCGATAAAAAAATTGAGCAATTTGCTCAATATTT<br>ACAACGTAAAAATAACTTAGATCACATACAGTTTCTAAAAATTC<br>GTTTAGGGATGCAGGTACTAGCGATAAATATTGAAAAGTCTATA<br>GTTGTGTATGGGCTAGCAATAATCTTTCATACTTTCTTTTACAC<br>ACTTTTAACTCATTTAAGTTATTTTTTAATTAGGAGACATGCAC<br>ACGGTACGCATGCAAATTCGTCATTGTTATGTCATATTCAGAAC<br>ATAATTTTCTTTATTATCTTTCCATACTTAATAATAAAGTTAGA<br>TATTAACTATTTTGTTCTTTTATCTATGGCATTAGTCGGATTAA<br>TTATTACCATTTTATACGCACCTGCAGCAACTAAGAAACAACCT<br>ATACCTAGACGTCTTGTAAAGCGAAAAAAAATACTCTCCATATT<br>TTTATATTGTACTATCGTAGTTATTTCATTAGTAACTAAAGAAC<br>CGGTAAATAAACTTATTTTATTCGGTGTAATTTTAGAATCTTTA<br>ACATTACTACCCATCTTTTTCCCTAAGGAGGATATTAATCATGG<br>AAAACATTTTTAATTTATTTATAAAATTTTTCACTACAATCTTG<br>GAATTTATTGGTACTGTAGCAGGAGATAGTGTATGTGCCTCTTA<br>CTTTGACGAACCAGAAGTACCAGAAGAACTGACTAAACTATACG<br>AGTAA	This work
S. epidermidis agrCA	CDS	ATGGATGATATTAATCTATTTCCGTTTGCAGGCCTACAAATCTT<br>TTTAATGATTTGGGTTACTAAAGTTATCATTAATATGAAATTTA<br>ATTTTAGGGATTACATAATCGTTTTTACGATTGTAATCCCTTCT<br>GCAATAATGTATTACTTTTGGCAAAGTAAAGCATTAATAGTTTT<br>GGTTATAATAATCACCATTTTCTTTTATACAAAAATAAAGCTTT<br>ATTCAATATTAGTTGTATTATTCACCACTATGATCTTATATATA<br>ACTAATTTCATAACTGTATACATACATTTGACTATAAAAGATTA<br>TATTCCGTTTAAATTTGCTTTACAGTTAATACATTTTACCTCTT<br>TTGTAATCATAACTCTAATTATTGCTTATTTAACTCAACTATTG<br>TTCAATAAATTAAAAGTATCCTACTTGTCACTCAATAAAAGATA<br>CTTATTGATAATAACAATAGTACTTTTCATATCATTTATTTTAC<br>TTTATATGGTGTCACAAACTGATATGCGAGGAAATGATACCCTT<br>AAATTATATGCCATCTTATTGTTGGGTATTATGGTTTTTTTAAG<br>TGTAGTGATATTAGTGATGTCCAATTTTACACTCCGTGAAATGA<br>GGTATAAACGTAATGTAAAAGAAATCGAAGCATATTATGAGTAC<br>ACGTTACGTATAGAAAGCATTAACAATGAAATGCGTAAGTTCCG	This work

		ACATGATTATGTGAATATCCTCACCACTCTTTCAGATTACATTA<br>GAGAAGATGATATGCCTGGATTACGTAAATATTTTAATGAAAAT<br>ATCGTTCCAATGAAAGATAAATTAAAAACTCGCTCTATTAAAAT<br>GAATGGTATTGAAAAGTTGAAAGTGAGAGAAATTAAAGGGCTGA<br>TTACTACTAAAATTATTCAAGCTCAAGAAAAACGTATTCCAATT<br>AGTATTGAGGTTCCTGATGAAATTGATCGTATCGATATGAATAC<br>TGTTGAGCTAAGTCGTATTATCGGTATTATAGTTGATAATGCTA<br>TTGAAGCTTCAGAAAATCTTGAGGAACCACTCATCAATATCGCA<br>TTCATCGATAATGAGGAATCTGTCACTTTTATCGTTATGAATAA<br>ATGTAGTAATGATATCCCTAAAATTCATGAGTTGTTTGAACAAG<br>GTTTTTCTACTAAAGGTGATAATCGCGGTTTAGGTTTATCAACT<br>TTAAAAGAACTGACAGACTCAAACGAGAATGTTTTATTAGATAC<br>TGTCATCGAAAATGGTTACTTTGTACAAAAAGTAGAAATAAATA<br>ATAAGGAATCATAAGGATGTGTAGAATTAAATGAAAATTTTTGT<br>TTGTGAAGATGACCAAAGACAAAGAGAACATATGGTATCAATCA<br>TTAAAAACTACATAATGATTGAAGAAAAGCCAATGGAGTTAGCT<br>TTAGCAACAAATGATCCTTATGAGGTCTTAGAGCAATCAAAAGA<br>ACTTAATGACATTGGTTGTTACTTCCTTGATATTCAATTAGAAG<br>CTGATATGAACGGTATTAAATTAGCCAGTGAAATTCGTAAACAT<br>GATCCTGTTGGTAATATTATATTTGTAACCAGTCACAGTGAGCT<br>GACTTATTTGACGTTTGTTTATAAAGTGGCTGCTATGGATTTTA<br>TTTTTAAAGATGATCCATCTGAATTAAAAATGAGAATCATAGAT<br>TGTCTTGAAACAGCACATACACGACTCAAATTATTATCAAAAGA<br>AAGTAATGTAGATACGATTGAGTTAAAGCGGGGAAGTAATTCAG<br>TATACGTTCAATATGATGATATTATGTTTTTTGAATCATCTACG<br>AAATCTCATAGACTCATTGCACATCTTGATAATCGACAAATTGA<br>ATTTTATGGAAATTTAAAGGAATTAGCACAGCTTGATGAACGTT<br>TCTTTAGATGTCATAACAGTTTTGTAATAAACAGGCATAATATT<br>GAATCTATTGACTCAAAAGAACGTATTGTTTACTTTAAGAATGG<br>CGAAAATTGTTTCGCTTCAGTACGTAATGTTAAAAAAATATAA	
S. epidermidis agrC	CDS	TTATGATTCCTTATTATTTATTTCTACTTTTTGTACAAAGTAAC<br>CATTTTCGATGACAGTATCTAATAAAACATTCTCGTTTGAGTCT<br>GTCAGTTCTTTTAAAGTTGATAAACCTAAACCGCGATTATCACC<br>TTTAGTAGAAAAACCTTGTTCAAACAACTCATGAATTTTAGGGA<br>TATCATTACTACATTTATTCATAACGATAAAAGTGACAGATTCC<br>TCATTATCGATGAATGCGATATTGATGAGTGGTTCCTCAAGATT<br>TTCTGAAGCTTCAATAGCATTATCAACTATAATACCGATAATAC<br>GACTTAGCTCAACAGTATTCATATCGATACGATCAATTTCATCA<br>GGAACCTCAATACTAATTGGAATACGTTTTTCTTGAGCTTGAAT<br>AATTTTAGTAGTAATCAGCCCTTTAATTTCTCTCACTTTCAACT<br>TTTCAATACCATTCATTTTAATAGAGCGAGTTTTTAATTTATCT<br>TTCATTGGAACGATATTTTCATTAAAATATTTACGTAATCCAGG<br>CATATCATCTTCTCTAATGTAATCTGAAAGAGTGGTGAGGATAT<br>TCACATAATCATGTCGGAACTTACGCATTTCATTGTTAATGCTT<br>TCTATACGTAACGTGTACTCATAATATGCTTCGATTTCTTTTAC<br>ATTACGTTTATACCTCATTTCACGGAGTGTAAAATTGGACATCA<br>CTAATATCACTACACTTAAAAAAACCATAATACCCAACAATAAG<br>ATGGCATATAATTTAAGGGTATCATTTCCTCGCATATCAGTTTG<br>TGACACCATATAAAGTAAAATAAATGATATGAAAAGTACTATTG<br>TTATTATCAATAAGTATCTTTTATTGAGTGACAAGTAGGATACT<br>TTTAATTTATTGAACAATAGTTGAGTTAAATAAGCAATAATTAG<br>AGTTATGATTACAAAAGAGGTAAAATGTATTAACTGTAAAGCAA<br>ATTTAAACGGAATATAATCTTTTATAGTCAAATGTATGTATACA<br>GTTATGAAATTAGTTATATATAAGATCATAGTGGTGAATAATAC<br>AACTAATATTGAATAAAGCTTTATTTTTGTATAAAAGAAAATGG<br>TGATTATTATAACCAAAACTATTAATGCTTTACTTTGCCAAAAG<br>TAATACATTATTGCAGAAGGGATTACAATCGTAAAAACGATTAT<br>GTAATCCCTAAAATTAAATTTCATATTAATGATAACTTTAGTAA<br>CCCAAATCATTAAAAAGATTTGTAGGCCTGCAAACGGAAATAGA<br>TTAATATCATCCAT	This work

S. epidermidis agrA	CDS	TTATATTTTTTTAACATTACGTACTGAAGCGAAACAATTTTCGC<br>CATTCTTAAAGTAAACAATACGTTCTTTTGAGTCAATAGATTCA<br>ATATTATGCCTGTTTATTACAAAACTGTTATGACATCTAAAGAA<br>ACGTTCATCAAGCTGTGCTAATTCCTTTAAATTTCCATAAAATT<br>CAATTTGTCGATTATCAAGATGTGCAATGAGTCTATGAGATTTC<br>GTAGATGATTCAAAAAACATAATATCATCATATTGAACGTATAC<br>TGAATTACTTCCCCGCTTTAACTCAATCGTATCTACATTACTTT<br>CTTTTGATAATAATTTGAGTCGTGTATGTGCTGTTTCAAGACAA<br>TCTATGATTCTCATTTTTAATTCAGATGGATCATCTTTAAAAAT<br>AAAATCCATAGCAGCCACTTTATAAACAAACGTCAAATAAGTCA<br>GCTCACTGTGACTGGTTACAAATATAATATTACCAACAGGATCA<br>TGTTTACGAATTTCACTGGCTAATTTAATACCGTTCATATCAGC<br>TTCTAATTGAATATCAAGGAAGTAACAACCAATGTCATTAAGTT<br>CTTTTGATTGCTCTAAGACCTCATAAGGATCATTTGTTGCTAAA<br>GCTAACTCCATTGGCTTTTCTTCAATCATTATGTAGTTTTTAAT<br>GATTGATACCATATGTTCTCTTTGTCTTTGGTCATCTTCACAAA<br>CAAAAATTTTCAT	This work
S. epidermidis agrBDCA	CDS	ATGAAAATCATCGATAAAAAAATTGAGCAATTTGCTCAATATTT<br>ACAACGTAAAAATAACTTAGATCACATACAGTTTCTAAAAATTC<br>GTTTAGGGATGCAGGTACTAGCGATAAATATTGAAAAGTCTATA<br>GTTGTGTATGGGCTAGCAATAATCTTTCATACTTTCTTTTACAC<br>ACTTTTAACTCATTTAAGTTATTTTTTAATTAGGAGACATGCAC<br>ACGGTACGCATGCAAATTCGTCATTGTTATGTCATATTCAGAAC<br>ATAATTTTCTTTATTATCTTTCCATACTTAATAATAAAGTTAGA<br>TATTAACTATTTTGTTCTTTTATCTATGGCATTAGTCGGATTAA<br>TTATTACCATTTTATACGCACCTGCAGCAACTAAGAAACAACCT<br>ATACCTAGACGTCTTGTAAAGCGAAAAAAAATACTCTCCATATT<br>TTTATATTGTACTATCGTAGTTATTTCATTAGTAACTAAAGAAC<br>CGGTAAATAAACTTATTTTATTCGGTGTAATTTTAGAATCTTTA<br>ACATTACTACCCATCTTTTTCCCTAAGGAGGATATTAATCATGG<br>AAAACATTTTTAATTTATTTATAAAATTTTTCACTACAATCTTG<br>GAATTTATTGGTACTGTAGCAGGAGATAGTGTATGTGCCTCTTA<br>CTTTGACGAACCAGAAGTACCAGAAGAACTGACTAAACTATACG<br>AGTAAATATAACCCTAGAAAGTGTGTAAGATATGGATGATATTA<br>ATCTATTTCCGTTTGCAGGCCTACAAATCTTTTTAATGATTTGG<br>GTTACTAAAGTTATCATTAATATGAAATTTAATTTTAGGGATTA<br>CATAATCGTTTTTACGATTGTAATCCCTTCTGCAATAATGTATT<br>ACTTTTGGCAAAGTAAAGCATTAATAGTTTTGGTTATAATAATC<br>ACCATTTTCTTTTATACAAAAATAAAGCTTTATTCAATATTAGT<br>TGTATTATTCACCACTATGATCTTATATATAACTAATTTCATAA<br>CTGTATACATACATTTGACTATAAAAGATTATATTCCGTTTAAA<br>TTTGCTTTACAGTTAATACATTTTACCTCTTTTGTAATCATAAC<br>TCTAATTATTGCTTATTTAACTCAACTATTGTTCAATAAATTAA<br>AAGTATCCTACTTGTCACTCAATAAAAGATACTTATTGATAATA<br>ACAATAGTACTTTTCATATCATTTATTTTACTTTATATGGTGTC<br>ACAAACTGATATGCGAGGAAATGATACCCTTAAATTATATGCCA<br>TCTTATTGTTGGGTATTATGGTTTTTTTAAGTGTAGTGATATTA<br>GTGATGTCCAATTTTACACTCCGTGAAATGAGGTATAAACGTAA<br>TGTAAAAGAAATCGAAGCATATTATGAGTACACGTTACGTATAG<br>AAAGCATTAACAATGAAATGCGTAAGTTCCGACATGATTATGTG<br>AATATCCTCACCACTCTTTCAGATTACATTAGAGAAGATGATAT<br>GCCTGGATTACGTAAATATTTTAATGAAAATATCGTTCCAATGA<br>AAGATAAATTAAAAACTCGCTCTATTAAAATGAATGGTATTGAA<br>AAGTTGAAAGTGAGAGAAATTAAAGGGCTGATTACTACTAAAAT<br>TATTCAAGCTCAAGAAAAACGTATTCCAATTAGTATTGAGGTTC<br>CTGATGAAATTGATCGTATCGATATGAATACTGTTGAGCTAAGT<br>CGTATTATCGGTATTATAGTTGATAATGCTATTGAAGCTTCAGA<br>AAATCTTGAGGAACCACTCATCAATATCGCATTCATCGATAATG<br>AGGAATCTGTCACTTTTATCGTTATGAATAAATGTAGTAATGAT<br>ATCCCTAAAATTCATGAGTTGTTTGAACAAGGTTTTTCTACTAA<br>AGGTGATAATCGCGGTTTAGGTTTATCAACTTTAAAAGAACTGA<br>CAGACTCAAACGAGAATGTTTTATTAGATACTGTCATCGAAAAT	This work

		GGTTACTTTGTACAAAAAGTAGAAATAAATAATAAGGAATCATA<br>AGGATGTGTAGAATTAAATGAAAATTTTTGTTTGTGAAGATGAC<br>CAAAGACAAAGAGAACATATGGTATCAATCATTAAAAACTACAT<br>AATGATTGAAGAAAAGCCAATGGAGTTAGCTTTAGCAACAAATG<br>ATCCTTATGAGGTCTTAGAGCAATCAAAAGAACTTAATGACATT<br>GGTTGTTACTTCCTTGATATTCAATTAGAAGCTGATATGAACGG<br>TATTAAATTAGCCAGTGAAATTCGTAAACATGATCCTGTTGGTA<br>ATATTATATTTGTAACCAGTCACAGTGAGCTGACTTATTTGACG<br>TTTGTTTATAAAGTGGCTGCTATGGATTTTATTTTTAAAGATGA<br>TCCATCTGAATTAAAAATGAGAATCATAGATTGTCTTGAAACAG<br>CACATACACGACTCAAATTATTATCAAAAGAAAGTAATGTAGAT<br>ACGATTGAGTTAAAGCGGGGAAGTAATTCAGTATACGTTCAATA<br>TGATGATATTATGTTTTTTGAATCATCTACGAAATCTCATAGAC<br>TCATTGCACATCTTGATAATCGACAAATTGAATTTTATGGAAAT<br>TTAAAGGAATTAGCACAGCTTGATGAACGTTTCTTTAGATGTCA<br>TAACAGTTTTGTAATAAACAGGCATAATATTGAATCTATTGACT<br>CAAAAGAACGTATTGTTTACTTTAAGAATGGCGAAAATTGTTTC<br>GCTTCAGTACGTAATGTTAAAAAAATATAA	
L. monocytogenes agrBD	CDS	TTATTTATTTTCGTTTTTTTCTTGCATTTTCACAAATGGACTTT<br>TTGGTTCGTATACAAACATGAAGCAAGCTTTACTCATAGAAGAA<br>TCCGCAACTTTCATGGATTGTTCTTCTAGTTTTCTAGAAAGGAA<br>TTTACCAACTGATTTATTCATATTTTTCATAGTTCCGATACCTC<br>CTTTTCAATAGTTTGTAAGTAAGTGGATTAATACTTATCACCTG<br>AAACAAAGATCCTACCATGATTAATGTTTTCATCTCTGCGAATG<br>GTATTAGCAACGCAATTCCCGTTAAAATTAGCGTCCCTATCATC<br>GCTTTTCTTTTTAGTGTTTTCCGGTGTTCTTCACCGATTAAAGG<br>CAAACTTTCTGTGTCTGCCGGAGCGAATAAAAACATGTTGAGCA<br>GTATAAATCCAAATGTACCTAAAACAATCCAATTATTAGAGGGG<br>ATATTTTGAAAAACAAATGGCGCAAGCACAAACATCAATAAGCT<br>AATTAACGTGCAATTCAATGTTTTGGTTGCATGTAATCCAAAAG<br>AATACCGTCTGAGCCATAAATATGACAGATGCACTGTCACTGTT<br>TGCAGTAAAAGCCCTGTTACTAAGGCGATACCGTATACGAGAGC<br>AAACTTCATGACATTAATTAGAATTATTTCCAGTCCATATTTTA<br>CTTTTAAATAACCTTCTTCATCATCTTTCCAGCGGTCTTTCGAA<br>ATCAACACATCCGCCATTCTTTCTGACAAAGGGACTTTTGCAGT<br>AAAATTACTCAA	This work
L. monocytogenes agrCA	CDS	ATGTTTAGTATTTTGATGGCAATTATACAGATAACGGGTATTTT<br>TATTGCAATCCAGATTTTAACAAACAAAGTTTTTTCAATTAAAG<br>AGGGATTGGTTACTATAGCAATTGCTATGCTAGCCTTCCCTTTA<br>TTTACTTTAGTTCAATACTGGTCGATGATTTTTGTATTGATTGT<br>TTTTGTAAGTGCCTTATATTGGAAAAATAAAAATGTAGTAGTTT<br>CAGCTTCTATTACGCTTGTGGTTATTATTTTACTTACTATTAGT<br>GACTCTATAGTAGGTTTTATTTTAGTACCGGGCTTGAACTTTAA<br>ATATGATGAGATATTTAATGAACTGTTACCAACGTTAATTTACT<br>GCGCGGGAATGTTGGCGAATTTGTTGGTATTTTCGTTTCTTCTT<br>AGAAAATTGATTGAAAAAGTTAATATTTCTAGATTTGTTGAACA<br>TAGAAAATATGCGTATATTATTTTTTCTATCGTTGCTCTTACTG<br>TCTTAGCGTTCTATATGAACATATACGCGGGGTCAATCGCAGGT<br>TTTGATGGATCAGTTTTGAAAATTAATACGCTTATTTTTACAGG<br>ATATACCATTTTGTTAATAGTTATAGTGACGGTTGTTATTAATA<br>CGGCAACCAACGAACTTAAAGTACAGAATCAGAAGGAACAGCTA<br>GAACAGTTACAGGATTATGTTACTACATTAGAGTCCCTGCATAG<br>AGAAATGCGCGTTTTTCGTCATGATTATGTGAATATCCTGTCAA<br>CACTTGTTGGATATATTGATAATAATGATATGCCGGGTTTGAAG<br>TATTACTTTGAAAATAATATTGTACCTATAAATAAAACAATTGA<br>ATCGAACAACTATAAGATTTCATTGCTTCAAAATATTCATGTTA<br>TTGAACTAAAGGGCTTGCTAGCTGTTAAATTAATACGAGCTCAA<br>GAGTTGAAAATTGATGCAATTTTGGAAGTAGTTGAACCGATTGA<br>CAAGATTTCGATGGATAGTATAGATTTATGCAAAGTTGTCGGTA<br>TTTTGCTAGATAATGCGGTTGAAGCGGCGTTAACTTGTGAAAAT<br>CCAGTTATTCGAATCGCGTTTGTGAAAAAAGGTGATAGCATAAT<br>TATTGTATTTGCGAATAGTTTACCAGTAAATATGCCACCGATTT<br>ATAAGATATTTGAAGAAGGTTTCTCTACAAAAGGAGAAGGTCGT	This work

		GGATTAGGCCTTGCTAGTTTGCGGGAAATTATGAAGAAATATTC<br>GCACGTTGCCTTAGATACGAAAGTGACCGATAGAGAAGTTATTC<br>AAGAATTAGAAATTATGTAGAAGAATGGGGATGAATTTATGCTA<br>CCGGTTTTTATTTGTGAAGATAACAGAATGCAGCGAGAAAGGTT<br>AACGAAATATATTGAAGACTATATTATGGTTGAACATTTTGATA<br>TGAAGTTAGAACTTTCAACAGGAGATCCGTTTGAGTTAGTATCA<br>CGAATGCCTACACATCAAGGTATGGGGCTTTATTTTTTAGACAT<br>TGATTTGGGGCAGCCGGACATGAATGGTTTTGAATTAGCTCAGG<br>AAATTCGGAAGTTTGATCCGCGTGGTTTCATTATTTTTATTACA<br>ACACATGCGGAGTTAAGTTATATGACTTTCACGTATAAGGTGGA<br>AGCGCTTGATTACATTATTAAAGACGATATTGATTTGCTGCATG<br>ACCGGGTACTTGCCTGTATGAAGCAAGCAGAAGAACGGATTTCC<br>AATGATCAAGATATGCAGAAGTATTTTACGTTTAAAGTGTCGGA<br>TAAGAAGATTATTCATGAACTGTTAGACGATATCTTGTTTTTTG<br>AGACAGCGCCAACTATTCATAAAGTAATTTTACATGGCAAAAAT<br>CGCCAAGTGGAATTTTATGGTAAGTTGAAGAATATCGAGAAAAT<br>GTTGGACGAATCATTTTATCGGTGCCACAGGTCGTATATTGTGA<br>ATAAGAAAAATATTCATGAGCTAGATACGACAAAAGGTGTGGTG<br>AAGATGTCTAACGGTGAAAATTGTTATGCATCATCGAAATTAAT<br>TAAAAGCTTGAGTTTATAA	
L. monocytogenes agrC	CDS	CTACATAATTTCTAATTCTTGAATAACTTCTCTATCGGTCACTT<br>TCGTATCTAAGGCAACGTGCGAATATTTCTTCATAATTTCCCGC<br>AAACTAGCAAGGCCTAATCCACGACCTTCTCCTTTTGTAGAGAA<br>ACCTTCTTCAAATATCTTATAAATCGGTGGCATATTTACTGGTA<br>AACTATTCGCAAATACAATAATTATGCTATCACCTTTTTTCACA<br>AACGCGATTCGAATAACTGGATTTTCACAAGTTAACGCCGCTTC<br>AACCGCATTATCTAGCAAAATACCGACAACTTTGCATAAATCTA<br>TACTATCCATCGAAATCTTGTCAATCGGTTCAACTACTTCCAAA<br>ATTGCATCAATTTTCAACTCTTGAGCTCGTATTAATTTAACAGC<br>TAGCAAGCCCTTTAGTTCAATAACATGAATATTTTGAAGCAATG<br>AAATCTTATAGTTGTTCGATTCAATTGTTTTATTTATAGGTACA<br>ATATTATTTTCAAAGTAATACTTCAAACCCGGCATATCATTATT<br>ATCAATATATCCAACAAGTGTTGACAGGATATTCACATAATCAT<br>GACGAAAAACGCGCATTTCTCTATGCAGGGACTCTAATGTAGTA<br>ACATAATCCTGTAACTGTTCTAGCTGTTCCTTCTGATTCTGTAC<br>TTTAAGTTCGTTGGTTGCCGTATTAATAACAACCGTCACTATAA<br>CTATTAACAAAATGGTATATCCTGTAAAAATAAGCGTATTAATT<br>TTCAAAACTGATCCATCAAAACCTGCGATTGACCCCGCGTATAT<br>GTTCATATAGAACGCTAAGACAGTAAGAGCAACGATAGAAAAAA<br>TAATATACGCATATTTTCTATGTTCAACAAATCTAGAAATATTA<br>ACTTTTTCAATCAATTTTCTAAGAAGAAACGAAAATACCAACAA<br>ATTCGCCAACATTCCCGCGCAGTAAATTAACGTTGGTAACAGTT<br>CATTAAATATCTCATCATATTTAAAGTTCAAGCCCGGTACTAAA<br>ATAAAACCTACTATAGAGTCACTAATAGTAAGTAAAATAATAAC<br>CACAAGCGTAATAGAAGCTGAAACTACTACATTTTTATTTTTCC<br>AATATAAGGCACTTACAAAAACAATCAATACAAAAATCATCGAC<br>CAGTATTGAACTAAAGTAAATAAAGGGAAGGCTAGCATAGCAAT<br>TGCTATAGTAACCAATCCCTCTTTAATTGAAAAAACTTTGTTTG<br>TTAAAATCTGGATTGCAATAAAAATACCCGTTATCTGTATAATT<br>GCCATCAAAATACTAAACAT	This work
L. monocytogenes agrA	CDS	TTATAAACTCAAGCTTTTAATTAATTTCGATGATGCATAACAAT<br>TTTCACCGTTAGACATCTTCACCACACCTTTTGTCGTATCTAGC<br>TCATGAATATTTTTCTTATTCACAATATACGACCTGTGGCACCG<br>ATAAAATGATTCGTCCAACATTTTCTCGATATTCTTCAACTTAC<br>CATAAAATTCCACTTGGCGATTTTTGCCATGTAAAATTACTTTA<br>TGAATAGTTGGCGCTGTCTCAAAAAACAAGATATCGTCTAACAG<br>TTCATGAATAATCTTCTTATCCGACACTTTAAACGTAAAATACT<br>TCTGCATATCTTGATCATTGGAAATCCGTTCTTCTGCTTGCTTC<br>ATACAGGCAAGTACCCGGTCATGCAGCAAATCAATATCGTCTTT<br>AATAATGTAATCAAGCGCTTCCACCTTATACGTGAAAGTCATAT<br>AACTTAACTCCGCATGTGTTGTAATAAAAATAATGAAACCACGC<br>GGATCAAACTTCCGAATTTCCTGAGCTAATTCAAAACCATTCAT	This work

		GTCCGGCTGCCCCAAATCAATGTCTAAAAAATAAAGCCCCATAC<br>CTTGATGTGTAGGCATTCGTGATACTAACTCAAACGGATCTCCT<br>GTTGAAAGTTCTAACTTCATATCAAAATGTTCAACCATAATATA<br>GTCTTCAATATATTTCGTTAACCTTTCTCGCTGCATTCTGTTAT<br>CTTCACAAATAAAAACCGGTAGCAT	
L. monocytogenes agrBDCA	CDS	TTGAGTAATTTTACTGCAAAAGTCCCTTTGTCAGAAAGAATGGC<br>GGATGTGTTGATTTCGAAAGACCGCTGGAAAGATGATGAAGAAG<br>GTTATTTAAAAGTAAAATATGGACTGGAAATAATTCTAATTAAT<br>GTCATGAAGTTTGCTCTCGTATACGGTATCGCCTTAGTAACAGG<br>GCTTTTACTGCAAACAGTGACAGTGCATCTGTCATATTTATGGC<br>TCAGACGGTATTCTTTTGGATTACATGCAACCAAAACATTGAAT<br>TGCACGTTAATTAGCTTATTGATGTTTGTGCTTGCGCCATTTGT<br>TTTTCAAAATATCCCCTCTAATAATTGGATTGTTTTAGGTACAT<br>TTGGATTTATACTGCTCAACATGTTTTTATTCGCTCCGGCAGAC<br>ACAGAAAGTTTGCCTTTAATCGGTGAAGAACACCGGAAAACACT<br>AAAAAGAAAAGCGATGATAGGGACGCTAATTTTAACGGGAATTG<br>CGTTGCTAATACCATTCGCAGAGATGAAAACATTAATCATGGTA<br>GGATCTTTGTTTCAGGTGATAAGTATTAATCCACTTACTTACAA<br>ACTATTGAAAAGGAGGTATCGGAACTATGAAAAATATGAATAAA<br>TCAGTTGGTAAATTCCTTTCTAGAAAACTAGAAGAACAATCCAT<br>GAAAGTTGCGGATTCTTCTATGAGTAAAGCTTGCTTCATGTTTG<br>TATACGAACCAAAAAGTCCATTTGTGAAAATGCAAGAAAAAAAC<br>GAAAATAAATAAATTTAAAACTGTATAACATCAAAATTCATGCT<br>AAAATAAAAAGAAGGCAAACTTACATATTTAAACATAGATATTT<br>TAGACAGTGGAGGATTAATATGTTTAGTATTTTGATGGCAATTA<br>TACAGATAACGGGTATTTTTATTGCAATCCAGATTTTAACAAAC<br>AAAGTTTTTTCAATTAAAGAGGGATTGGTTACTATAGCAATTGC<br>TATGCTAGCCTTCCCTTTATTTACTTTAGTTCAATACTGGTCGA<br>TGATTTTTGTATTGATTGTTTTTGTAAGTGCCTTATATTGGAAA<br>AATAAAAATGTAGTAGTTTCAGCTTCTATTACGCTTGTGGTTAT<br>TATTTTACTTACTATTAGTGACTCTATAGTAGGTTTTATTTTAG<br>TACCGGGCTTGAACTTTAAATATGATGAGATATTTAATGAACTG<br>TTACCAACGTTAATTTACTGCGCGGGAATGTTGGCGAATTTGTT<br>GGTATTTTCGTTTCTTCTTAGAAAATTGATTGAAAAAGTTAATA<br>TTTCTAGATTTGTTGAACATAGAAAATATGCGTATATTATTTTT<br>TCTATCGTTGCTCTTACTGTCTTAGCGTTCTATATGAACATATA<br>CGCGGGGTCAATCGCAGGTTTTGATGGATCAGTTTTGAAAATTA<br>ATACGCTTATTTTTACAGGATATACCATTTTGTTAATAGTTATA<br>GTGACGGTTGTTATTAATACGGCAACCAACGAACTTAAAGTACA<br>GAATCAGAAGGAACAGCTAGAACAGTTACAGGATTATGTTACTA<br>CATTAGAGTCCCTGCATAGAGAAATGCGCGTTTTTCGTCATGAT<br>TATGTGAATATCCTGTCAACACTTGTTGGATATATTGATAATAA<br>TGATATGCCGGGTTTGAAGTATTACTTTGAAAATAATATTGTAC<br>CTATAAATAAAACAATTGAATCGAACAACTATAAGATTTCATTG<br>CTTCAAAATATTCATGTTATTGAACTAAAGGGCTTGCTAGCTGT<br>TAAATTAATACGAGCTCAAGAGTTGAAAATTGATGCAATTTTGG<br>AAGTAGTTGAACCGATTGACAAGATTTCGATGGATAGTATAGAT<br>TTATGCAAAGTTGTCGGTATTTTGCTAGATAATGCGGTTGAAGC<br>GGCGTTAACTTGTGAAAATCCAGTTATTCGAATCGCGTTTGTGA<br>AAAAAGGTGATAGCATAATTATTGTATTTGCGAATAGTTTACCA<br>GTAAATATGCCACCGATTTATAAGATATTTGAAGAAGGTTTCTC<br>TACAAAAGGAGAAGGTCGTGGATTAGGCCTTGCTAGTTTGCGGG<br>AAATTATGAAGAAATATTCGCACGTTGCCTTAGATACGAAAGTG<br>ACCGATAGAGAAGTTATTCAAGAATTAGAAATTATGTAGAAGAA<br>TGGGGATGAATTTATGCTACCGGTTTTTATTTGTGAAGATAACA<br>GAATGCAGCGAGAAAGGTTAACGAAATATATTGAAGACTATATT<br>ATGGTTGAACATTTTGATATGAAGTTAGAACTTTCAACAGGAGA<br>TCCGTTTGAGTTAGTATCACGAATGCCTACACATCAAGGTATGG<br>GGCTTTATTTTTTAGACATTGATTTGGGGCAGCCGGACATGAAT<br>GGTTTTGAATTAGCTCAGGAAATTCGGAAGTTTGATCCGCGTGG<br>TTTCATTATTTTTATTACAACACATGCGGAGTTAAGTTATATGA<br>CTTTCACGTATAAGGTGGAAGCGCTTGATTACATTATTAAAGAC	This work

		GATATTGATTTGCTGCATGACCGGGTACTTGCCTGTATGAAGCA<br>AGCAGAAGAACGGATTTCCAATGATCAAGATATGCAGAAGTATT<br>TTACGTTTAAAGTGTCGGATAAGAAGATTATTCATGAACTGTTA<br>GACGATATCTTGTTTTTTGAGACAGCGCCAACTATTCATAAAGT<br>AATTTTACATGGCAAAAATCGCCAAGTGGAATTTTATGGTAAGT<br>TGAAGAATATCGAGAAAATGTTGGACGAATCATTTTATCGGTGC<br>CACAGGTCGTATATTGTGAATAAGAAAAATATTCATGAGCTAGA<br>TACGACAAAAGGTGTGGTGAAGATGTCTAACGGTGAAAATTGTT<br>ATGCATCATCGAAATTAATTAAAAGCTTGAGTTTATAA	
lacIT	Terminator	TAACCGGGCAGGCCATGTCTGCCCGTATTTCG	González *et al.*, 2020[3]
T7	Terminator	CTAGCATAACCCCTTGGGGCCTCTAAACGGGTCTTGAGGGGTTT<br>TTTG	González *et al.*, 2020[3]
B. subtilis amyE	Secretion tag	ATGTTTGCAAAACGATTCAAAACCTCTTTACTGCCGTTATTCGC<br>TGGATTTTTATTGCTGTTTCATTTGGTTCTGGCAGGACCGGCGG<br>CTGCGAGTGCT	González *et al.*, 2020[3]
S. simulans lysT	Bacterial metalloendo-peptidase	ACACATGAACATTCAGCACAATGGTTGAATAATTACAAAAAAGG<br>ATATGGTTACGGTCCTTATCCATTAGGTATAAATGGCGGTATGC<br>ACTACGGAGTTGATTTTTTTATGAATATTGGAACACCAGTAAAA<br>GCTATTTCAAGCGGAAAAATAGTTGAAGCTGGTTGGAGTAATTA<br>CGGAGGAGGTAATCAAATAGGTCTTATTGAAAATGATGGAGTGC<br>ATAGACAATGGTATATGCATCTAAGTAAATATAATGTTAAAGTA<br>GGAGATTATGTCAAAGCTGGTCAAATAATCGGTTGGTCTGGAAG<br>CACTGGTTATTCTACAGCACCACATTTACACTTCCAAAGAATGG<br>TTAATTCATTTTCAAATTCAACTGCCCAAGATCCAATGCCTTTC<br>TTAAAGAGCGCAGGATATGGAAAAGCAGGTGGTACAGTAACTCC<br>AACGCCGAATACAGGTTGGAAAACAAACAAATATGGCACACTAT<br>ATAAATCAGAGTCAGCTAGCTTCACACCTAATACAGATATAATA<br>ACAAGAACGACTGGTCCATTTAGAAGCATGCCGCAGTCAGGAGT<br>CTTAAAAGCAGGTCAAACAATTCATTATGATGAAGTGATGAAAC<br>AAGACGGTCATGTTTGGGTAGGTTATACAGGTAACAGTGGCCAA<br>CGTATTTACTTGCCTGTAAGAACATGGAATAAATCTACTAATAC<br>TTTAGGTGTTCTTTGGGGAACTATAAAGTAA	González *et al.*, 2020[3]

Plasmid sequences.

pXyl-Sa-agrBD

```
5′-
TTGAGATCCTTTTTTTCTGCGCGTAATCTGCTGCTTGCAAACAAAAAAACCACCGCTACCAGCGGTGGTTTGTTTGCCGGATCA
AGAGCTACCAACTCTTTTTCCGAAGGTAACTGGCTTCAGCAGAGCGCAGATACCAAATACTGTTCTTCTAGTGTAGCCGTAGTT
AGGCCACCACTTCAAGAACTCTGTAGCACCGCCTACATACCTCGCTCTGCTAATCCTGTTACCAGTGGCTGCTGCCAGTGGCGA
TAAGTCGTGTCTTACCGGGTTGGACTCAAGACGATAGTTACCGGATAAGGCGCAGCGGTCGGGCTGAACGGGGGGTTCGTGCAC
ACAGCCCAGCTTGGAGCGAACGACCTACACCGAACTGAGATACCTACAGCGTGAGCTATGAGAAAGCGCCACGCTTCCCGAAGG
GAGAAAGGCGGACAGGTATCCGGTAAGCGGCAGGGTCGGAACAGGAGAGCGCACGAGGGAGCTTCCAGGGGGAAACGCCTGGTA
TCTTTATAGTCCTGTCGGGTTTCGCCACCTCTGACTTGAGCGTCGATTTTTGTGATGCTCGTCAGGGGGGCGGAGCCTATGGAA
AAACGCCAGCAACGCGGCCTTTTTACGGTTCCTGGCCTTTTGCTGGCCTTTTGCTCACATGTTCTTTCCTGCGTTATCCCCTGA
TTCTGTGGATAACCGTATTACCGCCTTTGAGTGAGCTGATACCGCTCGCCGCAGCCGAACGACCGAGCGCAGCGAGTCAGTGAG
CGAGGAAGCGGAAGAGCGCCCAATACGCAAACCGCCTCTCCCCGCGCGTTGGCCGATTCATTAATGCAGCTGGCACGACAGGTT
TCCCGACTGGAAAGCGGGCAGTGAGCGCAACGCAATTAATGTGAGTTAGCTCACTCATTAGGCACCCCAGGCTTTACACTTTAT
GCTTCCGGCTCGTATGTTGTGTGGAATTGTGAGCGGATAACAATTTCACACAGGAAACAGCTATGACCATGATTACGCCAAGCT
TGCATGCCTGCATTATTCGTGTAATTGTGTTAATTCTTTTGGTACTTCAACTTCATCCATTATGAAGTCACAAGTACTATAAGC
TGCGATGTTACCAATGTTTTTTAAAATCCCAGTAATAAAATCAAAAAATAAGTTAAATAATGTATTCATTTTAAGTCCTCCTTA
ATAAAGAAAATAGGTAATAATGTAATAGCTTCTATTATGATGCCTAATTGAATGAATTGGGCAAATGGCTCTTTGATGATAAGT
GTGATAATGAAAAGGGTTAAACTAACAATAATCGCATAATATTTTTTTCGTTTAATAAGTCGCACAGGAATGGGCTTCTTTTTA
GTTGCTGCAGGAGCATATACTGAGATTACACCTAAAGAAATAACTGTTAAAATAATCATAATTAAAAAGTTAATATGAAAATTT
ACTATTACTAAAGGTAAAAGTATAAATAGTATAATACTTTCTACATAACACCAAAAAGAAGAAGGTGCATGTGCACCATGTGCA
TGTCTTCTTATTAAATAAAATGTTAAATTCGTAATTAACGTAAACAGAAAAATGTTTAAAATATAGGCAATAGTATACATAACA
ATTAATTTACCTATATTTTTAGCTAAGACCTGCATCCCTAATCGTACTTGCAAAAATTGAATATGATCTAAGTTATTTCTCTTT
TGAAGATACGTGGCAAACTGGTCAATTTTATTATCAAAATAATTCATAGTAGTTCCTCCTTATCTACAGATGCATTTTATTTCA
TATAGTAAGTACATCACCTATTAGTTTGTTGTTTAAACAAACTAACTTATTTTCATCTTATATAACCTCGTCAGTATTTTCAAT
ATTTTTTTTAGTTTTTTATGAACACATTAGATATAATAAAGGGAAGATTCGCTATGTACTATGTTGATACTTAATTTAAAGATT
AAACAAATGGAGTGGATGAAGTGGATATCGCTGATCAAACCTTTGTCAAAAAAGTAAATCAAAAGTTATTATTAAAAGAAATCC
TTAAAAATTCACCTATTTCAAGAGCAAAATTATCTGAAATGACTGGATTAAATAAATCAACTGTCTCATCACAGGTAAACACGT
TAATGAAAGAAAGTATGGTATTTGAAATAGGTCAAGGACAATCAAGTGGCGGAAGAAGACCTGTCATGCTTGTTTTTAATAAAA
AGGCAGGATACTCCGTTGGAATAGATGTTGGTGTGGATTATATTAATGGCATTTTAACAGACCTTGAAGGAACAATCGTTCTTG
ATCAATACCGCCATTTGGAATCCAATTCTCCAGAAATAACGAAAGACATTTTGATTGATATGATTCATCACTTTATTACGCAAA
TGCCCCAATCTCCGTACGGGCTTATTGGTATAGGTATTTGCGTGCCTGGACTCATTGATAAAGATCAAAAAATTGTTTTCACTC
CGAACTCCAACTGGAGAGATATTGACTTAAAATCTTCGATACAAGAGAAGTACAATGTGCCTGTTTTTATTGAAAATGAGGCAA
ATGCTGGCGCATATGGAGAAAAAGTATTTGGAGCTGCAAAAAATCACGATAACATTATTTACGTAAGTATCAGCACAGGAATAG
GGATCGGTGTTATTATCAACAATCATTTATATAGAGGAGTAAGCGGCTTCTCTGGAGAAATGGGACATATGACAATAGACTTTA
ATGGTCCTAAATGCAGTTGCGGAAACCGAGGATGCTGGGAATTGTATGCTTCAGAGAAGGCTTTATTAAAATCTCTTCAGACCA
AAGAGAAAAAACTGTCCTATCAAGATATCATAAACCTCGCCCATCTGAATGATATCGGAACCTTAAATGCATTACAAAATTTTG
GATTCTATTTAGGAATAGGCCTTACCAATATTCTAAATACTTTCAACCCACAAGCCGTAATTTTAAGAAATAGCATAATTGAAT
CGCATCCTATGGTTTTAAATTCAATGAGAAGTGAAGTATCATCAAGGGTTTATTCCCAATTAGGCAATAGCTATGAATTATTGC
CATCTTCCTTAGGACAGAATGCACCGGCATTAGGAATGTCCTCCATTGTGATTGATCATTTTCTGGACATGATTACAATGTAAT
TTTTTATGGAATGGACAGCTCATCTTTAAAGATGAGTTTTTTTATTCTAGGAGTATTTCTGAAGCAATAGTGACATGGCACCTT
CTCATATGAAAAAGGAGTTCTAAAATAGAAATCTCCTTTTTCATGTGCAAATTATTTTTCTTTATAACGAAAATATCTAAAGTC
GGCCAATTCACTGGCCGTCGTTTTACAACGTCGTGACTGGGAAAACCCTGGCGTTACCCAACTTAATCGCCTTGCAGCACATCC
CCCTTTCGCCAGCTGGCGTAATAGCGAAGAGGCCCGCACCGATCGCCCTTCCCAACAGTTGCGCAGCCTGAATGGCGAATGGCG
CCTGATGCGGTATTTTCTCCTTACGCATCTGTGCGGTATTTCACACCGCATATGGTGCACTCTCAGTACAATCTGCTCTGATGC
CGCATAGTTAAGCCAGCCCCGACACCCGCCAACACCCGCTGACGCGCCCTGACGGGCTTGTCTGCTCCCGGCATCCGCTTACAG
ACAAGCTGTGACCGTCTCCGGGAGCTGCATGTGTCAGAGGTTTTCACCGTCATCACCGAAACGCGCGAGACGAAAGGGCCTCGT
GATACGCCTATTTTTATAGGTTAATGTCATGATAATAATGGTTTCTTAGACGTCAGGTGGCACTTTTCGGGGAAATGTGCGCGG
AACCCCTATTTGTTTATTTTTCTAAATACATTCAAATATGTATCCGCTCATGAGACAATAACCCTGATAAATGCTTCAATAATC
CATCCTCCAAAGTTGGAGAGTGAGTTTTATGTCGCAAATATTAATGTTTCTGGTGAACCTTATCAAATTTTCGTTGATTTAATA
GAAACATAGCGGTAAAATTAGCAGTAACTTAATAGAACGGAAATGAAAAAAGCCACTCTCATATGCTATTGGCTACCAACCTTT
AGCGAGAATGACTTAATCCTGTACAGCCATACAGGACTTCGACTTATAAGAGGCGCCAACTTCAAATAAGTTATTTGCCTTGTT
TTCGCGAACAAGGCTTATTAGATACACCTATTGTACCGTTACTCTACGAATATTTCAAGTAGTAATTACTAGCATTGTCCGTTA
CTCTACGAATATTTCAAGTAGTAATTACTAGCATTGTCCGTTACTCTACGAATATTTCAAGTAGTAATTACTAGCATTGTCATA
TACATAATAAAACGGATATAAAAGGGCGTTTTCTATACCTAGAAGTCTTGTAAATGTACAGGGCGTTTAGATATAGAGAACGCC
CTTTTTGTGTTCCGTTCCAGTGGAAGCTACCACTTTAAAAAGATGGTCTAGTGTAGCCAATGCAGGAGAGTACACTCGGATATC
AGTTGTCGTTGCATTCAACTGTCTGACGTAAGCGAGGTAAAGGACACAAGCCTTGCATAAAACAAGCCTACGGGATGTAAATCC
TAATAATGATGATAACCAAGACGTTAGCGGCAAAAAGTGTTGGGGGTTCAAAATAAGACATGATTGTGCGACTGGAGTTAAACA
GTTACTCGTAAGCGGCGATCATGACACTGATTCACGGCTATTCTTGTACAAGCTAGCTTTATTACAAGGATATGCGGGTTATAT
AGCGAATCACCCGAAAGGGAACGGTGTTGGGCGTGAGAAACGCACCGTACGGCGCAATACAATGCCAATAAGCTATATACGGAC
GGTATAGTAGTTTTGTAAGCTATAACCGTTTGTCGTCAATGCAACCAATCTCAATTCGAGACCTCGGCATCTAAGCCAGTACGA
```

```
ATGAGTGGGCGTTTTAACCTCGTAAATTTTCAACAGGGGTTACTATGCCCAAAACTACATTCAGATTTCCTAACAAACTCGCCA
GTATGAAAACCTTAAGACCTTAAAGTCAAGGGATTTGAAGGATTTTAACCTCGATTAGCAAAAAATGTAGAGTACTGAAGCAAC
TACCATTAACTAAGATAGTGGGGGATTGAGGAAGAATCCAGAGCTGTTTAAATCAAGTGAAAGACAAGATGAAATTAAAAGAAT
AGTGAAAGATAGGGGAGTGGTTCTCTATGAGAAAGGAAATGGCTAGAGAACAAAGGCAGCGGTTTATTGATCTATTGTTAGACT
TTATGGTAAAGAATCCTCATTTATTTGTTAATGGTACAGAGGATGAAAGTAATAATGTTGTTACAAAATGTAATAGTGATATTA
AAGAGGTTGCGGAGTCATATTTAACTCTTTTATAGTGAGAGGGTTAAAACTAATTAATATGTATTAAGGCCCAATGTTGGAATT
ATTGTATTTCACTAGGCAACCTACTTACTAAAAGTAAGATTATCCATTAGTGGATGTTATAATATTGGGTTTTTTAACACAATA
ATCATCGCCTTTCGGTGTCGTTTGATAGAAAAGTAACCATTAGCGATGAAAAAGTCAATATAAAAAGCCATCCGTAAAAAACGG
ATGGCTTACCGTACATAGGATCGTTGGTAGGGCGGCGTATCCTACATCTCTGGTAACTTACCTAGCCAATCAAATGCTTGAGAA
CGGCGGTTAGATAAGCGCGTGGGGAACCTTTCCCACCTCAAAGATCCTATATCATTATTATGTTACTTTCTACAGGTAGTATAC
CATGTTCTTATATTTTAGTAAACTCCCCGTTAGCTTAACAGGTCTTTGTAAGCAATTAAACGTCCACTATTCAATCGTCTTTGG
ATTTTCGCAGGACCGTTTTTTAGATCGAACATAGTTGATAAGAACAAATAACCGCTTGGGTCCAACTTTATAGCAATTAGTATA
TGGTCATTTAAAATCTTTACCAATTCAACGCTATTAGGTTCTTTAGGATTTTGCCCGACATAGTCGGGGTGTTCAACGATATCT
TTTATGTGCGATGAATATTTTTCATAAATACCAGGATGTTGTTTCTTTACGTGCTTTATAAATCCGGGAAACATTTTTACATCG
TTAGAAGTGCAAGTCAAGTTATATGTATCTATAATGATTTGTGGAAGTTTTGCCACAACAGTTGGTTTATTTACAATCTTTTTT
TTATTAGCCGTCAAATTTCTCCCTCATCTCGTCTCTTTATATCTTTATTTTATCATAAAGGAGTATTTGAACCGTCGCGCGGGA
CAGGTTTATGATAGGGATATTTTATTGAATAATTGATGGTATAAGGGACTTTCATGCTTGGAAAGTGGGGATTATGAATTAGAT
GCTTGTCCACAATATGTTCCAATGTAATTAAAATTTATGTTCCCACCTTGACCAAACATCACGTCCATACTTAAATCGTCCCTC
CTTTAATAGGTAAAATATTAATTTACCTTAATAAAAAAATAATGGATAATAGTATTCGTCTGAATTTATATAATCAGGGGGAAC
TATTGATGCTGGGGATACTATTTACAGCGGCGCCATCTACTGATGTCGTAAAGGATTTGCAAGATAAAGTTATATCATTGCAGG
ATCATGAGGTAGCGTTTTTGAACACCACGATATCTAATATGTTGATCCCCGAAGCAAACTTAAGAGTGTGTTGATAGTGCAGTA
TCTTAAAATTTTGTGTATAATAGGAATTGAAGTTAAATTAGATGCTAAAAATTTGTAATTAAGAAGGAGGGATTCGTCATGTTG
GTATTCCAAATGCGTAATGTAGATAAAACATCTACTGTTTTGAAACAGACTAAAAACAGTGATTACGCAGATAAATAAATACGT
TAGATTAATTCCTACCAGTGACTAATCTTATGACTTTTTAAACAGATAACTAAAATTACAAACAAATCGTTTAACTTCTGTATT
TATTTACAGATGTAATCACTTCAGGAGTAATTACATGAACAAAAATATAAAATATTCTCAAAACTTTTTAACGAGTGAAAAAGT
ACTCAACCAAATAATAAAACAATTGAATTTAAAAGAAACCGATACCGTTTACGAAATTGGAACAGGTAAAGGGCATTTAACGAC
GAAACTGGCTAAAATAAGTAAACAGGTAACGTCTATTGAATTAGACAGTCATCTATTCAACTTATCGTCAGAAAAATTAAAACT
GAACATTCGTGTCACTTTAATTCACCAAGATATTCTACAGTTTCAATTCCCTAACAAACAGAGGTATAAAATTGTTGGGAGTAT
TCCTTACCATTTAAGCACACAAATTATTAAAAAAGTGGTTTTTGAAAGCCATGCGTCTGACATCTATCTGATTGTTGAAGAAGG
ATTCTACAAGCGTACCTTGGATATTCACCGAACACTAGGGTTGCTCTTGCACACTCAAGTCTCGATTCAGCAATTGCTTAAGCT
GCCAGCGGAATGCTTTCATCCTAAACCAAAAGTAAACAGTGTCTTAATAAAACTTACCCGCCATACCACAGATGTTCCAGATAA
ATATTGGAAGCTATATACGTACTTTGTTTCAAAATGGGTCAATCGAGAATATCGTCAACTGTTTACTAAAAATCAGTTTCATCA
AGCAATGAAACACGCCAAAGTAAACAATTTAAGTACCATTACTTATGAGCAAGTATTGTCTATTTTTAATAGTTATCTATTATT
TAACGGGAGGAAATAATTCTATGAGTCGCTTTTTTAAATTTGGAAAGTTACACGTTACTAAAGGGAATGGAGATAAATTATTAG
ATATACTACTGACAGCTTCCAAGAAGCTAAAGAGGTCCCTAGCGCCTACGGGGAATTTGGGGTACATTGAAAAAGGAAGAGTAT
GAGTATTCAACATTTCCGTGTCGCCCTTATTCCCTTTTTTGCGGCATTTTGCCTTCCTGTTTTTGCTCACCCAGAAACGCTGGT
GAAAGTAAAAGATGCTGAAGATCAGTTGGGTGCACGAGTGGGTTACATCGAACTGGATCTCAACAGCGGTAAGATCCTTGAGAG
TTTTCGCCCCGAAGAACGTTTTCCAATGATGAGCACTTTTAAAGTTCTGCTATGTGGCGCGGTATTATCCCGTATTGACGCCGG
GCAAGAGCAACTCGGTCGCCGCATACACTATTCTCAGAATGACTTGGTTGAGTACTCACCAGTCACAGAAAAGCATCTTACGGA
TGGCATGACAGTAAGAGAATTATGCAGTGCTGCCATAACCATGAGTGATAACACTGCGGCCAACTTACTTCTGACAACGATCGG
AGGACCGAAGGAGCTAACCGCTTTTTTGCACAACATGGGGGATCATGTAACTCGCCTTGATCGTTGGGAACCGGAGCTGAATGA
AGCCATACCAAACGACGAGCGTGACACCACGATGCCTGTAGCAATGGCAACAACGTTGCGCAAACTATTAACTGGCGAACTACT
TACTCTAGCTTCCCGGCAACAATTAATAGACTGGATGGAGGCGGATAAAGTTGCAGGACCACTTCTGCGCTCGGCCCTTCCGGC
TGGCTGGTTTATTGCTGATAAATCTGGAGCCGGTGAGCGTGGGTCTCGCGGTATCATTGCAGCACTGGGGCCAGATGGTAAGCC
CTCCCGTATCGTAGTTATCTACACGACGGGGAGTCAGGCAACTATGGATGAACGAAATAGACAGATCGCTGAGATAGGTGCCTC
ACTGATTAAGCATTGGTAACTGTCAGACCAAGTTTACTCATATATACTTTAGATTGATTTAAAACTTCATTTTTAATTTAAAAG
GATCTAGGTGAAGATCCTTTTTGATAATCTCATGACCAAAATCCCTTAACGTGAGTTTTCGTTCCACTGAGCGTCAGACCCCGT
AGAAAAGATCAAAGGATCTTC-3’
```

pXyl-Sa-agrCA-GFP

```
5’-
GAAGATCCTTTGATCTTTTCTACGGGGTCTGACGCTCAGTGGAACGAAAACTCACGTTAAGGGATTTTGGTCATGAGATTATCA
AAAAGGATCTTCACCTAGATCCTTTTAAATTAAAAATGAAGTTTTAAATCAATCTAAAGTATATATGAGTAAACTTGGTCTGAC
AGTTACCAATGCTTAATCAGTGAGGCACCTATCTCAGCGATCTGTCTATTTCGTTCATCCATAGTTGCCTGACTCCCCGTCGTG
TAGATAACTACGATACGGGAGGGCTTACCATCTGGCCCCAGTGCTGCAATGATACCGCGAGACCCACGCTCACCGGCTCCAGAT
TTATCAGCAATAAACCAGCCAGCCGGAAGGGCCGAGCGCAGAAGTGGTCCTGCAACTTTATCCGCCTCCATCCAGTCTATTAAT
TGTTGCCGGGAAGCTAGAGTAAGTAGTTCGCCAGTTAATAGTTTGCGCAACGTTGTTGCCATTGCTACAGGCATCGTGGTGTCA
CGCTCGTCGTTTGGTATGGCTTCATTCAGCTCCGGTTCCCAACGATCAAGGCGAGTTACATGATCCCCCATGTTGTGCAAAAAA
GCGGTTAGCTCCTTCGGTCCTCCGATCGTTGTCAGAAGTAAGTTGGCCGCAGTGTTATCACTCATGGTTATGGCAGCACTGCAT
AATTCTCTTACTGTCATGCCATCCGTAAGATGCTTTTCTGTGACTGGTGAGTACTCAACCAAGTCATTCTGAGAATAGTGTATG
CGGCGACCGAGTTGCTCTTGCCCGGCGTCAATACGGGATAATACCGCGCCACATAGCAGAACTTTAAAAGTGCTCATCATTGGA
AAACGTTCTTCGGGGCGAAAACTCTCAAGGATCTTACCGCTGTTGAGATCCAGTTCGATGTAACCCACTCGTGCACCCAACTGA
```

```
TCTTCAGCATCTTTTACTTTCACCAGCGTTTCTGGGTGAGCAAAAACAGGAAGGCAAAATGCCGCAAAAAAGGGAATAAGGGCG
ACACGGAAATGTTGAATACTCATACTCTTCCTTTTTCAATGTACCCCAAATTCCCCGTAGGCGCTAGGGACCTCTTTAGCTTCT
TGGAAGCTGTCAGTAGTATATCTAATAATTTATCTCCATTCCCTTTAGTAACGTGTAACTTTCCAAATTTAAAAAAGCGACTCA
TAGAATTATTTCCTCCCGTTAAATAATAGATAACTATTAAAAATAGACAATACTTGCTCATAAGTAATGGTACTTAAATTGTTT
ACTTTGGCGTGTTTCATTGCTTGATGAAACTGATTTTTAGTAAACAGTTGACGATATTCTCGATTGACCCATTTTGAAACAAAG
TACGTATATAGCTTCCAATATTTATCTGGAACATCTGTGGTATGGCGGGTAAGTTTTATTAAGACACTGTTTACTTTTGGTTTA
GGATGAAAGCATTCCGCTGGCAGCTTAAGCAATTGCTGAATCGAGACTTGAGTGTGCAAGAGCAACCCTAGTGTTCGGTGAATA
TCCAAGGTACGCTTGTAGAATCCTTCTTCAACAATCAGATAGATGTCAGACGCATGGCTTTCAAAAACCACTTTTTTAATAATT
TGTGTGCTTAAATGGTAAGGAATACTCCCAACAATTTTATACCTCTGTTTGTTAGGGAATTGAAACTGTAGAATATCTTGGTGA
ATTAAAGTGACACGAATGTTCAGTTTTAATTTTTCTGACGATAAGTTGAATAGATGACTGTCTAATTCAATAGACGTTACCTGT
TTACTTATTTTAGCCAGTTTCGTCGTTAAATGCCCTTTACCTGTTCCAATTTCGTAAACGGTATCGGTTTCTTTTAAATTCAAT
TGTTTTATTATTTGGTTGAGTACTTTTTCACTCGTTAAAAAGTTTTGAGAATATTTTATATTTTTGTTCATGTAATTACTCCTG
AAGTGATTACATCTGTAAATAAATACAGAAGTTAAACGATTTGTTTGTAATTTTAGTTATCTGTTTAAAAAGTCATAAGATTAG
TCACTGGTAGGAATTAATCTAACGTATTTATTTATCTGCGTAATCACTGTTTTTAGTCTGTTTCAAAACAGTAGATGTTTTATC
TACATTACGCATTTGGAATACCAACATGACGAATCCCTCCTTCTTAATTACAAATTTTTAGCATCTAATTTAACTTCAATTCCT
ATTATACACAAAATTTTAAGATACTGCACTATCAACACACTCTTAAGTTTGCTTCGGGGATCAACATATTAGATATCGTGGTGT
TCAAAAACGCTACCTCATGATCCTGCAATGATATAACTTTATCTTGCAAATCCTTTACGACATCAGTAGATGGCGCCGCTGTAA
ATAGTATCCCCAGCATCAATAGTTCCCCCTGATTATATAAATTCAGACGAATACTATTATCCATTATTTTTTTATTAAGGTAAA
TTAATATTTTACCTATTAAAGGAGGGACGATTTAAGTATGGACGTGATGTTTGGTCAAGGTGGGAACATAAATTTTAATTACAT
TGGAACATATTGTGGACAAGCATCTAATTCATAATCCCCACTTTCCAAGCATGAAAGTCCCTTATACCATCAATTATTCAATAA
AATATCCCTATCATAAACCTGTCCCGCGCGACGGTTCAAATACTCCTTTATGATAAAATAAAGATATAAAGAGACGAGATGAGG
GAGAAATTTGACGGCTAATAAAAAAAAGATTGTAAATAAACCAACTGTTGTGGCAAAACTTCCACAAATCATTATAGATACATA
TAACTTGACTTGCACTTCTAACGATGTAAAAATGTTTCCCGGATTTATAAAGCACGTAAAGAAACAACATCCTGGTATTTATGA
AAAATATTCATCGCACATAAAAGATATCGTTGAACACCCCGACTATGTCGGGCAAAATCCTAAAGAACCTAATAGCGTTGAATT
GGTAAAGATTTTAAATGACCATATACTAATTGCTATAAAGTTGGACCCAAGCGGTTATTTGTTCTTATCAACTATGTTCGATCT
AAAAAACGGTCCTGCGAAAATCCAAAGACGATTGAATAGTGGACGTTTAATTGCTTACAAAGACCTGTTAAGCTAACGGGGAGT
TTACTAAAATATAAGAACATGGTATACTACCTGTAGAAAGTAACATAATAATGATATAGGATCTTTGAGGTGGGAAAGGTTCCC
CACGCGCTTATCTAACCGCCGTTCTCAAGCATTTGATTGGCTAGGTAAGTTACCAGAGATGTAGGATACGCCGCCCTACCAACG
ATCCTATGTACGGTAAGCCATCCGTTTTTTACGGATGGCTTTTTATATTGACTTTTTCATCGCTAATGGTTACTTTTCTATCAA
ACGACACCGAAAGGCGATGATTATTGTGTTAAAAAACCCAATATTATAACATCCACTAATGGATAATCTTACTTTTAGTAAGTA
GGTTGCCTAGTGAAATACAATAATTCCAACATTGGGCCTTAATACATATTAATTAGTTTTAACCCTCTCACTATAAAAGAGTTA
AATATGACTCCGCAACCTCTTTAATATCACTATTACATTTTGTAACAACATTATTACTTTCATCCTCTGTACCATTAACAAATA
AATGAGGATTCTTTACCATAAAGTCTAACAATAGATCAATAAACCGCTGCCTTTGTTCTCTAGCCATTTCCTTTCTCATAGAGA
ACCACTCCCCTATCTTTCACTATTCTTTTAATTTCATCTTGTCTTTCACTTGATTTAAACAGCTCTGGATTCTTCCTCAATCCC
CCACTATCTTAGTTAATGGTAGTTGCTTCAGTACTCTACATTTTTTGCTAATCGAGGTTAAAATCCTTCAAATCCCTTGACTTT
AAGGTCTTAAGGTTTTCATACTGGCGAGTTTGTTAGGAAATCTGAATGTAGTTTTGGGCATAGTAACCCCTGTTGAAAATTTAC
GAGGTTAAAACGCCCACTCATTCGTACTGGCTTAGATGCCGAGGTCTCGAATTGAGATTGGTTGCATTGACGACAAACGGTTAT
AGCTTACAAAACTACTATACCGTCCGTATATAGCTTATTGGCATTGTATTGCGCCGTACGGTGCGTTTCTCACGCCCAACACCG
TTCCCTTTCGGGTGATTCGCTATATAACCCGCATATCCTTGTAATAAAGCTAGCTTGTACAAGAATAGCCGTGAATCAGTGTCA
TGATCGCCGCTTACGAGTAACTGTTTAACTCCAGTCGCACAATCATGTCTTATTTTGAACCCCCAACACTTTTTGCCGCTAACG
TCTTGGTTATCATCATTATTAGGATTTACATCCCGTAGGCTTGTTTTATGCAAGGCTTGTGTCCTTTACCTCGCTTACGTCAGA
CAGTTGAATGCAACGACAACTGATATCCGAGTGTACTCTCCTGCATTGGCTACACTAGACCATCTTTTTAAAGTGGTAGCTTCC
ACTGGAACGGAACACAAAAAGGGCGTTCTCTATATCTAAACGCCCTGTACATTTACAAGACTTCTAGGTATAGAAAACGCCCTT
TTATATCCGTTTTATTATGTATATGACAATGCTAGTAATTACTACTTGAAATATTCGTAGAGTAACGGACAATGCTAGTAATTA
CTACTTGAAATATTCGTAGAGTAACGGACAATGCTAGTAATTACTACTTGAAATATTCGTAGAGTAACGGTACAATAGGTGTAT
CTAATAAGCCTTGTTCGCGAAAACAAGGCAAATAACTTATTTGAAGTTGGCGCCTCTTATAAGTCGAAGTCCTGTATGGCTGTA
CAGGATTAAGTCATTCTCGCTAAAGGTTGGTAGCCAATAGCATATGAGAGTGGCTTTTTTCATTTCCGTTCTATTAAGTTACTG
CTAATTTTACCGCTATGTTTCTATTAAATCAACGAAAATTTGATAAGGTTCACCAGAAACATTAATATTTGCGACATAAAACTC
ACTCTCCAACTTTGGAGGATGGATTATTGAAGCATTTATCAGGGTTATTGTCTCATGAGCGGATACATATTTGAATGTATTTAG
AAAAATAAACAAATAGGGGTTCCGCGCACATTTCCCCGAAAAGTGCCACCTGACGTCTAAGAAACCATTATTATCATGACATTA
ACCTATAAAAATAGGCGTATCACGAGGCCCTTTCGTCTCGCGCGTTTCGGTGATGACGGTGAAAACCTCTGACACATGCAGCTC
CCGGAGACGGTCACAGCTTGTCTGTAAGCGGATGCCGGGAGCAGACAAGCCCGTCAGGGCGCGTCAGCGGGTGTTGGCGGGTGT
CGGGGCTGGCTTAACTATGCGGCATCAGAGCAGATTGTACTGAGAGTGCACCATATGCGGTGTGAAATACCGCACAGATGCGTA
AGGAGAAAATACCGCATCAGGCGCCATTCGCCATTCAGGCTGCGCAACTGTTGGGAAGGGCGATCGGTGCGGGCCTCTTCGCTA
TTACGCCAGCTGGCGAAAGGGGGATGTGCTGCAAGGCGATTAAGTTGGGTAACGCCAGGGTTTTCCCAGTCACGACGTTCAAAA
AACCCCTCAAGACCCGTTTAGAGGCCCCAAGGGGTTATGCTAGTTATTGCTCAGCGGTGGCAGCAGCCTAGGTTATTTGTATAG
TTCATCCATGCCATGTGTAATCCCAGCAGCTGTTACAAACTCAAGAAGGACCATGTGGTCTCTCTTTTCGTTGGGATCTTTCGA
AAGCTTAGATTGTGTGGACAGGTAATGGTTGTCTGGTAAAAGGACAGGGCCATCGCCAATTGGAGTATTTTGTTGATAATGGTC
TGCTAGTTGAACGCTTCCATCTTCAATGTTGTGTCTAATTTTGAAGTTAACTTTGATTCCATTCTTTTGTTTGTCTGCCATGAT
GTATACATTGTGTGAGTTATAGTTGTATTCCAATTTGTGTCCAAGAATGTTTCCATCTTCTTTAAAATCAATACCTTTTAACTC
GATTCTATTAACAAGGGTATCACCTTCAAACTTGACTTCAGCACGTGTCTTGTAGTTCCCGTCATCTTTGAAAAATATAGTTCT
TTCCTGTACATAACCTTCGGGCATGGCACTCTTGAAAAAGTCATGCTGTTTCATATGATCTGGGTATCTCGCAAAGCATTGAAG
ACCATACGCGAAAGTAGTGACAAGTGTTGGCCATGGAACAGGTAGTTTTCCAGTAGTGCAAATAAATTTAAGGGTAAGTTTTCC
```

GTATGTTGCATCACCTTCACCCTCTCCACTGACAGAAAATTTGTGCCCATTAACATCACCATCTAATTCAACAAGAATTGGGAC
AACTCCAGTGAAAAGTTCTTCTCCTTTCATTGTACATTTCACCTCCTTGGTTAATTAAGACTGACTGACCACATCTCTGTGATC
TAGTTATATTAAAACATGCTAAAAGCATTTATTTTCCAATTTTTCTTAACTAGTCGTTTTTTATTCTTAACTGTAAATTTTTTT
ATGTTAAAATATTAAATACAAATTACATTTAACAGTTAAGTATTTATTTCCTACAGTTAGGCAATATAATGATAAAAGATTGTA
CTAAATCGTATAATGACAGTGAAGCCAGGCATTAACCGGGCAGGCCATGTCTGCCCGTATTTCGGTAAAACGACGGCCAGTGAA
TTGGCCGACTTTAGATATTTTCGTTATAAAGAAAAATAATTTGCACATGAAAAAGGAGATTTCTATTTTAGAACTCCTTTTTCA
TATGAGAAGGTGCCATGTCACTATTGCTTCAGAAATACTCCTAGAATAAAAAAACTCATCTTTAAAGATGAGCTGTCCATTCCA
TAAAAAATTACATTGTAATCATGTCCAGAAAATGATCAATCACAATGGAGGACATTCCTAATGCCGGTGCATTCTGTCCTAAGG
AAGATGGCAATAATTCATAGCTATTGCCTAATTGGGAATAAACCCTTGATGATACTTCACTTCTCATTGAATTTAAAACCATAG
GATGCGATTCAATTATGCTATTTCTTAAAATTACGGCTTGTGGGTTGAAAGTATTTAGAATATTGGTAAGGCCTATTCCTAAAT
AGAATCCAAAATTTTGTAATGCATTTAAGGTTCCGATATCATTCAGATGGGCGAGGTTTATGATATCTTGATAGGACAGTTTTT
TCTCTTTGGTCTGAAGAGATTTTAATAAAGCCTTCTCTGAAGCATACAATTCCCAGCATCCTCGGTTTCCGCAACTGCATTTAG
GACCATTAAAGTCTATTGTCATATGTCCCATTTCTCCAGAGAAGCCGCTTACTCCTCTATATAAATGATTGTTGATAATAACAC
CGATCCCTATTCCTGTGCTGATACTTACGTAAATAATGTTATCGTGATTTTTTGCAGCTCCAAATACTTTTTCTCCATATGCGC
CAGCATTTGCCTCATTTTCAATAAAAACAGGCACATTGTACTTCTCTTGTATCGAAGATTTTAAGTCAATATCTCTCCAGTTGG
AGTTCGGAGTGAAAACAATTTTTTGATCTTTATCAATGAGTCCAGGCACGCAAATACCTATACCAATAAGCCCGTACGGAGATT
GGGGCATTTGCGTAATAAAGTGATGAATCATATCAATCAAAATGTCTTTCGTTATTTCTGGAGAATTGGATTCCAAATGGCGGT
ATTGATCAAGAACGATTGTTCCTTCAAGGTCTGTTAAAATGCCATTAATATAATCCACACCAACATCTATTCCAACGGAGTATC
CTGCCTTTTTATTAAAAACAAGCATGACAGGTCTTCTTCCGCCACTTGATTGTCCTTGACCTATTTCAAATACCATACTTTCTT
TCATTAACGTGTTTACCTGTGATGAGACAGTTGATTTATTTAATCCAGTCATTTCAGATAATTTTGCTCTTGAAATAGGTGAAT
TTTTAAGGATTTCTTTTAATAATAACTTTTGATTTACTTTTTTGACAAAGGTTTGATCAGCGATATCCACTTCATCCACTCCAT
TTGTTTAATCTTTAAATTAAGTATCAACATAGTACATAGCGAATCTTCCCTTTATTATATCTAATGTGTTCATAAAAAACTAAA
AAAAATATTGAAAATACTGACGAGGTTATATAAGATGAAAATAAGTTAGTTTGTTTAAACAACAAACTAATAGGTGATGTACTT
ACTATATGAAATAAAATGCATCTGTAGATAAGGAGGAACTACTATGGAATTATTAAATAGTTATAATTTTGTTTTATTCGTATT
AACTCAAATGATATTAATGTTTACAATACCAGCTATAATTAGTGGTATTAAGTACAGTAAACTTGATTATTTTTTCATCATAGT
AATTTCGACATTATCGTTATTTCTATTTAAAATGTTTGATAGCGCGTCCTTAATCATATTAACTTCATTTATTATTATAATGTA
TTTTGTCAAAATCAAATGGTATTCTATTTTGTTGATTATGACTTCGCAGATTATTCTATACTGTGCTAACTACATGTATATAGT
TATATATGCATATATCACCAAAATTTCTGATAGTATATTTGTAATATTCCCTAGCTTTTTTGTAGTTTATGTGACTATTAGTAT
ACTATTCTCATATATAATAAATAGAGTTCTCAAAAAAATTAGCACACCATATCTAATACTAAACAAAGGATTTTTAATAGTTAT
TTCGACTATCTTACTGCTTACTTTTTCATTATTTTTCTTTTATTCACAAATAAACTCGGATGAAGCTAAAGTAATAAGGCAGTA
TTCTTTTATTTTTATTGGTATCACTATATTTTTAAGTATATTAACATTTGTTATTTCTCAATTTCTCCTTAAAGAGATGAAATA
TAAACGTAATCAAGAAGAAATTGAAACCTATTATGAATATACATTGAAGATTGAAGCTATCAACAACGAAATGCGCAAGTTCCG
TCATGATTATGTCAATATCTTAACGACACTTTCAGAATACATTCGAGAAGATGACATGCCTGGCCTACGTGATTATTTCAATAA
AAATATTGTACCTATGAAAGACAATTTACAAATGAATGCTATAAAATTAAATGGTATCGAGAATCTTAAAGTACGTGAAATTAA
AGGCTTAATTACTGCGAAAATTTTACGTGCACAAGAAATGAATATTCCGATTAGTATCGAAATACCCGATGAAGTAAGTAGCAT
TAACTTGAATATGATCGATTTAAGTCGCAGTATTGGTATTATTCTTGATAATGCAATTGAGGCATCAACTGAAATTGATGACCC
TATCATTCGCGTTGCATTTATTGAAAGTGAAAATTCAGTAACGTTTATTGTTATGAATAAATGCGCTGATGATATACCACGCAT
TCATGAATTGTTCCAAGAAAGTTTTTCTACTAAAGGTGAAGGTCGTGGTTTAGGTCTATCAACTTTAAAAGAAATTGCTGATAA
TGCAGACAATGTCTTATTAGATACAATTATCGAAAATGGTTTCTTTATTCAAAAAGTTGAAATTATTAACAACTAGCCATAAGG
ATGTGAATGTATGAAAATTTTCATTTGCGAAGACGATCCAAAACAAAGAGAAAACATGGTTACCATTATTAAAAATTATATAAT
GATAGAAGAAAAGCCTATGGAAATTGCCCTCGCAACTGATAATCCTTATGAGGTGCTTGAGCAAGCTAAAAATATGAATGACAT
AGGCTGTTACTTTTTAGATATTCAACTTTCAACTGATATTAATGGTATCAAATTAGGCAGTGAAATTCGTAAGCATGACCCAGT
TGGTAACATTATTTTCGTTACGAGTCACAGTGAACTTACCTATTTAACATTTGTCTACAAAGTTGCAGCGATGGATTTTATTTT
TAAAGATGATCCAGCTGAATTAAGAACTCGAATTATAGACTGTTTAGAAACTGCACATACACGCTTACAATTGTTGTCTAAAGA
TAATAGCGTTGAAACGATTGAATTAAAACGTGGCAGTAATTCAGTGTATGTTCAATATGATGATATTATGTTTTTTGAATCATC
AACAAAATCTCACAGACTCATTGCCCATTTAGATAACCGTCAAATTGAATTTTATGGTAATTTAAAAGAACTGAGTCAATTAGA
TGATCGTTTCTTTAGATGTCATAATAGCTTTGTCGTCAATCGCCATAATATTGAATCTATAGATTCGAAAGAGCGAATTGTCTA
TTTTAAAAAATAAAGAACACTGCTATGCATCGGTGAGAAACGTTAAAAAAATATAATGCAGGCATGCAAGCTTGGCGTAATCATG
GTCATAGCTGTTTCCTGTGTGAAATTGTTATCCGCTCACAATTCCACACAACATACGAGCCGGAAGCATAAAGTGTAAAGCCTG
GGGTGCCTAATGAGTGAGCTAACTCACATTAATTGCGTTGCGCTCACTGCCCGCTTTCCAGTCGGGAAACCTGTCGTGCCAGCT
GCATTAATGAATCGGCCAACGCGCGGGGAGAGGCGGTTTGCGTATTGGGCGCTCTTCCGCTTCCTCGCTCACTGACTCGCTGCG
CTCGGTCGTTCGGCTGCGGCGAGCGGTATCAGCTCACTCAAAGGCGGTAATACGGTTATCCACAGAATCAGGGGATAACGCAGG
AAAGAACATGTGAGCAAAAGGCCAGCAAAAGGCCAGGAACCGTAAAAAGGCCGCGTTGCTGGCGTTTTTCCATAGGCTCCGCCC
CCCTGACGAGCATCACAAAAATCGACGCTCAAGTCAGAGGTGGCGAAACCCGACAGGACTATAAAGATACCAGGCGTTTCCCCC
TGGAAGCTCCCTCGTGCGCTCTCCTGTTCCGACCCTGCCGCTTACCGGATACCTGTCCGCCTTTCTCCCTTCGGGAAGCGTGGC
GCTTTCTCATAGCTCACGCTGTAGGTATCTCAGTTCGGTGTAGGTCGTTCGCTCCAAGCTGGGCTGTGTGCACGAACCCCCCGT
TCAGCCCGACCGCTGCGCCTTATCCGGTAACTATCGTCTTGAGTCCAACCCGGTAAGACACGACTTATCGCCACTGGCAGCAGC
CACTGGTAACAGGATTAGCAGAGCGAGGTATGTAGGCGGTGCTACAGAGTTCTTGAAGTGGTGGCCTAACTACGGCTACACTAG
AAGAACAGTATTTGGTATCTGCGCTCTGCTGAAGCCAGTTACCTTCGGAAAAAGAGTTGGTAGCTCTTGATCCGGCAAACAAAC
CACCGCTGGTAGCGGTGGTTTTTTTGTTTGCAAGCAGCAGATTACGCGCAGAAAAAAAGGATCTCAA-3’

pXyl-Sa-agrBDCA-GFP
5′-<br>TTGAGATCCTTTTTTTCTGCGCGTAATCTGCTGCTTGCAAACAAAAAAACCACCGCTACCAGCGGTGGTTTGTTTGCCGGATCA<br>AGAGCTACCAACTCTTTTTCCGAAGGTAACTGGCTTCAGCAGAGCGCAGATACCAAATACTGTTCTTCTAGTGTAGCCGTAGTT<br>AGGCCACCACTTCAAGAACTCTGTAGCACCGCCTACATACCTCGCTCTGCTAATCCTGTTACCAGTGGCTGCTGCCAGTGGCGA<br>TAAGTCGTGTCTTACCGGGTTGGACTCAAGACGATAGTTACCGGATAAGGCGCAGCGGTCGGGCTGAACGGGGGGTTCGTGCAC<br>ACAGCCCAGCTTGGAGCGAACGACCTACACCGAACTGAGATACCTACAGCGTGAGCTATGAGAAAGCGCCACGCTTCCCGAAGG<br>GAGAAAGGCGGACAGGTATCCGGTAAGCGGCAGGGTCGGAACAGGAGAGCGCACGAGGGAGCTTCCAGGGGGAAACGCCTGGTA<br>TCTTTATAGTCCTGTCGGGTTTCGCCACCTCTGACTTGAGCGTCGATTTTTGTGATGCTCGTCAGGGGGGCGGAGCCTATGGAA<br>AAACGCCAGCAACGCGGCCTTTTTACGGTTCCTGGCCTTTTGCTGGCCTTTTGCTCACATGTTCTTTCCTGCGTTATCCCCTGA<br>TTCTGTGGATAACCGTATTACCGCCTTTGAGTGAGCTGATACCGCTCGCCGCAGCCGAACGACCGAGCGCAGCGAGTCAGTGAG<br>CGAGGAAGCGGAAGAGCGCCCAATACGCAAACCGCCTCTCCCCGCGCGTTGGCCGATTCATTAATGCAGCTGGCACGACAGGTT<br>TCCCGACTGGAAAGCGGGCAGTGAGCGCAACGCAATTAATGTGAGTTAGCTCACTCATTAGGCACCCCAGGCTTTACACTTTAT<br>GCTTCCGGCTCGTATGTTGTGTGAATTGTGAGCGGATAACAATTTCACACAGGAAACAGCTATGGCCATGATTACGCCAAGCTT<br>GCATGCCTGCATTATATTTTTTTAACGTTTCTCACCGATGCATAGCAGTGTTCTTTATTTTTAAAATAGACAATTCGCTCTTTC<br>GAATCTATAGATTCAATATTATGGCGATTGACGACAAAGCTATTATGACATCTAAAGAAACGATCATCTAATTGACTCAGTTCT<br>TTTAAATTACCATAAAATTCAATTTGACGGTTATCTAAATGGGCAATGAGTCTGTGAGATTTTGTTGATGATTCAAAAAACATA<br>ATATCATCATATTGAACATACACTGAATTACTGCCACGTTTTAATTCAATCGTTTCAACGCTATTATCTTTAGACAACAATTGT<br>AAGCGTGTATGTGCAGTTTCTAAACAGTCTATAATTCGAGTTCTTAATTCAGCTGGATCATCTTTAAAAATAAAATCCATCGCT<br>GCAACTTTGTAGACAAATGTTAAATAGGTAAGTTCACTGTGACTCGTAACGAAAATAATGTTACCAACTGGGTCATGCTTACGA<br>ATTTCACTGCCTAATTTGATACCATTAATATCAGTTGAAAGTTGAATATCTAAAAAGTAACAGCCTATGTCATTCATATTTTTA<br>GCTTGCTCAAGCACCTCATAAGGATTATCAGTTGCGAGGGCAATTTCCATAGGCTTTTCTTCTATCATTATATAATTTTTAATA<br>ATGGTAACCATGTTTTCTCTTTGTTTTGGATCGTCTTCGCAAATGAAAATTTTCATACATTCACATCCTTATGGCTAGTTGTTA<br>ATAATTTCAACTTTTTGAATAAAGAAACCATTTTCGATAATTGTATCTAATAAGACATTGTCTGCATTATCAGCAATTTCTTTT<br>AAAGTTGATAGACCTAAACCACGACCTTCACCTTTAGTAGAAAAACTTTCTTGGAACAATTCATGAATGCGTGGTATATCATCA<br>GCGCATTTATTCATAACAATAAACGTTACTGAATTTTCACTTTCAATAAATGCAACGCGAATGATAGGGTCATCAATTTCAGTT<br>GATGCCTCAATTGCATTATCAAGAATAATACCAATACTGCGACTTAAATCGATCATATTCAAGTTAATGCTACTTACTTCATCG<br>GGTATTTCGATACTAATCGGAATATTCATTTCTTGTGCACGTAAAATTTTCGCAGTAATTAAGCCTTTAATTTCACGTACTTTA<br>AGATTCTCGATACCATTTAATTTTATAGCATTCATTTGTAAATTGTCTTTCATAGGTACAATATTTTTATTGAAATAATCACGT<br>AGGCCAGGCATGTCATCTTCTCGAATGTATTCTGAAAGTGTCGTTAAGATATTGACATAATCATGACGGAACTTGCGCATTTCG<br>TTGTTGATAGCTTCAATCTTCAATGTATATTCATAATAGGTTTCAATTTCTTCTTGATTACGTTTATATTTCATCTCTTTAAGG<br>AGAAATTGAGAAATAACAAATGTTAATATACTTAAAAATATAGTGATACCAATAAAAATAAAAGAATACTGCCTTATTACTTTA<br>GCTTCATCCGAGTTTATTTGTGAATAAAAGAAAAATAATGAAAAAGTAAGCAGTAAGATAGTCGAAATAACTATTAAAAATCCT<br>TTGTTTAGTATTAGATATGGTGTGCTAATTTTTTTGAGAACTCTATTTATTATATATGAGAATAGTATACTAATAGTCACATAA<br>ACTACAAAAAAGCTAGGGAATATTACAAATATACTATCAGAAATTTTGGTGATATATGCATATATAACTATATACATGTAGTTA<br>GCACAGTATAGAATAATCTGCGAAGTCATAATCAACAAAATAGAATACCATTTGATTTTGACAAAATACATTATAATAATAAAT<br>GAAGTTAATATGATTAAGGACGCGCTATCAAACATTTTAAATAGAAATAACGATAATGTCGAAATTACTATGATGAAAAAATAA<br>TCAAGTTTACTGTACTTAATACCACTAATTATAGCTGGTATTGTAAACATTAATATCATTTGAGTTAATACGAATAAAACAAAA<br>TTATAACTATTTAATAATTCCACCTACTATCACACTCTCTATTTAAATTATTCGTGTAATTGTGTTAATTCTTTTGGTACTTCA<br>ACTTCATCCATTATGAAGTCACAAGTACTATAAGCTGCGATGTTACCAATGTTTTTTAAAATCCCAGTAATAAAATCAAAAAAT<br>AAGTTAAATAATGTATTCATTTTAAGTCCTCCTTAATAAAGAAAATAGGTAATAATGTAATAGCTTCTATTATGATGCCTAATT<br>GAATGAATTGGGCAAATGGCTCTTTGATGATAAGTGTGATAATGAAAAGGGTTAAACTAACAATAATCGCATAATATTTTTTTC<br>GTTTAATAAGTCGCACAGGAATGGGCTTCTTTTTAGTTGCTGCAGGAGCATATACTGAGATTACACCTAAAGAAATAACTGTTA<br>AAATAATCATAATTAAAAAGTTAATATGAAAATTTACTATTACTAAAGGTAAAAGTATAAATAGTATAATACTTTCTACATAAC<br>ACCAAAAAGAAGAAGGTGCATGTGCACCATGTGCATGTCTTCTTATTAAATAAAATGTTAAATTCGTAATTAACGTAAACAGAA<br>AAATGTTTAAAATATAGGCAATAGTATACATAACAATTAATTTACCTATATTTTTAGCTAAGACCTGCATCCCTAATCGTACTT<br>GCAAAAATTGAATATGATCTAAGTTATTTCTCTTTTGAAGATACGTGGCAAACTGGTCAATTTTATTATCAAAATAATTCATAG<br>TAGTTCCTCCTTATCTACAGATGCATTTTATTTCATATAGTAAGTACATCACCTATTAGTTTGTTGTTTAAACAAACTAACTTA<br>TTTTCATCTTATATAACCTCGTCAGTATTTTCAATATTTTTTTTAGTTTTTTATGAACACATTAGATATAATAAAGGGAAGATT<br>CGCTATGTACTATGTTGATACTTAATTTAAAGATTAAACAAATGGAGTGGATGAAGTGGATATCGCTGATCAAACCTTTGTCAA<br>AAAAGTAAATCAAAAGTTATTATTAAAAGAAATCCTTAAAAATTCACCTATTTCAAGAGCAAAATTATCTGAAATGACTGGATT<br>AAATAAATCAACTGTCTCATCACAGGTAAACACGTTAATGAAAGAAAGTATGGTATTTGAAATAGGTCAAGGACAATCAAGTGG<br>CGGAAGAAGACCTGTCATGCTTGTTTTTAATAAAAAGGCAGGATACTCCGTTGGAATAGATGTTGGTGTGGATTATATTAATGG<br>CATTTTAACAGACCTTGAAGGAACAATCGTTCTTGATCAATACCGCCATTTGGAATCCAATTCTCCAGAAATAACGAAAGACAT<br>TTTGATTGATATGATTCATCACTTTATTACGCAAATGCCCCAATCTCCGTACGGGCTTATTGGTATAGGTATTTGCGTGCCTGG<br>ACTCATTGATAAAGATCAAAAAATTGTTTTCACTCCGAACTCCAACTGGAGAGATATTGACTTAAAATCTTCGATACAAGAGAA<br>GTACAATGTGCCTGTTTTTATTGAAAATGAGGCAAATGCTGGCGCATATGGAGAAAAAGTATTTGGAGCTGCAAAAAATCACGA<br>TAACATTATTTACGTAAGTATCAGCACAGGAATAGGGATCGGTGTTATTATCAACAATCATTTATATAGAGGAGTAAGCGGCTT<br>CTCTGGAGAAATGGGACATATGACAATAGACTTTAATGGTCCTAAATGCAGTTGCGGAAACCGAGGATGCTGGGAATTGTATGC<br>TTCAGAGAAGGCTTTATTAAAATCTCTTCAGACCAAAGAGAAAAAACTGTCCTATCAAGATATCATAAACCTCGCCCATCTGAA<br>TGATATCGGAACCTTAAATGCATTACAAAATTTTGGATTCTATTTAGGAATAGGCCTTACCAATATTCTAAATACTTTCAACCC<br>ACAAGCCGTAATTTTAAGAAATAGCATAATTGAATCGCATCCTATGGTTTTAAATTCAATGAGAAGTGAAGTATCATCAAGGGT

```
TTATTCCCAATTAGGCAATAGCTATGAATTATTGCCATCTTCCTTAGGACAGAATGCACCGGCATTAGGAATGTCCTCCATTGT
GATTGATCATTTTCTGGACATGATTACAATGTAATTTTTTATGGAATGGACAGCTCATCTTTAAAGATGAGTTTTTTTATTCTA
GGAGTATTTCTGAAGCAATAGTGACATGGCACCTTCTCATATGAAAAAGGAGTTCTAAAATAGAAATCTCCTTTTTCATGTGCA
AATTATTTTTCTTTATAACGAAAATATCTAAAGTCGGCCAATTCACTGGCCGTCGTTTTACCGAAATACGGGCAGACATGGCCT
GCCCGGTTAATGCCTGGCTTCACTGTCATTATACGATTTAGTACAATCTTTTATCATTATATTGCCTAACTGTAGGAAATAAAT
ACTTAACTGTTAAATGTAATTTGTATTTAATATTTTAACATAAAAAAATTTACAGTTAAGAATAAAAAACGACTAGTTAAGAAA
AATTGGAAAATAAATGCTTTTAGCATGTTTTAATATAACTAGATCACAGAGATGTGGTCAGTCAGTCTTAATTAACCAAGGAGG
TGAAATGTACAATGAAAGGAGAAGAACTTTTCACTGGAGTTGTCCCAATTCTTGTTGAATTAGATGGTGATGTTAATGGGCACA
AATTTTCTGTCAGTGGAGAGGGTGAAGGTGATGCAACATACGGAAAACTTACCCTTAAATTTATTTGCACTACTGGAAAACTAC
CTGTTCCATGGCCAACACTTGTCACTACTTTCGCGTATGGTCTTCAATGCTTTGCGAGATACCCAGATCATATGAAACAGCATG
ACTTTTTCAAGAGTGCCATGCCCGAAGGTTATGTACAGGAAAGAACTATATTTTTCAAAGATGACGGGAACTACAAGACACGTG
CTGAAGTCAAGTTTGAAGGTGATACCCTTGTTAATAGAATCGAGTTAAAAGGTATTGATTTTAAAGAAGATGGAAACATTCTTG
GACACAAATTGGAATACAACTATAACTCACACAATGTATACATCATGGCAGACAAACAAAAGAATGGAATCAAAGTTAACTTCA
AAATTAGACACAACATTGAAGATGGAAGCGTTCAACTAGCAGACCATTATCAACAAAATACTCCAATTGGCGATGGCCCTGTCC
TTTTACCAGACAACCATTACCTGTCCACACAATCTAAGCTTTCGAAAGATCCCAACGAAAAGAGAGACCACATGGTCCTTCTTG
AGTTTGTAACAGCTGCTGGGATTACACATGGCATGGATGAACTATACAAATAACCTAGGCTGCTGCCACCGCTGAGCAATAACT
AGCATAACCCCTTGGGGCCTCTAAACGGGTCTTGAGGGGTTTTTTGAACGTCGTGACTGGGAAAACCCTGGCGTTACCCAACTT
AATCGCCTTGCAGCACATCCCCCTTTCGCCAGCTGGCGTAATAGCGAAGAGGCCCGCACCGATCGCCCTTCCCAACAGTTGCGC
AGCCTGAATGGCGAATGGCGCCTGATGCGGTATTTTCTCCTTACGCATCTGTGCGGTATTTCACACCGCATATGGTGCACTCTC
AGTACAATCTGCTCTGATGCCGCATAGTTAAGCCAGCCCCGACACCCGCCAACACCCGCTGACGCGCCCTGACGGGCTTGTCTG
CTCCCGGCATCCGCTTACAGACAAGCTGTGACCGTCTCCGGGAGCTGCATGTGTCAGAGGTTTTCACCGTCATCACCGAAACGC
GCGAGACGAAAGGGCCTCGTGATACGCCTATTTTTATAGGTTAATGTCATGATAATAATGGTTTCTTAGACGTCAGGTGGCACT
TTTCGGGGAAATGTGCGCGGAACCCCTATTTGTTTATTTTTCTAAATACATTCAAATATGTATCCGCTCATGAGACAATAACCC
TGATAAATGCTTCAATAATCCATCCTCCAAAGTTGGAGAGTGAGTTTTATGTCGCAAATATTAATGTTTCTGGTGAACCTTATC
AAATTTTCGTTGATTTAATAGAAACATAGCGGTAAAATTAGCAGTAACTTAATAGAACGGAAATGAAAAAAGCCACTCTCATAT
GCTATTGGCTACCAACCTTTAGCGAGAATGACTTAATCCTGTACAGCCATACAGGACTTCGACTTATAAGAGGCGCCAACTTCA
AATAAGTTATTTGCCTTGTTTTCGCGAACAAGGCTTATTAGATACACCTATTGTACCGTTACTCTACGAATATTTCAAGTAGTA
ATTACTAGCATTGTCCGTTACTCTACGAATATTTCAAGTAGTAATTACTAGCATTGTCCGTTACTCTACGAATATTTCAAGTAG
TAATTACTAGCATTGTCATATACATAATAAAACGGATATAAAAGGGCGTTTTCTATACCTAGAAGTCTTGTAAATGTACAGGGC
GTTTAGATATAGAGAACGCCCTTTTTGTGTTCCGTTCCAGTGGAAGCTACCACTTTAAAAAGATGGTCTAGTGTAGCCAATGCA
GGAGAGTACACTCGGATATCAGTTGTCGTTGCATTCAACTGTCTGACGTAAGCGAGGTAAAGGACACAAGCCTTGCATAAAACA
AGCCTACGGGATGTAAATCCTAATAATGATGATAACCAAGACGTTAGCGGCAAAAAGTGTTGGGGGTTCAAAATAAGACATGAT
TGTGCGACTGGAGTTAAACAGTTACTCGTAAGCGGCGATCATGACACTGATTCACGGCTATTCTTGTACAAGCTAGCTTTATTA
CAAGGATATGCGGGTTATATAGCGAATCACCCGAAAGGGAACGGTGTTGGGCGTGAGAAACGCACCGTACGGCGCAATACAATG
CCAATAAGCTATATACGGACGGTATAGTAGTTTTGTAAGCTATAACCGTTTGTCGTCAATGCAACCAATCTCAATTCGAGACCT
CGGCATCTAAGCCAGTACGAATGAGTGGGCGTTTTAACCTCGTAAATTTTCAACAGGGGTTACTATGCCCAAAACTACATTCAG
ATTTCCTAACAAACTCGCCAGTATGAAAACCTTAAGACCTTAAAGTCAAGGGATTTGAAGGATTTTAACCTCGATTAGCAAAAA
ATGTAGAGTACTGAAGCAACTACCATTAACTAAGATAGTGGGGGATTGAGGAAGAATCCAGAGCTGTTTAAATCAAGTGAAAGA
CAAGATGAAATTAAAAGAATAGTGAAAGATAGGGGAGTGGTTCTCTATGAGAAAGGAAATGGCTAGAGAACAAAGGCAGCGGTT
TATTGATCTATTGTTAGACTTTATGGTAAAGAATCCTCATTTATTTGTTAATGGTACAGAGGATGAAAGTAATAATGTTGTTAC
AAAATGTAATAGTGATATTAAAGAGGTTGCGGAGTCATATTTAACTCTTTTATAGTGAGAGGGTTAAAACTAATTAATATGTAT
TAAGGCCCAATGTTGGAATTATTGTATTTCACTAGGCAACCTACTTACTAAAAGTAAGATTATCCATTAGTGGATGTTATAATA
TTGGGTTTTTAACACAATAATCATCGCCTTTCGGTGTCGTTTGATAGAAAAGTAACCATTAGCGATGAAAAAGTCAATATAAA
AAGCCATCCGTAAAAAACGGATGGCTTACCGTACATAGGATCGTTGGTAGGGCGGCGTATCCTACATCTCTGGTAACTTACCTA
GCCAATCAAATGCTTGAGAACGGCGGTTAGATAAGCGCGTGGGGAACCTTTCCCACCTCAAAGATCCTATATCATTATTATGTT
ACTTTCTACAGGTAGTATACCATGTTCTTATATTTTAGTAAACTCCCCGTTAGCTTAACAGGTCTTTGTAAGCAATTAAACGTC
CACTATTCAATCGTCTTTGGATTTTCGCAGGACCGTTTTTTAGATCGAACATAGTTGATAAGAACAAATAACCGCTTGGGTCCA
ACTTTATAGCAATTAGTATATGGTCATTTAAAATCTTTACCAATTCAACGCTATTAGGTTCTTTAGGATTTTGCCCGACATAGT
CGGGGTGTTCAACGATATCTTTTATGTGCGATGAATATTTTTCATAAATACCAGGATGTTGTTTCTTTACGTGCTTTATAAATC
CGGGAAACATTTTTACATCGTTAGAAGTGCAAGTCAAGTTATATGTATCTATAATGATTTGTGGAAGTTTTGCCACAACAGTTG
GTTTATTTACAATCTTTTTTTTATTAGCCGTCAAATTTCTCCCTCATCTCGTCTCTTTATATCTTTATTTTATCATAAAGGAGT
ATTTGAACCGTCGCGCGGGACAGGTTTATGATAGGGATATTTTATTGAATAATTGATGGTATAAGGGACTTTCATGCTTGGAAA
GTGGGGATTATGAATTAGATGCTTGTCCACAATATGTTCCAATGTAATTAAAATTTATGTTCCCACCTTGACCAAACATCACGT
CCATACTTAAATCGTCCCTCCTTTAATAGGTAAAATATTAATTTACCTTAATAAAAAAATAATGGATAATAGTATTCGTCTGAA
TTTATATAATCAGGGGGAACTATTGATGCTGGGGATACTATTTACAGCGGCGCCATCTACTGATGTCGTAAAGGATTTGCAAGA
TAAAGTTATATCATTGCAGGATCATGAGGTAGCGTTTTTGAACACCACGATATCTAATATGTTGATCCCCGAAGCAAACTTAAG
AGTGTGTTGATAGTGCAGTATCTTAAAATTTTGTGTATAATAGGAATTGAAGTTAAATTAGATGCTAAAAATTTGTAATTAAGA
AGGAGGGATTCGTCATGTTGGTATTCCAAATGCGTAATGTAGATAAAACATCTACTGTTTTGAAACAGACTAAAAACAGTGATT
ACGCAGATAAATAAATACGTTAGATTAATTCCTACCAGTGACTAATCTTATGACTTTTTAAACAGATAACTAAAATTACAAACA
AATCGTTTAACTTCTGTATTTATTTACAGATGTAATCACTTCAGGAGTAATTACATGAACAAAAATATAAAATATTCTCAAAAC
TTTTTAACGAGTGAAAAAGTACTCAACCAAATAATAAAACAATTGAATTTAAAAGAAACCGATACCGTTTACGAAATTGGAACA
GGTAAAGGGCATTTAACGACGAAACTGGCTAAAATAAGTAAACAGGTAACGTCTATTGAATTAGACAGTCATCTATTCAACTTA
TCGTCAGAAAAATTAAAACTGAACATTCGTGTCACTTTAATTCACCAAGATATTCTACAGTTTCAATTCCCTAACAAACAGAGG
```

TATAAAATTGTTGGGAGTATTCCTTACCATTTAAGCACACAAATTATTAAAAAAGTGGTTTTTGAAAGCCATGCGTCTGACATC
TATCTGATTGTTGAAGAAGGATTCTACAAGCGTACCTTGGATATTCACCGAACACTAGGGTTGCTCTTGCACACTCAAGTCTCG
ATTCAGCAATTGCTTAAGCTGCCAGCGGAATGCTTTCATCCTAAACCAAAAGTAAACAGTGTCTTAATAAAACTTACCCGCCAT
ACCACAGATGTTCCAGATAAATATTGGAAGCTATATACGTACTTTGTTTCAAAATGGGTCAATCGAGAATATCGTCAACTGTTT
ACTAAAAATCAGTTTCATCAAGCAATGAAACACGCCAAAGTAAACAATTTAAGTACCATTACTTATGAGCAAGTATTGTCTATT
TTTAATAGTTATCTATTATTTAACGGGAGGAAATAATTCTATGAGTCGCTTTTTTAAATTTGGAAAGTTACACGTTACTAAAGG
GAATGGAGATAAATTATTAGATATACTACTGACAGCTTCCAAGAAGCTAAAGAGGTCCCTAGCGCCTACGGGGAATTTGGGGTA
CATTGAAAAAGGAAGAGTATGAGTATTCAACATTTCCGTGTCGCCCTTATTCCCTTTTTTGCGGCATTTTGCCTTCCTGTTTTT
GCTCACCCAGAAACGCTGGTGAAAGTAAAAGATGCTGAAGATCAGTTGGGTGCACGAGTGGGTTACATCGAACTGGATCTCAAC
AGCGGTAAGATCCTTGAGAGTTTTCGCCCCGAAGAACGTTTTCCAATGATGAGCACTTTTAAAGTTCTGCTATGTGGCGCGGTA
TTATCCCGTATTGACGCCGGGCAAGAGCAACTCGGTCGCCGCATACACTATTCTCAGAATGACTTGGTTGAGTACTCACCAGTC
ACAGAAAAGCATCTTACGGATGGCATGACAGTAAGAGAATTATGCAGTGCTGCCATAACCATGAGTGATAACACTGCGGCCAAC
TTACTTCTGACAACGATCGGAGGACCGAAGGAGCTAACCGCTTTTTTGCACAACATGGGGGATCATGTAACTCGCCTTGATCGT
TGGGAACCGGAGCTGAATGAAGCCATACCAAACGACGAGCGTGACACCACGATGCCTGTAGCAATGGCAACAACGTTGCGCAAA
CTATTAACTGGCGAACTACTTACTCTAGCTTCCCGGCAACAATTAATAGACTGGATGGAGGCGGATAAAGTTGCAGGACCACTT
CTGCGCTCGGCCCTTCCGGCTGGCTGGTTTATTGCTGATAAATCTGGAGCCGGTGAGCGTGGGTCTCGCGGTATCATTGCAGCA
CTGGGGCCAGATGGTAAGCCCTCCCGTATCGTAGTTATCTACACGACGGGGAGTCAGGCAACTATGGATGAACGAAATAGACAG
ATCGCTGAGATAGGTGCCTCACTGATTAAGCATTGGTAACTGTCAGACCAAGTTTACTCATATATACTTTAGATTGATTTAAAA
CTTCATTTTTAATTTAAAAGGATCTAGGTGAAGATCCTTTTTGATAATCTCATGACCAAAATCCCTTAACGTGAGTTTTCGTTC
CACTGAGCGTCAGACCCCGTAGAAAAGATCAAAGGATCTTC-3’

pSa-control-GFP

5’-
TTGAGATCCTTTTTTTCTGCGCGTAATCTGCTGCTTGCAAACAAAAAAACCACCGCTACCAGCGGTGGTTTGTTTGCCGGATCA
AGAGCTACCAACTCTTTTTCCGAAGGTAACTGGCTTCAGCAGAGCGCAGATACCAAATACTGTTCTTCTAGTGTAGCCGTAGTT
AGGCCACCACTTCAAGAACTCTGTAGCACCGCCTACATACCTCGCTCTGCTAATCCTGTTACCAGTGGCTGCTGCCAGTGGCGA
TAAGTCGTGTCTTACCGGGTTGGACTCAAGACGATAGTTACCGGATAAGGCGCAGCGGTCGGGCTGAACGGGGGGTTCGTGCAC
ACAGCCCAGCTTGGAGCGAACGACCTACACCGAACTGAGATACCTACAGCGTGAGCTATGAGAAAGCGCCACGCTTCCCGAAGG
GAGAAAGGCGGACAGGTATCCGGTAAGCGGCAGGGTCGGAACAGGAGAGCGCACGAGGGAGCTTCCAGGGGGAAACGCCTGGTA
TCTTTATAGTCCTGTCGGGTTTCGCCACCTCTGACTTGAGCGTCGATTTTTGTGATGCTCGTCAGGGGGGCGGAGCCTATGGAA
AAACGCCAGCAACGCGGCCTTTTTACGGTTCCTGGCCTTTTGCTGGCCTTTTGCTCACATGTTCTTTCCTGCGTTATCCCCTGA
TTCTGTGGATAACCGTATTACCGCCTTTGAGTGAGCTGATACCGCTCGCCGCAGCCGAACGACCGAGCGCAGCGAGTCAGTGAG
CGAGGAAGCGGAAGAGCGCCCAATACGCAAACCGCCTCTCCCCGCGCGTTGGCCGATTCATTAATGCAGCTGGCACGACAGGTT
TCCCGACTGGAAAGCGGGCAGTGAGCGCAACGCAATTAATGTGAGTTAGCTCACTCATTAGGCACCCCAGGCTTTACACTTTAT
GCTTCCGGCTCGTATGTTGTGTGGAATTGTGAGCGGATAACAATTTCACACAGGAAACAGCTATGGCCATGATTACGCCAAGCTT
GCATGCCTGCACAGATGCATTTTATTTCATATAGTAAGTACATCACCTATTAGTTTGTTGTTTAAACAAACTAACTTATTTTCA
TCTTATATAACCTCGTCAGTATTTTCAATATTTTTTTAGTTTTTTATGAACACATTAGATATAATAAAGGGAAGATTCGCTAT
GTACTATGTTGATACTTAATTTAAAGATTAAACAAATGGAGTGGATGAAGTGGATATCGCTGATCAAACCTTTGTCAAAAAAGT
AAATCAAAAGTTATTATTAAAAGAAATCCTTAAAAATTCACCTATTTCAAGAGCAAAATTATCTGAAATGACTGGATTAAATAA
ATCAACTGTCTCATCACAGGTAAACACGTTAATGAAAGAAAGTATGGTATTTGAAATAGGTCAAGGACAATCAAGTGGCGGAAG
AAGACCTGTCATGCTTGTTTTTAATAAAAAGGCAGGATACTCCGTTGGAATAGATGTTGGTGTGGATTATATTAATGGCATTTT
AACAGACCTTGAAGGAACAATCGTTCTTGATCAATACCGCCATTTGGAATCCAATTCTCCAGAAATAACGAAAGACATTTTGAT
TGATATGATTCATCACTTTATTACGCAAATGCCCCAATCTCCGTACGGGCTTATTGGTATAGGTATTTGCGTGCCTGGACTCAT
TGATAAAGATCAAAAAATTGTTTTCACTCCGAACTCCAACTGGAGAGATATTGACTTAAAATCTTCGATACAAGAGAAGTACAA
TGTGCCTGTTTTTATTGAAAATGAGGCAAATGCTGGCGCATATGGAGAAAAAGTATTTGGAGCTGCAAAAAATCACGATAACAT
TATTTACGTAAGTATCAGCACAGGAATAGGGATCGGTGTTATTATCAACAATCATTTATATAGAGGAGTAAGCGGCTTCTCTGG
AGAAATGGGACATATGACAATAGACTTTAATGGTCCTAAATGCAGTTGCGGAAACCGAGGATGCTGGGAATTGTATGCTTCAGA
GAAGGCTTTATTAAAATCTCTTCAGACCAAAGAGAAAAAACTGTCCTATCAAGATATCATAAACCTCGCCCATCTGAATGATAT
CGGAACCTTAAATGCATTACAAAATTTTGGATTCTATTTAGGAATAGGCCTTACCAATATTCTAAATACTTTCAACCCACAAGC
CGTAATTTTAAGAAATAGCATAATTGAATCGCATCCTATGGTTTTAAATTCAATGAGAAGTGAAGTATCATCAAGGGTTTATTC
CCAATTAGGCAATAGCTATGAATTATTGCCATCTTCCTTAGGACAGAATGCACCGGCATTAGGAATGTCCTCCATTGTGATTGA
TCATTTTCTGGACATGATTACAATGTAATTTTTTATGGAATGGACAGCTCATCTTTAAAGATGAGTTTTTTTATTCTAGGAGTA
TTTCTGAAGCAATAGTGACATGGCACCTTCTCATATGAAAAAGGAGTTCTAAAATAGAAATCTCCTTTTTCATGTGCAAATTAT
TTTTCTTTATAACGAAAATATCTAAAGTCGGCCAATTCACTGGCCGTCGTTTTACCGAAATACGGGCAGACATGGCCTGCCCGG
TTAATGCCTGGCTTCACTGTCATTATACGATTTAGTACAATCTTTTATCATTATATTGCCTAACTGTAGGAAATAAATACTTAA
CTGTTAAATGTAATTTGTATTTAATATTTTAACATAAAAAAATTTACAGTTAAGAATAAAAAACGACTAGTTAAGAAAAATTGG
AAAATAAATGCTTTTAGCATGTTTTAATATAACTAGATCACAGAGATGTGGTCAGTCAGTCTTAATTAACCAAGGAGGTGAAAT
GTACAATGAAAGGAGAAGAACTTTTCACTGGAGTTGTCCCAATTCTTGTTGAATTAGATGGTGATGTTAATGGGCACAAATTTT
CTGTCAGTGGAGAGGGTGAAGGTGATGCAACATACGGAAAACTTACCCTTAAATTTATTTGCACTACTGGAAAACTACCTGTTC
CATGGCCAACACTTGTCACTACTTTCGCGTATGGTCTTCAATGCTTTGCGAGATACCCAGATCATATGAAACAGCATGACTTTT
TCAAGAGTGCCATGCCCGAAGGTTATGTACAGGAAAGAACTATATTTTTCAAAGATGACGGGAACTACAAGACACGTGCTGAAG
TCAAGTTTGAAGGTGATACCCTTGTTAATAGAATCGAGTTAAAAGGTATTGATTTTAAAGAAGATGGAAACATTCTTGGACACA

```
AATTGGAATACAACTATAACTCACACAATGTATACATCATGGCAGACAAACAAAAGAATGGAATCAAAGTTAACTTCAAAATTA
GACACAACATTGAAGATGGAAGCGTTCAACTAGCAGACCATTATCAACAAAATACTCCAATTGGCGATGGCCCTGTCCTTTTAC
CAGACAACCATTACCTGTCCACACAATCTAAGCTTTCGAAAGATCCCAACGAAAAGAGAGACCACATGGTCCTTCTTGAGTTTG
TAACAGCTGCTGGGATTACACATGGCATGGATGAACTATACAAATAACCTAGGCTGCTGCCACCGCTGAGCAATAACTAGCATA
ACCCCTTGGGGCCTCTAAACGGGTCTTGAGGGGTTTTTTGAACGTCGTGACTGGGAAAACCCTGGCGTTACCCAACTTAATCGC
CTTGCAGCACATCCCCCTTTCGCCAGCTGGCGTAATAGCGAAGAGGCCCGCACCGATCGCCCTTCCCAACAGTTGCGCAGCCTG
AATGGCGAATGGCGCCTGATGCGGTATTTTCTCCTTACGCATCTGTGCGGTATTTCACACCGCATATGGTGCACTCTCAGTACA
ATCTGCTCTGATGCCGCATAGTTAAGCCAGCCCCGACACCCGCCAACACCCGCTGACGCGCCCTGACGGGCTTGTCTGCTCCCG
GCATCCGCTTACAGACAAGCTGTGACCGTCTCCGGGAGCTGCATGTGTCAGAGGTTTTCACCGTCATCACCGAAACGCGCGAGA
CGAAAGGGCCTCGTGATACGCCTATTTTTATAGGTTAATGTCATGATAATAATGGTTTCTTAGACGTCAGGTGGCACTTTTCGG
GGAAATGTGCGCGGAACCCCTATTTGTTTATTTTTCTAAATACATTCAAATATGTATCCGCTCATGAGACAATAACCCTGATAA
ATGCTTCAATAATCCATCCTCCAAAGTTGGAGAGTGAGTTTTATGTCGCAAATATTAATGTTTCTGGTGAACCTTATCAAATTT
TCGTTGATTTAATAGAAACATAGCGGTAAAATTAGCAGTAACTTAATAGAACGGAAATGAAAAAAGCCACTCTCATATGCTATT
GGCTACCAACCTTTAGCGAGAATGACTTAATCCTGTACAGCCATACAGGACTTCGACTTATAAGAGGCGCCAACTTCAAATAAG
TTATTTGCCTTGTTTTCGCGAACAAGGCTTATTAGATACACCTATTGTACCGTTACTCTACGAATATTTCAAGTAGTAATTACT
AGCATTGTCCGTTACTCTACGAATATTTCAAGTAGTAATTACTAGCATTGTCCGTTACTCTACGAATATTTCAAGTAGTAATTA
CTAGCATTGTCATATACATAATAAAACGGATATAAAAGGGCGTTTTCTATACCTAGAAGTCTTGTAAATGTACAGGGCGTTTAG
ATATAGAGAACGCCCTTTTTGTGTTCCGTTCCAGTGGAAGCTACCACTTTAAAAAGATGGTCTAGTGTAGCCAATGCAGGAGAG
TACACTCGGATATCAGTTGTCGTTGCATTCAACTGTCTGACGTAAGCGAGGTAAAGGACACAAGCCTTGCATAAAACAAGCCTA
CGGGATGTAAATCCTAATAATGATGATAACCAAGACGTTAGCGGCAAAAAGTGTTGGGGGTTCAAAATAAGACATGATTGTGCG
ACTGGAGTTAAACAGTTACTCGTAAGCGGCGATCATGACACTGATTCACGGCTATTCTTGTACAAGCTAGCTTTATTACAAGGA
TATGCGGGTTATATAGCGAATCACCCGAAAGGGAACGGTGTTGGGCGTGAGAAACGCACCGTACGGCGCAATACAATGCCAATA
AGCTATATACGGACGGTATAGTAGTTTTGTAAGCTATAACCGTTTGTCGTCAATGCAACCAATCTCAATTCGAGACCTCGGCAT
CTAAGCCAGTACGAATGAGTGGGCGTTTTAACCTCGTAAATTTTCAACAGGGGTTACTATGCCCAAAACTACATTCAGATTTCC
TAACAAACTCGCCAGTATGAAAACCTTAAGACCTTAAAGTCAAGGGATTTGAAGGATTTTAACCTCGATTAGCAAAAAATGTAG
AGTACTGAAGCAACTACCATTAACTAAGATAGTGGGGGATTGAGGAAGAATCCAGAGCTGTTTAAATCAAGTGAAAGACAAGAT
GAAATTAAAAGAATAGTGAAAGATAGGGGAGTGGTTCTCTATGAGAAAGGAAATGGCTAGAGAACAAAGGCAGCGGTTTATTGA
TCTATTGTTAGACTTTATGGTAAAGAATCCTCATTTATTTGTTAATGGTACAGAGGATGAAAGTAATAATGTTGTTACAAAATG
TAATAGTGATATTAAAGAGGTTGCGGAGTCATATTTAACTCTTTTATAGTGAGAGGGTTAAAACTAATTAATATGTATTAAGGC
CCAATGTTGGAATTATTGTATTTCACTAGGCAACCTACTTACTAAAAGTAAGATTATCCATTAGTGGATGTTATAATATTGGGT
TTTTTAACACAATAATCATCGCCTTTCGGTGTCGTTTGATAGAAAAGTAACCATTAGCGATGAAAAAGTCAATATAAAAAGCCA
TCCGTAAAAAACGGATGGCTTACCGTACATAGGATCGTTGGTAGGGCGGCGTATCCTACATCTCTGGTAACTTACCTAGCCAAT
CAAATGCTTGAGAACGGCGGTTAGATAAGCGCGTGGGGAACCTTTCCCACCTCAAAGATCCTATATCATTATTATGTTACTTTC
TACAGGTAGTATACCATGTTCTTATATTTTAGTAAACTCCCCGTTAGCTTAACAGGTCTTTGTAAGCAATTAAACGTCCACTAT
TCAATCGTCTTTGGATTTTCGCAGGACCGTTTTTTAGATCGAACATAGTTGATAAGAACAAATAACCGCTTGGGTCCAACTTTA
TAGCAATTAGTATATGGTCATTTAAAATCTTTACCAATTCAACGCTATTAGGTTCTTTAGGATTTTGCCCGACATAGTCGGGGT
GTTCAACGATATCTTTTATGTGCGATGAATATTTTTCATAAATACCAGGATGTTGTTTCTTTACGTGCTTTATAAATCCGGGAA
ACATTTTTACATCGTTAGAAGTGCAAGTCAAGTTATATGTATCTATAATGATTTGTGGAAGTTTTGCCACAACAGTTGGTTTAT
TTACAATCTTTTTTTTTATTAGCCGTCAAATTTCTCCCTCATCTCGTCTCTTTATATCTTTATTTTATCATAAAGGAGTATTTGA
ACCGTCGCGCGGGACAGGTTTATGATAGGGATATTTTATTGAATAATTGATGGTATAAGGGACTTTCATGCTTGGAAAGTGGGG
ATTATGAATTAGATGCTTGTCCACAATATGTTCCAATGTAATTAAAATTTATGTTCCCACCTTGACCAAACATCACGTCCATAC
TTAAATCGTCCCTCCTTTAATAGGTAAAATATTAATTTACCTTAATAAAAAAATAATGGATAATAGTATTCGTCTGAATTTATA
TAATCAGGGGGAACTATTGATGCTGGGGATACTATTTACAGCGGCGCCATCTACTGATGTCGTAAAGGATTTGCAAGATAAAGT
TATATCATTGCAGGATCATGAGGTAGCGTTTTTGAACACCACGATATCTAATATGTTGATCCCCGAAGCAAACTTAAGAGTGTG
TTGATAGTGCAGTATCTTAAAATTTTGTGTATAATAGGAATTGAAGTTAAATTAGATGCTAAAAATTTGTAATTAAGAAGGAGG
GATTCGTCATGTTGGTATTCCAAATGCGTAATGTAGATAAAACATCTACTGTTTTGAAACAGACTAAAAACAGTGATTACGCAG
ATAAATAAATACGTTAGATTAATTCCTACCAGTGACTAATCTTATGACTTTTTAAACAGATAACTAAAATTACAAACAAATCGT
TTAACTTCTGTATTTATTTACAGATGTAATCACTTCAGGAGTAATTACATGAACAAAAATATAAAATATTCTCAAAACTTTTTA
ACGAGTGAAAAAGTACTCAACCAAATAATAAAACAATTGAATTTAAAAGAAACCGATACCGTTTACGAAATTGGAACAGGTAAA
GGGCATTTAACGACGAAACTGGCTAAAATAAGTAAACAGGTAACGTCTATTGAATTAGACAGTCATCTATTCAACTTATCGTCA
GAAAAATTAAAACTGAACATTCGTGTCACTTTAATTCACCAAGATATTCTACAGTTTCAATTCCCTAACAAACAGAGGTATAAA
ATTGTTGGGAGTATTCCTTACCATTTAAGCACACAAATTATTAAAAAAGTGGTTTTTGAAAGCCATGCGTCTGACATCTATCTG
ATTGTTGAAGAAGGATTCTACAAGCGTACCTTGGATATTCACCGAACACTAGGGTTGCTCTTGCACACTCAAGTCTCGATTCAG
CAATTGCTTAAGCTGCCAGCGGAATGCTTTCATCCTAAACCAAAAGTAAACAGTGTCTTAATAAAACTTACCCGCCATACCACA
GATGTTCCAGATAAATATTGGAAGCTATATACGTACTTTGTTTCAAAATGGGTCAATCGAGAATATCGTCAACTGTTTACTAAA
AATCAGTTTCATCAAGCAATGAAACACGCCAAAGTAAACAATTTAAGTACCATTACTTATGAGCAAGTATTGTCTATTTTTAAT
AGTTATCTATTATTTAACGGGAGGAAATAATTCTATGAGTCGCTTTTTTAAATTTGGAAAGTTACACGTTACTAAAGGGAATGG
AGATAAATTATTAGATATACTACTGACAGCTTCCAAGAAGCTAAAGAGGTCCCTAGCGCCTACGGGGAATTTGGGGTACATTGA
AAAAGGAAGAGTATGAGTATTCAACATTTCCGTGTCGCCCTTATTCCCTTTTTTGCGGCATTTTGCCTTCCTGTTTTTGCTCAC
CCAGAAACGCTGGTGAAAGTAAAAGATGCTGAAGATCAGTTGGGTGCACGAGTGGGTTACATCGAACTGGATCTCAACAGCGGT
AAGATCCTTGAGAGTTTTCGCCCCGAAGAACGTTTTCCAATGATGAGCACTTTTAAAGTTCTGCTATGTGGCGCGGTATTATCC
CGTATTGACGCCGGGCAAGAGCAACTCGGTCGCCGCATACACTATTCTCAGAATGACTTGGTTGAGTACTCACCAGTCACAGAA
AAGCATCTTACGGATGGCATGACAGTAAGAGAATTATGCAGTGCTGCCATAACCATGAGTGATAACACTGCGGCCAACTTACTT
```

CTGACAACGATCGGAGGACCGAAGGAGCTAACCGCTTTTTTGCACAACATGGGGGATCATGTAACTCGCCTTGATCGTTGGGAA
CCGGAGCTGAATGAAGCCATACCAAACGACGAGCGTGACACCACGATGCCTGTAGCAATGGCAACAACGTTGCGCAAACTATTA
ACTGGCGAACTACTTACTCTAGCTTCCCGGCAACAATTAATAGACTGGATGGAGGCGGATAAAGTTGCAGGACCACTTCTGCGC
TCGGCCCTTCCGGCTGGCTGGTTTATTGCTGATAAATCTGGAGCCGGTGAGCGTGGGTCTCGCGGTATCATTGCAGCACTGGGG
CCAGATGGTAAGCCCTCCCGTATCGTAGTTATCTACACGACGGGGAGTCAGGCAACTATGGATGAACGAAATAGACAGATCGCT
GAGATAGGTGCCTCACTGATTAAGCATTGGTAACTGTCAGACCAAGTTTACTCATATATACTTTAGATTGATTTAAAACTTCAT
TTTTAATTTAAAAGGATCTAGGTGAAGATCCTTTTTGATAATCTCATGACCAAAATCCCTTAACGTGAGTTTTCGTTCCACTGA
GCGTCAGACCCCGTAGAAAAGATCAAAGGATCTTC-3′

pXyl-Sa-agrC

5′-
TTGAGATCCTTTTTTTCTGCGCGTAATCTGCTGCTTGCAAACAAAAAAACCACCGCTACCAGCGGTGGTTTGTTTGCCGGATCA
AGAGCTACCAACTCTTTTTCCGAAGGTAACTGGCTTCAGCAGAGCGCAGATACCAAATACTGTTCTTCTAGTGTAGCCGTAGTT
AGGCCACCACTTCAAGAACTCTGTAGCACCGCCTACATACCTCGCTCTGCTAATCCTGTTACCAGTGGCTGCTGCCAGTGGCGA
TAAGTCGTGTCTTACCGGGTTGGACTCAAGACGATAGTTACCGGATAAGGCGCAGCGGTCGGGCTGAACGGGGGGTTCGTGCAC
ACAGCCCAGCTTGGAGCGAACGACCTACACCGAACTGAGATACCTACAGCGTGAGCTATGAGAAAGCGCCACGCTTCCCGAAGG
GAGAAAGGCGGACAGGTATCCGGTAAGCGGCAGGGTCGGAACAGGAGAGCGCACGAGGGAGCTTCCAGGGGGAAACGCCTGGTA
TCTTTATAGTCCTGTCGGGTTTCGCCACCTCTGACTTGAGCGTCGATTTTTGTGATGCTCGTCAGGGGGGCGGAGCCTATGGAA
AAACGCCAGCAACGCGGCCTTTTTACGGTTCCTGGCCTTTTGCTGGCCTTTTGCTCACATGTTCTTTCCTGCGTTATCCCCTGA
TTCTGTGGATAACCGTATTACCGCCTTTGAGTGAGCTGATACCGCTCGCCGCAGCCGAACGACCGAGCGCAGCGAGTCAGTGAG
CGAGGAAGCGGAAGAGCGCCCAATACGCAAACCGCCTCTCCCCGCGCGTTGGCCGATTCATTAATGCAGCTGGCACGACAGGTT
TCCCGACTGGAAAGCGGGCAGTGAGCGCAACGCAATTAATGTGAGTTAGCTCACTCATTAGGCACCCCAGGCTTTACACTTTAT
GCTTCCGGCTCGTATGTTGTGTGAATTGTGAGCGGATAACAATTTCACACAGGAAACAGCTATGGCCATGATTACGCCAAGCTT
GCATGCCTGCACTAGTTGTTAATAATTTCAACTTTTTGAATAAAGAAACCATTTTCGATAATTGTATCTAATAAGACATTGTCT
GCATTATCAGCAATTTCTTTTAAAGTTGATAGACCTAAACCACGACCTTCACCTTTAGTAGAAAAACTTTCTTGGAACAATTCA
TGAATGCGTGGTATATCATCAGCGCATTTATTCATAACAATAAACGTTACTGAATTTTCACTTTCAATAAATGCAACGCGAATG
ATAGGGTCATCAATTTCAGTTGATGCCTCAATTGCATTATCAAGAATAATACCAATACTGCGACTTAAATCGATCATATTCAAG
TTAATGCTACTTACTTCATCGGGTATTTCGATACTAATCGGAATATTCATTTCTTGTGCACGTAAAATTTTCGCAGTAATTAAG
CCTTTAATTTCACGTACTTTAAGATTCTCGATACCATTTAATTTTATAGCATTCATTTGTAAATTGTCTTTCATAGGTACAATA
TTTTTATTGAAATAATCACGTAGGCCAGGCATGTCATCTTCTCGAATGTATTCTGAAAGTGTCGTTAAGATATTGACATAATCA
TGACGGAACTTGCGCATTTCGTTGTTGATAGCTTCAATCTTCAATGTATATTCATAATAGGTTTCAATTTCTTCTTGATTACGT
TTATATTTCATCTCTTTAAGGAGAAATTGAGAAATAACAAATGTTAATATACTTAAAAATATAGTGATACCAATAAAAATAAAA
GAATACTGCCTTATTACTTTAGCTTCATCCGAGTTTATTTGTGAATAAAAGAAAAATAATGAAAAAGTAAGCAGTAAGATAGTC
GAAATAACTATTAAAAATCCTTTGTTTAGTATTAGATATGGTGTGCTAATTTTTTTGAGAACTCTATTTATTATATATGAGAAT
AGTATACTAATAGTCACATAAACTACAAAAAAGCTAGGGAATATTACAAATATACTATCAGAAATTTTGGTGATATATGCATAT
ATAACTATATACATGTAGTTAGCACAGTATAGAATAATCTGCGAAGTCATAATCAACAAAATAGAATACCATTTGATTTTGACA
AAATACATTATAATAATAAATGAAGTTAATATGATTAAGGACGCGCTATCAAACATTTTAAATAGAAATAACGATAATGTCGAA
ATTACTATGATGAAAAAATAATCAAGTTTACTGTACTTAATACCACTAATTATAGCTGGTATTGTAAACATTAATATCATTTGA
GTTAATACGAATAAAACAAAATTATAACTATTTAATAATTCCACAGTAGTTCCTCCTTATCTACAGATGCATTTTATTTCATAT
AGTAAGTACATCACCTATTAGTTTGTTGTTTAAACAAACTAACTTATTTTCATCTTATATAACCTCGTCAGTATTTTCAATATT
TTTTTTAGTTTTTTATGAACACATTAGATATAATAAAGGGAAGATTCGCTATGTACTATGTTGATACTTAATTTAAAGATTAAA
CAAATGGAGTGGATGAAGTGGATATCGCTGATCAAACCTTTGTCAAAAAAGTAAATCAAAAGTTATTATTAAAAGAAATCCTTA
AAAATTCACCTATTTCAAGAGCAAAATTATCTGAAATGACTGGATTAAATAAATCAACTGTCTCATCACAGGTAAACACGTTAA
TGAAAGAAAGTATGGTATTTGAAATAGGTCAAGGACAATCAAGTGGCGGAAGAAGACCTGTCATGCTTGTTTTTAATAAAAAGG
CAGGATACTCCGTTGGAATAGATGTTGGTGTGGATTATATTAATGGCATTTTAACAGACCTTGAAGGAACAATCGTTCTTGATC
AATACCGCCATTTGGAATCCAATTCTCCAGAAATAACGAAAGACATTTTGATTGATATGATTCATCACTTTATTACGCAAATGC
CCCAATCTCCGTACGGGCTTATTGGTATAGGTATTTGCGTGCCTGGACTCATTGATAAAGATCAAAAAATTGTTTTCACTCCGA
ACTCCAACTGGAGAGATATTGACTTAAAATCTTCGATACAAGAGAAGTACAATGTGCCTGTTTTTATTGAAAATGAGGCAAATG
CTGGCGCATATGGAGAAAAAGTATTTGGAGCTGCAAAAAATCACGATAACATTATTTACGTAAGTATCAGCACAGGAATAGGGA
TCGGTGTTATTATCAACAATCATTTATATAGAGGAGTAAGCGGCTTCTCTGGAGAAATGGGACATATGACAATAGACTTTAATG
GTCCTAAATGCAGTTGCGGAAACCGAGGATGCTGGGAATTGTATGCTTCAGAGAAGGCTTTATTAAAATCTCTTCAGACCAAAG
AGAAAAAACTGTCCTATCAAGATATCATAAACCTCGCCCATCTGAATGATATCGGAACCTTAAATGCATTACAAAATTTTGGAT
TCTATTTAGGAATAGGCCTTACCAATATTCTAAATACTTTCAACCCACAAGCCGTAATTTTAAGAAATAGCATAATTGAATCGC
ATCCTATGGTTTTAAATTCAATGAGAAGTGAAGTATCATCAAGGGTTTATTCCCAATTAGGCAATAGCTATGAATTATTGCCAT
CTTCCTTAGGACAGAATGCACCGGCATTAGGAATGTCCTCCATTGTGATTGATCATTTTCTGGACATGATTACAATGTAATTTT
TTATGGAATGGACAGCTCATCTTTAAAGATGAGTTTTTTTATTCTAGGAGTATTTCTGAAGCAATAGTGACATGGCACCTTCTC
ATATGAAAAAGGAGTTCTAAAATAGAAATCTCCTTTTTCATGTGCAAATTATTTTTCTTTATAACGAAAATATCTAAAGTCGGC
CAATTCACTGGCCGTCGTTTTACCGAAATACGGGCAGACATGGCCTGCCCGGTTAATGCCTGGCTTCACTGTCATTATACGATT
TAGTACAATCTTTTATCATTATATTGCCTAACTGTAGGAAATAAATACTTAACTGTTAAATGTAATTTGTATTTAATATTTTAA
CATAAAAAAATTTACAGTTAAGAATAAAAAACGACTAGTTAAGAAAAATTGGAAAATAAATGCTTTTAGCATGTTTTAATATAA
CTAGATCACAGAGATGTGGTCAGTCAGTCTTAATTAACCAAGGAGGTGAAATGTACAATGAAAGGAGAAGAACTTTTCACTGGA
GTTGTCCCAATTCTTGTTGAATTAGATGGTGATGTTAATGGGCACAAATTTTCTGTCAGTGGAGAGGGTGAAGGTGATGCAACA

```
TACGGAAAACTTACCCTTAAATTTATTTGCACTACTGGAAAACTACCTGTTCCATGGCCAACACTTGTCACTACTTTCGCGTAT
GGTCTTCAATGCTTTGCGAGATACCCAGATCATATGAAACAGCATGACTTTTTCAAGAGTGCCATGCCCGAAGGTTATGTACAG
GAAAGAACTATATTTTTCAAAGATGACGGGAACTACAAGACACGTGCTGAAGTCAAGTTTGAAGGTGATACCCTTGTTAATAGA
ATCGAGTTAAAAGGTATTGATTTTAAAGAAGATGGAAACATTCTTGGACACAAATTGGAATACAACTATAACTCACACAATGTA
TACATCATGGCAGACAAACAAAAGAATGGAATCAAAGTTAACTTCAAAATTAGACACAACATTGAAGATGGAAGCGTTCAACTA
GCAGACCATTATCAACAAAATACTCCAATTGGCGATGGCCCTGTCCTTTTACCAGACAACCATTACCTGTCCACACAATCTAAG
CTTTCGAAAGATCCCAACGAAAAGAGAGACCACATGGTCCTTCTTGAGTTTGTAACAGCTGCTGGGATTACACATGGCATGGAT
GAACTATACAAATAACCTAGGCTGCTGCCACCGCTGAGCAATAACTAGCATAACCCCTTGGGGCCTCTAAACGGGTCTTGAGGG
GTTTTTTGAACGTCGTGACTGGGAAAACCCTGGCGTTACCCAACTTAATCGCCTTGCAGCACATCCCCCTTTCGCCAGCTGGCG
TAATAGCGAAGAGGCCCGCACCGATCGCCCTTCCCAACAGTTGCGCAGCCTGAATGGCGAATGGCGCCTGATGCGGTATTTTCT
CCTTACGCATCTGTGCGGTATTTCACACCGCATATGGTGCACTCTCAGTACAATCTGCTCTGATGCCGCATAGTTAAGCCAGCC
CCGACACCCGCCAACACCCGCTGACGCGCCCTGACGGGCTTGTCTGCTCCCGGCATCCGCTTACAGACAAGCTGTGACCGTCTC
CGGGAGCTGCATGTGTCAGAGGTTTTCACCGTCATCACCGAAACGCGCGAGACGAAAGGGCCTCGTGATACGCCTATTTTTATA
GGTTAATGTCATGATAATAATGGTTTCTTAGACGTCAGGTGGCACTTTTCGGGGAAATGTGCGCGGAACCCCTATTTGTTTATT
TTTCTAAATACATTCAAATATGTATCCGCTCATGAGACAATAACCCTGATAAATGCTTCAATAATCCATCCTCCAAAGTTGGAG
AGTGAGTTTTATGTCGCAAATATTAATGTTTCTGGTGAACCTTATCAAATTTTCGTTGATTTAATAGAAACATAGCGGTAAAAT
TAGCAGTAACTTAATAGAACGGAAATGAAAAAAGCCACTCTCATATGCTATTGGCTACCAACCTTTAGCGAGAATGACTTAATC
CTGTACAGCCATACAGGACTTCGACTTATAAGAGGCGCCAACTTCAAATAAGTTATTTGCCTTGTTTTCGCGAACAAGGCTTAT
TAGATACACCTATTGTACCGTTACTCTACGAATATTTCAAGTAGTAATTACTAGCATTGTCCGTTACTCTACGAATATTTCAAG
TAGTAATTACTAGCATTGTCCGTTACTCTACGAATATTTCAAGTAGTAATTACTAGCATTGTCATATACATAATAAAACGGATA
TAAAAGGGCGTTTTCTATACCTAGAAGTCTTGTAAATGTACAGGGCGTTTAGATATAGAGAACGCCCTTTTTGTGTTCCGTTCC
AGTGGAAGCTACCACTTTAAAAAGATGGTCTAGTGTAGCCAATGCAGGAGAGTACACTCGGATATCAGTTGTCGTTGCATTCAA
CTGTCTGACGTAAGCGAGGTAAAGGACACAAGCCTTGCATAAAACAAGCCTACGGGATGTAAATCCTAATAATGATGATAACCA
AGACGTTAGCGGCAAAAAGTGTTGGGGGTTCAAAATAAGACATGATTGTGCGACTGGAGTTAAACAGTTACTCGTAAGCGGCGA
TCATGACACTGATTCACGGCTATTCTTGTACAAGCTAGCTTTATTACAAGGATATGCGGGTTATATAGCGAATCACCCGAAAGG
GAACGGTGTTGGGCGTGAGAAACGCACCGTACGGCGCAATACAATGCCAATAAGCTATATACGGACGGTATAGTAGTTTTGTAA
GCTATAACCGTTTGTCGTCAATGCAACCAATCTCAATTCGAGACCTCGGCATCTAAGCCAGTACGAATGAGTGGGCGTTTTAAC
CTCGTAAATTTTCAACAGGGGTTACTATGCCCAAAACTACATTCAGATTTCCTAACAAACTCGCCAGTACGAAAACCTTAAGAC
CTTAAAGTCAAGGGATTTGAAGGATTTTAACCTCGATTAGCAAAAAATGTAGAGTACTGAAGCAACTACCATTAACTAAGATAG
TGGGGGATTGAGGAAGAATCCAGAGCTGTTTAAATCAAGTGAAAGACAAGATGAAATTAAAAGAATAGTGAAAGATAGGGGAGT
GGTTCTCTATGAGAAAGGAAATGGCTAGAGAACAAAGGCAGCGGTTTATTGATCTATTGTTAGACTTTATGGTAAAGAATCCTC
ATTTATTTGTTAATGGTACAGAGGATGAAAGTAATAATGTTGTTACAAAATGTAATAGTGATATTAAAGAGGTTGCGGAGTCAT
ATTTAACTCTTTTATAGTGAGAGGGTTAAAACTAATTAATATGTATTAAGGCCCAATGTTGGAATTATTGTATTTCACTAGGCA
ACCTACTTACTAAAAGTAAGATTATCCATTAGTGGATGTTATAATATTGGGTTTTTTAACACAATAATCATCGCCTTTCGGTGT
CGTTTGATAGAAAAGTAACCATTAGCGATGAAAAAGTCAATATAAAAAGCCATCCATAAAAAAACGGATGGCTTACCGTACATAG
GATCGTTGGTAGGGCGGCGTATCCTACATCTCTGGTAACTTACCTAGCCAATCAAATGCTTGAGAACGGCGGTTAGATAAGCGC
GTGGGGAACCTTTCCCACCTCAAAGATCCTATATCATTATTATGTTACTTTCTACAGGTAGTATACCATGTTCTTATATTTTAG
TAAACTCCCCGTTAGCTTAACAGGTCTTTGTAAGCAATTAAACGTCCACTATTCAATCGTCTTTGGATTTTCGCAGGACCGTTT
TTTAGATCGAACATAGTTGATAAGAACAAATAACCGCTTGGGTCCAACTTTATAGCAATTAGTATATGGTCATTTAAAATCTTT
ACCAATTCAACGCTATTAGGTTCTTTAGGATTTTGCCCGACATAGTCGGGGTGTTCAACGATATCTTTTATGTGCGATGAATAT
TTTTCATAAATACCAGGATGTTGTTTCTTTACGTGCTTTATAAATCCGGGAAACATTTTTACATCGTTAGAAGTGCAAGTCAAG
TTATATGTATCTATAATGATTTGTGGAAGTTTTGCCACAACAGTTGGTTTATTTACAATCTTTTTTTTATTAGCCGTCAAATTT
CTCCCTCATCTCGTCTCTTTATATCTTTATTTTATCATAAAGGAGTATTTGAACCGTCGCGCGGGACAGGTTTATGATAGGGAT
ATTTTATTGAATAATTGATGGTATAAGGGACTTTCATGCTTGGAAAGTGGGGATTATGAATTAGATGCTTGTCCACAATATGTT
CCAATGTAATTAAAATTTATGTTCCCACCTTGACCAAACATCACGTCCATACTTAAATCGTCCCTCCTTTAATAGGTAAAATAT
TAATTTACCTTAATAAAAAAATAATGGATAATAGTATTCGTCTGAATTTATATAATCAGGGGGAACTATTGATGCTGGGGATAC
TATTTACAGCGGCGCCATCTACTGATGTCGTAAAGGATTTGCAAGATAAAGTTATATCATTGCAGGATCATGAGGTAGCGTTTT
TGAACACCACGATATCTAATATGTTGATCCCCGAAGCAAACTTAAGAGTGTGTTGATAGTGCAGTATCTTAAAATTTTGTGTAT
AATAGGAATTGAAGTTAAATTAGATGCTAAAAATTTGTAATTAAGAAGGAGGGATTCGTCATGTTGGTATTCCAAATGCGTAAT
GTAGATAAAACATCTACTGTTTTGAAACAGACTAAAAACAGTGATTACGCAGATAAATAAATACGTTAGATTAATTCCTACCAG
TGACTAATCTTATGACTTTTTAAACAGATAACTAAAATTACAAACAAATCGTTTAACTTCTGTATTTATTTACAGATGTAATCA
CTTCAGGAGTAATTACATGAACAAAAATATAAAATATTCTCAAAACTTTTTAACGAGTGAAAAAGTACTCAACCAAATAATAAA
ACAATTGAATTTAAAAGAAACCGATACCGTTTACGAAATTGGAACAGGTAAAGGGCATTTAACGACGAAACTGGCTAAAATAAG
TAAACAGGTAACGTCTATTGAATTAGACAGTCATCTATTCAACTTATCGTCAGAAAAATTAAAACTGAACATTCGTGTCACTTT
AATTCACCAAGATATTCTACAGTTTCAATTCCCTAACAAACAGAGGTATAAAATTGTTGGGAGTATTCCTTACCATTTAAGCAC
ACAAATTATTAAAAAAGTGGTTTTTGAAAGCCATGCGTCTGACATCTATCTGATTGTTGAAGAAGGATTCTACAAGCGTACCTT
GGATATTCACCGAACACTAGGGTTGCTCTTGCACACTCAAGTCTCGATTCAGCAATTGCTTAAGCTGCCAGCGGAATGCTTTCA
TCCTAAACCAAAAGTAAACAGTGTCTTAATAAAACTTACCCGCCATACCACAGATGTTCCAGATAAATATTGGAAGCTATATAC
GTACTTTGTTTCAAAATGGGTCAATCGAGAATATCGTCAACTGTTTACTAAAAATCAGTTTCATCAAGCAATGAAACACGCCAA
AGTAAACAATTTAAGTACCATTACTTATGAGCAAGTATTGTCTATTTTTAATAGTTATCTATTATTTAACGGGAGGAAATAATT
CTATGAGTCGCTTTTTTAAATTTGGAAAGTTACACGTTACTAAAGGGAATGGAGATAAATTATTAGATATACTACTGACAGCTT
CCAAGAAGCTAAAGAGGTCCCTAGCGCCTACGGGGAATTTGGGGTACATTGAAAAAGGAAGAGTATGAGTATTCAACATTTCCG
TGTCGCCCTTATTCCCTTTTTTGCGGCATTTTGCCTTCCTGTTTTTGCTCACCCAGAAACGCTGGTGAAAGTAAAAGATGCTGA
```

```
AGATCAGTTGGGTGCACGAGTGGGTTACATCGAACTGGATCTCAACAGCGGTAAGATCCTTGAGAGTTTTCGCCCCGAAGAACG
TTTTCCAATGATGAGCACTTTTAAAGTTCTGCTATGTGGCGCGGTATTATCCCGTATTGACGCCGGGCAAGAGCAACTCGGTCG
CCGCATACACTATTCTCAGAATGACTTGGTTGAGTACTCACCAGTCACAGAAAAGCATCTTACGGATGGCATGACAGTAAGAGA
ATTATGCAGTGCTGCCATAACCATGAGTGATAACACTGCGGCCAACTTACTTCTGACAACGATCGGAGGACCGAAGGAGCTAAC
CGCTTTTTTGCACAACATGGGGGATCATGTAACTCGCCTTGATCGTTGGGAACCGGAGCTGAATGAAGCCATACCAAACGACGA
GCGTGACACCACGATGCCTGTAGCAATGGCAACAACGTTGCGCAAACTATTAACTGGCGAACTACTTACTCTAGCTTCCCGGCA
ACAATTAATAGACTGGATGGAGGCGGATAAAGTTGCAGGACCACTTCTGCGCTCGGCCCTTCCGGCTGGCTGGTTTATTGCTGA
TAAATCTGGAGCCGGTGAGCGTGGGTCTCGCGGTATCATTGCAGCACTGGGGCCAGATGGTAAGCCCTCCCGTATCGTAGTTAT
CTACACGACGGGGAGTCAGGCAACTATGGATGAACGAAATAGACAGATCGCTGAGATAGGTGCCTCACTGATTAAGCATTGGTA
ACTGTCAGACCAAGTTTACTCATATATACTTTAGATTGATTTAAAACTTCATTTTTAATTTAAAAGGATCTAGGTGAAGATCCT
TTTTGATAATCTCATGACCAAAATCCCTTAACGTGAGTTTTCGTTCCACTGAGCGTCAGACCCCGTAGAAAAGATCAAAGGATC
TTC-3'
```

pXyl-Sa-agrA

```
5'-
TTGAGATCCTTTTTTTCTGCGCGTAATCTGCTGCTTGCAAACAAAAAAACCACCGCTACCAGCGGTGGTTTGTTTGCCGGATCA
AGAGCTACCAACTCTTTTTCCGAAGGTAACTGGCTTCAGCAGAGCGCAGATACCAAATACTGTTCTTCTAGTGTAGCCGTAGTT
AGGCCACCACTTCAAGAACTCTGTAGCACCGCCTACATACCTCGCTCTGCTAATCCTGTTACCAGTGGCTGCTGCCAGTGGCGA
TAAGTCGTGTCTTACCGGGTTGGACTCAAGACGATAGTTACCGGATAAGGCGCAGCGGTCGGGCTGAACGGGGGGTTCGTGCAC
ACAGCCCAGCTTGGAGCGAACGACCTACACCGAACTGAGATACCTACAGCGTGAGCTATGAGAAAGCGCCACGCTTCCCGAAGG
GAGAAAGGCGGACAGGTATCCGGTAAGCGGCAGGGTCGGAACAGGAGAGCGCACGAGGGAGCTTCCAGGGGGAAACGCCTGGTA
TCTTTATAGTCCTGTCGGGTTTCGCCACCTCTGACTTGAGCGTCGATTTTTGTGATGCTCGTCAGGGGGGCGGAGCCTATGGAA
AAACGCCAGCAACGCGGCCTTTTTACGGTTCCTGGCCTTTTGCTGGCCTTTTGCTCACATGTTCTTTCCTGCGTTATCCCCTGA
TTCTGTGGATAACCGTATTACCGCCTTTGAGTGAGCTGATACCGCTCGCCGCAGCCGAACGACCGAGCGCAGCGAGTCAGTGAG
CGAGGAAGCGGAAGAGCGCCCAATACGCAAACCGCCTCTCCCCGCGCGTTGGCCGATTCATTAATGCAGCTGGCACGACAGGTT
TCCCGACTGGAAAGCGGGCAGTGAGCGCAACGCAATTAATGTGAGTTAGCTCACTCATTAGGCACCCCAGGCTTTACACTTTAT
GCTTCCGGCTCGTATGTTGTGTGAATTGTGAGCGGATAACAATTTCACACAGGAAACAGCTATGGCCATGATTACGCCAAGCTT
GCATGCCTGCATTATATTTTTTTAACGTTTCTCACCGATGCATAGCAGTGTTCTTTATTTTTAAAATAGACAATTCGCTCTTTC
GAATCTATAGATTCAATATTATGGCGATTGACGACAAAGCTATTATGACATCTAAAGAAACGATCATCTAATTGACTCAGTTCT
TTTAAATTACCATAAAATTCAATTTGACGGTTATCTAAATGGGCAATGAGTCTGTGAGATTTTGTTGATGATTCAAAAAACATA
ATATCATCATATTGAACATACACTGAATTACTGCCACGTTTTAATTCAATCGTTTCAACGCTATTATCTTTAGACAACAATTGT
AAGCGTGTATGTGCAGTTTCTAAACAGTCTATAATTCGAGTTCTTAATTCAGCTGGATCATCTTTAAAAATAAAATCCATCGCT
GCAACTTTGTAGACAAATGTTAAATAGGTAAGTTCACTGTGACTCGTAACGAAAATAATGTTACCAACTGGGTCATGCTTACGA
ATTTCACTGCCTAATTTGATACCATTAATATCAGTTGAAAGTTGAATATCTAAAAAGTAACAGCCTATGTCATTCATATTTTTA
GCTTGCTCAAGCACCTCATAAGGATTATCAGTTGCGAGGGCAATTTCCATAGGCTTTTCTTCTATCATTATATAATTTTTAATA
ATGGTAACCATGTTTTCTCTTTGTTTTGGATCGTCTTCGCAAATGAAAATTTTCATAGTAGTTCCTCCTTATCTACAGATGCAT
TTTATTTCATATAGTAAGTACATCACCTATTAGTTTGTTGTTTAAACAAACTAACTTATTTTCATCTTATATAACCTCGTCAGT
ATTTTCAATATTTTTTTTAGTTTTTTATGAACACATTAGATATAATAAAGGGAAGATTCGCTATGTACTATGTTGATACTTAAT
TTAAAGATTAAACAAATGGAGTGGATGAAGTGGATATCGCTGATCAAACCTTTGTCAAAAAAGTAAATCAAAAGTTATTATTAA
AAGAAATCCTTAAAAATTCACCTATTTCAAGAGCAAAATTATCTGAAATGACTGGATTAAATAAATCAACTGTCTCATCACAGG
TAAACACGTTAATGAAAGAAAGTATGGTATTTGAAATAGGTCAAGGACAATCAAGTGGCGGAAGAAGACCTGTCATGCTTGTTT
TTAATAAAAAGGCAGGATACTCCGTTGGAATAGATGTTGGTGTGGATTATATTAATGGCATTTTAACAGACCTTGAAGGAACAA
TCGTTCTTGATCAATACCGCCATTTGGAATCCAATTCTCCAGAAATAACGAAAGACATTTTGATTGATATGATTCATCACTTTA
TTACGCAAATGCCCCAATCTCCGTACGGGCTTATTGGTATAGGTATTTGCGTGCCTGGACTCATTGATAAAGATCAAAAAATTG
TTTTCACTCCGAACTCCAACTGGAGAGATATTGACTTAAAATCTTCGATACAAGAGAAGTACAATGTGCCTGTTTTTATTGAAA
ATGAGGCAAATGCTGGCGCATATGGAGAAAAAGTATTTGGAGCTGCAAAAAATCACGATAACATTATTTACGTAAGTATCAGCA
CAGGAATAGGGATCGGTGTTATTATCAACAATCATTTATATAGAGGAGTAAGCGGCTTCTCTGGAGAAATGGGACATATGACAA
TAGACTTTAATGGTCCTAAATGCAGTTGCGGAAACCGAGGATGCTGGGAATTGTATGCTTCAGAGAAGGCTTTATTAAAATCTC
TTCAGACCAAAGAGAAAAAACTGTCCTATCAAGATATCATAAACCTCGCCCATCTGAATGATATCGGAACCTTAAATGCATTAC
AAAATTTTGGATTCTATTTAGGAATAGGCCTTACCAATATTCTAAATACTTTCAACCCACAAGCCGTAATTTTAAGAAATAGCA
TAATTGAATCGCATCCTATGGTTTTAAATTCAATGAGAAGTGAAGTATCATCAAGGGTTTATTCCCAATTAGGCAATAGCTATG
AATTATTGCCATCTTCCTTAGGACAGAATGCACCGGCATTAGGAATGTCCTCCATTGTGATTGATCATTTTCTGGACATGATTA
CAATGTAATTTTTTATGGAATGGACAGCTCATCTTTAAAGATGAGTTTTTTTATTCTAGGAGTATTTCTGAAGCAATAGTGACA
TGGCACCTTCTCATATGAAAAAGGAGTTCTAAAATAGAAATCTCCTTTTTCATGTGCAAATTATTTTTCTTTATAACGAAAATA
TCTAAAGTCGGCCAATTCACTGGCCGTCGTTTTACCGAAATACGGGCAGACATGGCCTGCCCGGTTAATGCCTGGCTTCACTGT
CATTATACGATTTAGTACAATCTTTTATCATTATATTGCCTAACTGTAGGAAATAAATACTTAACTGTTAAATGTAATTTGTAT
TTAATATTTTAACATAAAAAAATTTACAGTTAAGAATAAAAAACGACTAGTTAAGAAAAATTGGAAAATAAATGCTTTTAGCAT
GTTTTAATATAACTAGATCACAGAGATGTGGTCAGTCAGTCTTAATTAACCAAGGAGGTGAAATGTACAATGAAAGGAGAAGAA
CTTTTCACTGGAGTTGTCCCAATTCTTGTTGAATTAGATGGTGATGTTAATGGGCACAAATTTTCTGTCAGTGGAGAGGGTGAA
GGTGATGCAACATACGGAAAACTTACCCTTAAATTTATTTGCACTACTGGAAAACTACCTGTTCCATGGCCAACACTTGTCACT
ACTTTCGCGTATGGTCTTCAATGCTTTGCGAGATACCCAGATCATATGAAACAGCATGACTTTTTCAAGAGTGCCATGCCCGAA
GGTTATGTACAGGAAAGAACTATATTTTTCAAAGATGACGGGAACTACAAGACACGTGCTGAAGTCAAGTTTGAAGGTGATACC
```

```
CTTGTTAATAGAATCGAGTTAAAAGGTATTGATTTTAAAGAAGATGGAAACATTCTTGGACACAAATTGGAATACAACTATAAC
TCACACAATGTATACATCATGGCAGACAAACAAAAGAATGGAATCAAAGTTAACTTCAAAATTAGACACAACATTGAAGATGGA
AGCGTTCAACTAGCAGACCATTATCAACAAAATACTCCAATTGGCGATGGCCCTGTCCTTTTACCAGACAACCATTACCTGTCC
ACACAATCTAAGCTTTCGAAAGATCCCAACGAAAAGAGAGACCACATGGTCCTTCTTGAGTTTGTAACAGCTGCTGGGATTACA
CATGGCATGGATGAACTATACAAATAACCTAGGCTGCTGCCACCGCTGAGCAATAACTAGCATAACCCCTTGGGGCCTCTAAAC
GGGTCTTGAGGGGTTTTTTGAACGTCGTGACTGGGAAAACCCTGGCGTTACCCAACTTAATCGCCTTGCAGCACATCCCCCTTT
CGCCAGCTGGCGTAATAGCGAAGAGGCCCGCACCGATCGCCCTTCCCAACAGTTGCGCAGCCTGAATGGCGAATGGCGCCTGAT
GCGGTATTTTCTCCTTACGCATCTGTGCGGTATTTCACACCGCATATGGTGCACTCTCAGTACAATCTGCTCTGATGCCGCATA
GTTAAGCCAGCCCCGACACCCGCCAACACCCGCTGACGCGCCCTGACGGGCTTGTCTGCTCCCGGCATCCGCTTACAGACAAGC
TGTGACCGTCTCCGGGAGCTGCATGTGTCAGAGGTTTTCACCGTCATCACCGAAACGCGCGAGACGAAAGGGCCTCGTGATACG
CCTATTTTTATAGGTTAATGTCATGATAATAATGGTTTCTTAGACGTCAGGTGGCACTTTTCGGGGAAATGTGCGCGGAACCCC
TATTTGTTTATTTTTCTAAATACATTCAAATATGTATCCGCTCATGAGACAATAACCCTGATAAATGCTTCAATAATCCATCCT
CCAAAGTTGGAGAGTGAGTTTTATGTCGCAAATATTAATGTTTCTGGTGAACCTTATCAAATTTTCGTTGATTTAATAGAAACA
TAGCGGTAAAATTAGCAGTAACTTAATAGAACGGAAATGAAAAAAGCCACTCTCATATGCTATTGGCTACCAACCTTTAGCGAG
AATGACTTAATCCTGTACAGCCATACAGGACTTCGACTTATAAGAGGCGCCAACTTCAAATAAGTTATTTGCCTTGTTTTCGCG
AACAAGGCTTATTAGATACACCTATTGTACCGTTACTCTACGAATATTTCAAGTAGTAATTACTAGCATTGTCCGTTACTCTAC
GAATATTTCAAGTAGTAATTACTAGCATTGTCCGTTACTCTACGAATATTTCAAGTAGTAATTACTAGCATTGTCATATACATA
ATAAAACGGATATAAAAGGGCGTTTTCTATACCTAGAAGTCTTGTAAATGTACAGGGCGTTTAGATATAGAGAACGCCCTTTTT
GTGTTCCGTTCCAGTGGAAGCTACCACTTTAAAAAGATGGTCTAGTGTAGCCAATGCAGGAGAGTACACTCGGATATCAGTTGT
CGTTGCATTCAACTGTCTGACGTAAGCGAGGTAAAGGACACAAGCCTTGCATAAAACAAGCCTACGGGATGTAAATCCTAATAA
TGATGATAACCAAGACGTTAGCGGCAAAAAGTGTTGGGGGTTCAAAATAAGACATGATTGTGCGACTGGAGTTAAACAGTTACT
CGTAAGCGGCGATCATGACACTGATTCACGGCTATTCTTGTACAAGCTAGCTTTATTACAAGGATATGCGGGTTATATAGCGAA
TCACCCGAAAGGGAACGGTGTTGGGCGTGAGAAACGCACCGTACGGCGCAATACAATGCCAATAAGCTATATACGGACGGTATA
GTAGTTTTGTAAGCTATAACCGTTTGTCGTCAATGCAACCAATCTCAATTCGAGACCTCGGCATCTAAGCCAGTACGAATGAGT
GGGCGTTTTAACCTCGTAAATTTTCAACAGGGGTTACTATGCCCAAAACTACATTCAGATTTCCTAACAAACTCGCCAGTATGA
AAACCTTAAGACCTTAAAGTCAAGGGATTTGAAGGATTTTAACCTCGATTAGCAAAAAATGTAGAGTACTGAAGCAACTACCAT
TAACTAAGATAGTGGGGGATTGAGGAAGAATCCAGAGCTGTTTAAATCAAGTGAAAGACAAGATGAAATTAAAAGAATAGTGAA
AGATAGGGGAGTGGTTCTCTATGAGAAAGGAAATGGCTAGAGAACAAAGGCAGCGGTTTATTGATCTATTGTTAGACTTTATGG
TAAAGAATCCTCATTTATTTGTTAATGGTACAGAGGATGAAAGTAATAATGTTGTTACAAAATGTAATAGTGATATTAAAGAGG
TTGCGGAGTCATATTTAACTCTTTTATAGTGAGAGGGTTAAAACTAATTAATATGTATTAAGGCCCAATGTTGGAATTATTGTA
TTTCACTAGGCAACCTACTTACTAAAAGTAAGATTATCCATTAGTGGATGTTATAATATTGGGTTTTTTAACACAATAATCATC
GCCTTTCGGTGTCGTTTGATAGAAAAGTAACCATTAGCGATGAAAAAGTCAATATAAAAAGCCATCCGTAAAAAACGGATGGCT
TACCGTACATAGGATCGTTGGTAGGGCGGCGTATCCTACATCTCTGGTAACTTACCTAGCCAATCAAATGCTTGAGAACGGCGG
TTAGATAAGCGCGTGGGGAACCTTTCCCACCTCAAAGATCCTATATCATTATTATGTTACTTTCTACAGGTAGTATACCATGTT
CTTATATTTTAGTAAACTCCCCGTTAGCTTAACAGGTCTTTGTAAGCAATTAAACGTCCACTATTCAATCGTCTTTGGATTTTC
GCAGGACCGTTTTTTAGATCGAACATAGTTGATAAGAACAAATAACCGCTTGGGTCCAACTTTATAGCAATTAGTATATGGTCA
TTTAAAATCTTTACCAATTCAACGCTATTAGGTTCTTTAGGATTTTGCCCGACATAGTCGGGGTGTTCAACGATATCTTTTATG
TGCGATGAATATTTTTCATAAATACCAGGATGTTGTTTCTTTACGTGCTTTATAAATCCGGGAAACATTTTTACATCGTTAGAA
GTGCAAGTCAAGTTATATGTATCTATAATGATTTGTGGAAGTTTTGCCACAACAGTTGGTTTATTTACAATCTTTTTTTTATTA
GCCGTCAAATTTCTCCCTCATCTCGTCTCTTTATATCTTTATTTTATCATAAAGGAGTATTTGAACCGTCGCGCGGGACAGGTT
TATGATAGGGATATTTTATTGAATAATTGATGGTATAAGGGACTTTCATGCTTGGAAAGTGGGGATTATGAATTAGATGCTTGT
CCACAATATGTTCCAATGTAATTAAAATTTATGTTCCCACCTTGACCAAACATCACGTCCATACTTAAATCGTCCCTCCTTTAA
TAGGTAAAATATTAATTTACCTTAATAAAAAAATAATGGATAATAGTATTCGTCTGAATTTATATAATCAGGGGGAACTATTGA
TGCTGGGGATACTATTTACAGCGGCGCCATCTACTGATGTCGTAAAGGATTTGCAAGATAAAGTTATATCATTGCAGGATCATG
AGGTAGCGTTTTTGAACACCACGATATCTAATATGTTGATCCCCGAAGCAAACTTAAGAGTGTGTTGATAGTGCAGTATCTTAA
AATTTTGTGTATAATAGGAATTGAAGTTAAATTAGATGCTAAAAATTTGTAATTAAGAAGGAGGGATTCGTCATGTTGGTATTC
CAAATGCGTAATGTAGATAAAACATCTACTGTTTTGAAACAGACTAAAAACAGTGATTACGCAGATAAATAAATACGTTAGATT
AATTCCTACCAGTGACTAATCTTATGACTTTTTAAACAGATAACTAAAATTACAAACAAATCGTTTAACTTCTGTATTTATTTA
CAGATGTAATCACTTCAGGAGTAATTACATGAACAAAAATATAAAATATTCTCAAAACTTTTTAACGAGTGAAAAAGTACTCAA
CCAAATAATAAAACAATTGAATTTAAAAGAAACCGATACCGTTTACGAAATTGGAACAGGTAAAGGGCATTTAACGACGAAACT
GGCTAAAATAAGTAAACAGGTAACGTCTATTGAATTAGACAGTCATCTATTCAACTTATCGTCAGAAAAATTAAAACTGAACAT
TCGTGTCACTTTAATTCACCAAGATATTCTACAGTTTCAATTCCCTAACAAACAGAGGTATAAAATTGTTGGGAGTATTCCTTA
CCATTTAAGCACACAAATTATTAAAAAAGTGGTTTTTGAAAGCCATGCGTCTGACATCTATCTGATTGTTGAAGAAGGATTCTA
CAAGCGTACCTTGGATATTCACCGAACACTAGGGTTGCTCTTGCACACTCAAGTCTCGATTCAGCAATTGCTTAAGCTGCCAGC
GGAATGCTTTCATCCTAAACCAAAAGTAAACAGTGTCTTAATAAAACTTACCCGCCATACCACAGATGTTCCAGATAAATATTG
GAAGCTATATACGTACTTTGTTTCAAAATGGGTCAATCGAGAATATCGTCAACTGTTTACTAAAAATCAGTTTCATCAAGCAAT
GAAACACGCCAAAGTAAACAATTTAAGTACCATTACTTATGAGCAAGTATTGTCTATTTTTAATAGTTATCTATTATTTAACGG
GAGGAAATAATTCTATGAGTCGCTTTTTTAAATTTGGAAAGTTACACGTTACTAAAGGGAATGGAGATAAATTATTAGATATAC
TACTGACAGCTTCCAAGAAGCTAAAGAGGTCCCTAGCGCCTACGGGGAATTTGGGGTACATTGAAAAAGGAAGAGTATGAGTAT
TCAACATTTCCGTGTCGCCCTTATTCCCTTTTTTGCGGCATTTTGCCTTCCTGTTTTTGCTCACCCAGAAACGCTGGTGAAAGT
AAAAGATGCTGAAGATCAGTTGGGTGCACGAGTGGGTTACATCGAACTGGATCTCAACAGCGGTAAGATCCTTGAGAGTTTTCG
CCCCGAAGAACGTTTTCCAATGATGAGCACTTTTAAAGTTCTGCTATGTGGCGCGGTATTATCCCGTATTGACGCCGGGCAAGA
GCAACTCGGTCGCCGCATACACTATTCTCAGAATGACTTGGTTGAGTACTCACCAGTCACAGAAAAGCATCTTACGGATGGCAT
```

```
GACAGTAAGAGAATTATGCAGTGCTGCCATAACCATGAGTGATAACACTGCGGCCAACTTACTTCTGACAACGATCGGAGGACC
GAAGGAGCTAACCGCTTTTTTGCACAACATGGGGGATCATGTAACTCGCCTTGATCGTTGGGAACCGGAGCTGAATGAAGCCAT
ACCAAACGACGAGCGTGACACCACGATGCCTGTAGCAATGGCAACAACGTTGCGCAAACTATTAACTGGCGAACTACTTACTCT
AGCTTCCCGGCAACAATTAATAGACTGGATGGAGGCGGATAAAGTTGCAGGACCACTTCTGCGCTCGGCCCTTCCGGCTGGCTG
GTTTATTGCTGATAAATCTGGAGCCGGTGAGCGTGGGTCTCGCGGTATCATTGCAGCACTGGGGCCAGATGGTAAGCCCTCCCG
TATCGTAGTTATCTACACGACGGGGAGTCAGGCAACTATGGATGAACGAAATAGACAGATCGCTGAGATAGGTGCCTCACTGAT
TAAGCATTGGTAACTGTCAGACCAAGTTTACTCATATATACTTTAGATTGATTTAAAACTTCATTTTTAATTTAAAAGGATCTA
GGTGAAGATCCTTTTTGATAATCTCATGACCAAAATCCCTTAACGTGAGTTTTCGTTCCACTGAGCGTCAGACCCCGTAGAAAA
GATCAAAGGATCTTC-3'
```

pXyl-Se-agrBD

```
5'-
TTGAGATCCTTTTTTTCTGCGCGTAATCTGCTGCTTGCAAACAAAAAAACCACCGCTACCAGCGGTGGTTTGTTTGCCGGATCA
AGAGCTACCAACTCTTTTTCCGAAGGTAACTGGCTTCAGCAGAGCGCAGATACCAAATACTGTTCTTCTAGTGTAGCCGTAGTT
AGGCCACCACTTCAAGAACTCTGTAGCACCGCCTACATACCTCGCTCTGCTAATCCTGTTACCAGTGGCTGCTGCCAGTGGCGA
TAAGTCGTGTCTTACCGGGTTGGACTCAAGACGATAGTTACCGGATAAGGCGCAGCGGTCGGGCTGAACGGGGGGTTCGTGCAC
ACAGCCCAGCTTGGAGCGAACGACCTACACCGAACTGAGATACCTACAGCGTGAGCTATGAGAAAGCGCCACGCTTCCCGAAGG
GAGAAAGGCGGACAGGTATCCGGTAAGCGGCAGGGTCGGAACAGGAGAGCGCACGAGGGAGCTTCCAGGGGGAAACGCCTGGTA
TCTTTATAGTCCTGTCGGGTTTCGCCACCTCTGACTTGAGCGTCGATTTTTGTGATGCTCGTCAGGGGGGCGGAGCCTATGGAA
AAACGCCAGCAACGCGGCCTTTTTACGGTTCCTGGCCTTTTGCTGGCCTTTTGCTCACATGTTCTTTCCTGCGTTATCCCCTGA
TTCTGTGGATAACCGTATTACCGCCTTTGAGTGAGCTGATACCGCTCGCCGCAGCCGAACGACCGAGCGCAGCGAGTCAGTGAG
CGAGGAAGCGGAAGAGCGCCCAATACGCAAACCGCCTCTCCCCGCGCGTTGGCCGATTCATTAATGCAGCTGGCACGACAGGTT
TCCCGACTGGAAAGCGGGCAGTGAGCGCAACGCAATTAATGTGAGTTAGCTCACTCATTAGGCACCCCAGGCTTTACACTTTAT
GCTTCCGGCTCGTATGTTGTGTGGAATTGTGAGCGGATAACAATTTCACACAGGAAACAGCTATGACCATGATTACGCCAAGCT
TGCATGCCTGCATTACTCGTATAGTTTAGTCAGTTCTTCTGGTACTTCTGGTTCGTCAAAGTAAGAGGCACATACACTATCTCC
TGCTACAGTACCAATAAATTCCAAGATTGTAGTGAAAAATTTTATAAATAAATTAAAAATGTTTTCCATGATTAATATCCTCCT
TAGGGAAAAAGATGGGTAGTAATGTTAAAGATTCTAAAATTACACCGAATAAAATAAGTTTATTTACCGGTTCTTTAGTTACTA
ATGAAATAACTACGATAGTACAATATAAAAATATGGAGAGTATTTTTTTTCGCTTTACAAGACGTCTAGGTATAGGTTGTTTCT
TAGTTGCTGCAGGTGCGTATAAAATGGTAATAATTAATCCGACTAATGCCATAGATAAAAGAACAAAATAGTTAATATCTAACT
TTATTATTAAGTATGGAAAGATAATAAAGAAAATTATGTTCTGAATATGACATAACAATGACGAATTTGCATGCGTACCGTGTG
CATGTCTCCTAATTAAAAAATAACTTAAATGAGTTAAAAGTGTGTAAAAGAAAGTATGAAAGATTATTGCTAGCCCATACACAA
CTATAGACTTTTCAATATTTATCGCTAGTACCTGCATCCCTAAACGAATTTTTAGAAACTGTATGTGATCTAAGTTATTTTTAC
GTTGTAAATATTGAGCAAATTGCTCAATTTTTTTATCGATGATTTTCATAGTAGTTCCTCCTTATCTACAGATGCATTTTATTT
CATATAGTAAGTACATCACCTATTAGTTTGTTGTTTAAACAAACTAACTTATTTTCATCTTATATAACCTCGTCAGTATTTTCA
ATATTTTTTTTAGTTTTTTATGAACACATTAGATATAATAAAGGGAAGATTCGCTATGTACTATGTTGATACTTAATTTAAAGA
TTAAACAAATGGAGTGGATGAAGTGGATATCGCTGATCAAACCTTTGTCAAAAAAGTAAATCAAAAGTTATTATTAAAAGAAAT
CCTTAAAAATTCACCTATTTCAAGAGCAAAATTATCTGAAATGACTGGATTAAATAAATCAACTGTCTCATCACAGGTAAACAC
GTTAATGAAAGAAAGTATGGTATTTGAAATAGGTCAAGGACAATCAAGTGGCGGAAGAAGACCTGTCATGCTTGTTTTTAATAA
AAAGGCAGGATACTCCGTTGGAATAGATGTTGGTGTGGATTATATTAATGGCATTTTAACAGACCTTGAAGGAACAATCGTTCT
TGATCAATACCGCCATTTGGAATCCAATTCTCCAGAAATAACGAAAGACATTTTGATTGATATGATTCATCACTTTATTACGCA
AATGCCCCAATCTCCGTACGGGCTTATTGGTATAGGTATTTGCGTGCCTGGACTCATTGATAAAGATCAAAAAATTGTTTTCAC
TCCGAACTCCAACTGGAGAGATATTGACTTAAAATCTTCGATACAAGAGAAGTACAATGTGCCTGTTTTTATTGAAAATGAGGC
AAATGCTGGCGCATATGGAGAAAAAGTATTTGGAGCTGCAAAAAATCACGATAACATTATTTACGTAAGTATCAGCACAGGAAT
AGGGATCGGTGTTATTATCAACAATCATTTATATAGAGGAGTAAGCGGCTTCTCTGGAGAAATGGGACATATGACAATAGACTT
TAATGGTCCTAAATGCAGTTGCGGAAACCGAGGATGCTGGGAATTGTATGCTTCAGAGAAGGCTTTATTAAAATCTCTTCAGAC
CAAAGAGAAAAAACTGTCCTATCAAGATATCATAAACCTCGCCCATCTGAATGATATCGGAACCTTAAATGCATTACAAAATTT
TGGATTCTATTTAGGAATAGGCCTTACCAATATTCTAAATACTTTCAACCCACAAGCCGTAATTTTAAGAAATAGCATAATTGA
ATCGCATCCTATGGTTTTAAATTCAATGAGAAGTGAAGTATCATCAAGGGTTTATTCCCAATTAGGCAATAGCTATGAATTATT
GCCATCTTCCTTAGGACAGAATGCACCGGCATTAGGAATGTCCTCCATTGTGATTGATCATTTTCTGGACATGATTACAATGTA
ATTTTTTATGGAATGGACAGCTCATCTTTAAAGATGAGTTTTTTTATTCTAGGAGTATTTCTGAAGCAATAGTGACATGGCACC
TTCTCATATGAAAAAGGAGTTCTAAAATAGAAATCTCCTTTTTCATGTGCAAATTATTTTTCTTTATAACGAAAATATCTAAAG
TCGGCCAATTCACTGGCCGTCGTTTTACAACGTCGTGACTGGGAAAACCCTGGCGTTACCCAACTTAATCGCCTTGCAGCACAT
CCCCCTTTCGCCAGCTGGCGTAATAGCGAAGAGGCCCGCACCGATCGCCCTTCCCAACAGTTGCGCAGCCTGAATGGCGAATGG
CGCCTGATGCGGTATTTTCTCCTTACGCATCTGTGCGGTATTTCACACCGCATATGGTGCACTCTCAGTACAATCTGCTCTGAT
GCCGCATAGTTAAGCCAGCCCCGACACCCGCCAACACCCGCTGACGCGCCCTGACGGGCTTGTCTGCTCCCGGCATCCGCTTAC
AGACAAGCTGTGACCGTCTCCGGGAGCTGCATGTGTCAGAGGTTTTCACCGTCATCACCGAAACGCGCGAGACGAAAGGGCCTC
GTGATACGCCTATTTTTATAGGTTAATGTCATGATAATAATGGTTTCTTAGACGTCAGGTGGCACTTTTCGGGGAAATGTGCGC
GGAACCCCTATTTGTTTATTTTTCTAAATACATTCAAATATGTATCCGCTCATGAGACAATAACCCTGATAAATGCTTCAATAA
TCCATCCTCCAAAGTTGGAGAGTGAGTTTTATGTCGCAAATATTAATGTTTCTGGTGAACCTTATCAAATTTTCGTTGATTTAA
TAGAAACATAGCGGTAAAATTAGCAGTAACTTAATAGAACGGAAATGAAAAAAGCCACTCTCATATGCTATTGGCTACCAACCT
TTAGCGAGAATGACTTAATCCTGTACAGCCATACAGGACTTCGACTTATAAGAGGCGCCAACTTCAAATAAGTTATTTGCCTTG
TTTTCGCGAACAAGGCTTATTAGATACACCTATTGTACCGTTACTCTACGAATATTTCAAGTAGTAATTACTAGCATTGTCCGT
```

```
TACTCTACGAATATTTCAAGTAGTAATTACTAGCATTGTCCGTTACTCTACGAATATTTCAAGTAGTAATTACTAGCATTGTCA
TATACATAATAAAACGGATATAAAAGGGCGTTTTCTATACCTAGAAGTCTTGTAAATGTACAGGGCGTTTAGATATAGAGAACG
CCCTTTTTGTGTTCCGTTCCAGTGGAAGCTACCACTTTAAAAAGATGGTCTAGTGTAGCCAATGCAGGAGAGTACACTCGGATA
TCAGTTGTCGTTGCATTCAACTGTCTGACGTAAGCGAGGTAAAGGACACAAGCCTTGCATAAAACAAGCCTACGGGATGTAAAT
CCTAATAATGATGATAACCAAGACGTTAGCGGCAAAAAGTGTTGGGGGTTCAAAATAAGACATGATTGTGCGACTGGAGTTAAA
CAGTTACTCGTAAGCGGCGATCATGACACTGATTCACGGCTATTCTTGTACAAGCTAGCTTTATTACAAGGATATGCGGGTTAT
ATAGCGAATCACCCGAAAGGGAACGGTGTTGGGCGTGAGAAACGCACCGTACGGCGCAATACAATGCCAATAAGCTATATACGG
ACGGTATAGTAGTTTTGTAAGCTATAACCGTTTGTCGTCAATGCAACCAATCTCAATTCGAGACCTCGGCATCTAAGCCAGTAC
GAATGAGTGGGCGTTTTAACCTCGTAAATTTTCAACAGGGGTTACTATGCCCAAAACTACATTCAGATTTCCTAACAAACTCGC
CAGTATGAAAACCTTAAGACCTTAAAGTCAAGGGATTTGAAGGATTTTAACCTCGATTAGCAAAAAATGTAGAGTACTGAAGCA
ACTACCATTAACTAAGATAGTGGGGGATTGAGGAAGAATCCAGAGCTGTTTAAATCAAGTGAAAGACAAGATGAAATTAAAAGA
ATAGTGAAAGATAGGGGAGTGGTTCTCTATGAGAAAGGAAATGGCTAGAGAACAAAGGCAGCGGTTTATTGATCTATTGTTAGA
CTTTATGGTAAAGAATCCTCATTTATTTGTTAATGGTACAGAGGATGAAAGTAATAATGTTGTTACAAAATGTAATAGTGATAT
TAAAGAGGTTGCGGAGTCATATTTAACTCTTTTATAGTGAGAGGGTTAAAACTAATTAATATGTATTAAGGCCCAATGTTGGAA
TTATTGTATTTCACTAGGCAACCTACTTACTAAAAGTAAGATTATCCATTAGTGGATGTTATAATATTGGGTTTTTTAACACAA
TAATCATCGCCTTTCGGTGTCGTTTGATAGAAAAGTAACCATTAGCGATGAAAAAGTCAATATAAAAAGCCATCCGTAAAAAAC
GGATGGCTTACCGTACATAGGATCGTTGGTAGGGCGGCGTATCCTACATCTCTGGTAACTTACCTAGCCAATCAAATGCTTGAG
AACGGCGGTTAGATAAGCGCGTGGGGAACCTTTCCCACCTCAAAGATCCTATATCATTATTATGTTACTTTCTACAGGTAGTAT
ACCATGTTCTTATATTTTAGTAAACTCCCCGTTAGCTTAACAGGTCTTTGTAAGCAATTAAACGTCCACTATTCAATCGTCTTT
GGATTTTCGCAGGACCGTTTTTTAGATCGAACATAGTTGATAAGAACAAATAACCGCTTGGGTCCAACTTTATAGCAATTAGTA
TATGGTCATTTAAAATCTTTACCAATTCAACGCTATTAGGTTCTTTAGGATTTTGCCCGACATAGTCGGGGTGTTCAACGATAT
CTTTTATGTGCGATGAATATTTTTCATAAATACCAGGATGTTGTTTCTTTACGTGCTTTATAAATCCGGGAAACATTTTTACAT
CGTTAGAAGTGCAAGTCAAGTTATATGTATCTATAATGATTTGTGGAAGTTTTGCCACAACAGTTGGTTTATTTACAATCTTTT
TTTTATTAGCCGTCAAATTTCTCCCTCATCTCGTCTCTTTATATCTTTATTTTATCATAAAGGAGTATTTGAACCGTCGCGCGG
GACAGGTTTATGATAGGGATATTTTATTGAATAATTGATGGTATAAGGGACTTTCATGCTTGGAAAGTGGGGATTATGAATTAG
ATGCTTGTCCACAATATGTTCCAATGTAATTAAAATTTATGTTCCCACCTTGACCAAACATCACGTCCATACTTAAATCGTCCC
TCCTTTAATAGGTAAAATATTAATTTACCTTAATAAAAAAATAATGGATAATAGTATTCGTCTGAATTTATATAATCAGGGGGA
ACTATTGATGCTGGGGATACTATTTACAGCGGCGCCATCTACTGATGTCGTAAAGGATTTGCAAGATAAAGTTATATCATTGCA
GGATCATGAGGTAGCGTTTTTGAACACCACGATATCTAATATGTTGATCCCCGAAGCAAACTTAAGAGTGTGTTGATAGTGCAG
TATCTTAAAATTTTGTGTATAATAGGAATTGAAGTTAAATTAGATGCTAAAAATTTGTAATTAAGAAGGAGGGATTCGTCATGT
TGGTATTCCAAATGCGTAATGTAGATAAAACATCTACTGTTTTGAAACAGACTAAAAACAGTGATTACGCAGATAAATAAATAC
GTTAGATTAATTCCTACCAGTGACTAATCTTATGACTTTTTAAACAGATAACTAAAATTACAAACAAATCGTTTAACTTCTGTA
TTTATTTACAGATGTAATCACTTCAGGAGTAATTACATGAACAAAAATATAAAATATTCTCAAAACTTTTTAACGAGTGAAAAA
GTACTCAACCAAATAATAAAACAATTGAATTTAAAAGAAACCGATACCGTTTACGAAATTGGAACAGGTAAAGGGCATTTAACG
ACGAAACTGGCTAAAATAAGTAAACAGGTAACGTCTATTGAATTAGACAGTCATCTATTCAACTTATCGTCAGAAAAATTAAAA
CTGAACATTCGTGTCACTTTAATTCACCAAGATATTCTACAGTTTCAATTCCCTAACAAACAGAGGTATAAAATTGTTGGGAGT
ATTCCTTACCATTTAAGCACACAAATTATTAAAAAAGTGGTTTTTGAAAGCCATGCGTCTGACATCTATCTGATTGTTGAAGAA
GGATTCTACAAGCGTACCTTGGATATTCACCGAACACTAGGGTTGCTCTTGCACACTCAAGTCTCGATTCAGCAATTGCTTAAG
CTGCCAGCGGAATGCTTTCATCCTAAACCAAAAGTAAACAGTGTCTTAATAAAACTTACCCGCCATACCACAGATGTTCCAGAT
AAATATTGGAAGCTATATACGTACTTTGTTTCAAAATGGGTCAATCGAGAATATCGTCAACTGTTTACTAAAAATCAGTTTCAT
CAAGCAATGAAACACGCCAAAGTAAACAATTTAAGTACCATTACTTATGAGCAAGTATTGTCTATTTTTAATAGTTATCTATTA
TTTAACGGGAGGAAATAATTCTATGAGTCGCTTTTTTAAATTTGGAAAGTTACACGTTACTAAAGGGAATGGAGATAAATTATT
AGATATACTACTGACAGCTTCCAAGAAGCTAAAGAGGTCCCTAGCGCCTACGGGGAATTTGGGGTACATTGAAAAAGGAAGAGT
ATGAGTATTCAACATTTCCGTGTCGCCCTTATTCCCTTTTTTGCGGCATTTTGCCTTCCTGTTTTTGCTCACCCAGAAACGCTG
GTGAAAGTAAAAGATGCTGAAGATCAGTTGGGTGCACGAGTGGGTTACATCGAACTGGATCTCAACAGCGGTAAGATCCTTGAG
AGTTTTCGCCCCGAAGAACGTTTTCCAATGATGAGCACTTTTAAAGTTCTGCTATGTGGCGCGGTATTATCCCGTATTGACGCC
GGGCAAGAGCAACTCGGTCGCCGCATACACTATTCTCAGAATGACTTGGTTGAGTACTCACCAGTCACAGAAAAGCATCTTACG
GATGGCATGACAGTAAGAGAATTATGCAGTGCTGCCATAACCATGAGTGATAACACTGCGGCCAACTTACTTCTGACAACGATC
GGAGGACCGAAGGAGCTAACCGCTTTTTTGCACAACATGGGGGATCATGTAACTCGCCTTGATCGTTGGGAACCGGAGCTGAAT
GAAGCCATACCAAACGACGAGCGTGACACCACGATGCCTGTAGCAATGGCAACAACGTTGCGCAAACTATTAACTGGCGAACTA
CTTACTCTAGCTTCCCGGCAACAATTAATAGACTGGATGGAGGCGGATAAAGTTGCAGGACCACTTCTGCGCTCGGCCCTTCCG
GCTGGCTGGTTTATTGCTGATAAATCTGGAGCCGGTGAGCGTGGGTCTCGCGGTATCATTGCAGCACTGGGGCCAGATGGTAAG
CCCTCCCGTATCGTAGTTATCTACACGACGGGGAGTCAGGCAACTATGGATGAACGAAATAGACAGATCGCTGAGATAGGTGCC
TCACTGATTAAGCATTGGTAACTGTCAGACCAAGTTTACTCATATATACTTTAGATTGATTTAAAACTTCATTTTTAATTTAAA
AGGATCTAGGTGAAGATCCTTTTTGATAATCTCATGACCAAAATCCCTTAACGTGAGTTTTCGTTCCACTGAGCGTCAGACCCC
GTAGAAAAGATCAAAGGATCTTC-3’
```

pXyl-Se-agrCA-GFP
5'-<br>TTGAGATCCTTTTTTTCTGCGCGTAATCTGCTGCTTGCAAACAAAAAAACCACCGCTACCAGCGGTGGTTTGTTTGCCGGATCA<br>AGAGCTACCAACTCTTTTTCCGAAGGTAACTGGCTTCAGCAGAGCGCAGATACCAAATACTGTTCTTCTAGTGTAGCCGTAGTT<br>AGGCCACCACTTCAAGAACTCTGTAGCACCGCCTACATACCTCGCTCTGCTAATCCTGTTACCAGTGGCTGCTGCCAGTGGCGA<br>TAAGTCGTGTCTTACCGGGTTGGACTCAAGACGATAGTTACCGGATAAGGCGCAGCGGTCGGGCTGAACGGGGGGTTCGTGCAC<br>ACAGCCCAGCTTGGAGCGAACGACCTACACCGAACTGAGATACCTACAGCGTGAGCTATGAGAAAGCGCCACGCTTCCCGAAGG<br>GAGAAAGGCGGACAGGTATCCGGTAAGCGGCAGGGTCGGAACAGGAGAGCGCACGAGGGAGCTTCCAGGGGGAAACGCCTGGTA<br>TCTTTATAGTCCTGTCGGGTTTCGCCACCTCTGACTTGAGCGTCGATTTTTGTGATGCTCGTCAGGGGGGCGGAGCCTATGGAA<br>AAACGCCAGCAACGCGGCCTTTTTACGGTTCCTGGCCTTTTGCTGGCCTTTTGCTCACATGTTCTTTCCTGCGTTATCCCCTGA<br>TTCTGTGGATAACCGTATTACCGCCTTTGAGTGAGCTGATACCGCTCGCCGCAGCCGAACGACCGAGCGCAGCGAGTCAGTGAG<br>CGAGGAAGCGGAAGAGCGCCCAATACGCAAACCGCCTCTCCCCGCGCGTTGGCCGATTCATTAATGCAGCTGGCACGACAGGTT<br>TCCCGACTGGAAAGCGGGCAGTGAGCGCAACGCAATTAATGTGAGTTAGCTCACTCATTAGGCACCCCAGGCTTTACACTTTAT<br>GCTTCCGGCTCGTATGTTGTGTGGAATTGTGAGCGGATAACAATTTCACACAGGAAACAGCTATGACCATGATTACGCCAAGCT<br>TGCATGCCTGCATTATATTTTTTTAACATTACGTACTGAAGCGAAACAATTTTCGCCATTCTTAAAGTAAACAATACGTTCTTT<br>TGAGTCAATAGATTCAATATTATGCCTGTTTATTACAAAACTGTTATGACATCTAAAGAAACGTTCATCAAGCTGTGCTAATTC<br>CTTTAAATTTCCATAAAATTCAATTTGTCGATTATCAAGATGTGCAATGAGTCTATGAGATTTCGTAGATGATTCAAAAAACAT<br>AATATCATCATATTGAACGTATACTGAATTACTTCCCCGCTTTAACTCAATCGTATCTACATTACTTTCTTTTGATAATAATTT<br>GAGTCGTGTATGTGCTGTTTCAAGACAATCTATGATTCTCATTTTTAATTCAGATGGATCATCTTTAAAAATAAAATCCATAGC<br>AGCCACTTTATAAACAAACGTCAAATAAGTCAGCTCACTGTGACTGGTTACAAATATAATATTACCAACAGGATCATGTTTACG<br>AATTTCACTGGCTAATTTAATACCGTTCATATCAGCTTCTAATTGAATATCAAGGAAGTAACAACCAATGTCATTAAGTTCTTT<br>TGATTGCTCTAAGACCTCATAAGGATCATTTGTTGCTAAAGCTAACTCCATTGGCTTTTCTTCAATCATTATGTAGTTTTTAAT<br>GATTGATACCATATGTTCTCTTTGTCTTTGGTCATCTTCACAAACAAAAATTTTCATTTAATTCTACACATCCTTATGATTCCT<br>TATTATTTATTTCTACTTTTTGTACAAAGTAACCATTTTCGATGACAGTATCTAATAAAACATTCTCGTTTGAGTCTGTCAGTT<br>CTTTTAAAGTTGATAAACCTAAACCGCGATTATCACCTTTAGTAGAAAAACCTTGTTCAAACAACTCATGAATTTTAGGGATAT<br>CATTACTACATTTATTCATAACGATAAAAGTGACAGATTCCTCATTATCGATGAATGCGATATTGATGAGTGGTTCCTCAAGAT<br>TTTCTGAAGCTTCAATAGCATTATCAACTATAATACCGATAATACGACTTAGCTCAACAGTATTCATATCGATACGATCAATTT<br>CATCAGGAACCTCAATACTAATTGGAATACGTTTTTCTTGAGCTTGAATAATTTTAGTAGTAATCAGCCCTTTAATTTCTCTCA<br>CTTTCAACTTTTCAATACCATTCATTTTAATAGAGCGAGTTTTTAATTTATCTTTCATTGGAACGATATTTTCATTAAAATATT<br>TACGTAATCCAGGCATATCATCTTCTCTAATGTAATCTGAAAGAGTGGTGAGGATATTCACATAATCATGTCGGAACTTACGCA<br>TTTCATTGTTAATGCTTTCTATACGTAACGTGTACTCATAATATGCTTCGATTTCTTTTACATTACGTTTATACCTCATTTCAC<br>GGAGTGTAAAATTGGACATCACTAATATCACTACACTTAAAAAAACCATAATACCCAACAATAAGATGGCATATAATTTAAGGG<br>TATCATTTCCTCGCATATCAGTTTGTGACACCATATAAAGTAAAATAAATGATATGAAAAGTACTATTGTTATTATCAATAAGT<br>ATCTTTTATTGAGTGACAAGTAGGATACTTTTAATTTATTGAACAATAGTTGAGTTAAATAAGCAATAATTAGAGTTATGATTA<br>CAAAAGAGGTAAAATGTATTAACTGTAAAGCAAATTTAAACGGAATATAATCTTTTATAGTCAAATGTATGTATACAGTTATGA<br>AATTAGTTATATATAAGATCATAGTGGTGAATAATACAACTAATATTGAATAAAGCTTTATTTTTGTATAAAAGAAAATGGTGA<br>TTATTATAACCAAAACTATTAATGCTTTACTTTGCCAAAAGTAATACATTATTGCAGAAGGGATTACAATCGTAAAAACGATTA<br>TGTAATCCCTAAAATTAAATTTCATATTAATGATAACTTTAGTAACCCAAATCATTAAAAAGATTTGTAGGCCTGCAAACGGAA<br>ATAGATTAATATCATCCATAGTAGTTCCTCCTTATCTACAGATGCATTTTATTTCATATAGTAAGTACATCACCTATTAGTTTG<br>TTGTTTAAACAAACTAACTTATTTTCATCTTATATAACCTCGTCAGTATTTTCAATATTTTTTTTAGTTTTTTATGAACACATT<br>AGATATAATAAAGGGAAGATTCGCTATGTACTATGTTGATACTTAATTTAAAGATTAAACAAATGGAGTGGATGAAGTGGATAT<br>CGCTGATCAAACCTTTGTCAAAAAAGTAAATCAAAAGTTATTATTAAAAGAAATCCTTAAAAATTCACCTATTTCAAGAGCAAA<br>ATTATCTGAAATGACTGGATTAAATAAATCAACTGTCTCATCACAGGTAAACACGTTAATGAAAGAAAGTATGGTATTTGAAAT<br>AGGTCAAGGACAATCAAGTGGCGGAAGAAGACCTGTCATGCTTGTTTTTAATAAAAAGGCAGGATACTCCGTTGGAATAGATGT<br>TGGTGTGGATTATATTAATGGCATTTTAACAGACCTTGAAGGAACAATCGTTCTTGATCAATACCGCCATTTGGAATCCAATTC<br>TCCAGAAATAACGAAAGACATTTTGATTGATATGATTCATCACTTTATTACGCAAATGCCCCAATCTCCGTACGGGCTTATTGG<br>TATAGGTATTTGCGTGCCTGGACTCATTGATAAAGATCAAAAAATTGTTTTCACTCCGAACTCCAACTGGAGAGATATTGACTT<br>AAAATCTTCGATACAAGAGAAGTACAATGTGCCTGTTTTTATTGAAAATGAGGCAAATGCTGGCGCATATGGAGAAAAAGTATT<br>TGGAGCTGCAAAAAATCACGATAACATTATTTACGTAAGTATCAGCACAGGAATAGGGATCGGTGTTATTATCAACAATCATTT<br>ATATAGAGGAGTAAGCGGCTTCTCTGGAGAAATGGGACATATGACAATAGACTTTAATGGTCCTAAATGCAGTTGCGGAAACCG<br>AGGATGCTGGGAATTGTATGCTTCAGAGAAGGCTTTATTAAAATCTCTTCAGACCAAAGAGAAAAAACTGTCCTATCAAGATAT<br>CATAAACCTCGCCCATCTGAATGATATCGGAACCTTAAATGCATTACAAAATTTTGGATTCTATTTAGGAATAGGCCTTACCAA<br>TATTCTAAATACTTTCAACCCACAAGCCGTAATTTTAAGAAATAGCATAATTGAATCGCATCCTATGGTTTTAAATTCAATGAG<br>AAGTGAAGTATCATCAAGGGTTTATTCCCAATTAGGCAATAGCTATGAATTATTGCCATCTTCCTTAGGACAGAATGCACCGGC<br>ATTAGGAATGTCCTCCATTGTGATTGATCATTTTCTGGACATGATTACAATGTAATTTTTTATGGAATGGACAGCTCATCTTTA<br>AAGATGAGTTTTTTTATTCTAGGAGTATTTCTGAAGCAATAGTGACATGGCACCTTCTCATATGAAAAAGGAGTTCTAAAATAG<br>AAATCTCCTTTTTCATGTGCAAATTATTTTTCTTTATAACGAAAATATCTAAAGTCGGCCAATTCACTGGCCGTCGTTTTACCG<br>AAATACGGGCAGACATGGCCTGCCCGGTTAATGCCTGGCTCCAAGTGTCATTATACAATTTTGCGCAACATTTTTTAGAAAGCA<br>TGCCTAACTGTTAAAAAAATATACCTAAGTGTTTTAATTAAGTACTATTAGATATTTTACCATATTTAGTTTTACAGTTGAGTA<br>CTAAATATTGCTATTTACGAAATTTTAATCTTTAAATGGAAAAATCATGTTTTAATAGACTCAGATCACAGAGATGTGGTCAGT<br>CAGTCTTAATTAACCAAGGAGGTGAAATGTACAATGAAAGGAGAAGAACTTTTCACTGGAGTTGTCCCAATTCTTGTTGAATTA<br>GATGGTGATGTTAATGGGCACAAATTTTCTGTCAGTGGAGAGGGTGAAGGTGATGCAACATACGGAAAACTTACCCTTAAATTT

```
ATTTGCACTACTGGAAAACTACCTGTTCCATGGCCAACACTTGTCACTACTTTCGCGTATGGTCTTCAATGCTTTGCGAGATAC
CCAGATCATATGAAACAGCATGACTTTTTCAAGAGTGCCATGCCCGAAGGTTATGTACAGGAAAGAACTATATTTTTCAAAGAT
GACGGGAACTACAAGACACGTGCTGAAGTCAAGTTTGAAGGTGATACCCTTGTTAATAGAATCGAGTTAAAAGGTATTGATTTT
AAAGAAGATGGAAACATTCTTGGACACAAATTGGAATACAACTATAACTCACACAATGTATACATCATGGCAGACAAACAAAAG
AATGGAATCAAAGTTAACTTCAAAATTAGACACAACATTGAAGATGGAAGCGTTCAACTAGCAGACCATTATCAACAAAATACT
CCAATTGGCGATGGCCCTGTCCTTTTACCAGACAACCATTACCTGTCCACACAATCTAAGCTTTCGAAAGATCCCAACGAAAAG
AGAGACCACATGGTCCTTCTTGAGTTTGTAACAGCTGCTGGGATTACACATGGCATGGATGAACTATACAAATAACCTAGGCTG
CTGCCACCGCTGAGCAATAACTAGCATAACCCCTTGGGGCCTCTAAACGGGTCTTGAGGGGTTTTTTGAACGTCGTGACTGGGA
AAACCCTGGCGTTACCCAACTTAATCGCCTTGCAGCACATCCCCCTTTCGCCAGCTGGCGTAATAGCGAAGAGGCCCGCACCGA
TCGCCCTTCCCAACAGTTGCGCAGCCTGAATGGCGAATGGCGCCTGATGCGGTATTTTCTCCTTACGCATCTGTGCGGTATTTC
ACACCGCATATGGTGCACTCTCAGTACAATCTGCTCTGATGCCGCATAGTTAAGCCAGCCCCGACACCCGCCAACACCCGCTGA
CGCGCCCTGACGGGCTTGTCTGTTCCCGGCATCCGCTTACAGACAAGCTGTGACCGTCTCCGGGAGCTGCATGTGTCAGAGGTT
TTCACCGTCATCACCGAAACGCGCGAGACGAAAGGGCCTCGTGATACGCCTATTTTTATAGGTTAATGTCATGATAATAATGGT
TTCTTAGACGTCAGGTGGCACTTTTCGGGGAAATGTGCGCGGAACCCCTATTTGTTTATTTTTCTAAATACATTCAAATATGTA
TCCGCTCATGAGACAATAACCCTGATAAATGCTTCAATAATCCATCCTCCAAAGTTGGAGAGTGAGTTTTATGTCGCAAATATT
AATGTTTCTGGTGAACCTTATCAAATTTTCGTTGATTTAATAGAAACATAGCGGTAAAATTAGCAGTAACTTAATAGAACGGAA
ATGAAAAAAGCCACTCTCATATGCTATTGGCTACCAACCTTTAGCGAGAATGACTTAATCCTGTACAGCCATACAGGACTTCGA
CTTATAAGAGGCGCCAACTTCAAATAAGTTATTTGCCTTGTTTTCGCGAACAAGGCTTATTAGATACACCTATTGTACCGTTAC
TCTACGAATATTTCAAGTAGTAATTACTAGCATTGTCCGTTACTCTACGAATATTTCAAGTAGTAATTACTAGCATTGTCCGTT
ACTCTACGAATATTTCAAGTAGTAATTACTAGCATTGTCATATACATAATAAAACGGATATAAAAGGGCGTTTTCTATACCTAG
AAGTCTTGTAAATGTACAGGGCGTTTAGATATAGAGAACGCCCTTTTTGTGTTCCGTTCCAGTGGAAGCTACCACTTTAAAAAG
ATGGTCTAGTGTAGCCAATGCAGGAGAGTACACTCGGATATCAGTTGTCGTTGCATTCAACTGTCTGACGTAAGCGAGGTAAAG
GACACAAGCCTTGCATAAAACAAGCCTACGGGATGTAAATCCTAATAATGATGATAACCAAGACGTTAGCGGCAAAAAGTGTTG
GGGGTTCAAAATAAGACATGATTGTGCGACTGGAGTTAAACAGTTACTCGTAAGCGGCGATCATGACACTGATTCACGGCTATT
CTTGTACAAGCTAGCTTTATTACAAGGATATGCGGGTTATATAGCGAATCACCCGAAAGGGAACGGTGTTGGGCGTGAGAAACG
CACCGTACGGCGCAATACAATGCCAATAAGCTATATACGGACGGTATAGTAGTTTTGTAAGCTATAACCGTTTGTCGTCAATGC
AACCAATCTCAATTCGAGACCTCGGCATCTAAGCCAGTACGAATGAGTGGGCGTTTTAACCTCGTAAATTTTCAACAGGGGTTA
CTATGCCCAAAACTACATTCAGATTTCCTAACAAACTCGCCAGTATGAAAACCTTAAGACCTTAAAGTCAAGGGATTTGAAGGA
TTTTAACCTCGATTAGCAAAAAATGTAGAGTACTGAAGCAACTACCATTAACTAAGATAGTGGGGGATTGAGGAAGAATCCAGA
GCTGTTTAAATCAAGTGAAAGACAAGATGAAATTAAAAGAATAGTGAAAGATAGGGGAGTGGTTCTCTATGAGAAAGGAAATGG
CTAGAGAACAAAGGCAGCGGTTTATTGATCTATTGTTAGACTTTATGGTAAAGAATCCTCATTTATTTGTTAATGGTACAGAGG
ATGAAAGTAATAATGTTGTTACAAAATGTAATAGTGATATTAAAGAGGTTGCGGAGTCATATTTAACTCTTTTATAGTGAGAGG
GTTAAAACTAATTAATATGTATTAAGGCCCAATGTTGGAATTATTGTATTTCACTAGGCAACCTACTTACTAAAAGTAAGATTA
TCCATTAGTGGATGTTATAATATTGGGTTTTTTAACACAATAATCATCGCCTTTCGGTGTCGTTTGATAGAAAAGTAACCATTA
GCGATGAAAAAGTCAATATAAAAAGCCATCCGTAAAAAACGGATGGCTTACCGTACATAGGATCGTTGGTAGGGCGGCGTATCC
TACATCTCTGGTAACTTACCTAGCCAATCAAATGCTTGAGAACGGCGGTTAGATAAGCGCGTGGGGAACCTTTCCCACCTCAAA
GATCCTATATCATTATTATGTTACTTTCTACAGGTAGTATACCATGTTCTTATATTTTAGTAAACTCCCCGTTAGCTTAACAGG
TCTTTGTAAGCAATTAAACGTCCACTATTCAATCGTCTTTGGATTTTCGCAGGACCGTTTTTTAGATCGAACATAGTTGATAAG
AACAAATAACCGCTTGGGTCCAACTTTATAGCAATTAGTATATGGTCATTTAAAATCTTTACCAATTCAACGCTATTAGGTTCT
TTAGGATTTTGCCCGACATAGTCGGGGTGTTCAACGATATCTTTTATGTGCGATGAATATTTTTCATAAATACCAGGATGTTGT
TTCTTTACGTGCTTTATAAATCCGGGAAACATTTTTACATCGTTAGAAGTGCAAGTCAAGTTATATGTATCTATAATGATTTGT
GGAAGTTTTGCCACAACAGTTGGTTTATTTACAATCTTTTTTTTATTAGCCGTCAAATTTCTCCCTCATCTCGTCTCTTTATAT
CTTTATTTTATCATAAAGGAGTATTTGAACCGTCGCGCGGGACAGGTTTATGATAGGGATATTTTATTGAATAATTGATGGTAT
AAGGGACTTTCATGCTTGGAAAGTGGGGATTATGAATTAGATGCTTGTCCACAATATGTTCCAATGTAATTAAAATTTATGTTC
CCACCTTGACCAAACATCACGTCCATACTTAAATCGTCCCTCCTTTAATAGGTAAAATATTAATTTACCTTAATAAAAAAATAA
TGGATAATAGTATTCGTCTGAATTTATATAATCAGGGGGAACTATTGATGCTGGGGATACTATTTACAGCGGCGCCATCTACTG
ATGTCGTAAAGGATTTGCAAGATAAAGTTATATCATTGCAGGATCATGAGGTAGCGTTTTTGAACACCACGATATCTAATATGT
TGATCCCCGAAGCAAACTTAAGAGTGTGTTGATAGTGCAGTATCTTAAAATTTTGTGTATAATAGGAATTGAAGTTAAATTAGA
TGCTAAAAATTTGTAATTAAGAAGGAGGGATTCGTCATGTTGGTATTCCAAATGCGTAATGTAGATAAAACATCTACTGTTTTG
AAACAGACTAAAAACAGTGATTACGCAGATAAATAAATACGTTAGATTAATTCCTACCAGTGACTAATCTTATGACTTTTTAAA
CAGATAACTAAAATTACAAACAAATCGTTTAACTTCTGTATTTATTTACAGATGTAATCACTTCAGGAGTAATTACATGAACAA
AAATATAAAATATTCTCAAAACTTTTTAACGAGTGAAAAAGTACTCAACCAAATAATAAAACAATTGAATTTAAAAGAAACCGA
TACCGTTTACGAAATTGGAACAGGTAAAGGGCATTTAACGACGAAACTGGCTAAAATAAGTAAACAGGTAACGTCTATTGAATT
AGACAGTCATCTATTCAACTTATCGTCAGAAAAATTAAAACTGAACATTCGTGTCACTTTAATTCACCAAGATATTCTACAGTT
TCAATTCCCTAACAAACAGAGGTATAAAATTGTTGGGAGTATTCCTTACCATTTAAGCACACAAATTATTAAAAAAGTGGTTTT
TGAAAGCCATGCGTCTGACATCTATCTGATTGTTGAAGAAGGATTCTACAAGCGTACCTTGGATATTCACCGAACACTAGGGTT
GCTCTTGCACACTCAAGTCTCGATTCAGCAATTGCTTAAGCTGCCAGCGGAATGCTTTCATCCTAAACCAAAAGTAAACAGTGT
CTTAATAAAACTTACCCGCCATACCACAGATGTTCCAGATAAATATTGGAAGCTATATACGTACTTTGTTTCAAAATGGGTCAA
TCGAGAATATCGTCAACTGTTTACTAAAAATCAGTTTCATCAAGCAATGAAACACGCCAAAGTAAACAATTTAAGTACCATTAC
TTATGAGCAAGTATTGTCTATTTTTAATAGTTATCTATTATTTAACGGGAGGAAATAATTCTATGAGTCGCTTTTTTAAATTTG
GAAAGTTACACGTTACTAAAGGGAATGGAGATAAATTATTAGATATACTACTGACAGCTTCCAAGAAGCTAAAGAGGTCCCTAG
CGCCTACGGGGAATTTGGGGTACATTGAAAAAGGAAGAGTATGAGTATTCAACATTTCCGTGTCGCCCTTATTCCCTTTTTTGC
GGCATTTTGCCTTCCTGTTTTTGCTCACCCAGAAACGCTGGTGAAAGTAAAAGATGCTGAAGATCAGTTGGGTGCACGAGTGGG
```

```
TTACATCGAACTGGATCTCAACAGCGGTAAGATCCTTGAGAGTTTTCGCCCCGAAGAACGTTTTCCAATGATGAGCACTTTTAA
AGTTCTGCTATGTGGCGCGGTATTATCCCGTATTGACGCCGGGCAAGAGCAACTCGGTCGCCGCATACACTATTCTCAGAATGA
CTTGGTTGAGTACTCACCAGTCACAGAAAAGCATCTTACGGATGGCATGACAGTAAGAGAATTATGCAGTGCTGCCATAACCAT
GAGTGATAACACTGCGGCCAACTTACTTCTGACAACGATCGGAGGACCGAAGGAGCTAACCGCTTTTTTGCACAACATGGGGGA
TCATGTAACTCGCCTTGATCGTTGGGAACCGGAGCTGAATGAAGCCATACCAAACGACGAGCGTGACACCACGATGCCTGTAGC
AATGGCAACAACGTTGCGCAAACTATTAACTGGCGAACTACTTACTCTAGCTTCCCGGCAACAATTAATAGACTGGATGGAGGC
GGATAAAGTTGCAGGACCACTTCTGCGCTCGGCCCTTCCGGCTGGCTGGTTTATTGCTGATAAATCTGGAGCCGGTGAGCGTGG
GTCTCGCGGTATCATTGCAGCACTGGGGCCAGATGGTAAGCCCTCCCGTATCGTAGTTATCTACACGACGGGGAGTCAGGCAAC
TATGGATGAACGAAATAGACAGATCGCTGAGATAGGTGCCTCACTGATTAAGCATTGGTAACTGTCAGACCAAGTTTACTCATA
TATACTTTAGATTGATTTAAAACTTCATTTTTAATTTAAAAGGATCTAGGTGAAGATCCTTTTTGATAATCTCATGACCAAAAT
CCCTTAACGTGAGTTTTCGTTCCACTGAGCGTCAGACCCCGTAGAAAAGATCAAAGGATCTTC-3’
```

pXyl-Se-agrBDCA-GFP

```
5’-
TTGAGATCCTTTTTTTCTGCGCGTAATCTGCTGCTTGCAAACAAAAAAACCACCGCTACCAGCGGTGGTTTGTTTGCCGGATCA
AGAGCTACCAACTCTTTTTCCGAAGGTAACTGGCTTCAGCAGAGCGCAGATACCAAATACTGTTCTTCTAGTGTAGCCGTAGTT
AGGCCACCACTTCAAGAACTCTGTAGCACCGCCTACATACCTCGCTCTGCTAATCCTGTTACCAGTGGCTGCTGCCAGTGGCGA
TAAGTCGTGTCTTACCGGGTTGGACTCAAGACGATAGTTACCGGATAAGGCGCAGCGGTCGGGCTGAACGGGGGGTTCGTGCAC
ACAGCCCAGCTTGGAGCGAACGACCTACACCGAACTGAGATACCTACAGCGTGAGCTATGAGAAAGCGCCACGCTTCCCGAAGG
GAGAAAGGCGGACAGGTATCCGGTAAGCGGCAGGGTCGGAACAGGAGAGCGCACGAGGGAGCTTCCAGGGGGAAACGCCTGGTA
TCTTTATAGTCCTGTCGGGTTTCGCCACCTCTGACTTGAGCGTCGATTTTTGTGATGCTCGTCAGGGGGGCGGAGCCTATGGAA
AAACGCCAGCAACGCGGCCTTTTTACGGTTCCTGGCCTTTTGCTGGCCTTTTGCTCACATGTTCTTTCCTGCGTTATCCCCTGA
TTCTGTGGATAACCGTATTACCGCCTTTGAGTGAGCTGATACCGCTCGCCGCAGCCGAACGACCGAGCGCAGCGAGTCAGTGAG
CGAGGAAGCGGAAGAGCGCCCAATACGCAAACCGCCTCTCCCCGCGCGTTGGCCGATTCATTAATGCAGCTGGCACGACAGGTT
TCCCGACTGGAAAGCGGGCAGTGAGCGCAACGCAATTAATGTGAGTTAGCTCACTCATTAGGCACCCCAGGCTTTACACTTTAT
GCTTCCGGCTCGTATGTTGTGTGGAATTGTGAGCGGATAACAATTTCACACAGGAAACAGCTATGACCATGATTACGCCAAGCT
TGCATGCCTGCATTATATTTTTTTAACATTACGTACTGAAGCGAAACAATTTTCGCCATTCTTAAAGTAAACAATACGTTCTTT
TGAGTCAATAGATTCAATATTATGCCTGTTTATTACAAAACTGTTATGACATCTAAAGAAACGTTCATCAAGCTGTGCTAATTC
CTTTAAATTTCCATAAAATTCAATTTGTCGATTATCAAGATGTGCAATGAGTCTATGAGATTTCGTAGATGATTCAAAAAACAT
AATATCATCATATTGAACGTATACTGAATTACTTCCCCGCTTTAACTCAATCGTATCTACATTACTTTCTTTTGATAATAATTT
GAGTCGTGTATGTGCTGTTTCAAGACAATCTATGATTCTCATTTTTAATTCAGATGGATCATCTTTAAAAATAAAATCCATAGC
AGCCACTTTATAAACAAACGTCAAATAAGTCAGCTCACTGTGACTGGTTACAAATATAATATTACCAACAGGATCATGTTTACG
AATTTCACTGGCTAATTTAATACCGTTCATATCAGCTTCTAATTGAATATCAAGGAAGTAACAACCAATGTCATTAAGTTCTTT
TGATTGCTCTAAGACCTCATAAGGATCATTTGTTGCTAAAGCTAACTCCATTGGCTTTTCTTCAATCATTATGTAGTTTTTAAT
GATTGATACCATATGTTCTCTTTGTCTTTGGTCATCTTCACAAACAAAAATTTTCATTTAATTCTACACATCCTTATGATTCCT
TATTATTTATTTCTACTTTTTGTACAAAGTAACCATTTTCGATGACAGTATCTAATAAAACATTCTCGTTTGAGTCTGTCAGTT
CTTTTAAAGTTGATAAACCTAAACCGCGATTATCACCTTTAGTAGAAAAACCTTGTTCAAACAACTCATGAATTTTAGGGATAT
CATTACTACATTTATTCATAACGATAAAAGTGACAGATTCCTCATTATCGATGAATGCGATATTGATGAGTGGTTCCTCAAGAT
TTTCTGAAGCTTCAATAGCATTATCAACTATAATACCGATAATACGACTTAGCTCAACAGTATTCATATCGATACGATCAATTT
CATCAGGAACCTCAATACTAATTGGAATACGTTTTTCTTGAGCTTGAATAATTTTAGTAGTAATCAGCCCTTTAATTTCTCTCA
CTTTCAACTTTTCAATACCATTCATTTTAATAGAGCGAGTTTTTAATTTATCTTTCATTGGAACGATATTTTCATTAAAATATT
TACGTAATCCAGGCATATCATCTTCTCTAATGTAATCTGAAAGAGTGGTGAGGATATTCACATAATCATGTCGGAACTTACGCA
TTTCATTGTTAATGCTTTCTATACGTAACGTGTACTCATAATATGCTTCGATTTCTTTTACATTACGTTTATACCTCATTTCAC
GGAGTGTAAAATTGGACATCACTAATATCACTACACTTAAAAAAACCATAATACCCAACAATAAGATGGCATATAATTTAAGGG
TATCATTTCCTCGCATATCAGTTTGTGACACCATATAAAGTAAAATAAATGATATGAAAAGTACTATTGTTATTATCAATAAGT
ATCTTTTATTGAGTGACAAGTAGGATACTTTTAATTTATTGAACAATAGTTGAGTTAAATAAGCAATAATTAGAGTTATGATTA
CAAAAGAGGTAAAATGTATTAACTGTAAAGCAAATTTAAACGGAATATAATCTTTTATAGTCAAATGTATGTATACAGTTATGA
AATTAGTTATATATATAAGATCATAGTGGTGAATAATACAACTAATATTGAATAAAGCTTTATTTTTGTATAAAAGAAAATGGTGA
TTATTATAACCAAAACTATTAATGCTTTACTTTGCCAAAAGTAATACATTATTGCAGAAGGGATTACAATCGTAAAAACGATTA
TGTAATCCCTAAAATTAAATTTCATATTAATGATAACTTTAGTAACCCAAATCATTAAAAAGATTTGTAGGCCTGCAAACGGAA
ATAGATTAATATCATCCATATCTTACACACTTTCTAGGGTTATATTTACTCGTATAGTTTAGTCAGTTCTTCTGGTACTTCTGG
TTCGTCAAAGTAAGAGGCACATACACTATCTCCTGCTACAGTACCAATAAATTCCAAGATTGTAGTGAAAAATTTTATAAATAA
ATTAAAAATGTTTTCCATGATTAATATCCTCCTTAGGGAAAAAGATGGGTAGTAATGTTAAAGATTCTAAAATTACACCGAATA
AAATAAGTTTATTTACCGGTTCTTTAGTTACTAATGAAATAACTACGATAGTACAATATAAAAATATGGAGAGTATTTTTTTTC
GCTTTACAAGACGTCTAGGTATAGGTTGTTTCTTAGTTGCTGCAGGTGCGTATAAAATGGTAATAATTAATCCGACTAATGCCA
TAGATAAAAGAACAAAATAGTTAATATCTAACTTTATTATTAAGTATGGAAAGATAATAAAGAAAATTATGTTCTGAATATGAC
ATAACAATGACGAATTTGCATGCGTACCGTGTGCATGTCTCCTAATTAAAAAATAACTTAAATGAGTTAAAAGTGTGTAAAAGA
AAGTATGAAAGATTATTGCTAGCCCATACACAACTATAGACTTTTCAATATTTATCGCTAGTACCTGCATCCCTAAACGAATTT
TTAGAAACTGTATGTGATCTAAGTTATTTTTACGTTGTAAATATTGAGCAAATTGCTCAATTTTTTTATCGATGATTTTCATAG
TAGTTCCTCCTTATCTACAGATGCATTTTATTTCATATAGTAAGTACATCACCTATTAGTTTGTTGTTTAAACAAACTAACTTA
TTTTCATCTTATATAACCTCGTCAGTATTTTCAATATTTTTTTTAGTTTTTTATGAACACATTAGATATAATAAAGGGAAGATT
CGCTATGTACTATGTTGATACTTAATTTAAAGATTAAACAAATGGAGTGGATGAAGTGGATATCGCTGATCAAACCTTTGTCAA
```

```
AAAAGTAAATCAAAAGTTATTATTAAAAGAAATCCTTAAAAATTCACCTATTTCAAGAGCAAAATTATCTGAAATGACTGGATT
AAATAAATCAACTGTCTCATCACAGGTAAACACGTTAATGAAAGAAAGTATGGTATTTGAAATAGGTCAAGGACAATCAAGTGG
CGGAAGAAGACCTGTCATGCTTGTTTTTAATAAAAAGGCAGGATACTCCGTTGGAATAGATGTTGGTGTGGATTATATTAATGG
CATTTTAACAGACCTTGAAGGAACAATCGTTCTTGATCAATACCGCCATTTGGAATCCAATTCTCCAGAAATAACGAAAGACAT
TTTGATTGATATGATTCATCACTTTATTACGCAAATGCCCCAATCTCCGTACGGGCTTATTGGTATAGGTATTTGCGTGCCTGG
ACTCATTGATAAAGATCAAAAAATTGTTTTCACTCCGAACTCCAACTGGAGAGATATTGACTTAAAATCTTCGATACAAGAGAA
GTACAATGTGCCTGTTTTTATTGAAAATGAGGCAAATGCTGGCGCATATGGAGAAAAAGTATTTGGAGCTGCAAAAAATCACGA
TAACATTATTTACGTAAGTATCAGCACAGGAATAGGGATCGGTGTTATTATCAACAATCATTTATATAGAGGAGTAAGCGGCTT
CTCTGGAGAAATGGGACATATGACAATAGACTTTAATGGTCCTAAATGCAGTTGCGGAAACCGAGGATGCTGGGAATTGTATGC
TTCAGAGAAGGCTTTATTAAAATCTCTTCAGACCAAAGAGAAAAAACTGTCCTATCAAGATATCATAAACCTCGCCCATCTGAA
TGATATCGGAACCTTAAATGCATTACAAAATTTTGGATTCTATTTAGGAATAGGCCTTACCAATATTCTAAATACTTTCAACCC
ACAAGCCGTAATTTTAAGAAATAGCATAATTGAATCGCATCCTATGGTTTTAAATTCAATGAGAAGTGAAGTATCATCAAGGGT
TTATTCCCAATTAGGCAATAGCTATGAATTATTGCCATCTTCCTTAGGACAGAATGCACCGGCATTAGGAATGTCCTCCATTGT
GATTGATCATTTTCTGGACATGATTACAATGTAATTTTTTATGGAATGGACAGCTCATCTTTAAAGATGAGTTTTTTTATTCTA
GGAGTATTTCTGAAGCAATAGTGACATGGCACCTTCTCATATGAAAAAGGAGTTCTAAAATAGAAATCTCCTTTTTCATGTGCA
AATTATTTTTCTTTATAACGAAAATATCTAAAGTCGGCCAATTCACTGGCCGTCGTTTTACCGAAATACGGGCAGACATGGCCT
GCCCGGTTAATGCCTGGCTCCAAGTGTCATTATACAATTTTGCGCAACATTTTTTAGAAAGCATGCCTAACTGTTAAAAAAATA
TACCTAAGTGTTTTAATTAAGTACTATTAGATATTTTACCATATTTAGTTTTACAGTTGAGTACTAAATATTGCTATTTACGAA
ATTTTAATCTTTAAATGGAAAAATCATGTTTTAATAGACTCAGATCACAGAGATGTGGTCAGTCAGTCTTAATTAACCAAGGAG
GTGAAATGTACAATGAAAGGAGAAGAACTTTTCACTGGAGTTGTCCCAATTCTTGTTGAATTAGATGGTGATGTTAATGGGCAC
AAATTTTCTGTCAGTGGAGAGGGTGAAGGTGATGCAACATACGGAAAACTTACCCTTAAATTTATTTGCACTACTGGAAAACTA
CCTGTTCCATGGCCAACACTTGTCACTACTTTCGCGTATGGTCTTCAATGCTTTGCGAGATACCCAGATCATATGAAACAGCAT
GACTTTTTCAAGAGTGCCATGCCCGAAGGTTATGTACAGGAAAGAACTATATTTTTCAAAGATGACGGGAACTACAAGACACGT
GCTGAAGTCAAGTTTGAAGGTGATACCCTTGTTAATAGAATCGAGTTAAAAGGTATTGATTTTAAAGAAGATGGAAACATTCTT
GGACACAAATTGGAATACAACTATAACTCACACAATGTATACATCATGGCAGACAAACAAAAGAATGGAATCAAAGTTAACTTC
AAAATTAGACACAACATTGAAGATGGAAGCGTTCAACTAGCAGACCATTATCAACAAAATACTCCAATTGGCGATGGCCCTGTC
CTTTTACCAGACAACCATTACCTGTCCACACAATCTAAGCTTTCGAAAGATCCCAACGAAAAGAGAGACCACATGGTCCTTCTT
GAGTTTGTAACAGCTGCTGGGATTACACATGGCATGGATGAACTATACAAATAACCTAGGCTGCTGCCACCGCTGAGCAATAAC
TAGCATAACCCCTTGGGGCCTCTAAACGGGTCTTGAGGGGTTTTTTGAACGTCGTGACTGGGAAAACCCTGGCGTTACCCAACT
TAATCGCCTTGCAGCACATCCCCCTTTCGCCAGCTGGCGTAATAGCGAAGAGGCCCGCACCGATCGCCCTTCCCAACAGTTGCG
CAGCCTGAATGGCGAATGGCGCCTGATGCGGTATTTTCTCCTTACGCATCTGTGCGGTATTTCACACCGCATATGGTGCACTCT
CAGTACAATCTGCTCTGATGCCGCATAGTTAAGCCAGCCCCGACACCCGCCAACACCCGCTGACGCGCCCTGACGGGCTTGTCT
GTTCCCGGCATCCGCTTACAGACAAGCTGTGACCGTCTCCGGGAGCTGCATGTGTCAGAGGTTTTCACCGTCATCACCGAAACG
CGCGAGACGAAAGGGCCTCGTGATACGCCTATTTTTATAGGTTAATGTCATGATAATAATGGTTTCTTAGACGTCAGGTGGCAC
TTTTCGGGGAAATGTGCGCGGAACCCCTATTTGTTTATTTTTCTAAATACATTCAAATATGTATCCGCTCATGAGACAATAACC
CTGATAAATGCTTCAATAATCCATCCTCCAAAGTTGGAGAGTGAGTTTTATGTCGCAAATATTAATGTTTCTGGTGAACCTTAT
CAAATTTTCGTTGATTTAATAGAAACATAGCGGTAAAATTAGCAGTAACTTAATAGAACGGAAATGAAAAAAGCCACTCTCATA
TGCTATTGGCTACCAACCTTTAGCGAGAATGACTTAATCCTGTACAGCCATACAGGACTTCGACTTATAAGAGGCGCCAACTTC
AAATAAGTTATTTGCCTTGTTTTCGCGAACAAGGCTTATTAGATACACCTATTGTACCGTTACTCTACGAATATTTCAAGTAGT
AATTACTAGCATTGTCCGTTACTCTACGAATATTTCAAGTAGTAATTACTAGCATTGTCCGTTACTCTACGAATATTTCAAGTA
GTAATTACTAGCATTGTCATATACATAATAAAACGGATATAAAAGGGCGTTTTCTATACCTAGAAGTCTTGTAAATGTACAGGG
CGTTTAGATATAGAGAACGCCCTTTTTGTGTTCCGTTCCAGTGGAAGCTACCACTTTAAAAAGATGGTCTAGTGTAGCCAATGC
AGGAGAGTACACTCGGATATCAGTTGTCGTTGCATTCAACTGTCTGACGTAAGCGAGGTAAAGGACACAAGCCTTGCATAAAAC
AAGCCTACGGGATGTAAATCCTAATAATGATGATAACCAAGACGTTAGCGGCAAAAAGTGTTGGGGGTTCAAAATAAGACATGA
TTGTGCGACTGGAGTTAAACAGTTACTCGTAAGCGGCGATCATGACACTGATTCACGGCTATTCTTGTACAAGCTAGCTTTATT
ACAAGGATATGCGGGTTATATAGCGAATCACCCGAAAGGGAACGGTGTTGGGCGTGAGAAACGCACCGTACGGCGCAATACAAT
GCCAATAAGCTATATACGGACGGTATAGTAGTTTTGTAAGCTATAACCGTTTGTCGTCAATGCAACCAATCTCAATTCGAGACC
TCGGCATCTAAGCCAGTACGAATGAGTGGGCGTTTTAACCTCGTAAATTTTCAACAGGGGTTACTATGCCCAAAACTACATTCA
GATTTCCTAACAAACTCGCCAGTATGAAAACCTTAAGACCTTAAAGTCAAGGGATTTGAAGGATTTTAACCTCGATTAGCAAAA
AATGTAGAGTACTGAAGCAACTACCATTAACTAAGATAGTGGGGGATTGAGGAAGAATCCAGAGCTGTTTAAATCAAGTGAAAG
ACAAGATGAAATTAAAAGAATAGTGAAAGATAGGGGAGTGGTTCTCTATGAGAAAGGAAATGGCTAGAGAACAAAGGCAGCGGT
TTATTGATCTATTGTTAGACTTTATGGTAAAGAATCCTCATTTATTTGTTAATGGTACAGAGGATGAAAGTAATAATGTTGTTA
CAAAATGTAATAGTGATATTAAAGAGGTTGCGGAGTCATATTTAACTCTTTTATAGTGAGAGGGTTAAAACTAATTAATATGTA
TTAAGGCCCAATGTTGGAATTATTGTATTTCACTAGGCAACCTACTTACTAAAAGTAAGATTATCCATTAGTGGATGTTATAAT
ATTGGGTTTTTTAACACAATAATCATCGCCTTTCGGTGTCGTTTGATAGAAAAGTAACCATTAGCGATGAAAAAGTCAATATAA
AAAGCCATCCGTAAAAAACGGATGGCTTACCGTACATAGGATCGTTGGTAGGGCGGCGTATCCTACATCTCTGGTAACTTACCT
AGCCAATCAAATGCTTGAGAACGGCGGTTAGATAAGCGCGTGGGGAACCTTTCCCACCTCAAAGATCCTATATCATTATTATGT
TACTTTCTACAGGTAGTATACCATGTTCTTATATTTTAGTAAACTCCCCGTTAGCTTAACAGGTCTTTGTAAGCAATTAAACGT
CCACTATTCAATCGTCTTTGGATTTTCGCAGGACCGTTTTTTAGATCGAACATAGTTGATAAGAACAAATAACCGCTTGGGTCC
AACTTTATAGCAATTAGTATATGGTCATTTAAAATCTTTACCAATTCAACGCTATTAGGTTCTTTAGGATTTTGCCCGACATAG
TCGGGGTGTTCAACGATATCTTTTATGTGCGATGAATATTTTTCATAAATACCAGGATGTTGTTTCTTTACGTGCTTTATAAAT
CCGGGAAACATTTTTACATCGTTAGAAGTGCAAGTCAAGTTATATGTATCTATAATGATTTGTGGAAGTTTTGCCACAACAGTT
GGTTTATTTACAATCTTTTTTTTATTAGCCGTCAAATTTCTCCCTCATCTCGTCTCTTTATATCTTTATTTTATCATAAAGGAG
```

```
TATTTGAACCGTCGCGCGGGACAGGTTTATGATAGGGATATTTTATTGAATAATTGATGGTATAAGGGACTTTCATGCTTGGAA
AGTGGGGATTATGAATTAGATGCTTGTCCACAATATGTTCCAATGTAATTAAAATTTATGTTCCCACCTTGACCAAACATCACG
TCCATACTTAAATCGTCCCTCCTTTAATAGGTAAAATATTAATTTACCTTAATAAAAAAATAATGGATAATAGTATTCGTCTGA
ATTTATATAATCAGGGGGAACTATTGATGCTGGGGATACTATTTACAGCGGCGCCATCTACTGATGTCGTAAAGGATTTGCAAG
ATAAAGTTATATCATTGCAGGATCATGAGGTAGCGTTTTTGAACACCACGATATCTAATATGTTGATCCCCGAAGCAAACTTAA
GAGTGTGTTGATAGTGCAGTATCTTAAAATTTTGTGTATAATAGGAATTGAAGTTAAATTAGATGCTAAAAATTTGTAATTAAG
AAGGAGGGATTCGTCATGTTGGTATTCCAAATGCGTAATGTAGATAAAACATCTACTGTTTTGAAACAGACTAAAAACAGTGAT
TACGCAGATAAATAAATACGTTAGATTAATTCCTACCAGTGACTAATCTTATGACTTTTTAAACAGATAACTAAAATTACAAAC
AAATCGTTTAACTTCTGTATTTATTTACAGATGTAATCACTTCAGGAGTAATTACATGAACAAAAATATAAAATATTCTCAAAA
CTTTTTAACGAGTGAAAAAGTACTCAACCAAATAATAAAACAATTGAATTTAAAAGAAACCGATACCGTTTACGAAATTGGAAC
AGGTAAAGGGCATTTAACGACGAAACTGGCTAAAATAAGTAAACAGGTAACGTCTATTGAATTAGACAGTCATCTATTCAACTT
ATCGTCAGAAAAATTAAAACTGAACATTCGTGTCACTTTAATTCACCAAGATATTCTACAGTTTCAATTCCCTAACAAACAGAG
GTATAAAATTGTTGGGAGTATTCCTTACCATTTAAGCACACAAATTATTAAAAAAGTGGTTTTTGAAAGCCATGCGTCTGACAT
CTATCTGATTGTTGAAGAAGGATTCTACAAGCGTACCTTGGATATTCACCGAACACTAGGGTTGCTCTTGCACACTCAAGTCTC
GATTCAGCAATTGCTTAAGCTGCCAGCGGAATGCTTTCATCCTAAACCAAAAGTAAACAGTGTCTTAATAAAACTTACCCGCCA
TACCACAGATGTTCCAGATAAATATTGGAAGCTATATACGTACTTTGTTTCAAAATGGGTCAATCGAGAATATCGTCAACTGTT
TACTAAAAATCAGTTTCATCAAGCAATGAAACACGCCAAAGTAAACAATTTAAGTACCATTACTTATGAGCAAGTATTGTCTAT
TTTTAATAGTTATCTATTATTTAACGGGAGGAAATAATTCTATGAGTCGCTTTTTTAAATTTGGAAAGTTACACGTTACTAAAG
GGAATGGAGATAAATTATTAGATATACTACTGACAGCTTCCAAGAAGCTAAAGAGGTCCCTAGCGCCTACGGGGAATTTGGGGT
ACATTGAAAAAGGAAGAGTATGAGTATTCAACATTTCCGTGTCGCCCTTATTCCCTTTTTTGCGGCATTTTGCCTTCCTGTTTT
TGCTCACCCAGAAACGCTGGTGAAAGTAAAAGATGCTGAAGATCAGTTGGGTGCACGAGTGGGTTACATCGAACTGGATCTCAA
CAGCGGTAAGATCCTTGAGAGTTTTCGCCCCGAAGAACGTTTTCCAATGATGAGCACTTTTAAAGTTCTGCTATGTGGCGCGGT
ATTATCCCGTATTGACGCCGGGCAAGAGCAACTCGGTCGCCGCATACACTATTCTCAGAATGACTTGGTTGAGTACTCACCAGT
CACAGAAAAGCATCTTACGGATGGCATGACAGTAAGAGAATTATGCAGTGCTGCCATAACCATGAGTGATAACACTGCGGCCAA
CTTACTTCTGACAACGATCGGAGGACCGAAGGAGCTAACCGCTTTTTTGCACAACATGGGGGATCATGTAACTCGCCTTGATCG
TTGGGAACCGGAGCTGAATGAAGCCATACCAAACGACGAGCGTGACACCACGATGCCTGTAGCAATGGCAACAACGTTGCGCAA
ACTATTAACTGGCGAACTACTTACTCTAGCTTCCCGGCAACAATTAATAGACTGGATGGAGGCGGATAAAGTTGCAGGACCACT
TCTGCGCTCGGCCCTTCCGGCTGGCTGGTTTATTGCTGATAAATCTGGAGCCGGTGAGCGTGGGTCTCGCGGTATCATTGCAGC
ACTGGGGCCAGATGGTAAGCCCTCCCGTATCGTAGTTATCTACACGACGGGGAGTCAGGCAACTATGGATGAACGAAATAGACA
GATCGCTGAGATAGGTGCCTCACTGATTAAGCATTGGTAACTGTCAGACCAAGTTTACTCATATATACTTTAGATTGATTTAAA
ACTTCATTTTTAATTTAAAAGGATCTAGGTGAAGATCCTTTTTGATAATCTCATGACCAAAATCCCTTAACGTGAGTTTTCGTT
CCACTGAGCGTCAGACCCCGTAGAAAAGATCAAAGGATCTTC-3’
```

pSe-control-GFP

```
5’-
TTGAGATCCTTTTTTTCTGCGCGTAATCTGCTGCTTGCAAACAAAAAAACCACCGCTACCAGCGGTGGTTTGTTTGCCGGATCA
AGAGCTACCAACTCTTTTTCCGAAGGTAACTGGCTTCAGCAGAGCGCAGATACCAAATACTGTTCTTCTAGTGTAGCCGTAGTT
AGGCCACCACTTCAAGAACTCTGTAGCACCGCCTACATACCTCGCTCTGCTAATCCTGTTACCAGTGGCTGCTGCCAGTGGCGA
TAAGTCGTGTCTTACCGGGTTGGACTCAAGACGATAGTTACCGGATAAGGCGCAGCGGTCGGGCTGAACGGGGGGTTCGTGCAC
ACAGCCCAGCTTGGAGCGAACGACCTACACCGAACTGAGATACCTACAGCGTGAGCTATGAGAAAGCGCCACGCTTCCCGAAGG
GAGAAAGGCGGACAGGTATCCGGTAAGCGGCAGGGTCGGAACAGGAGAGCGCACGAGGGAGCTTCCAGGAGGAAACGCCTGGTA
TCTTTATAGTCCTGTCGGGTTTCGCCACCTCTGACTTGAGCGTCGATTTTTGTGATGCTCGTCAGGGGGGCGGAGCCTATGGAA
AAACGCCAGCAACGCGGCCTTTTTACGGTTCCTGGCCTTTTGCTGGCCTTTTGCTCACATGTTCTTTCCTGCGTTATCCCCTGA
TTCTGTGGATAACCGTATTACCGCCTTTGAGTGAGCTGATACCGCTCGCCGCAGCCGAACGACCGAGCGCAGCGAGTCAGTGAG
CGAGGAAGCGGAAGAGCGCCCAATACGCAAACCGCCTCTCCCCGCGCGTTGGCCGATTCATTAATGCAGCTGGCACGACAGGTT
TCCCGACTGGAAAGCGGGCAGTGAGCGCAACGCAATTAATGTGAGTTAGCTCACTCATTAGGCACCCCAGGCTTTACACTTTAT
GCTTCCGGCTCGTATGTTGTGTGGAATTGTGAGCGGATAACAATTTCACACAGGAAACAGCTATGACCATGATTACGCCAAGCT
TGCATGCCTGCCAGATGCATTTTATTTCATATAGTAAGTACATCACCTATTAGTTTGTTGTTTAAACAAACTAACTTATTTTCA
TCTTATATAACCTCGTCAGTATTTTCAATATTTTTTTTAGTTTTTTATGAACACATTAGATATAATAAAGGGAAGATTCGCTAT
GTACTATGTTGATACTTAATTTAAAGATTAAACAAATGGAGTGGATGAAGTGGATATCGCTGATCAAACCTTTGTCAAAAAAGT
AAATCAAAAGTTATTATTAAAAGAAATCCTTAAAAATTCACCTATTTCAAGAGCAAAATTATCTGAAATGACTGGATTAAATAA
ATCAACTGTCTCATCACAGGTAAACACGTTAATGAAAGAAAGTATGGTATTTGAAATAGGTCAAGGACAATCAAGTGGCGGAAG
AAGACCTGTCATGCTTGTTTTTAATAAAAAGGCAGGATACTCCGTTGGAATAGATGTTGGTGTGGATTATATTAATGGCATTTT
AACAGACCTTGAAGGAACAATCGTTCTTGATCAATACCGCCATTTGGAATCCAATTCTCCAGAAATAACGAAAGACATTTTGAT
TGATATGATTCATCACTTTATTACGCAAATGCCCCAATCTCCGTACGGGCTTATTGGTATAGGTATTTGCGTGCCTGGACTCAT
TGATAAAGATCAAAAAATTGTTTTCACTCCGAACTCCAACTGGAGAGATATTGACTTAAAATCTTCGATACAAGAGAAGTACAA
TGTGCCTGTTTTTATTGAAAATGAGGCAAATGCTGGCGCATATGGAGAAAAAGTATTTGGAGCTGCAAAAAATCACGATAACAT
TATTTACGTAAGTATCAGCACAGGAATAGGGATCGGTGTTATTATCAACAATCATTTATATAGAGGAGTAAGCGGCTTCTCTGG
AGAAATGGGACATATGACAATAGACTTTAATGGTCCTAAATGCAGTTGCGGAAACCGAGGATGCTGGGAATTGTATGCTTCAGA
GAAGGCTTTATTAAAATCTCTTCAGACCAAAGAGAAAAAACTGTCCTATCAAGATATCATAAACCTCGCCCATCTGAATGATAT
CGGAACCTTAAATGCATTACAAAATTTTGGATTCTATTTAGGAATAGGCCTTACCAATATTCTAAATACTTTCAACCCACAAGC
CGTAATTTTAAGAAATAGCATAATTGAATCGCATCCTATGGTTTTAAATTCAATGAGAAGTGAAGTATCATCAAGGGTTTATTC
```

```
CCAATTAGGCAATAGCTATGAATTATTGCCATCTTCCTTAGGACAGAATGCACCGGCATTAGGAATGTCCTCCATTGTGATTGA
TCATTTTCTGGACATGATTACAATGTAATTTTTTATGGAATGGACAGCTCATCTTTAAAGATGAGTTTTTTTATTCTAGGAGTA
TTTCTGAAGCAATAGTGACATGGCACCTTCTCACATGAAAAAGGAGTTCTAAAATAGAAATCTCCTTTTTCATGTGCAAATTAT
TTTTCTTTATAACGAAAATATCTAAAGTCGGCCAATTCACTGGCCGTCGTTTTACCGAAATACGGGCAGACATGGCCTGCCTGG
TTAATGCCTGGCTTCAAGTGTCATTATACAATTTTGCGCAACATTTTTTAGAAAGCATGCCTAACTGTTAAAAAAATATACCTA
AGTGTTTTAATTAAGTACTATTAGATATTTTACCATATTTAGTTTTACAGTTGAGTACTAAATATTGCTATTTACGAAATTTTA
ATCTTTAAATGGAAAAATCATGTTTTAATAGACTCAGATCACAGAGATGTGGTCAGTCAGTCTTAATTAACCAAGGAGGTGAAA
TGTACAATGAAAGGAGAAGAACTTTTCACTGGAGTTGTCCCAATTCTTGTTGAATTAGATGGTGATGTTAATGGGCACAAATTT
TCTGTCAGTGGAGAGGGTGAAGGTGATGCAACATACGGAAAACTTACCCTTAAATTTATTTGCACTACTGGAAAACTACCTGTT
CCATGGCCAACACTTGTCACTACTTTCGCGTATGGTCTTCAATGCTTTGCGAGATACCCAGATCATATGAAACAGCATGACTTT
TTCAAGAGTGCCATGCCCGAAGGTTATGTACAGGAAAGAACTATATTTTTCAAAGATGACGGGAACTACAAGACACGTGCTGAA
GTCAAGTTTGAAGGTGATACCCTTGTTAATAGAATCGAGTTAAAAGGTATTGATTTTAAAGAAGATGGAAACATTCTTGGACAC
AAATTGGAATACAACTATAACTCACACAATGTATACATCATGGCAGACAAACAAAAGAATGGAATCAAAGTTAACTTCAAAATT
AGACACAACATTGAAGATGGAAGCGTTCAACTAGCAGACCATTATCAACAAAATACTCCAATTGGCGATGGCCCTGTCCTTTTA
CCAGACAACCATTACCTGTCCACACAATCTAAGCTTTCGAAAGATCCCAACGAAAAGAGAGACCACATGGTCCTTCTTGAGTTT
GTAACAGCTGCTGGGATTACACATGGCATGGATGAACTATACAAATAACCTAGGCTGCTGCCACCGCTGAGCAATAACTAGCAT
AACCCCTTGGGGCCTCTAAACGGGTCTTGAGGGGTTTTTTGAACGTCGTGACTGGGAAAACCCTGGCGTTACCCAACTTAATCG
CCTTGCAGCACATCCCCCTTTCGCCAGCTGGCGTAATAGCGAAGAGGCCCGCACCGATCGCCCTTCCCAACAGTTGCGCAGCCT
GAATGGCGAATGGCGCCTGATGCGGTATTTTCTCCTTACGCATCTGTGCGGTATTTCACACCGCATATGGTGCACTCTCAGTAC
AATCTGCTCTGATGCCGCATAGTTAAGCCAGCCCCGACACCCGCCAACACCCGCTGACGCGCCCTGACGGGCTTGTCTGCTCCC
GGCATCCGCTTACAGACAAGCTGTGACCGTCTCCGGGAGCTGCATGTGTCAGAGGTTTTCACCGTCATCACCGAAACGCGCGAG
ACGAAAGGGCCTCGTGATACGCCTATTTTTATAGGTTAATGTCATGATAATAATGGTTTCTTAGACGTCAGGTGGCACTTTTCG
GGGAAATGTGCGCGGAACCCCTATTTGTTTATTTTTCTAAATACATTCAAATATGTATCCGCTCATGAGACAATAACCCTGATA
AATGCTTCAATAATCCATCCTCCAAAGTTGGAGAGTGAGTTTTATGTCGCAAATATTAATGTTTCTGGTGAACCTTATCAAATT
TTCGTTGATTTAATAGAAACATAGCGGTAAAATTAGCAGTAACTTAATAGAACGGAAATGAAAAAAGCCACTCTCATATGCTAT
TGGCTACCAACCTTTAGCGAGAATGACTTAATCCTGTACAGCCATACAGGACTTCGACTTATAAGAGGCGCCAACTTCAAATAA
GTTATTTGCCTTGTTTTCGCGAACAAGGCTTATTAGATACACCTATTGTACCGTTACTCTACGAATATTTCAAGTAGTAATTAC
TAGCATTGTCCGTTACTCTACGAATATTTCAAGTAGTAATTACTAGCATTGTCCGTTACTCTACGAATATTTCAAGTAGTAATT
ACTAGCATTGTCATATACATAATAAAACGGATATAAAAGGGCGTTTTCTATACCTAGAAGTCTTGTAAATGTACAGGGCGTTTA
GATATAGAGAACGCCCTTTTTGTGTTCCGTTCCAGTGGAAGCTACCACTTTAAAAAGATGGTCTAGTGTAGCCAATGCAGGAGA
GTACACTCGGATATCAGTTGTCGTTGCATTCAACTGTCTGACGTAAGCGAGGTAAAGGACACAAGCCTTGCATAAAACAAGCCT
ACGGGATGTAAATCCTAATAATGATGATAACCAAGACGTTAGCGGCAAAAAGTGTTGGGGGTTCAAAATAAGACATGATTGTGC
GACTGGAGTTAAACAGTTACTCGTAAGCGGCGATCATGACACTGATTCACGGCTATTCTTGTACAAGCTAGCTTTATTACAAGG
ATATGCGGGTTATATAGCGAATCACCCGAAAGGGAACGGTGTTGGGCGTGAGAAACGCACCGTACGGCGCAATACAATGCCAAT
AAGCTATATACGGACGGTATAGTAGTTTTGTAAGCTATAACCGTTTGTCGTCAATGCAACCAATCTCAATTCGAGACCTCGGCA
TCTAAGCCAGTACGAATGAGTGGGCGTTTTAACCTCGTAAATTTTCAACAGGGGTTACTATGCCCAAAACTACATTCAGATTTC
CTAACAAACTCGCCAGTATGAAAACCTTAAGACCTTAAAGTCAAGGGATTTGAAGGATTTTAACCTCGATTAGCAAAAAATGTA
GAGTACTGAAGCAACTACCATTAACTAAGATAGTGGGGGATTGAGGAAGAATCCAGAGCTGTTTAAATCAAGTGAAAGACAAGA
TGAAATTAAAAGAATAGTGAAAGATAGGGGAGTGGTTCTCTATGAGAAAGGAAATGGCTAGAGAACAAAGGCAGCGGTTTATTG
ATCTATTGTTAGACTTTATGGTAAAGAATCCTCATTTATTTGTTAATGGTACAGAGGATGAAAGTAATAATGTTGTTACAAAAT
GTAATAGTGATATTAAAGAGGTTGCGGAGTCATATTTAACTCTTTTATAGTGAGAGGGTTAAAACTAATTAATATGTATTAAGG
CCCAATGTTGGAATTATTGTATTTCACTAGGCAACCTACTTACTAAAAGTAAGATTATCCATTAGTGGATGTTATAATATTGGG
TTTTTTAACACAATAATCATCGCCTTTCGGTGTCGTTTGATAGAAAAGTAACCATTAGCGATGAAAAAGTCAATATAAAAAGCC
ATCCGTAAAAAACGGATGGCTTACCGTACATAGGATCGTTGGTAGGGCGGCGTATCCTACATCTCTGGTAACTTACCTAGCCAA
TCAAATGCTTGAGAACGGCGGTTAGATAAGCGCGTGGGGAACCTTTCCCACCTCAAAGATCCTATATCATTATTATGTTACTTT
CTACAGGTAGTATACCATGTTCTTATATTTTAGTAAACTCCCCGTTAGCTTAACAGGTCTTTGTAAGCAATTAAACGTCCACTA
TTCAATCGTCTTTGGATTTTCGCAGGACCGTTTTTTAGATCGAACATAGTTGATAAGAACAAATAACCGCTTGGGTCCAACTTT
ATAGCAATTAGTATATGGTCATTTAAAATCTTTACCAATTCAACGCTATTAGGTTCTTTAGGATTTTGCCCGACATAGTCGGGG
TGTTCAACGATATCTTTTATGTGCGATGAATATTTTTCATAAATACCAGGATGTTGTTTCTTTACGTGCTTTATAAATCCGGGA
AACATTTTTACATCGTTAGAAGTGCAAGTCAAGTTATATGTATCTATAATGATTTGTGGAAGTTTTGCCACAACAGTTGGTTTA
TTTACAATCTTTTTTTTATTAGCCGTCAAATTTCTCCCTCATCTCGTCTCTTTATATCTTTATTTTATCATAAAGGAGTATTTG
AACCGTCGCGCGGGACAGGTTTATGATAGGGATATTTTATTGAATAATTGATGGTATAAGGGACTTTCATGCTTGGAAAGTGGG
GATTATGAATTAGATGCTTGTCCACAATATGTTCCAATGTAATTAAAATTTATGTTCCCACCTTGACCAAACATCACGTCCATA
CTTAAATCGTCCCTCCTTTAATAGGTAAAATATTAATTTACCTTAATAAAAAAATAATGGATAATAGTATTCGTCTGAATTTAT
ATAATCAGGGGGAACTATTGATGCTGGGGATACTATTTACAGCGGCGCCATCTACTGATGTCGTAAAGGATTTGCAAGATAAAG
TTATATCATTGCAGGATCATGAGGTAGCGTTTTTGAACACCACGATATCTAATATGTTGATCCCCGAAGCAAACTTAAGAGTGT
GTTGATAGTGCAGTATCTTAAAATTTTGTGTATAATAGGAATTGAAGTTAAATTAGATGCTAAAAATTTGTAATTAAGAAGGAG
GGATTCGTCATGTTGGTATTCCAAATGCGTAATGTAGATAAAACATCTACTGTTTTGAAACAGACTAAAAACAGTGATTACGCA
GATAAATAAATACGTTAGATTAATTCCTACCAGTGACTAATCTTATGACTTTTTAAACAGATAACTAAAATTACAAACAAATCG
TTTAACTTCTGTATTTATTTACAGATGTAATCACTTCAGGAGTAATTACATGAACAAAAATATAAAATATTCTCAAAACTTTTT
AACGAGTGAAAAAGTACTCAACCAAATAATAAAACAATTGAATTTAAAAGAAACCGATACCGTTTACGAAATTGGAACAGGTAA
AGGGCATTTAACGACGAAACTGGCTAAAATAAGTAAACAGGTAACGTCTATTGAATTAGACAGTCATCTATTCAACTTATCGTC
AGAAAAATTAAAACTGAACATTCGTGTCACTTTAATTCACCAAGATATTCTACAGTTTCAATTCCCTAACAAACAGAGGTATAA
```

AATTGTTGGGAGTATTCCTTACCATTTAAGCACACAAATTATTAAAAAAGTGGTTTTTGAAAGCCATGCGTCTGACATCTATCT
GATTGTTGAAGAAGGATTCTACAAGCGTACCTTGGATATTCACCGAACACTAGGGTTGCTCTTGCACACTCAAGTCTCGATTCA
GCAATTGCTTAAGCTGCCAGCGGAATGCTTTCATCCTAAACCAAAAGTAAACAGTGTCTTAATAAAACTTACCCGCCATACCAC
AGATGTTCCAGATAAATATTGGAAGCTATATACGTACTTTGTTTCAAAATGGGTCAATCGAGAATATCGTCAACTGTTTACTAA
AAATCAGTTTCATCAAGCAATGAAACACGCCAAAGTAAACAATTTAAGTACCATTACTTATGAGCAAGTATTGTCTATTTTTAA
TAGTTATCTATTATTTAACGGGAGGAAATAATTCTATGAGTCGCTTTTTTAAATTTGGAAAGTTACACGTTACTAAAGGGAATG
GAGATAAATTATTAGATATACTACTGACAGCTTCCAAGAAGCTAAAGAGGTCCCTAGCGCCTACGGGGAATTTGGGGTACATTG
AAAAAGGAAGAGTATGAGTATTCAACATTTCCGTGTCGCCCTTATTCCCTTTTTTGCGGCATTTTGCCTTCCTGTTTTTGCTCA
CCCAGAAACGCTGGTGAAAGTAAAAGATGCTGAAGATCAGTTGGGTGCACGAGTGGGTTACATCGAACTGGATCTCAACAGCGG
TAAGATCCTTGAGAGTTTTCGCCCCGAAGAACGTTTTCCAATGATGAGCACTTTTAAAGTTCTGCTATGTGGCGCGGTATTATC
CCGTATTGACGCCGGGCAAGAGCAACTCGGTCGCCGCATACACTATTCTCAGAATGACTTGGTTGAGTACTCACCAGTCACAGA
AAAGCATCTTACGGATGGCATGACAGTAAGAGAATTATGCAGTGCTGCCATAACCATGAGTGATAACACTGCGGCCAACTTACT
TCTGACAACGATCGGAGGACCGAAGGAGCTAACCGCTTTTTTGCACAACATGGGGGATCATGTAACTCGCCTTGATCGTTGGGA
ACCGGAGCTGAATGAAGCCATACCAAACGACGAGCGTGACACCACGATGCCTGTAGCAATGGCAACAACGTTGCGCAAACTATT
AACTGGCGAACTACTTACTCTAGCTTCCCGGCAACAATTAATAGACTGGATGGAGGCGGATAAAGTTGCAGGACCACTTCTGCG
CTCGGCCCTTCCGGCTGGCTGGTTTATTGCTGATAAATCTGGAGCCGGTGAGCGTGGGTCTCGCGGTATCATTGCAGCACTGGG
GCCAGATGGTAAGCCCTCCCGTATCGTAGTTATCTACACGACGGGGAGTCAGGCAACTATGGATGAACGAAATAGACAGATCGC
TGAGATAGGTGCCTCACTGATTAAGCATTGGTAACTGTCAGACCAAGTTTACTCATATATACTTTAGATTGATTTAAAACTTCA
TTTTTAATTTAAAAGGATCTAGGTGAAGATCCTTTTTGATAATCTCATGACCAAAATCCCTTAACGTGAGTTTTCGTTCCACTG
AGCGTCAGACCCCGTAGAAAAGATCAAAGGATCTTC-3′

pXyl-Se-agrC

5′-
TTGAGATCCTTTTTTTCTGCGCGTAATCTGCTGCTTGCAAACAAAAAAACCACCGCTACCAGCGGTGGTTTGTTTGCCGGATCA
AGAGCTACCAACTCTTTTTCCGAAGGTAACTGGCTTCAGCAGAGCGCAGATACCAAATACTGTTCTTCTAGTGTAGCCGTAGTT
AGGCCACCACTTCAAGAACTCTGTAGCACCGCCTACATACCTCGCTCTGCTAATCCTGTTACCAGTGGCTGCTGCCAGTGGCGA
TAAGTCGTGTCTTACCGGGTTGGACTCAAGACGATAGTTACCGGATAAGGCGCAGCGGTCGGGCTGAACGGGGGGTTCGTGCAC
ACAGCCCAGCTTGGAGCGAACGACCTACACCGAACTGAGATACCTACAGCGTGAGCTATGAGAAAGCGCCACGCTTCCCGAAGG
GAGAAAGGCGGACAGGTATCCGGTAAGCGGCAGGGTCGGAACAGGAGAGCGCACGAGGGAGCTTCCAGGGGGAAACGCCTGGTA
TCTTTATAGTCCTGTCGGGTTTCGCCACCTCTGACTTGAGCGTCGATTTTTGTGATGCTCGTCAGGGGGGCGGAGCCTATGGAA
AAACGCCAGCAACGCGGCCTTTTTACGGTTCCTGGCCTTTTGCTGGCCTTTTGCTCACATGTTCTTTCCTGCGTTATCCCCTGA
TTCTGTGGATAACCGTATTACCGCCTTTGAGTGAGCTGATACCGCTCGCCGCAGCCGAACGACCGAGCGCAGCGAGTCAGTGAG
CGAGGAAGCGGAAGAGCGCCCAATACGCAAACCGCCTCTCCCCGCGCGTTGGCCGATTCATTAATGCAGCTGGCACGACAGGTT
TCCCGACTGGAAAGCGGGCAGTGAGCGCAACGCAATTAATGTGAGTTAGCTCACTCATTAGGCACCCCAGGCTTTACACTTTAT
GCTTCCGGCTCGTATGTTGTGTGGAATTGTGAGCGGATAACAATTTCACACAGGAAACAGCTATGACCATGATTACGCCAAGCT
TGCATGCCTGCATTATGATTCCTTATTATTTATTTCTACTTTTTGTACAAAGTAACCATTTTCGATGACAGTATCTAATAAAAC
ATTCTCGTTTGAGTCTGTCAGTTCTTTTAAAGTTGATAAACCTAAACCGCGATTATCACCTTTAGTAGAAAAACCTTGTTCAAA
CAACTCATGAATTTTAGGGATATCATTACTACATTTATTCATAACGATAAAAGTGACAGATTCCTCATTATCGATGAATGCGAT
ATTGATGAGTGGTTCCTCAAGATTTTCTGAAGCTTCAATAGCATTATCAACTATAATACCGATAATACGACTTAGCTCAACAGT
ATTCATATCGATACGATCAATTTCATCAGGAACCTCAATACTAATTGGAATACGTTTTTCTTGAGCTTGAATAATTTTAGTAGT
AATCAGCCCTTTAATTTCTCTCACTTTCAACTTTTCAATACCATTCATTTTAATAGAGCGAGTTTTTAATTTATCTTTCATTGG
AACGATATTTTCATTAAAATATTTACGTAATCCAGGCATATCATCTTCTCTAATGTAATCTGAAAGAGTGGTGAGGATATTCAC
ATAATCATGTCGGAACTTACGCATTTCATTGTTAATGCTTTCTATACGTAACGTGTACTCATAATATGCTTCGATTTCTTTTAC
ATTACGTTTATACCTCATTTCACGGAGTGTAAAATTGGACATCACTAATATCACTACACTTAAAAAAACCATAATACCCAACAA
TAAGATGGCATATAATTTAAGGGTATCATTTCCTCGCATATCAGTTTGTGACACCATATAAAGTAAAATAAATGATATGAAAAG
TACTATTGTTATTATCAATAAGTATCTTTTATTGAGTGACAAGTAGGATACTTTTAATTTATTGAACAATAGTTGAGTTAAATA
AGCAATAATTAGAGTTATGATTACAAAAGAGGTAAAATGTATTAACTGTAAAGCAAATTTAAACGGAATATAATCTTTTATAGT
CAAATGTATGTATACAGTTATGAAATTAGTTATATATAAGATCATAGTGGTGAATAATACAACTAATATTGAATAAAGCTTTAT
TTTTGTATAAAAGAAAATGGTGATTATTATAACCAAAACTATTAATGCTTTACTTTGCCAAAAGTAATACATTATTGCAGAAGG
GATTACAATCGTAAAAACGATTATGTAATCCCTAAAATTAAATTTCATATTAATGATAACTTTAGTAACCCAAATCATTAAAAA
GATTTGTAGGCCTGCAAACGGAAATAGATTAATATCATCCATAGTAGTTCCTCCTTATCTACAGATGCATTTTATTTCATATAG
TAAGTACATCACCTATTAGTTTGTTGTTTAAACAAACTAACTTATTTTCATCTTATATAACCTCGTCAGTATTTTCAATATTTT
TTTTAGTTTTTTATGAACACATTAGATATAATAAAGGGAAGATTCGCTATGTACTATGTTGATACTTAATTTAAAGATTAAACA
AATGGAGTGGATGAAGTGGATATCGCTGATCAAACCTTTGTCAAAAAAGTAAATCAAAAGTTATTATTAAAAGAAATCCTTAAA
AATTCACCTATTTCAAGAGCAAAATTATCTGAAATGACTGGATTAAATAAATCAACTGTCTCATCACAGGTAAACACGTTAATG
AAAGAAAGTATGGTATTTGAAATAGGTCAAGGACAATCAAGTGGCGGAAGAAGACCTGTCATGCTTGTTTTTAATAAAAAGGCA
GGATACTCCGTTGGAATAGATGTTGGTGTGGATTATATTAATGGCATTTTAACAGACCTTGAAGGAACAATCGTTCTTGATCAA
TACCGCCATTTGGAATCCAATTCTCCAGAAATAACGAAAGACATTTTGATTGATATGATTCATCACTTTATTACGCAAATGCCC
CAATCTCCGTACGGGCTTATTGGTATAGGTATTTGCGTGCCTGGACTCATTGATAAAGATCAAAAAATTGTTTTCACTCCGAAC
TCCAACTGGAGAGATATTGACTTAAAATCTTCGATACAAGAGAAGTACAATGTGCCTGTTTTTATTGAAAATGAGGCAAATGCT
GGCGCATATGGAGAAAAAGTATTTGGAGCTGCAAAAAATCACGATAACATTATTTACGTAAGTATCAGCACAGGAATAGGGATC
GGTGTTATTATCAACAATCATTTATATAGAGGAGTAAGCGGCTTCTCTGGAGAAATGGGACATATGACAATAGACTTTAATGGT

```
CCTAAATGCAGTTGCGGAAACCGAGGATGCTGGGAATTGTATGCTTCAGAGAAGGCTTTATTAAAATCTCTTCAGACCAAAGAG
AAAAAACTGTCCTATCAAGATATCATAAACCTCGCCCATCTGAATGATATCGGAACCTTAAATGCATTACAAAATTTTGGATTC
TATTTAGGAATAGGCCTTACCAATATTCTAAATACTTTCAACCCACAAGCCGTAATTTTAAGAAATAGCATAATTGAATCGCAT
CCTATGGTTTTAAATTCAATGAGAAGTGAAGTATCATCAAGGGTTTATTCCCAATTAGGCAATAGCTATGAATTATTGCCATCT
TCCTTAGGACAGAATGCACCGGCATTAGGAATGTCCTCCATTGTGATTGATCATTTTCTGGACATGATTACAATGTAATTTTTT
ATGGAATGGACAGCTCATCTTTAAAGATGAGTTTTTTTATTCTAGGAGTATTTCTGAAGCAATAGTGACATGGCACCTTCTCAT
ATGAAAAAGGAGTTCTAAAATAGAAATCTCCTTTTTCATGTGCAAATTATTTTTCTTTATAACGAAAATATCTAAAGTCGGCCA
ATTCACTGGCCGTCGTTTTACCGAAATACGGGCAGACATGGCCTGCCCGGTTAATGCCTGGCTCCAAGTGTCATTATACAATTT
TGCGCAACATTTTTTAGAAAGCATGCCTAACTGTTAAAAAAATATACCTAAGTGTTTTAATTAAGTACTATTAGATATTTTACC
ATATTTAGTTTTACAGTTGAGTACTAAATATTGCTATTTACGAAATTTTAATCTTTAAATGGAAAAATCATGTTTTAATAGACT
CAGATCACAGAGATGTGGTCAGTCAGTCTTAATTAACCAAGGAGGTGAAATGTACAATGAAAGGAGAAGAACTTTTCACTGGAG
TTGTCCCAATTCTTGTTGAATTAGATGGTGATGTTAATGGGCACAAATTTTCTGTCAGTGGAGAGGGTGAAGGTGATGCAACAT
ACGGAAAACTTACCCTTAAATTTATTTGCACTACTGGAAAACTACCTGTTCCATGGCCAACACTTGTCACTACTTTCGCGTATG
GTCTTCAATGCTTTGCGAGATACCCAGATCATATGAAACAGCATGACTTTTTCAAGAGTGCCATGCCCGAAGGTTATGTACAGG
AAAGAACTATATTTTTCAAAGATGACGGGAACTACAAGACACGTGCTGAAGTCAAGTTTGAAGGTGATACCCTTGTTAATAGAA
TCGAGTTAAAAGGTATTGATTTTAAAGAAGATGGAAACATTCTTGGACACAAATTGGAATACAACTATAACTCACACAATGTAT
ACATCATGGCAGACAAACAAAAGAATGGAATCAAAGTTAACTTCAAAATTAGACACAACATTGAAGATGGAAGCGTTCAACTAG
CAGACCATTATCAACAAAATACTCCAATTGGCGATGGCCCTGTCCTTTTACCAGACAACCATTACCTGTCCACACAATCTAAGC
TTTCGAAAGATCCCAACGAAAAGAGAGACCACATGGTCCTTCTTGAGTTTGTAACAGCTGCTGGGATTACACATGGCATGGATG
AACTATACAAATAACCTAGGCTGCTGCCACCGCTGAGCAATAACTAGCATAACCCCTTGGGGCCTCTAAACGGGTCTTGAGGGG
TTTTTTGAACGTCGTGACTGGGAAAACCCTGGCGTTACCCAACTTAATCGCCTTGCAGCACATCCCCCTTTCGCCAGCTGGCGT
AATAGCGAAGAGGCCCGCACCGATCGCCCTTCCCAACAGTTGCGCAGCCTGAATGGCGAATGGCGCCTGATGCGGTATTTTCTC
CTTACGCATCTGTGCGGTATTTCACACCGCATATGGTGCACTCTCAGTACAATCTGCTCTGATGCCGCATAGTTAAGCCAGCCC
CGACACCCGCCAACACCCGCTGACGCGCCCTGACGGGCTTGTCTGTTCCCGGCATCCGCTTACAGACAAGCTGTGACCGTCTCC
GGGAGCTGCATGTGTCAGAGGTTTTCACCGTCATCACCGAAACGCGCGAGACGAAAGGGCCTCGTGATACGCCTATTTTTATAG
GTTAATGTCATGATAATAATGGTTTCTTAGACGTCAGGTGGCACTTTTCGGGGAAATGTGCGCGGAACCCCTATTTGTTTATTT
TTCTAAATACATTCAAATATGTATCCGCTCATGAGACAATAACCCTGATAAATGCTTCAATAATCCATCCTCCAAAGTTGGAGA
GTGAGTTTTATGTCGCAAATATTAATGTTTCTGGTGAACCTTATCAAATTTTCGTTGATTTAATAGAAACATAGCGGTAAAATT
AGCAGTAACTTAATAGAACGGAAATGAAAAAAGCCACTCTCATATGCTATTGGCTACCAACCTTTAGCGAGAATGACTTAATCC
TGTACAGCCATACAGGACTTCGACTTATAAGAGGCGCCAACTTCAAATAAGTTATTTGCCTTGTTTTCGCGAACAAGGCTTATT
AGATACACCTATTGTACCGTTACTCTACGAATATTTCAAGTAGTAATTACTAGCATTGTCCGTTACTCTACGAATATTTCAAGT
AGTAATTACTAGCATTGTCCGTTACTCTACGAATATTTCAAGTAGTAATTACTAGCATTGTCATATACATAATAAAACGGATAT
AAAAGGGCGTTTTCTATACCTAGAAGTCTTGTAAATGTACAGGGCGTTTAGATATAGAGAACGCCCTTTTTGTGTTCCGTTCCA
GTGGAAGCTACCACTTTAAAAAGATGGTCTAGTGTAGCCAATGCAGGAGAGTACACTCGGATATCAGTTGTCGTTGCATTCAAC
TGTCTGACGTAAGCGAGGTAAAGGACACAAGCCTTGCATAAAACAAGCCTACGGGATGTAAATCCTAATAATGATGATAACCAA
GACGTTAGCGGCAAAAAGTGTTGGGGGTTCAAAATAAGACATGATTGTGCGACTGGAGTTAAACAGTTACTCGTAAGCGGCGAT
CATGACACTGATTCACGGCTATTCTTGTACAAGCTAGCTTTATTACAAGGATATGCGGGTTATATAGCGAATCACCCGAAAGGG
AACGGTGTTGGGCGTGAGAAACGCACCGTACGGCGCAATACAATGCCAATAAGCTATATACGGACGGTATAGTAGTTTTGTAAG
CTATAACCGTTTGTCGTCAATGCAACCAATCTCAATTCGAGACCTCGGCATCTAAGCCAGTACGAATGAGTGGGCGTTTTAACC
TCGTAAATTTTCAACAGGGGTTACTATGCCCAAAACTACATTCAGATTTCCTAACAAACTCGCCAGTATGAAAACCTTAAGACC
TTAAAGTCAAGGGATTTGAAGGATTTTAACCTCGATTAGCAAAAAATGTAGAGTACTGAAGCAACTACCATTAACTAAGATAGT
GGGGGATTGAGGAAGAATCCAGAGCTGTTTAAATCAAGTGAAAGACAAGATGAAATTAAAAGAATAGTGAAAGATAGGGGAGTG
GTTCTCTATGAGAAAGGAAATGGCTAGAGAACAAAGGCAGCGGTTTATTGATCTATTGTTAGACTTTATGGTAAAGAATCCTCA
TTTATTTGTTAATGGTACAGAGGATGAAAGTAATAATGTTGTTACAAAATGTAATAGTGATATTAAAGAGGTTGCGGAGTCATA
TTTAACTCTTTTATAGTGAGAGGGTTAAAACTAATTAATATGTATTAAGGCCCAATGTTGGAATTATTGTATTTCACTAGGCAA
CCTACTTACTAAAAGTAAGATTATCCATTAGTGGATGTTATAATATTGGGTTTTTTAACACAATAATCATCGCCTTTCGGTGTC
GTTTGATAGAAAAGTAACCATTAGCGATGAAAAAGTCAATATAAAAAGCCATCCGTAAAAAACGGATGGCTTACCGTACATAGG
ATCGTTGGTAGGGCGGCGTATCCTACATCTCTGGTAACTTACCTAGCCAATCAAATGCTTGAGAACGGCGGTTAGATAAGCGCG
TGGGGAACCTTTCCCACCTCAAAGATCCTATATCATTATTATGTTACTTTCTACAGGTAGTATACCATGTTCTTATATTTTAGT
AAACTCCCCGTTAGCTTAACAGGTCTTTGTAAGCAATTAAACGTCCACTATTCAATCGTCTTTGGATTTTCGCAGGACCGTTTT
TTAGATCGAACATAGTTGATAAGAACAAATAACCGCTTGGGTCCAACTTTATAGCAATTAGTATATGGTCATTTAAAATCTTTA
CCAATTCAACGCTATTAGGTTCTTTAGGATTTTGCCCGACATAGTCGGGGTGTTCAACGATATCTTTTATGTGCGATGAATATT
TTTCATAAATACCAGGATGTTGTTTCTTTACGTGCTTTATAAATCCGGGAAACATTTTTACATCGTTAGAAGTGCAAGTCAAGT
TATATGTATCTATAATGATTTGTGGAAGTTTTGCCACAACAGTTGGTTTATTTACAATCTTTTTTTTTATTAGCCGTCAAATTTC
TCCCTCATCTCGTCTCTTTATATCTTTATTTTATCATAAAGGAGTATTTGAACCGTCGCGCGGGACAGGTTTATGATAGGGATA
TTTTATTGAATAATTGATGGTATAAGGGACTTTCATGCTTGGAAAGTGGGGATTATGAATTAGATGCTTGTCCACAATATGTTC
CAATGTAATTAAAATTTGTGTTCCCACCTTGACCAAACATCACGTCCATACTTAAATCGTCCCTCCTTTAATAGGTAAAATATT
AATTTACCTTAATAAAAAAATAATGGATAATAGTATTCGTCTGAATTTATATAATCAGGGGGAACTATTGATGCTGGGGATACT
ATTTACAGCGGCGCCATCTACTGATGTCGTAAAGGATTTGCAAGATAAAGTTATATCATTGCAGGATCATGAGGTAGCGTTTTT
GAACACCACGATATCTAATATGTTGATCCCCGAAGCAAACTTAAGAGTGTGTTGATAGTGCAGTATCTTAAAATTTTGTGTATA
ATAGGAATTGAAGTTAAATTAGATGCTAAAAATTTGTAATTAAGAAGGAGGGATTCGTCATGTTGGTATTCCAAATGCGTAATG
TAGATAAAACATCTACTGTTTTGAAACAGACTAAAAACAGTGATTACGCAGATAAATAAATACGTTAGATTAATTCCTACCAGT
GACTAATCTTATGACTTTTTAAACAGATAACTAAAATTACAAACAAATCGTTTAACTTCTGTATTTATTTACAGATGTAATCAC
```

```
TTCAGGAGTAATTACATGAACAAAAATATAAAATATTCTCAAAACTTTTTAACGAGTGAAAAAGTACTCAACCAAATAATAAAA
CAATTGAATTTAAAAGAAACCGATACCGTTTACGAAATTGGAACAGGTAAAGGGCATTTAACGACGAAACTGGCTAAAATAAGT
AAACAGGTAACGTCTATTGAATTAGACAGTCATCTATTCAACTTATCGTCAGAAAAATTAAAACTGAACATTCGTGTCACTTTA
ATTCACCAAGATATTCTACAGTTTCAATTCCCTAACAAACAGAGGTATAAAATTGTTGGGAGTATTCCTTACCATTTAAGCACA
CAAATTATTAAAAAAGTGGTTTTTGAAAGCCATGCGTCTGACATCTATCTGATTGTTGAAGAAGGATTCTACAAGCGTACCTTG
GATATTCACCGAACACTAGGGTTGCTCTTGCACACTCAAGTCTCGATTCAGCAATTGCTTAAGCTGCCAGCGGAATGCTTTCAT
CCTAAACCAAAAGTAAACAGTGTCTTAATAAAACTTACCCGCCATACCACAGATGTTCCAGATAAATATTGGAAGCTATATACG
TACTTTGTTTCAAAATGGGTCAATCGAGAATATCGTCAACTGTTTACTAAAAATCAGTTTCATCAAGCAATGAAACACGCCAAA
GTAAACAATTTAAGTACCATTACTTATGAGCAAGTATTGTCTATTTTTAATAGTTATCTATTATTTAACGGGAGGAAATAATTC
TATGAGTCGCTTTTTTAAATTTGGAAAGTTACACGTTACTAAAGGGAATGGAGATAAATTATTAGATATACTACTGACAGCTTC
CAAGAAGCTAAAGAGGTCCCTAGCGCCTACGGGGAATTTGGGGTACATTGAAAAAGGAAGAGTATGAGTATTCAACATTTCCGT
GTCGCCCTTATTCCCTTTTTTGCGGCATTTTGCCTTCCTGTTTTTGCTCACCCAGAAACGCTGGTGAAAGTAAAAGATGCTGAA
GATCAGTTGGGTGCACGAGTGGGTTACATCGAACTGGATCTCAACAGCGGTAAGATCCTTGAGAGTTTTCGCCCCGAAGAACGT
TTTCCAATGATGAGCACTTTTAAAGTTCTGCTATGTGGCGCGGTATTATCCCGTATTGACGCCGGGCAAGAGCAACTCGGTCGC
CGCATACACTATTCTCAGAATGACTTGGTTGAGTACTCACCAGTCACAGAAAAGCATCTTACGGATGGCATGACAGTAAGAGAA
TTATGCAGTGCTGCCATAACCATGAGTGATAACACTGCGGCCAACTTACTTCTGACAACGATCGGAGGACCGAAGGAGCTAACC
GCTTTTTTGCACAACATGGGGGATCATGTAACTCGCCTTGATCGTTGGGAACCGGAGCTGAATGAAGCCATACCAAACGACGAG
CGTGACACCACGATGCCTGTAGCAATGGCAACAACGTTGCGCAAACTATTAACTGGCGAACTACTTACTCTAGCTTCCCGGCAA
CAATTAATAGACTGGATGGAGGCGGATAAAGTTGCAGGACCACTTCTGCGCTCGGCCCTTCCGGCTGGCTGGTTTATTGCTGAT
AAATCTGGAGCCGGTGAGCGTGGGTCTCGCGGTATCATTGCAGCACTGGGGCCAGATGGTAAGCCCTCCCGTATCGTAGTTATC
TACACGACGGGGAGTCAGGCAACTATGGATGAACGAAATAGACAGATCGCTGAGATAGGTGCCTCACTGATTAAGCATTGGTAA
CTGTCAGACCAAGTTTACTCATATATACTTTAGATTGATTTAAAACTTCATTTTTAATTTAAAAGGATCTAGGTGAAGATCCTT
TTTGATAATCTCATGACCAAAATCCCTTAACGTGAGTTTTCGTTCCACTGAGCGTCAGACCCCGTAGAAAAGATCAAAGGATCT
TC-3'
```

pXyl-Se-agrA

```
5'-
TTGAGATCCTTTTTTTCTGCGCGTAATCTGCTGCTTGCAAACAAAAAAACCACCGCTACCAGCGGTGGTTTGTTTGCCGGATCA
AGAGCTACCAACTCTTTTTCCGAAGGTAACTGGCTTCAGCAGAGCGCAGATACCAAATACTGTTCTTCTAGTGTAGCCGTAGTT
AGGCCACCACTTCAAGAACTCTGTAGCACCGCCTACATACCTCGCTCTGCTAATCCTGTTACCAGTGGCTGCTGCCAGTGGCGA
TAAGTCGTGTCTTACCGGGTTGGACTCAAGACGATAGTTACCGGATAAGGCGCAGCGGTCGGGCTGAACGGGGGGTTCGTGCAC
ACAGCCCAGCTTGGAGCGAACGACCTACACCGAACTGAGATACCTACAGCGTGAGCTATGAGAAAGCGCCACGCTTCCCGAAGG
GAGAAAGGCGGACAGGTATCCGGTAAGCGGCAGGGTCGGAACAGGAGAGCGCACGAGGGAGCTTCCAGGGGGAAACGCCTGGTA
TCTTTATAGTCCTGTCGGGTTTCGCCACCTCTGACTTGAGCGTCGATTTTTGTGATGCTCGTCAGGGGGGCGGAGCCTATGGAA
AAACGCCAGCAACGCGGCCTTTTTACGGTTCCTGGCCTTTTGCTGGCCTTTTGCTCACATGTTCTTTCCTGCGTTATCCCCTGA
TTCTGTGGATAACCGTATTACCGCCTTTGAGTGAGCTGATACCGCTCGCCGCAGCCGAACGACCGAGCGCAGCGAGTCAGTGAG
CGAGGAAGCGGAAGAGCGCCCAATACGCAAACCGCCTCTCCCCGCGCGTTGGCCGATTCATTAATGCAGCTGGCACGACAGGTT
TCCCGACTGGAAAGCGGGCAGTGAGCGCAACGCAATTAATGTGAGTTAGCTCACTCATTAGGCACCCCAGGCTTTACACTTTAT
GCTTCCGGCTCGTATGTTGTGTGGAATTGTGAGCGGATAACAATTTCACACAGGAAACAGCTATGACCATGATTACGCCAAGCT
TGCATGCCTGCATTATATTTTTTTAACATTACGTACTGAAGCGAAACAATTTTCGCCATTCTTAAAGTAAACAATACGTTCTTT
TGAGTCAATAGATTCAATATTATGCCTGTTTATTACAAAACTGTTATGACATCTAAAGAAACGTTCATCAAGCTGTGCTAATTC
CTTTAAATTTCCATAAAATTCAATTTGTCGATTATCAAGATGTGCAATGAGTCTATGAGATTTCGTAGATGATTCAAAAAACAT
AATATCATCATATTGAACGTATACTGAATTACTTCCCCGCTTTAACTCAATCGTATCTACATTACTTTCTTTTGATAATAATTT
GAGTCGTGTATGTGCTGTTTCAAGACAATCTATGATTCTCATTTTTAATTCAGATGGATCATCTTTAAAAATAAAATCCATAGC
AGCCACTTTATAAACAAACGTCAAATAAGTCAGCTCACTGTGACTGGTTACAAATATAATATTACCAACAGGATCATGTTTACG
AATTTCACTGGCTAATTTAATACCGTTCATATCAGCTTCTAATTGAATATCAAGGAAGTAACAACCAATGTCATTAAGTTCTTT
TGATTGCTCTAAGACCTCATAAGGATCATTTGTTGCTAAAGCTAACTCCATTGGCTTTTCTTCAATCATTATGTAGTTTTTAAT
GATTGATACCATATGTTCTCTTTGTCTTTGGTCATCTTCACAAACAAAAATTTTCATAGTAGTTCCTCCTTATCTACAGATGCA
TTTTATTTCATATAGTAAGTACATCACCTATTAGTTTGTTGTTTAAACAAACTAACTTATTTTCATCTTATATAACCTCGTCAG
TATTTTCAATATTTTTTTTAGTTTTTTATGAACACATTAGATATAATAAAGGGAAGATTCGCTATGTACTATGTTGATACTTAA
TTTAAAGATTAAACAAATGGAGTGGATGAAGTGGATATCGCTGATCAAACCTTTGTCAAAAAAGTAAATCAAAAGTTATTATTA
AAAGAAATCCTTAAAAATTCACCTATTTCAAGAGCAAAATTATCTGAAATGACTGGATTAAATAAATCAACTGTCTCATCACAG
GTAAACACGTTAATGAAAGAAAGTATGGTATTTGAAATAGGTCAAGGACAATCAAGTGGCGGAAGAAGACCTGTCATGCTTGTT
TTTAATAAAAAGGCAGGATACTCCGTTGGAATAGATGTTGGTGTGGATTATATTAATGGCATTTTAACAGACCTTGAAGGAACA
ATCGTTCTTGATCAATACCGCCATTTGGAATCCAATTCTCCAGAAATAACGAAAGACATTTTGATTGATATGATTCATCACTTT
ATTACGCAAATGCCCCAATCTCCGTACGGGCTTATTGGTATAGGTATTTGCGTGCCTGGACTCATTGATAAAGATCAAAAAATT
GTTTTCACTCCGAACTCCAACTGGAGAGATATTGACTTAAAATCTTCGATACAAGAGAAGTACAATGTGCCTGTTTTTATTGAA
AATGAGGCAAATGCTGGCGCATATGGAGAAAAAGTATTTGGAGCTGCAAAAAATCACGATAACATTATTTACGTAAGTATCAGC
ACAGGAATAGGGATCGGTGTTATTATCAACAATCATTTATATAGAGGAGTAAGCGGCTTCTCTGGAGAAATGGGACATATGACA
ATAGACTTTAATGGTCCTAAATGCAGTTGCGGAAACCGAGGATGCTGGGAATTGTATGCTTCAGAGAAGGCTTTATTAAAATCT
CTTCAGACCAAAGAGAAAAAACTGTCCTATCAAGATATCATAAACCTCGCCCATCTGAATGATATCGGAACCTTAAATGCATTA
CAAAATTTTGGATTCTATTTAGGAATAGGCCTTACCAATATTCTAAATACTTTCAACCCACAAGCCGTAATTTTAAGAAATAGC
```

```
ATAATTGAATCGCATCCTATGGTTTTAAATTCAATGAGAAGTGAAGTATCATCAAGGGTTTATTCCCAATTAGGCAATAGCTAT
GAATTATTGCCATCTTCCTTAGGACAGAATGCACCGGCATTAGGAATGTCCTCCATTGTGATTGATCATTTTCTGGACATGATT
ACAATGTAATTTTTTATGGAATGGACAGCTCATCTTTAAAGATGAGTTTTTTTATTCTAGGAGTATTTCTGAAGCAATAGTGAC
ATGGCACCTTCTCATATGAAAAAGGAGTTCTAAAATAGAAATCTCCTTTTTCATGTGCAAATTATTTTTCTTTATAACGAAAAT
ATCTAAAGTCGGCCAATTCACTGGCCGTCGTTTTACCGAAATACGGGCAGACATGGCCTGCCCGGTTAATGCCTGGCTCCAAGT
GTCATTATACAATTTTGCGCAACATTTTTTAGAAAGCATGCCTAACTGTTAAAAAAATATACCTAAGTGTTTTAATTAAGTACT
ATTAGATATTTTACCATATTTAGTTTTACAGTTGAGTACTAAATATTGCTATTTACGAAATTTTAATCTTTAAATGGAAAAATC
ATGTTTTAATAGACTCAGATCACAGAGATGTGGTCAGTCAGTCTTAATTAACCAAGGAGGTGAAATGTACAATGAAAGGAGAAG
AACTTTTCACTGGAGTTGTCCCAATTCTTGTTGAATTAGATGGTGATGTTAATGGGCACAAATTTTCTGTCAGTGGAGAGGGTG
AAGGTGATGCAACATACGGAAAACTTACCCTTAAATTTATTTGCACTACTGGAAAACTACCTGTTCCATGGCCAACACTTGTCA
CTACTTTCGCGTATGGTCTTCAATGCTTTGCGAGATACCCAGATCATATGAAACAGCATGACTTTTTCAAGAGTGCCATGCCCG
AAGGTTATGTACAGGAAAGAACTATATTTTTCAAAGATGACGGGAACTACAAGACACGTGCTGAAGTCAAGTTTGAAGGTGATA
CCCTTGTTAATAGAATCGAGTTAAAAGGTATTGATTTTAAAGAAGATGGAAACATTCTTGGACACAAATTGGAATACAACTATA
ACTCACACAATGTATACATCATGGCAGACAAACAAAAGAATGGAATCAAAGTTAACTTCAAAATTAGACACAACATTGAAGATG
GAAGCGTTCAACTAGCAGACCATTATCAACAAAATACTCCAATTGGCGATGGCCCTGTCCTTTTACCAGACAACCATTACCTGT
CCACACAATCTAAGCTTTCGAAAGATCCCAACGAAAAGAGAGACCACATGGTCCTTCTTGAGTTTGTAACAGCTGCTGGGATTA
CACATGGCATGGATGAACTATACAAATAACCTAGGCTGCTGCCACCGCTGAGCAATAACTAGCATAACCCCTTGGGGCCTCTAA
ACGGGTCTTGAGGGGTTTTTTGAACGTCGTGACTGGGAAAACCCTGGCGTTACCCAACTTAATCGCCTTGCAGCACATCCCCCT
TTCGCCAGCTGGCGTAATAGCGAAGAGGCCCGCACCGATCGCCCTTCCCAACAGTTGCGCAGCCTGAATGGCGAATGGCGCCTG
ATGCGGTATTTTCTCCTTACGCATCTGTGCGGTATTTCACACCGCATATGGTGCACTCTCAGTACAATCTGCTCTGATGCCGCA
TAGTTAAGCCAGCCCCGACACCCGCCAACACCCGCTGACGCGCCCTGACGGGCTTGTCTGTTCCCGGCATCCGCTTACAGACAA
GCTGTGACCGTCTCCGGGAGCTGCATGTGTCAGAGGTTTTCACCGTCATCACCGAAACGCGCGAGACGAAAGGGCCTCGTGATA
CGCCTATTTTTATAGGTTAATGTCATGATAATAATGGTTTCTTAGACGTCAGGTGGCACTTTTCGGGGAAATGTGCGCGGAACC
CCTATTTGTTTATTTTTCTAAATACATTCAAATATGTATCCGCTCATGAGACAATAACCCTGATAAATGCTTCAATAATCCATC
CTCCAAAGTTGGAGAGTGAGTTTTATGTCGCAAATATTAATGTTTCTGGTGAACCTTATCAAATTTTCGTTGATTTAATAGAAA
CATAGCGGTAAAATTAGCAGTAACTTAATAGAACGGAAATGAAAAAAGCCACTCTCATATGCTATTGGCTACCAACCTTTAGCG
AGAATGACTTAATCCTGTACAGCCATACAGGACTTCGACTTATAAGAGGCGCCAACTTCAAATAAGTTATTTGCCTTGTTTTCG
CGAACAAGGCTTATTAGATACACCTATTGTACCGTTACTCTACGAATATTTCAAGTAGTAATTACTAGCATTGTCCGTTACTCT
ACGAATATTTCAAGTAGTAATTACTAGCATTGTCCGTTACTCTACGAATATTTCAAGTAGTAATTACTAGCATTGTCATATACA
TAATAAAACGGATATAAAAGGGCGTTTTCTATACCTAGAAGTCTTGTAAATGTACAGGGCGTTTAGATATAGAGAACGCCCTTT
TTGTGTTCCGTTCCAGTGGAAGCTACCACTTTAAAAAGATGGTCTAGTGTAGCCAATGCAGGAGAGTACACTCGGATATCAGTT
GTCGTTGCATTCAACTGTCTGACGTAAGCGAGGTAAAGGACACAAGCCTTGCATAAAACAAGCCTACGGGATGTAAATCCTAAT
AATGATGATAACCAAGACGTTAGCGGCAAAAAGTGTTGGGGGTTCAAAATAAGACATGATTGTGCGACTGGAGTTAAACAGTTA
CTCGTAAGCGGCGATCATGACACTGATTCACGGCTATTCTTGTACAAGCTAGCTTTATTACAAGGATATGCGGGTTATATAGCG
AATCACCCGAAAGGGAACGGTGTTGGGCGTGAGAAACGCACCGTACGGCGCAATACAATGCCAATAAGCTATATACGGACGGTA
TAGTAGTTTTGTAAGCTATAACCGTTTGTCGTCAATGCAACCAATCTCAATTCGAGACCTCGGCATCTAAGCCAGTACGAATGA
GTGGGCGTTTTAACCTCGTAAATTTTCAACAGGGGTTACTATGCCCAAAACTACATTCAGATTTCCTAACAAACTCGCCAGTAT
GAAAACCTTAAGACCTTAAAGTCAAGGGATTTGAAGGATTTTAACCTCGATTAGCAAAAAATGTAGAGTACTGAAGCAACTACC
ATTAACTAAGATAGTGGGGGATTGAGGAAGAATCCAGAGCTGTTTAAATCAAGTGAAAGACAAGATGAAATTAAAAGAATAGTG
AAAGATAGGGGAGTGGTTCTCTATGAGAAAGGAAATGGCTAGAGAACAAAGGCAGCGGTTTATTGATCTATTGTTAGACTTTAT
GGTAAAGAATCCTCATTTATTTGTTAATGGTACAGAGGATGAAAGTAATAATGTTGTTACAAAATGTAATAGTGATATTAAAGA
GGTTGCGGAGTCATATTTAACTCTTTTATAGTGAGAGGGTTAAAACTAATTAATATGTATTAAGGCCCAATGTTGGAATTATTG
TATTTCACTAGGCAACCTACTTACTAAAAGTAAGATTATCCATTAGTGGATGTTATAATATTGGGTTTTTTAACACAATAATCA
TCGCCTTTCGGTGTCGTTTGATAGAAAAGTAACCATTAGCGATGAAAAAGTCAATATAAAAAGCCATCCGTAAAAAACGGATGG
CTTACCGTACATAGGATCGTTGGTAGGGCGGCGTATCCTACATCTCTGGTAACTTACCTAGCCAATCAAATGCTTGAGAACGGC
GGTTAGATAAGCGCGTGGGGAACCTTTCCCACCTCAAAGATCCTATATCATTATTATGTTACTTTCTACAGGTAGTATACCATG
TTCTTATATTTTAGTAAACTCCCCGTTAGCTTAACAGGTCTTTGTAAGCAATTAAACGTCCACTATTCAATCGTCTTTGGATTT
TCGCAGGACCGTTTTTTAGATCGAACATAGTTGATAAGAACAAATAACCGCTTGGGTCCAACTTTATAGCAATTAGTATATGGT
CATTTAAAATCTTTACCAATTCAACGCTATTAGGTTCTTTAGGATTTTGCCCGACATAGTCGGGGTGTTCAACGATATCTTTTA
TGTGCGATGAATATTTTTCATAAATACCAGGATGTTGTTTCTTTACGTGCTTTATAAATCCGGGAAACATTTTTACATCGTTAG
AAGTGCAAGTCAAGTTATATGTATCTATAATGATTTGTGGAAGTTTTGCCACAACAGTTGGTTTATTTACAATCTTTTTTTTAT
TAGCCGTCAAATTTCTCCCTCATCTCGTCTCTTTATATCTTTATTTTATCATAAAGGAGTATTTGAACCGTCGCGCGGGACAGG
TTTATGATAGGGATATTTTATTGAATAATTGATGGTATAAGGGACTTTCATGCTTGGAAAGTGGGGATTATGAATTAGATGCTT
GTCCACAATATGTTCCAATGTAATTAAAATTTATGTTCCCACCTTGACCAAACATCACGTCCATACTTAAATCGTCCCTCCTTT
AATAGGTAAAATATTAATTTACCTTAATAAAAAAATAATGGATAATAGTATTCGTCTGAATTTATATAATCAGGGGGAACTATT
GATGCTGGGGATACTATTTACAGCGGCGCCATCTACTGATGTCGTAAAGGATTTGCAAGATAAAGTTATATCATTGCAGGATCA
TGAGGTAGCGTTTTTGAACACCACGATATCTAATATGTTGATCCCCGAAGCAAACTTAAGAGTGTGTTGATAGTGCAGTATCTT
AAAATTTTGTGTATAATAGGAATTGAAGTTAAATTAGATGCTAAAAATTTGTAATTAAGAAGGAGGGATTCGTCATGTTGGTAT
TCCAAATGCGTAATGTAGATAAAACATCTACTGTTTTGAAACAGACTAAAAACAGTGATTACGCAGATAAATAAATACGTTAGA
TTAATTCCTACCAGTGACTAATCTTATGACTTTTTAAACAGATAACTAAAATTACAAACAAATCGTTTAACTTCTGTATTTATT
TACAGATGTAATCACTTCAGGAGTAATTACATGAACAAAAATATAAAATATTCTCAAAACTTTTTAACGAGTGAAAAAGTACTC
AACCAAATAATAAAACAATTGAATTTAAAAGAAACCGATACCGTTTACGAAATTGGAACAGGTAAAGGGCATTTAACGACGAAA
CTGGCTAAAATAAGTAAACAGGTAACGTCTATTGAATTAGACAGTCATCTATTCAACTTATCGTCAGAAAAATTAAAACTGAAC
```

```
ATTCGTGTCACTTTAATTCACCAAGATATTCTACAGTTTCAATTCCCTAACAAACAGAGGTATAAAATTGTTGGGAGTATTCCT
TACCATTTAAGCACACAAATTATTAAAAAAGTGGTTTTTGAAAGCCATGCGTCTGACATCTATCTGATTGTTGAAGAAGGATTC
TACAAGCGTACCTTGGATATTCACCGAACACTAGGGTTGCTCTTGCACACTCAAGTCTCGATTCAGCAATTGCTTAAGCTGCCA
GCGGAATGCTTTCATCCTAAACCAAAAGTAAACAGTGTCTTAATAAAACTTACCCGCCATACCACAGATGTTCCAGATAAATAT
TGGAAGCTATATACGTACTTTGTTTCAAAATGGGTCAATCGAGAATATCGTCAACTGTTTACTAAAAATCAGTTTCATCAAGCA
ATGAAACACGCCAAAGTAAACAATTTAAGTACCATTACTTATGAGCAAGTATTGTCTATTTTTAATAGTTATCTATTATTTAAC
GGGAGGAAATAATTCTATGAGTCGCTTTTTTAAATTTGGAAAGTTACACGTTACTAAAGGGAATGGAGATAAATTATTAGATAT
ACTACTGACAGCTTCCAAGAAGCTAAAGAGGTCCCTAGCGCCTACGGGGAATTTGGGGTACATTGAAAAAGGAAGAGTATGAGT
ATTCAACATTTCCGTGTCGCCCTTATTCCCTTTTTTGCGGCATTTTGCCTTCCTGTTTTTGCTCACCCAGAAACGCTGGTGAAA
GTAAAAGATGCTGAAGATCAGTTGGGTGCACGAGTGGGTTACATCGAACTGGATCTCAACAGCGGTAAGATCCTTGAGAGTTTT
CGCCCCGAAGAACGTTTTCCAATGATGAGCACTTTTAAAGTTCTGCTATGTGGCGCGGTATTATCCCGTATTGACGCCGGGCAA
GAGCAACTCGGTCGCCGCATACACTATTCTCAGAATGACTTGGTTGAGTACTCACCAGTCACAGAAAAGCATCTTACGGATGGC
ATGACAGTAAGAGAATTATGCAGTGCTGCCATAACCATGAGTGATAACACTGCGGCCAACTTACTTCTGACAACGATCGGAGGA
CCGAAGGAGCTAACCGCTTTTTTGCACAACATGGGGGATCATGTAACTCGCCTTGATCGTTGGGAACCGGAGCTGAATGAAGCC
ATACCAAACGACGAGCGTGACACCACGATGCCTGTAGCAATGGCAACAACGTTGCGCAAACTATTAACTGGCGAACTACTTACT
CTAGCTTCCCGGCAACAATTAATAGACTGGATGGAGGCGGATAAAGTTGCAGGACCACTTCTGCGCTCGGCCCTTCCGGCTGGC
TGGTTTATTGCTGATAAATCTGGAGCCGGTGAGCGTGGGTCTCGCGGTATCATTGCAGCACTGGGGCCAGATGGTAAGCCCTCC
CGTATCGTAGTTATCTACACGACGGGGAGTCAGGCAACTATGGATGAACGAAATAGACAGATCGCTGAGATAGGTGCCTCACTG
ATTAAGCATTGGTAACTGTCAGACCAAGTTTACTCATATATACTTTAGATTGATTTAAAACTTCATTTTTAATTTAAAAGGATC
TAGGTGAAGATCCTTTTTGATAATCTCATGACCAAAATCCCTTAACGTGAGTTTTCGTTCCACTGAGCGTCAGACCCCGTAGAA
AAGATCAAAGGATCTTC-3'
```

pXyl-Lm-agrBD

```
5'-
TTGAGATCCTTTTTTTCTGCGCGTAATCTGCTGCTTGCAAACAAAAAAACCACCGCTACCAGCGGTGGTTTGTTTGCCGGATCA
AGAGCTACCAACTCTTTTTCCGAAGGTAACTGGCTTCAGCAGAGCGCAGATACCAAATACTGTTCTTCTAGTGTAGCCGTAGTT
AGGCCACCACTTCAAGAACTCTGTAGCACCGCCTACATACCTCGCTCTGCTAATCCTGTTACCAGTGGCTGCTGCCAGTGGCGA
TAAGTCGTGTCTTACCGGGTTGGACTCAAGACGATAGTTACCGGATAAGGCGCAGCGGTCGGGCTGAACGGGGGGTTCGTGCAC
ACAGCCCAGCTTGGAGCGAACGACCTACACCGAACTGAGATACCTACAGCGTGAGCTATGAGAAAGCGCCACGCTTCCCGAAGG
GAGAAAGGCGGACAGGTATCCGGTAAGCGGCAGGGTCGGAACAGGAGAGCGCACGAGGGAGCTTCCAGGGGGAAACGCCTGGTA
TCTTTATAGTCCTGTCGGGTTTCGCCACCTCTGACTTGAGCGTCGATTTTTGTGATGCTCGTCAGGGGGGCGGAGCCTATGGAA
AAACGCCAGCAACGCGGCCTTTTTACGGTTCCTGGCCTTTTGCTGGCCTTTTGCTCACATGTTCTTTCCTGCGTTATCCCCTGA
TTCTGTGGATAACCGTATTACCGCCTTTGAGTGAGCTGATACCGCTCGCCGCAGCCGAACGACCGAGCGCAGCGAGTCAGTGAG
CGAGGAAGCGGAAGAGCGCCCAATACGCAAACCGCCTCTCCCCGCGCGTTGGCCGATTCATTAATGCAGCTGGCACGACAGGTT
TCCCGACTGGAAAGCGGGCAGTGAGCGCAACGCAATTAATGTGAGTTAGCTCACTCATTAGGCACCCCAGGCTTTACACTTTAT
GCTTCCGGCTCGTATGTTGTGTGGAATTGTGAGCGGATAACAATTTCACACAGGAAACAGCTATGACCATGATTACGCCAAGCT
TGCATGCCTGCATTATTTATTTTCGTTTTTTTCTTGCATTTTCACAAATGGACTTTTTGGTTCGTATACAAACATGAAGCAAGC
TTTACTCATAGAAGAATCCGCAACTTTCATGGATTGTTCTTCTAGTTTTCTAGAAAGGAATTTACCAACTGATTTATTCATATT
TTTCATAGTTCCGATACCTCCTTTTCAATAGTTTGTAAGTAAGTGGATTAATACTTATCACCTGAAACAAAGATCCTACCATGA
TTAATGTTTTCATCTCTGCGAATGGTATTAGCAACGCAATTCCCGTTAAAATTAGCGTCCCTATCATCGCTTTTCTTTTTAGTG
TTTTCCGGTGTTCTTCACCGATTAAAGGCAAACTTTCTGTGTCTGCCGGAGCGAATAAAAACATGTTGAGCAGTATAAATCCAA
ATGTACCTAAAACAATCCAATTATTAGAGGGGATATTTTGAAAAACAAATGGCGCAAGCACAAACATCAATAAGCTAATTAACG
TGCAATTCAATGTTTTGGTTGCATGTAATCCAAAAGAATACCGTCTGAGCCATAAATATGACAGATGCACTGTCACTGTTTGCA
GTAAAAGCCCTGTTACTAAGGCGATACCGTATACGAGAGCAAACTTCATGACATTAATTAGAATTATTTCCAGTCCATATTTTA
CTTTTAAATAACCTTCTTCATCATCTTTCCAGCGGTCTTTCGAAATCAACACATCCGCCATTCTTTCTGACAAAGGACTTTTG
CAGTAAAATTACTCAAAGTAGTTCCTCCTTATCTACAGATGCATTTTATTTCATATAGTAAGTACATCACCTATTAGTTTGTTG
TTTAAACAAACTAACTTATTTTCATCTTATATAACCTCGTCAGTATTTTCAATATTTTTTTAGTTTTTTATGAACACATTAGA
TATAATAAAGGGAAGATTCGCTATGTACTATGTTGATACTTAATTTAAAGATTAAACAAATGGAGTGGATGAAGTGGATATCGC
TGATCAAACCTTTGTCAAAAAAGTAAATCAAAAGTTATTATTAAAAGAAATCCTTAAAAAATTCACCTATTTCAAGAGCAAAATT
ATCTGAAATGACTGGATTAAATAAATCAACTGTCTCATCACAGGTAAACACGTTAATGAAAGAAAGTATGGTATTTGAAATAGG
TCAAGGACAATCAAGTGGCGGAAGAAGACCTGTCATGCTTGTTTTTAATAAAAAGGCAGGATACTCCGTTGGAATAGATGTTGG
TGTGGATTATATTAATGGCATTTTAACAGACCTTGAAGGAACAATCGTTCTTGATCAATACCGCCATTTGGAATCCAATTCTCC
AGAAATAACGAAAGACATTTTGATTGATATGATTCATCACTTTATTACGCAAATGCCCCAATCTCCGTACGGGCTTATTGGTAT
AGGTATTTGCGTGCCTGGACTCATTGATAAAGATCAAAAAATTGTTTTCACTCCGAACTCCAACTGGAGAGATATTGACTTAAA
ATCTTCGATACAAGAGAAGTACAATGTGCCTGTTTTTATTGAAAATGAGGCAAATGCTGGCGCATATGGAGAAAAAGTATTTGG
AGCTGCAAAAAATCACGATAACATTATTTACGTAAGTATCAGCACAGGAATAGGGATCGGTGTTATTATCAACAATCATTTATA
TAGAGGAGTAAGCGGCTTCTCTGGAGAAATGGGACATATGACAATAGACTTTAATGGTCCTAAATGCAGTTGCGGAAACCGAGG
ATGCTGGGAATTGTATGCTTCAGAGAAGGCTTTATTAAAATCTCTTCAGACCAAAGAGAAAAAACTGTCCTATCAAGATATCAT
AAACCTCGCCCATCTGAATGATATCGGAACCTTAAATGCATTACAAAATTTTGGATTCTATTTAGGAATAGGCCTTACCAATAT
TCTAAATACTTTCAACCCACAAGCCGTAATTTTAAGAAATAGCATAATTGAATCGCATCCTATGGTTTTAAATTCAATGAGAAG
TGAAGTATCATCAAGGGTTTATTCCCAATTAGGCAATAGCTATGAATTATTGCCATCTTCCTTAGGACAGAATGCACCGGCATT
AGGAATGTCCTCCATTGTGATTGATCATTTTCTGGACATGATTACAATGTAATTTTTTATGGAATGGACAGCTCATCTTTAAAG
```

```
ATGAGTTTTTTTATTCTAGGAGTATTTCTGAAGCAATAGTGACATGGCACCTTCTCATATGAAAAAGGAGTTCTAAAATAGAAA
TCTCCTTTTTCATGTGCAAATTATTTTTCTTTATAACGAAAATATCTAAAGTCGGCCAATTCACTGGCCGTCGTTTTACAACGT
CGTGACTGGGAAAACCCTGGCGTTACCCAACTTAATCGCCTTGCAGCACATCCCCCTTTCGCCAGCTGGCGTAATAGCGAAGAG
GCCCGCACCGATCGCCCTTCCCAACAGTTGCGCAGCCTGAATGGCGAATGGCGCCTGATGCGGTATTTTCTCCTTACGCATCTG
TGCGGTATTTCACACCGCATATGGTGCACTCTCAGTACAATCTGCTCTGATGCCGCATAGTTAAGCCAGCCCCGACACCCGCCA
ACACCCGCTGACGCGCCCTGACGGGCTTGTCTGCTCCCGGCATCCGCTTACAGACAAGCTGTGACCGTCTCCGGGAGCTGCATG
TGTCAGAGGTTTTCACCGTCATCACCGAAACGCGCGAGACGAAAGGGCCTCGTGATACGCCTATTTTTATAGGTTAATGTCATG
ATAATAATGGTTTCTTAGACGTCAGGTGGCACTTTTCGGGGAAATGTGCGCGGAACCCCTATTTGTTTATTTTTCTAAATACAT
TCAAATATGTATCCGCTCATGAGACAATAACCCTGATAAATGCTTCAATAATCCATCCTCCAAAGTTGGAGAGTGAGTTTTATG
TCGCAAATATTAATGTTTCTGGTGAACCTTATCAAATTTTCGTTGATTTAATAGAAACATAGCGGTAAAATTAGCAGTAACTTA
ATAGAACGGAAATGAAAAAAGCCACTCTCATATGCTATTGGCTACCAACCTTTAGCGAGAATGACTTAATCCTGTACAGCCATA
CAGGACTTCGACTTATAAGAGGCGCCAACTTCAAATAAGTTATTTGCCTTGTTTTCGCGAACAAGGCTTATTAGATACACCTAT
TGTACCGTTACTCTACGAATATTTCAAGTAGTAATTACTAGCATTGTCCGTTACTCTACGAATATTTCAAGTAGTAATTACTAG
CATTGTCCGTTACTCTACGAATATTTCAAGTAGTAATTACTAGCATTGTCATATACATAATAAAACGGATATAAAAGGGCGTTT
TCTATACCTAGAAGTCTTGTAAATGTACAGGGCGTTTAGATATAGAGAACGCCCTTTTTGTGTTCCGTTCCAGTGGAAGCTACC
ACTTTAAAAAGATGGTCTAGTGTAGCCAATGCAGGAGAGTACACTCGGATATCAGTTGTCGTTGCATTCAACTGTCTGACGTAA
GCGAGGTAAAGGACACAAGCCTTGCATAAAACAAGCCTACGGGATGTAAATCCTAATAATGATGATAACCAAGACGTTAGCGGC
AAAAAGTGTTGGGGGTTCAAAATAAGACATGATTGTGCGACTGGAGTTAAACAGTTACTCGTAAGCGGCGATCATGACACTGAT
TCACGGCTATTCTTGTACAAGCTAGCTTTATTACAAGGATATGCGGGTTATATAGCGAATCACCCGAAAGGGAACGGTGTTGGG
CGTGAGAAACGCACCGTACGGCGCAATACAATGCCAATAAGCTATATACGGACGGTATAGTAGTTTTGTAAGCTATAACCGTTT
GTCGTCAATGCAACCAATCTCAATTCGAGACCTCGGCATCTAAGCCAGTACGAATGAGTGGGCGTTTTAACCTCGTAAATTTTC
AACAGGGGTTACTATGCCCAAAACTACATTCAGATTTCCTAACAAACTCGCCAGTATGAAAACCTTAAGACCTTAAAGTCAAGG
GATTTGAAGGATTTTAACCTCGATTAGCAAAAAATGTAGAGTACTGAAGCAACTACCATTAACTAAGATAGTGGGGGATTGAGG
AAGAATCCAGAGCTGTTTAAATCAAGTGAAAGACAAGATGAAATTAAAAGAATAGTGAAAGATAGGGGAGTGGTTCTCTATGAG
AAAGGAAATGGCTAGAGAACAAAGGCAGCGGTTTATTGATCTATTGTTAGACTTTATGGTAAAGAATCCTCATTTATTTGTTAA
TGGTACAGAGGATGAAAGTAATAATGTTGTTACAAAATGTAATAGTGATATTAAAGAGGTTGCGGAGTCATATTTAACTCTTTT
ATAGTGAGAGGGTTAAAACTAATTAATATGTATTAAGGCCCAATGTTGGAATTATTGTATTTCACTAGGCAACCTACTTACTAA
AAGTAAGATTATCCATTAGTGGATGTTATAATATTGGGTTTTTTAACACAATAATCATCGCCTTTCGGTGTCGTTTGATAGAAA
AGTAACCATTAGCGATGAAAAAGTCAATATAAAAAGCCATCCGTAAAAAACGGATGGCTTACCGTACATAGGATCGTTGGTAGG
GCGGCGTATCCTACATCTCTGGTAACTTACCTAGCCAATCAAATGCTTGAGAACGGCGGTTAGATAAGCGCGTGGGGAACCTTT
CCCACCTCAAAGATCCTATATCATTATTATGTTACTTTCTACAGGTAGTATACCATGTTCTTATATTTTAGTAAACTCCCCGTT
AGCTTAACAGGTCTTTGTAAGCAATTAAACGTCCACTATTCAATCGTCTTTGGATTTTCGCAGGACCGTTTTTTAGATCGAACA
TAGTTGATAAGAACAAATAACCGCTTGGGTCCAACTTTATAGCAATTAGTATATGGTCATTTAAAATCTTTACCAATTCAACGC
TATTAGGTTCTTTAGGATTTTGCCCGACATAGTCGGGGTGTTCAACGATATCTTTTATGTGCGATGAATATTTTTCATAAATAC
CAGGATGTTGTTTCTTTACGTGCTTTATAAATCCGGGAAACATTTTTACATCGTTAGAAGTGCAAGTCAAGTTATATGTATCTA
TAATGATTTGTGGAAGTTTTGCCACAACAGTTGGTTTATTTACAATCTTTTTTTTATTAGCCGTCAAATTTCTCCCTCATCTCG
TCTCTTTATATCTTTATTTTATCATAAAGGAGTATTTGAACCGTCGCGCGGGACAGGTTTATGATAGGGATATTTTATTGAATA
ATTGATGGTATAAGGGACTTTCATGCTTGGAAAGTGGGGATTATGAATTAGATGCTTGTCCACAATATGTTCCAATGTAATTAA
AATTTATGTTCCCACCTTGACCAAACATCACGTCCATACTTAAATCGTCCCTCCTTTAATAGGTAAAATATTAATTTACCTTAA
TAAAAAAATAATGGATAATAGTATTCGTCTGAATTTATATAATCAGGGGGAACTATTGATGCTGGGGATACTATTTACAGCGGC
GCCATCTACTGATGTCGTAAAGGATTTGCAAGATAAAGTTATATCATTGCAGGATCATGAGGTAGCGTTTTTGAACACCACGAT
ATCTAATATGTTGATCCCCGAAGCAAACTTAAGAGTGTGTTGATAGTGCAGTATCTTAAAATTTTGTGTATAATAGGAATTGAA
GTTAAATTAGATGCTAAAAATTTGTAATTAAGAAGGAGGGATTCGTCATGTTGGTATTCCAAATGCGTAATGTAGATAAAACAT
CTACTGTTTTGAAACAGACTAAAAACAGTGATTACGCAGATAAATAAATACGTTAGATTAATTCCTACCAGTGACTAATCTTAT
GACTTTTTAAACAGATAACTAAAATTACAAACAAATCGTTTAACTTCTGTATTTATTTACAGATGTAATCACTTCAGGAGTAAT
TACATGAACAAAAATATAAAATATTCTCAAAACTTTTTAACGAGTGAAAAAGTACTCAACCAAATAATAAAACAATTGAATTTA
AAAGAAACCGATACCGTTTACGAAATTGGAACAGGTAAAGGGCATTTAACGACGAAACTGGCTAAAATAAGTAAACAGGTAACG
TCTATTGAATTAGACAGTCATCTATTCAACTTATCGTCAGAAAAATTAAAACTGAACATTCGTGTCACTTTAATTCACCAAGAT
ATTCTACAGTTTCAATTCCCTAACAAACAGAGGTATAAAATTGTTGGGAGTATTCCTTACCATTTAAGCACACAAATTATTAAA
AAAGTGGTTTTTGAAAGCCATGCGTCTGACATCTATCTGATTGTTGAAGAAGGATTCTACAAGCGTACCTTGGATATTCACCGA
ACACTAGGGTTGCTCTTGCACACTCAAGTCTCGATTCAGCAATTGCTTAAGCTGCCAGCGGAATGCTTTCATCCTAAACCAAAA
GTAAACAGTGTCTTAATAAAACTTACCCGCCATACCACAGATGTTCCAGATAAATATTGGAAGCTATATACGTACTTTGTTTCA
AAATGGGTCAATCGAGAATATCGTCAACTGTTTACTAAAAATCAGTTTCATCAAGCAATGAAACACGCCAAAGTAAACAATTTA
AGTACCATTACTTATGAGCAAGTATTGTCTATTTTTAATAGTTATCTATTATTTAACGGGAGGAAATAATTCTATGAGTCGCTT
TTTTAAATTTGGAAAGTTACACGTTACTAAAGGGAATGGAGATAAATTATTAGATATACTACTGACAGCTTCCAAGAAGCTAAA
GAGGTCCCTAGCGCCTACGGGGAATTTGGGGTACATTGAAAAAGGAAGAGTATGAGTATTCAACATTTCCGTGTCGCCCTTATT
CCCTTTTTTGCGGCATTTTGCCTTCCTGTTTTTGCTCACCCAGAAACGCTGGTGAAAGTAAAAGATGCTGAAGATCAGTTGGGT
GCACGAGTGGGTTACATCGAACTGGATCTCAACAGCGGTAAGATCCTTGAGAGTTTTCGCCCCGAAGAACGTTTTCCAATGATG
AGCACTTTTAAAGTTCTGCTATGTGGCGCGGTATTATCCCGTATTGACGCCGGGCAAGAGCAACTCGGTCGCCGCATACACTAT
TCTCAGAATGACTTGGTTGAGTACTCACCAGTCACAGAAAAGCATCTTACGGATGGCATGACAGTAAGAGAATTATGCAGTGCT
GCCATAACCATGAGTGATAACACTGCGGCCAACTTACTTCTGACAACGATCGGAGGACCGAAGGAGCTAACCGCTTTTTTGCAC
AACATGGGGGATCATGTAACTCGCCTTGATCGTTGGGAACCGGAGCTGAATGAAGCCATACCAAACGACGAGCGTGACACCACG
ATGCCTGTAGCAATGGCAACAACGTTGCGCAAACTATTAACTGGCGAACTACTTACTCTAGCTTCCCGGCAACAATTAATAGAC
```

```
TGGATGGAGGCGGATAAAGTTGCAGGACCACTTCTGCGCTCGGCCCTTCCGGCTGGCTGGTTTATTGCTGATAAATCTGGAGCC
GGTGAGCGTGGGTCTCGCGGTATCATTGCAGCACTGGGGCCAGATGGTAAGCCCTCCCGTATCGTAGTTATCTACACGACGGGG
AGTCAGGCAACTATGGATGAACGAAATAGACAGATCGCTGAGATAGGTGCCTCACTGATTAAGCATTGGTAACTGTCAGACCAA
GTTTACTCATATATACTTTAGATTGATTTAAAACTTCATTTTTAATTTAAAAGGATCTAGGTGAAGATCCTTTTTGATAATCTC
ATGACCAAAATCCCTTAACGTGAGTTTTCGTTCCACTGAGCGTCAGACCCCGTAGAAAAGATCAAAGGATCTTC-3’
```

pXyl-Lm-agrCA-GFP

```
5’-
TTGAGATCCTTTTTTTCTGCGCGTAATCTGCTGCTTGCAAACAAAAAAACCACCGCTACCAGCGGTGGTTTGTTTGCCGGATCA
AGAGCTACCAACTCTTTTTCCGAAGGTAACTGGCTTCAGCAGAGCGCAGATACCAAATACTGTTCTTCTAGTGTAGCCGTAGTT
AGGCCACCACTTCAAGAACTCTGTAGCACCGCCTACATACCTCGCTCTGCTAATCCTGTTACCAGTGGCTGCTGCCAGTGGCGA
TAAGTCGTGTCTTACCGGGTTGGACTCAAGACGATAGTTACCGGATAAGGCGCAGCGGTCGGGCTGAACGGGGGGTTCGTGCAC
ACAGCCCAGCTTGGAGCGAACGACCTACACCGAACTGAGATACCTACAGCGTGAGCTATGAGAAAGCGCCACGCTTCCCGAAGG
GAGAAAGGCGGACAGGTATCCGGTAAGCGGCAGGGTCGGAACAGGAGAGCGCACGAGGGAGCTTCCAGGGGGAAACGCCTGGTA
TCTTTATAGTCCTGTCGGGTTTCGCCACCTCTGACTTGAGCGTCGATTTTTGTGATGCTCGTCAGGGGGGCGGAGCCTATGGAA
AAACGCCAGCAACGCGGCCTTTTTACGGTTCCTGGCCTTTTGCTGGCCTTTTGCTCACATGTTCTTTCCTGCGTTATCCCCTGA
TTCTGTGGATAACCGTATTACCGCCTTTGAGTGAGCTGATACCGCTCGCCGCAGCCGAACGACCGAGCGCAGCGAGTCAGTGAG
CGAGGAAGCGGAAGAGCGCCCAATACGCAAACCGCCTCTCCCCGCGCGTTGGCCGATTCATTAATGCAGCTGGCACGACAGGTT
TCCCGACTGGAAAGCGGGCAGTGAGCGCAACGCAATTAATGTGAGTTAGCTCACTCATTAGGCACCCCAGGCTTTACACTTTAT
GCTTCCGGCTCGTATGTTGTGTGAATTGTGAGCGGATAACAATTTCACACAGGAAACAGCTATGGCCATGATTACGCCAAGCTT
GCATGCCTGCATTATAAACTCAAGCTTTTAATTAATTTCGATGATGCATAACAATTTTCACCGTTAGACATCTTCACCACACCT
TTTGTCGTATCTAGCTCATGAATATTTTTCTTATTCACAATATACGACCTGTGGCACCGATAAAATGATTCGTCCAACATTTTC
TCGATATTCTTCAACTTACCATAAAATTCCACTTGGCGATTTTTGCCATGTAAAATTACTTTATGAATAGTTGGCGCTGTCTCA
AAAAACAAGATATCGTCTAACAGTTCATGAATAATCTTCTTATCCGACACTTTAAACGTAAAATACTTCTGCATATCTTGATCA
TTGGAAATCCGTTCTTCTGCTTGCTTCATACAGGCAAGTACCCGGTCATGCAGCAAATCAATATCGTCTTTAATAATGTAATCA
AGCGCTTCCACCTTATACGTGAAAGTCATATAACTTAACTCCGCATGTGTTGTAATAAAAATAATGAAACCACGCGGATCAAAC
TTCCGAATTTCCTGAGCTAATTCAAAACCATTCATGTCCGGCTGCCCCAAATCAATGTCTAAAAAATAAAGCCCCATACCTTGA
TGTGTAGGCATTCGTGATACTAACTCAAACGGATCTCCTGTTGAAAGTTCTAACTTCATATCAAAATGTTCAACCATAATATAG
TCTTCAATATATTTCGTTAACCTTTCTCGCTGCATTCTGTTATCTTCACAAATAAAAACCGGTAGCATAAATTCATCCCCATTC
TTCTACATAATTTCTAATTCTTGAATAACTTCTCTATCGGTCACTTTCGTATCTAAGGCAACGTGCGAATATTTCTTCATAATT
TCCCGCAAACTAGCAAGGCCTAATCCACGACCTTCTCCTTTTGTAGAGAAACCTTCTTCAAATATCTTATAAATCGGTGGCATA
TTTACTGGTAAACTATTCGCAAATACAATAATTATGCTATCACCTTTTTTCACAAACGCGATTCGAATAACTGGATTTTCACAA
GTTAACGCCGCTTCAACCGCATTATCTAGCAAAATACCGACAACTTTGCATAAATCTATACTATCCATCGAAATCTTGTCAATC
GGTTCAACTACTTCCAAAATTGCATCAATTTTCAACTCTTGAGCTCGTATTAATTTAACAGCTAGCAAGCCCTTTAGTTCAATA
ACATGAATATTTTGAAGCAATGAAATCTTATAGTTGTTCGATTCAATTGTTTTATTTATAGGTACAATATTATTTTCAAAGTAA
TACTTCAAACCCGGCATATCATTATTATCAATATATCCAACAAGTGTTGACAGGATATTCACATAATCATGACGAAAAACGCGC
ATTTCTCTATGCAGGGACTCTAATGTAGTAACATAATCCTGTAACTGTTCTAGCTGTTCCTTCTGATTCTGTACTTTAAGTTCG
TTGGTTGCCGTATTAATAACAACCGTCACTATAACTATTAACAAAATGGTATATCCTGTAAAAATAAGCGTATTAATTTTCAAA
ACTGATCCATCAAAACCTGCGATTGACCCCGCGTATATGTTCATATAGAACGCTAAGACAGTAAGAGCAACGATAGAAAAAATA
ATATACGCATATTTTCTATGTTCAACAAATCTAGAAATATTAACTTTTTCAATCAATTTTCTAAGAAGAAACGAAAATACCAAC
AAATTCGCCAACATTCCCGCGCAGTAAATTAACGTTGGTAACAGTTCATTAAATATCTCATCATATTTAAAGTTCAAGCCCGGT
ACTAAAATAAAACCTACTATAGAGTCACTAATAGTAAGTAAAATAATAACCACAAGCGTAATAGAAGCTGAAACTACTACATTT
TTATTTTTCCAATATAAGGCACTTACAAAAACAATCAATACAAAAATCATCGACCAGTATTGAACTAAAGTAAATAAAGGGAAG
GCTAGCATAGCAATTGCTATAGTAACCAATCCCTCTTTAATTGAAAAAACTTTGTTTGTTAAAATCTGGATTGCAATAAAAATA
CCCGTTATCTGTATAATTGCCATCAAAATACTAAACATAGTAGTTCCTCCTTATCTACAGATGCATTTTATTTCATATAGTAAG
TACATCACCTATTAGTTTGTTGTTTAAACAAACTAACTTATTTTCATCTTATATAACCTCGTCAGTATTTTCAATATTTTTTTT
AGTTTTTTATGAACACATTAGATATAATAAAGGGAAGATTCGCTATGTACTATGTTGATACTTAATTTAAAGATTAAACAAATG
GAGTGGATGAAGTGGATATCGCTGATCAAACCTTTGTCAAAAAAGTAAATCAAAAGTTATTATTAAAAGAAATCCTTAAAAATT
CACCTATTTCAAGAGCAAAATTATCTGAAATGACTGGATTAAATAAATCAACTGTCTCATCACAGGTAAACACGTTAATGAAAG
AAAGTATGGTATTTGAAATAGGTCAAGGACAATCAAGTGGCGGAAGAAGACCTGTCATGCTTGTTTTTAATAAAAAGGCAGGAT
ACTCCGTTGGAATAGATGTTGGTGTGGATTATATTAATGGCATTTTAACAGACCTTGAAGGAACAATCGTTCTTGATCAATACC
GCCATTTGGAATCCAATTCTCCAGAAATAACGAAAGACATTTTGATTGATATGATTCATCACTTTATTACGCAAATGCCCCAAT
CTCCGTACGGGCTTATTGGTATAGGTATTTGCGTGCCTGGACTCATTGATAAAGATCAAAAAATTGTTTTCACTCCGAACTCCA
ACTGGAGAGATATTGACTTAAAATCTTCGATACAAGAGAAGTACAATGTGCCTGTTTTTATTGAAAATGAGGCAAATGCTGGCG
CATATGGAGAAAAAGTATTTGGAGCTGCAAAAAATCACGATAACATTATTTACGTAAGTATCAGCACAGGAATAGGGATCGGTG
TTATTATCAACAATCATTTATATAGAGGAGTAAGCGGCTTCTCTGGAGAAATGGGACATATGACAATAGACTTTAATGGTCCTA
AATGCAGTTGCGGAAACCGAGGATGCTGGGAATTGTATGCTTCAGAGAAGGCTTTATTAAAATCTCTTCAGACCAAAGAGAAAA
AACTGTCCTATCAAGATATCATAAACCTCGCCCATCTGAATGATATCGGAACCTTAAATGCATTACAAAATTTTGGATTCTATT
TAGGAATAGGCCTTACCAATATTCTAAATACTTTCAACCCACAAGCCGTAATTTTAAGAAATAGCATAATTGAATCGCATCCTA
TGGTTTTAAATTCAATGAGAAGTGAAGTATCATCAAGGGTTTATTCCCAATTAGGCAATAGCTATGAATTATTGCCATCTTCCT
TAGGACAGAATGCACCGGCATTAGGAATGTCCTCCATTGTGATTGATCATTTTCTGGACATGATTACAATGTAATTTTTTATGG
AATGGACAGCTCATCTTTAAAGATGAGTTTTTTTATTCTAGGAGTATTTCTGAAGCAATAGTGACATGGCACCTTCTCATATGA
```

```
AAAAGGAGTTCTAAAATAGAAATCTCCTTTTTCATGTGCAAATTATTTTTCTTTATAACGAAAATATCTAAAGTCGGCCAATTC
ACTGGCCGTCGTTTTACCGAAATACGGGCAGACATGGCCTGCCCGGTTAATGCCTGGCTCTTCAAACAGAACAAGACGATTAAT
AAAATGGAAAAGCCAACTGTGAACATTCTTTTGCAGTTGGTTTTTTTTATGAATAAACTCACTTAAATAAAATATTAATATGGG
TTTTTAAGCCTATTATACAGTCCTTTTTACACGTTCGAATATTCTGAAATGTATCCAAAAAATCGGCAATTCTGCTCTTTTTTG
GTCGTTCATGACAAGAATCGGACATTTCATTACATTTTTGGTTATTATGGGTAAATTCGTTGTAAAATATTAGTGGAGGTGAAT
TAGGATCACAGAGATGTGGTCAGTCAGTCTTAATTAACCAAGGAGGTGAAATGTACAATGAAAGGAGAAGAACTTTTCACTGGA
GTTGTCCCAATTCTTGTTGAATTAGATGGTGATGTTAATGGGCACAAATTTTCTGTCAGTGGAGAGGGTGAAGGTGATGCAACA
TACGGAAAACTTACCCTTAAATTTATTTGCACTACTGGAAAACTACCTGTTCCATGGCCAACACTTGTCACTACTTTCGCGTAT
GGTCTTCAATGCTTTGCGAGATACCCAGATCATATGAAACAGCATGACTTTTTCAAGAGTGCCATGCCCGAAGGTTATGTACAG
GAAAGAACTATATTTTTCAAAGATGACGGGAACTACAAGACACGTGCTGAAGTCAAGTTTGAAGGTGATACCCTTGTTAATAGA
ATCGAGTTAAAAGGTATTGATTTTAAAGAAGATGGAAACATTCTTGGACACAAATTGGAATACAACTATAACTCACACAATGTA
TACATCATGGCAGACAAACAAAAGAATGGAATCAAAGTTAACTTCAAAATTAGACACAACATTGAAGATGGAAGCGTTCAACTA
GCAGACCATTATCAACAAAATACTCCAATTGGCGATGGCCCTGTCCTTTTACCAGACAACCATTACCTGTCCACACAATCTAAG
CTTTCGAAAGATCCCAACGAAAAGAGAGACCACATGGTCCTTCTTGAGTTTGTAACAGCTGCTGGGATTACACATGGCATGGAT
GAACTATACAAATAACCTAGGCTGCTGCCACCGCTGAGCAATAACTAGCATAACCCCTTGGGGCCTCTAAACGGGTCTTGAGGG
GTTTTTTGAACGTCGTGACTGGGAAAACCCTGGCGTTACCCAACTTAATCGCCTTGCAGCACATCCCCCTTTCGCCAGCTGGCG
TAATAGCGAAGAGGCCCGCACCGATCGCCCTTCCCAACAGTTGCGCAGCCTGAATGGCGAATGGCGCCTGATGCGGTATTTTCT
CCTTACGCATCTGTGCGGTATTTCACACCGCATATGGTGCACTCTCAGTACAATCTGCTCTGATGCCGCATAGTTAAGCCAGCC
CCGACACCCGCCAACACCCGCTGACGCGCCCTGACGGGCTTGTCTGCTCCCGGCATCCGCTTACAGACAAGCTGTGACCGTCTC
CGGGAGCTGCATGTGTCAGAGGTTTTCACCGTCATCACCGAAACGCGCGAGACGAAAGGGCCTCGTGATACGCCTATTTTTATA
GGTTAATGTCATGATAATAATGGTTTCTTAGACGTCAGGTGGCACTTTTCGGGGAAATGTGCGCGGAACCCCTATTTGTTTATT
TTTCTAAATACATTCAAATATGTATCCGCTCATGAGACAATAACCCTGATAAATGCTTCAATAATCCATCCTCCAAAGTTGGAG
AGTGAGTTTTATGTCGCAAATATTAATGTTTCTGGTGAACCTTATCAAATTTTCGTTGATTTAATAGAAACATAGCGGTAAAAT
TAGCAGTAACTTAATAGAACGGAAATGAAAAAAGCCACTCTCATATGCTATTGGCTACCAACCTTTAGCGAGAATGACTTAATC
CTGTACAGCCATACAGGACTTCGACTTATAAGAGGCGCCAACTTCAAATAAGTTATTTGCCTTGTTTTCGCGAACAAGGCTTAT
TAGATACACCTATTGTACCGTTACTCTACGAATATTTCAAGTAGTAATTACTAGCATTGTCCGTTACTCTACGAATATTTCAAG
TAGTAATTACTAGCATTGTCCGTTACTCTACGAATATTTCAAGTAGTAATTACTAGCATTGTCATATACATAATAAAACGGATA
TAAAAGGGCGTTTTCTATACCTAGAAGTCTTGTAAATGTACAGGGCGTTTAGATATAGAGAACGCCCTTTTTGTGTTCCGTTCC
AGTGGAAGCTACCACTTTAAAAAGATGGTCTAGTGTAGCCAATGCAGGAGAGTACACTCGGATATCAGTTGTCGTTGCATTCAA
CTGTCTGACGTAAGCGAGGTAAAGGACACAAGCCTTGCATAAAACAAGCCTACGGGATGTAAATCCTAATAATGATGATAACCA
AGACGTTAGCGGCAAAAAGTGTTGGGGGTTCAAAATAAGACATGATTGTGCGACTGGAGTTAAACAGTTACTCGTAAGCGGCGA
TCATGACACTGATTCACGGCTATTCTTGTACAAGCTAGCTTTATTACAAGGATATGCGGGTTATATAGCGAATCACCCGAAAGG
GAACGGTGTTGGGCGTGAGAAACGCACCGTACGGCGCAATACAATGCCAATAAGCTATATACGGACGGTATAGTAGTTTTGTAA
GCTATAACCGTTTGTCGTCAATGCAACCAATCTCAATTCGAGACCTCGGCATCTAAGCCAGTACGAATGAGTGGGCGTTTTAAC
CTCGTAAATTTTCAACAGGGGTTACTATGCCCAAAACTACATTCAGATTTCCTAACAAACTCGCCAGTATGAAAACCTTAAGAC
CTTAAAGTCAAGGGATTTGAAGGATTTTAACCTCGATTAGCAAAAAATGTAGAGTACTGAAGCAACTACCATTAACTAAGATAG
TGGGGGATTGAGGAAGAATCCAGAGCTGTTTAAATCAAGTGAAAGACAAGATGAAATTAAAAGAATAGTGAAAGATAGGGGAGT
GGTTCTCTATGAGAAAGGAAATGGCTAGAGAACAAAGGCAGCGGTTTATTGATCTATTGTTAGACTTTATGGTAAAGAATCCTC
ATTTATTTGTTAATGGTACAGAGGATGAAAGTAATAATGTTGTTACAAAATGTAATAGTGATATTAAAGAGGTTGCGGAGTCAT
ATTTAACTCTTTTATAGTGAGAGGGTTAAAACTAATTAATATGTATTAAGGCCCAATGTTGGAATTATTGTATTTCACTAGGCA
ACCTACTTACTAAAAGTAAGATTATCCATTAGTGGATGTTATAATATTGGGTTTTTTAACACAATAATCATCGCCTTTCGGTGT
CGTTTGATAGAAAAGTAACCATTAGCGATGAAAAAGTCAATATAAAAAGCCATCCGTAAAAAACGGATGGCTTACCGTACATAG
GATCGTTGGTAGGGCGGCGTATCCTACATCTCTGGTAACTTACCTAGCCAATCAAATGCTTGAGAACGGCGGTTAGATAAGCGC
GTGGGGAACCTTTCCCACCTCAAAGATCCTATATCATTATTATGTTACTTTCTACAGGTAGTATACCATGTTCTTATATTTTAG
TAAACTCCCCGTTAGCTTAACAGGTCTTTGTAAGCAATTAAACGTCCACTATTCAATCGTCTTTGGATTTTCGCAGGACCGTTT
TTTAGATCGAACATAGTTGATAAGAACAAATAACCGCTTGGGTCCAACTTTATAGCAATTAGTATATGGTCATTTAAAATCTTT
ACCAATTCAACGCTATTAGGTTCTTTAGGATTTTGCCCGACATAGTCGGGGTGTTCAACGATATCTTTTATGTGCGATGAATAT
TTTTCATAAATACCAGGATGTTGTTTCTTTACGTGCTTTATAAATCCGGGAAACATTTTTACATCGTTAGAAGTGCAAGTCAAG
TTATATGTATCTATAATGATTTGTGGAAGTTTTGCCACAACAGTTGGTTTATTTACAATCTTTTTTTTATTAGCCGTCAAATTT
CTCCCTCATCTCGTCTCTTTATATCTTTATTTTATCATAAAGGAGTATTTGAACCGTCGCGCGGGACAGGTTTATGATAGGGAT
ATTTTATTGAATAATTGATGGTATAAGGGACTTTCATGCTTGGAAAGTGGGGATTATGAATTAGATGCTTGTCCACAATATGTT
CCAATGTAATTAAAATTTATGTTCCCACCTTGACCAAACATCACGTCCATACTTAAATCGTCCCTCCTTTAATAGGTAAAATAT
TAATTTACCTTAATAAAAAAATAATGGATAATAGTATTCGTCTGAATTTATATAATCAGGGGGAACTATTGATGCTGGGGATAC
TATTTACAGCGGCGCCATCTACTGATGTCGTAAAGGATTTGCAAGATAAAGTTATATCATTGCAGGATCATGAGGTAGCGTTTT
TGAACACCACGATATCTAATATGTTGATCCCCGAAGCAAACTTAAGAGTGTGTTGATAGTGCAGTATCTTAAAATTTTGTGTAT
AATAGGAATTGAAGTTAAATTAGATGCTAAAAATTTGTAATTAAGAAGGAGGGATTCGTCATGTTGGTATTCCAAATGCGTAAT
GTAGATAAAACATCTACTGTTTTGAAACAGACTAAAAACAGTGATTACGCAGATAAATAAATACGTTAGATTAATTCCTACCAG
TGACTAATCTTATGACTTTTTAAACAGATAACTAAAATTACAAACAAATCGTTTAACTTCTGTATTTATTTACAGATGTAATCA
CTTCAGGAGTAATTACATGAACAAAAATATAAAATATTCTCAAAACTTTTTAACGAGTGAAAAAGTACTCAACCAAATAATAAA
ACAATTGAATTTAAAAGAAACCGATACCGTTTACGAAATTGGAACAGGTAAAGGGCATTTAACGACGAAACTGGCTAAAATAAG
TAAACAGGTAACGTCTATTGAATTAGACAGTCATCTATTCAACTTATCGTCAGAAAAATTAAAACTGAACATTCGTGTCACTTT
AATTCACCAAGATATTCTACAGTTTCAATTCCCTAACAAACAGAGGTATAAAATTGTTGGGAGTATTCCTTACCATTTAAGCAC
ACAAATTATTAAAAAAGTGGTTTTTGAAAGCCATGCGTCTGACATCTATCTGATTGTTGAAGAAGGATTCTACAAGCGTACCTT
```

```
GGATATTCACCGAACACTAGGGTTGCTCTTGCACACTCAAGTCTCGATTCAGCAATTGCTTAAGCTGCCAGCGGAATGCTTTCA
TCCTAAACCAAAAGTAAACAGTGTCTTAATAAAACTTACCCGCCATACCACAGATGTTCCAGATAAATATTGGAAGCTATATAC
GTACTTTGTTTCAAAATGGGTCAATCGAGAATATCGTCAACTGTTTACTAAAAATCAGTTTCATCAAGCAATGAAACACGCCAA
AGTAAACAATTTAAGTACCATTACTTATGAGCAAGTATTGTCTATTTTTAATAGTTATCTATTATTTAACGGGAGGAAATAATT
CTATGAGTCGCTTTTTTAAATTTGGAAAGTTACACGTTACTAAAGGGAATGGAGATAAATTATTAGATATACTACTGACAGCTT
CCAAGAAGCTAAAGAGGTCCCTAGCGCCTACGGGGAATTTGGGGTACATTGAAAAAGGAAGAGTATGAGTATTCAACATTTCCG
TGTCGCCCTTATTCCCTTTTTTGCGGCATTTTGCCTTCCTGTTTTTGCTCACCCAGAAACGCTGGTGAAAGTAAAAGATGCTGA
AGATCAGTTGGGTGCACGAGTGGGTTACATCGAACTGGATCTCAACAGCGGTAAGATCCTTGAGAGTTTTCGCCCCGAAGAACG
TTTTCCAATGATGAGCACTTTTAAAGTTCTGCTATGTGGCGCGGTATTATCCCGTATTGACGCCGGGCAAGAGCAACTCGGTCG
CCGCATACACTATTCTCAGAATGACTTGGTTGAGTACTCACCAGTCACAGAAAAGCATCTTACGGATGGCATGACAGTAAGAGA
ATTATGCAGTGCTGCCATAACCATGAGTGATAACACTGCGGCCAACTTACTTCTGACAACGATCGGAGGACCGAAGGAGCTAAC
CGCTTTTTTGCACAACATGGGGGATCATGTAACTCGCCTTGATCGTTGGGAACCGGAGCTGAATGAAGCCATACCAAACGACGA
GCGTGACACCACGATGCCTGTAGCAATGGCAACAACGTTGCGCAAACTATTAACTGGCGAACTACTTACTCTAGCTTCCCGGCA
ACAATTAATAGACTGGATGGAGGCGGATAAAGTTGCAGGACCACTTCTGCGCTCGGCCCTTCCGGCTGGCTGGTTTATTGCTGA
TAAATCTGGAGCCGGTGAGCGTGGGTCTCGCGGTATCATTGCAGCACTGGGGCCAGATGGTAAGCCCTCCCGTATCGTAGTTAT
CTACACGACGGGGAGTCAGGCAACTATGGATGAACGAAATAGACAGATCGCTGAGATAGGTGCCTCACTGATTAAGCATTGGTA
ACTGTCAGACCAAGTTTACTCATATATACTTTAGATTGATTTAAAACTTCATTTTTAATTTAAAAGGATCTAGGTGAAGATCCT
TTTTGATAATCTCATGACCAAAATCCCTTAACGTGAGTTTTCGTTCCACTGAGCGTCAGACCCCGTAGAAAAGATCAAAGGATC
TTC-3’
```

pXyl-Lm-agrBDCA-GFP

```
5’-
TTGAGATCCTTTTTTTCTGCGCGTAATCTGCTGCTTGCAAACAAAAAAACCACCGCTACCAGCGGTGGTTTGTTTGCCGGATCA
AGAGCTACCAACTCTTTTTCCGAAGGTAACTGGCTTCAGCAGAGCGCAGATACCAAATACTGTTCTTCTAGTGTAGCCGTAGTT
AGGCCACCACTTCAAGAACTCTGTAGCACCGCCTACATACCTCGCTCTGCTAATCCTGTTACCAGTGGCTGCTGCCAGTGGCGA
TAAGTCGTGTCTTACCGGGTTGGACTCAAGACGATAGTTACCGGATAAGGCGCAGCGGTCGGGCTGAACGGGGGGTTCGTGCAC
ACAGCCCAGCTTGGAGCGAACGACCTACACCGAACTGAGATACCTACAGCGTGAGCTATGAGAAAGCGCCACGCTTCCCGAAGG
GAGAAAGGCGGACAGGTATCCGGTAAGCGGCAGGGTCGGAACAGGAGAGCGCACGAGGGAGCTTCCAGGGGGAAACGCCTGGTA
TCTTTATAGTCCTGTCGGGTTTCGCCACCTCTGACTTGAGCGTCGATTTTTGTGATGCTCGTCAGGGGGGCGGAGCCTATGGAA
AAACGCCAGCAACGCGGCCTTTTTACGGTTCCTGGCCTTTTGCTGGCCTTTTGCTCACATGTTCTTTCCTGCGTTATCCCCTGA
TTCTGTGGATAACCGTATTACCGCCTTTGAGTGAGCTGATACCGCTCGCCGCAGCCGAACGACCGAGCGCAGCGAGTCAGTGAG
CGAGGAAGCGGAAGAGCGCCCAATACGCAAACCGCCTCTCCCCGCGCGTTGGCCGATTCATTAATGCAGCTGGCACGACAGGTT
TCCCGACTGGAAAGCGGGCAGTGAGCGCAACGCAATTAATGTGAGTTAGCTCACTCATTAGGCACCCCAGGCTTTACACTTTAT
GCTTCCGGCTCGTATGTTGTGTGAATTGTGAGCGGATAACAATTTCACACAGGAAACAGCTATGGCCATGATTACGCCAAGCTT
GCATGCCTGCATTATAAACTCAAGCTTTTAATTAATTTCGATGATGCATAACAATTTTCACCGTTAGACATCTTCACCACACCT
TTTGTCGTATCTAGCTCATGAATATTTTTCTTATTCACAATATACGACCTGTGGCACCGATAAAATGATTCGTCCAACATTTTC
TCGATATTCTTCAACTTACCATAAAATTCCACTTGGCGATTTTTGCCATGTAAAATTACTTTATGAATAGTTGGCGCTGTCTCA
AAAAACAAGATATCGTCTAACAGTTCATGAATAATCTTCTTATCCGACACTTTAAACGTAAAATACTTCTGCATATCTTGATCA
TTGGAAATCCGTTCTTCTGCTTGCTTCATACAGGCAAGTACCCGGTCATGCAGCAAATCAATATCGTCTTTAATAATGTAATCA
AGCGCTTCCACCTTATACGTGAAAGTCATATAACTTAACTCCGCATGTGTTGTAATAAAAATAATGAAACCACGCGGATCAAAC
TTCCGAATTTCCTGAGCTAATTCAAAACCATTCATGTCCGGCTGCCCCAAATCAATGTCTAAAAAATAAAGCCCCATACCTTGA
TGTGTAGGCATTCGTGATACTAACTCAAACGGATCTCCTGTTGAAAGTTCTAACTTCATATCAAAATGTTCAACCATAATATAG
TCTTCAATATATTTCGTTAACCTTTCTCGCTGCATTCTGTTATCTTCACAAATAAAAACCGGTAGCATAAATTCATCCCCATTC
TTCTACATAATTTCTAATTCTTGAATAACTTCTCTATCGGTCACTTTCGTATCTAAGGCAACGTGCGAATATTTCTTCATAATT
TCCCGCAAACTAGCAAGGCCTAATCCACGACCTTCTCCTTTTGTAGAGAAACCTTCTTCAAATATCTTATAAATCGGTGGCATA
TTTACTGGTAAACTATTCGCAAATACAATAATTATGCTATCACCTTTTTTCACAAACGCGATTCGAATAACTGGATTTTCACAA
GTTAACGCCGCTTCAACCGCATTATCTAGCAAAATACCGACAACTTTGCATAAATCTATACTATCCATCGAAATCTTGTCAATC
GGTTCAACTACTTCCAAAATTGCATCAATTTTCAACTCTTGAGCTCGTATTAATTTAACAGCTAGCAAGCCCTTTAGTTCAATA
ACATGAATATTTTGAAGCAATGAAATCTTATAGTTGTTCGATTCAATTGTTTTATTTATAGGTACAATATTATTTTCAAAGTAA
TACTTCAAACCCGGCATATCATTATTATCAATATATCCAACAAGTGTTGACAGGATATTCACATAATCATGACGAAAAACGCGC
ATTTCTCTATGCAGGGACTCTAATGTAGTAACATAATCCTGTAACTGTTCTAGCTGTTCCTTCTGATTCTGTACTTTAAGTTCG
TTGGTTGCCGTATTAATAACAACCGTCACTATAACTATTAACAAAATGGTATATCCTGTAAAAATAAGCGTATTAATTTTCAAA
ACTGATCCATCAAAACCTGCGATTGACCCCGCGTATATGTTCATATAGAACGCTAAGACAGTAAGAGCAACGATAGAAAAAATA
ATATACGCATATTTTCTATGTTCAACAAATCTAGAAATATTAACTTTTTCAATCAATTTTCTAAGAAGAAACGAAAATACCAAC
AAATTCGCCAACATTCCCGCGCAGTAAATTAACGTTGGTAACAGTTCATTAAATATCTCATCATATTTAAAGTTCAAGCCCGGT
ACTAAAATAAAACCTACTATAGAGTCACTAATAGTAAGTAAAATAATAACCACAAGCGTAATAGAAGCTGAAACTACTACATTT
TTATTTTTCCAATATAAGGCACTTACAAAAACAATCAATACAAAAATCATCGACCAGTATTGAACTAAAGTAAATAAAGGGAAG
GCTAGCATAGCAATTGCTATAGTAACCAATCCCTCTTTAATTGAAAAAACTTTGTTTGTTAAAATCTGGATTGCAATAAAAATA
CCCGTTATCTGTATAATTGCCATCAAAATACTAAACATATTAATCCTCCACTGTCTAAAATATCTATGTTTAAATATGTAAGTT
TGCCTTCTTTTTATTTTAGCATGAATTTTGATGTTATACAGTTTTAAATTTATTTATTTTCGTTTTTTTCTTGCATTTTCACAA
ATGGACTTTTTGGTTCGTATACAAACATGAAGCAAGCTTTACTCATAGAAGAATCCGCAACTTTCATGGATTGTTCTTCTAGTT
TTCTAGAAAGGAATTTACCAACTGATTTATTCATATTTTTCATAGTTCCGATACCTCCTTTTCAATAGTTTGTAAGTAAGTGGA
```

```
TTAATACTTATCACCTGAAACAAAGATCCTACCATGATTAATGTTTTCATCTCTGCGAATGGTATTAGCAACGCAATTCCCGTT
AAAATTAGCGTCCCTATCATCGCTTTTCTTTTTAGTGTTTTCCGGTGTTCTTCACCGATTAAAGGCAAACTTTCTGTGTCTGCC
GGAGCGAATAAAAACATGTTGAGCAGTATAAATCCAAATGTACCTAAAACAATCCAATTATTAGAGGGGATATTTTGAAAAACA
AATGGCGCAAGCACAAACATCAATAAGCTAATTAACGTGCAATTCAATGTTTTGGTTGCATGTAATCCAAAAGAATACCGTCTG
AGCCATAAATATGACAGATGCACTGTCACTGTTTGCAGTAAAAGCCCTGTTACTAAGGCGATACCGTATACGAGAGCAAACTTC
ATGACATTAATTAGAATTATTTCCAGTCCATATTTTACTTTTAAATAACCTTCTTCATCATCTTTCCAGCGGTCTTTCGAAATC
AACACATCCGCCATTCTTTCTGACAAAGGGACTTTTGCAGTAAAATTACTCAAAGTAGTTCCTCCTTATCTACAGATGCATTTT
ATTTCATATAGTAAGTACATCACCTATTAGTTTGTTGTTTAAACAAACTAACTTATTTTCATCTTATATAACCTCGTCAGTATT
TTCAATATTTTTTTTAGTTTTTTATGAACACATTAGATATAATAAAGGGAAGATTCGCTATGTACTATGTTGATACTTAATTTA
AAGATTAAACAAATGGAGTGGATGAAGTGGATATCGCTGATCAAACCTTTGTCAAAAAAGTAAATCAAAAGTTATTATTAAAAG
AAATCCTTAAAAATTCACCTATTTCAAGAGCAAAATTATCTGAAATGACTGGATTAAATAAATCAACTGTCTCATCACAGGTAA
ACACGTTAATGAAAGAAAGTATGGTATTTGAAATAGGTCAAGGACAATCAAGTGGCGGAAGAAGACCTGTCATGCTTGTTTTTA
ATAAAAAGGCAGGATACTCCGTTGGAATAGATGTTGGTGTGGATTATATTAATGGCATTTTAACAGACCTTGAAGGAACAATCG
TTCTTGATCAATACCGCCATTTGGAATCCAATTCTCCAGAAATAACGAAAGACATTTTGATTGATATGATTCATCACTTTATTA
CGCAAATGCCCCAATCTCCGTACGGGCTTATTGGTATAGGTATTTGCGTGCCTGGACTCATTGATAAAGATCAAAAAATTGTTT
TCACTCCGAACTCCAACTGGAGAGATATTGACTTAAAATCTTCGATACAAGAGAAGTACAATGTGCCTGTTTTTATTGAAAATG
AGGCAAATGCTGGCGCATATGGAGAAAAAGTATTTGGAGCTGCAAAAAATCACGATAACATTATTTACGTAAGTATCAGCACAG
GAATAGGGATCGGTGTTATTATCAACAATCATTTATATAGAGGAGTAAGCGGCTTCTCTGGAGAAATGGGACATATGACAATAG
ACTTTAATGGTCCTAAATGCAGTTGCGGAAACCGAGGATGCTGGGAATTGTATGCTTCAGAGAAGGCTTTATTAAAATCTCTTC
AGACCAAAGAGAAAAAACTGTCCTATCAAGATATCATAAACCTCGCCCATCTGAATGATATCGGAACCTTAAATGCATTACAAA
ATTTTGGATTCTATTTAGGAATAGGCCTTACCAATATTCTAAATACTTTCAACCCACAAGCCGTAATTTTAAGAAATAGCATAA
TTGAATCGCATCCTATGGTTTTAAATTCAATGAGAAGTGAAGTATCATCAAGGGTTTATTCCCAATTAGGCAATAGCTATGAAT
TATTGCCATCTTCCTTAGGACAGAATGCACCGGCATTAGGAATGTCCTCCATTGTGATTGATCATTTTCTGGACATGATTACAA
TGTAATTTTTTATGGAATGGACAGCTCATCTTTAAAGATGAGTTTTTTTATTCTAGGAGTATTTCTGAAGCAATAGTGACATGG
CACCTTCTCATATGAAAAAGGAGTTCTAAAATAGAAATCTCCTTTTTCATGTGCAAATTATTTTTCTTTATAACGAAAATATCT
AAAGTCGGCCCATTCACTGGCCGTCGTTTTACCGAAATACGGGCAGACATGGCCTGCCCGGTTAATGCCTGGCTCTTCAAACAG
AACAAGACGATTAATAAAATGGAAAAGCCAACTGTGAACATTCTTTTGCAGTTGGTTTTTTTTATGAATAAACTCACTTAAATA
AAATATTAATATGGGTTTTTAAGCCTATTATACAGTCCTTTTTACACGTTCGAATATTCTGAAATGTATCCAAAAAATCGGCAA
TTCTGCTCTTTTTTGGTCGTTCATGACAAGAATCGGACATTTCATTACATTTTTGGTTATTATGGGTAAATTCGTTGTAAAATA
TTAGTGGAGGTGAATTAGGATCACAGAGATGTGGTCAGTCAGTCTTAATTAACCAAGGAGGTGAAATGTACAATGAAAGGAGAA
GAACTTTTCACTGGAGTTGTCCCAATTCTTGTTGAATTAGATGGTGATGTTAATGGGCACAAATTTTCTGTCAGTGGAGAGGGT
GAAGGTGATGCAACATACGGAAAACTTACCCTTAAATTTATTTGCACTACTGGAAAACTACCTGTTCCATGGCCAACACTTGTC
ACTACTTTCGCGTATGGTCTTCAATGCTTTGCGAGATACCCAGATCATATGAAACAGCATGACTTTTTCAAGAGTGCCATGCCC
GAAGGTTATGTACAGGAAAGAACTATATTTTTCAAAGATGACGGGAACTACAAGACACGTGCTGAAGTCAAGTTTGAAGGTGAT
ACCCTTGTTAATAGAATCGAGTTAAAAGGTATTGATTTTAAAGAAGATGGAAACATTCTTGGACACAAATTGGAATACAACTAT
AACTCACACAATGTATACATCATGGCAGACAAACAAAAGAATGGAATCAAAGTTAACTTCAAAATTAGACACAACATTGAAGAT
GGAAGCGTTCAACTAGCAGACCATTATCAACAAAATACTCCAATTGGCGATGGCCCTGTCCTTTTACCAGACAACCATTACCTG
TCCACACAATCTAAGCTTTCGAAAGATCCCAACGAAAAGAGAGACCACATGGTCCTTCTTGAGTTTGTAACAGCTGCTGGGATT
ACACATGGCATGGATGAACTATACAAATAACCTAGGCTGCTGCCACCGCTGAGCAATAACTAGCATAACCCCTTGGGGCCTCTA
AACGGGTCTTGAGGGGTTTTTTGAACGTCGTGACTGGGAAAACCCTGGCGTTACCCAACTTAATCGCCTTGCAGCACATCCCCC
TTTCGCCAGCTGGCGTAATAGCGAAGAGGCCCGCACCGATCGCCCTTCCCAACAGTTGCGCAGCCTGAATGGCGAATGGCGCCT
GATGCGGTATTTTCTCCTTACGCATCTGTGCGGTATTTCACACCGCATATGGTGCACTCTCAGTACAATCTGCTCTGATGCCGC
ATAGTTAAGCCAGCCCCGACACCCGCCAACACCCGCTGACGCGCCCTGACGGGCTTGTCTGCTCCCGGCATCCGCTTACAGACA
AGCTGTGACCGTCTCCGGGAGCTGCATGTGTCAGAGGTTTTCACCGTCATCACCGAAACGCGCGAGACGAAAGGGCCTCGTGAT
ACGCCTATTTTTATAGGTTAATGTCATGATAATAATGGTTTCTTAGACGTCAGGTGGCACTTTTCGGGGAAATGTGCGCGGAAC
CCCTATTTGTTTATTTTTCTAAATACATTCAAATATGTATCCGCTCATGAGACAATAACCCTGATAAATGCTTCAATAATCCAT
CCTCCAAAGTTGGAGAGTGAGTTTTATGTCGCAAATATTAATGTTTCTGGTGAACCTTATCAAATTTTCGTTGATTTAATAGAA
ACATAGCGGTAAAATTAGCAGTAACTTAATAGAACGGAAATGAAAAAAGCCACTCTCATATGCTATTGGCTACCAACCTTTAGC
GAGAATGACTTAATCCTGTACAGCCATACAGGACTTCGACTTATAAGAGGCGCCAACTTCAAATAAGTTATTTGCCTTGTTTTC
GCGAACAAGGCTTATTAGATACACCTATTGTACCGTTACTCTACGAATATTTCAAGTAGTAATTACTAGCATTGTCCGTTACTC
TACGAATATTTCAAGTAGTAATTACTAGCATTGTCCGTTACTCTACGAATATTTCAAGTAGTAATTACTAGCATTGTCATATAC
ATAATAAAACGGATATAAAAGGGCGTTTTCTATACCTAGAAGTCTTGTAAATGTACAGGGCGTTTAGATATAGAGAACGCCCTT
TTTGTGTTCCGTTCCAGTGGAAGCTACCACTTTAAAAAGATGGTCTAGTGTAGCCAATGCAGGAGAGTACACTCGGATATCAGT
TGTCGTTGCATTCAACTGTCTGACGTAAGCGAGGTAAAGGACACAAGCCTTGCATAAAACAAGCCTACGGGATGTAAATCCTAA
TAATGATGATAACCAAGACGTTAGCGGCAAAAAGTGTTGGGGGTTCAAAATAAGACATGATTGTGCGACTGGAGTTAAACAGTT
ACTCGTAAGCGGCGATCATGACACTGATTCACGGCTATTCTTGTACAAGCTAGCTTTATTACAAGGATATGCGGGTTATATAGC
GAATCACCCGAAAGGGAACGGTGTTGGGCGTGAGAAACGCACCGTACGGCGCAATACAATGCCAATAAGCTATATACGGACGGT
ATAGTAGTTTTGTAAGCTATAACCGTTTGTCGTCAATGCAACCAATCTCAATTCGAGACCTCGGCATCTAAGCCAGTACGAATG
AGTGGGCGTTTTAACCTCGTAAATTTTCAACAGGGGTTACTATGCCCAAAACTACATTCAGATTTCCTAACAAACTCGCCAGTA
TGAAAACCTTAAGACCTTAAAGTCAAGGGATTTGAAGGATTTTAACCTCGATTAGCAAAAAATGTAGAGTACTGAAGCAACTAC
CATTAACTAAGATAGTGGGGGATTGAGGAAGAATCCAGAGCTGTTTAAATCAAGTGAAAGACAAGATGAAATTAAAAGAATAGT
GAAAGATAGGGGAGTGGTTCTCTATGAGAAAGGAAATGGCTAGAGAACAAAGGCAGCGGTTTATTGATCTATTGTTAGACTTTA
TGGTAAAGAATCCTCATTTATTTGTTAATGGTACAGAGGATGAAAGTAATAATGTTGTTACAAAATGTAATAGTGATATTAAAG
```

```
AGGTTGCGGAGTCATATTTAACTCTTTTATAGTGAGAGGGTTAAAACTAATTAATATGTATTAAGGCCCAATGTTGGAATTATT
GTATTTCACTAGGCAACCTACTTACTAAAAGTAAGATTATCCATTAGTGGATGTTATAATATTGGGTTTTTTAACACAATAATC
ATCGCCTTTCGGTGTCGTTTGATAGAAAAGTAACCATTAGCGATGAAAAAGTCAATATAAAAAGCCATCCGTAAAAAACGGATG
GCTTACCGTACATAGGATCGTTGGTAGGGCGGCGTATCCTACATCTCTGGTAACTTACCTAGCCAATCAAATGCTTGAGAACGG
CGGTTAGATAAGCGCGTGGGGAACCTTTCCCACCTCAAAGATCCTATATCATTATTATGTTACTTTCTACAGGTAGTATACCAT
GTTCTTATATTTTAGTAAACTCCCCGTTAGCTTAACAGGTCTTTGTAAGCAATTAAACGTCCACTATTCAATCGTCTTTGGATT
TTCGCAGGACCGTTTTTTAGATCGAACATAGTTGATAAGAACAAATAACCGCTTGGGTCCAACTTTATAGCAATTAGTATATGG
TCATTTAAAATCTTTACCAATTCAACGCTATTAGGTTCTTTAGGATTTTGCCCGACATAGTCGGGGTGTTCAACGATATCTTTT
ATGTGCGATGAATATTTTTCATAAATACCAGGATGTTGTTTCTTTACGTGCTTTATAAATCCGGGAAACATTTTTACATCGTTA
GAAGTGCAAGTCAAGTTATATGTATCTATAATGATTTGTGGAAGTTTTGCCACAACAGTTGGTTTATTTACAATCTTTTTTTTA
TTAGCCGTCAAATTTCTCCCTCATCTCGTCTCTTTATATCTTTATTTTATCATAAAGGAGTATTTGAACCGTCGCGCGGGACAG
GTTTATGATAGGGATATTTTATTGAATAATTGATGGTATAAGGGACTTTCATGCTTGGAAAGTGGGGATTATGAATTAGATGCT
TGTCCACAATATGTTCCAATGTAATTAAAATTTATGTTCCCACCTTGACCAAACATCACGTCCATACTTAAATCGTCCCTCCTT
TAATAGGTAAAATATTAATTTACCTTAATAAAAAAATAATGGATAATAGTATTCGTCTGAATTTATATAATCAGGGGGAACTAT
TGATGCTGGGGATACTATTTACAGCGGCGCCATCTACTGATGTCGTAAAGGATTTGCAAGATAAAGTTATATCATTGCAGGATC
ATGAGGTAGCGTTTTTGAACACCACGATATCTAATATGTTGATCCCCGAAGCAAACTTAAGAGTGTGTTGATAGTGCAGTATCT
TAAAATTTTGTGTATAATAGGAATTGAAGTTAAATTAGATGCTAAAAATTTGTAATTAAGAAGGAGGGATTCGTCATGTTGGTA
TTCCAAATGCGTAATGTAGATAAAACATCTACTGTTTTGAAACAGACTAAAAACAGTGATTACGCAGATAAATAAATACGTTAG
ATTAATTCCTACCAGTGACTAATCTTATGACTTTTTAAACAGATAACTAAAATTACAAACAAATCGTTTAACTTCTGTATTTAT
TTACAGATGTAATCACTTCAGGAGTAATTACATGAACAAAAATATAAAATATTCTCAAAACTTTTTAACGAGTGAAAAAGTACT
CAACCAAATAATAAAACAATTGAATTTAAAAGAAACCGATACCGTTTACGAAATTGGAACAGGTAAAGGGCATTTAACGACGAA
ACTGGCTAAAATAAGTAAACAGGTAACGTCTATTGAATTAGACAGTCATCTATTCAACTTATCGTCAGAAAAAATTAAAACTGAA
CATTCGTGTCACTTTAATTCACCAAGATATTCTACAGTTTCAATTCCCTAACAAACAGAGGTATAAAATTGTTGGGAGTATTCC
TTACCATTTAAGCACACAAATTATTAAAAAAGTGGTTTTTGAAAGCCATGCGTCTGACATCTATCTGATTGTTGAAGAAGGATT
CTACAAGCGTACCTTGGATATTCACCGAACACTAGGGTTGCTCTTGCACACTCAAGTCTCGATTCAGCAATTGCTTAAGCTGCC
AGCGGAATGCTTTCATCCTAAACCAAAAGTAAACAGTGTCTTAATAAAACTTACCCGCCATACCACAGATGTTCCAGATAAATA
TTGGAAGCTATATACGTACTTTGTTTCAAAATGGGTCAATCGAGAATATCGTCAACTGTTTACTAAAAATCAGTTTCATCAAGC
AATGAAACACGCCAAAGTAAACAATTTAAGTACCATTACTTATGAGCAAGTATTGTCTATTTTTAATAGTTATCTATTATTTAA
CGGGAGGAAATAATTCTATGAGTCGCTTTTTTAAATTTGGAAAGTTACACGTTACTAAAGGGAATGGAGATAAATTATTAGATA
TACTACTGACAGCTTCCAAGAAGCTAAAGAGGTCCCTAGCGCCTACGGGGAATTTGGGGTACATTGAAAAAGGAAGAGTATGAG
TATTCAACATTTCCGTGTCGCCCTTATTCCCTTTTTTGCGGCATTTTGCCTTCCTGTTTTTGCTCACCCAGAAACGCTGGTGAA
AGTAAAAGATGCTGAAGATCAGTTGGGTGCACGAGTGGGTTACATCGAACTGGATCTCAACAGCGGTAAGATCCTTGAGAGTTT
TCGCCCCGAAGAACGTTTTCCAATGATGAGCACTTTTAAAGTTCTGCTATGTGGCGCGGTATTATCCCGTATTGACGCCGGGCA
AGAGCAACTCGGTCGCCGCATACACTATTCTCAGAATGACTTGGTTGAGTACTCACCAGTCACAGAAAAGCATCTTACGGATGG
CATGACAGTAAGAGAATTATGCAGTGCTGCCATAACCATGAGTGATAACACTGCGGCCAACTTACTTCTGACAACGATCGGAGG
ACCGAAGGAGCTAACCGCTTTTTTGCACAACATGGGGGATCATGTAACTCGCCTTGATCGTTGGGAACCGGAGCTGAATGAAGC
CATACCAAACGACGAGCGTGACACCACGATGCCTGTAGCAATGGCAACAACGTTGCGCAAACTATTAACTGGCGAACTACTTAC
TCTAGCTTCCCGGCAACAATTAATAGACTGGATGGAGGCGGATAAAGTTGCAGGACCACTTCTGCGCTCGGCCCTTCCGGCTGG
CTGGTTTATTGCTGATAAATCTGGAGCCGGTGAGCGTGGGTCTCGCGGTATCATTGCAGCACTGGGGCCAGATGGTAAGCCCTC
CCGTATCGTAGTTATCTACACGACGGGGAGTCAGGCAACTATGGATGAACGAAATAGACAGATCGCTGAGATAGGTGCCTCACT
GATTAAGCATTGGTAACTGTCAGACCAAGTTTACTCATATATACTTTAGATTGATTTAAAACTTCATTTTTAATTTAAAAGGAT
CTAGGTGAAGATCCTTTTTGATAATCTCATGACCAAAATCCCTTAACGTGAGTTTTCGTTCCACTGAGCGTCAGACCCCGTAGA
AAAGATCAAAGGATCTTC-3'
```

pLm-Control-GFP

```
5'-
TTGAGATCCTTTTTTTCTGCGCGTAATCTGCTGCTTGCAAACAAAAAAACCACCGCTACCAGCGGTGGTTTGTTTGCCGGATCA
AGAGCTACCAACTCTTTTTCCGAAGGTAACTGGCTTCAGCAGAGCGCAGATACCAAATACTGTTCTTCTAGTGTAGCCGTAGTT
AGGCCACCACTTCAAGAACTCTGTAGCACCGCCTACATACCTCGCTCTGCTAATCCTGTTACCAGTGGCTGCTGCCAGTGGCGA
TAAGTCGTGTCTTACCGGGTTGGACTCAAGACGATAGTTACCGGATAAGGCGCAGCGGTCGGGCTGAACGGGGGGTTCGTGCAC
ACAGCCCAGCTTGGAGCGAACGACCTACACCGAACTGAGATACCTACAGCGTGAGCTATGAGAAAGCGCCACGCTTCCCGAAGG
GAGAAAGGCGGACAGGTATCCGGTAAGCGGCAGGGTCGGAACAGGAGAGCGCACGAGGGAGCTTCCAGGGGGAAACGCCTGGTA
TCTTTATAGTCCTGTCGGGTTTCGCCACCTCTGACTTGAGCGTCGATTTTTGTGATGCTCGTCAGGGGGGCGGAGCCTATGGAA
AAACGCCAGCAACGCGGCCTTTTTACGGTTCCTGGCCTTTTGCTGGCCTTTTGCTCACATGTTCTTTCCTGCGTTATCCCCTGA
TTCTGTGGATAACCGTATTACCGCCTTTGAGTGAGCTGATACCGCTCGCCGCAGCCGAACGACCGAGCGCAGCGAGTCAGTGAG
CGAGGAAGCGGAAGAGCGCCCAATACGCAAACCGCCTCTCCCCGCGCGTTGGCCGATTCATTAATGCAGCTGGCACGACAGGTT
TCCCGACTGGAAAGCGGGCAGTGAGCGCAACGCAATTAATGTGAGTTAGCTCACTCATTAGGCACCCCAGGCTTTACACTTTAT
GCTTCCGGCTCGTATGTTGTGTGAATTGTGAGCGGATAACAATTTCACACAGGAAACAGCTATGGCCATGATTACGCCAAGCTT
GCATGCCTGCATATCTACAGATGCATTTTATTTCATATAGTAAGTACATCACCTATTAGTTTGTTGTTTAAACAAACTAACTTA
TTTTCATCTTATATAACCTCGTCAGTATTTTCAATATTTTTTTTAGTTTTTTATGAACACATTAGATATAATAAAGGGAAGATT
CGCTATGTACTATGTTGATACTTAATTTAAAGATTAAACAAATGGAGTGGATGAAGTGGATATCGCTGATCAAACCTTTGTCAA
AAAAGTAAATCAAAAGTTATTATTAAAAGAAATCCTTAAAAATTCACCTATTTCAAGAGCAAAATTATCTGAAATGACTGGATT
```

```
AAATAAATCAACTGTCTCATCACAGGTAAACACGTTAATGAAAGAAAGTATGGTATTTGAAATAGGTCAAGGACAATCAAGTGG
CGGAAGAAGACCTGTCATGCTTGTTTTTAATAAAAAGGCAGGATACTCCGTTGGAATAGATGTTGGTGTGGATTATATTAATGG
CATTTTAACAGACCTTGAAGGAACAATCGTTCTTGATCAATACCGCCATTTGGAATCCAATTCTCCAGAAATAACGAAAGACAT
TTTGATTGATATGATTCATCACTTTATTACGCAAATGCCCCAATCTCCGTACGGGCTTATTGGTATAGGTATTTGCGTGCCTGG
ACTCATTGATAAAGATCAAAAAATTGTTTTCACTCCGAACTCCAACTGGAGAGATATTGACTTAAAATCTTCGATACAAGAGAA
GTACAATGTGCCTGTTTTTATTGAAAATGAGGCAAATGCTGGCGCATATGGAGAAAAAGTATTTGGAGCTGCAAAAAATCACGA
TAACATTATTTACGTAAGTATCAGCACAGGAATAGGGATCGGTGTTATTATCAACAATCATTTATATAGAGGAGTAAGCGGCTT
CTCTGGAGAAATGGGACATATGACAATAGACTTTAATGGTCCTAAATGCAGTTGCGGAAACCGAGGATGCTGGGAATTGTATGC
TTCAGAGAAGGCTTTATTAAAATCTCTTCAGACCAAAGAGAAAAAACTGTCCTATCAAGATATCATAAACCTCGCCCATCTGAA
TGATATCGGAACCTTAAATGCATTACAAAATTTTGGATTCTATTTAGGAATAGGCCTTACCAATATTCTAAATACTTTCAACCC
ACAAGCCGTAATTTTAAGAAATAGCATAATTGAATCGCATCCTATGGTTTTAAATTCAATGAGAAGTGAAGTATCATCAAGGGT
TTATTCCCAATTAGGCAATAGCTATGAATTATTGCCATCTTCCTTAGGACAGAATGCACCGGCATTAGGAATGTCCTCCATTGT
GATTGATCATTTTCTGGACATGATTACAATGTAATTTTTTATGGAATGGACAGCTCATCTTTAAAGATGAGTTTTTTTATTCTA
GGAGTATTTCTGAAGCAATAGTGACATGGCACCTTCTCATATGAAAAAGGAGTTCTAAAATAGAAATCTCCTTTTTCATGTGCA
AATTATTTTTCTTTATAACGAAAATATCTAAAGTCGGCCAATTCACTGGCCGTCGTTTTACCGAAATACGGGCAGACATGGCCT
GCCCGGTTAATGCCTGGCTCTTCAAACAGAACAAGACGATTAATAAAATGGAAAAGCCAACTGTGAACATTCTTTTGCAGTTGG
TTTTTTTTATGAATAAACTCACTTAAATAAAATATTAATATGGGTTTTTAAGCCTATTATACAGTCCTTTTTACACGTTCGAAT
ATTCTGAAATGTATCCAAAAAATCGGCAATTCTGCTCTTTTTTGGTCGTTCATGACAAGAATCGGACATTTCATTACATTTTTG
GTTATTATGGGTAAATTCGTTGTAAAATATTAGTGGAGGTGAATTAGGATCACAGAGATGTGGTCAGTCAGTCTTAATTAACCA
AGGAGGTGAAATGTACAATGAAAGGAGAAGAACTTTTCACTGGAGTTGTCCCAATTCTTGTTGAATTAGATGGTGATGTTAATG
GGCACAAATTTTCTGTCAGTGGAGAGGGTGAAGGTGATGCAACATACGGAAAACTTACCCTTAAATTTATTTGCACTACTGGAA
AACTACCTGTTCCATGGCCAACACTTGTCACTACTTTCGCGTATGGTCTTCAATGCTTTGCGAGATACCCAGATCATATGAAAC
AGCATGACTTTTTCAAGAGTGCCATGCCCGAAGGTTATGTACAGGAAAGAACTATATTTTTCAAAGATGACGGGAACTACAAGA
CACGTGCTGAAGTCAAGTTTGAAGGTGATACCCTTGTTAATAGAATCGAGTTAAAAGGTATTGATTTTAAAGAAGATGGAAACA
TTCTTGGACACAAATTGGAATACAACTATAACTCACACAATGTATACATCATGGCAGACAAACAAAAGAATGGAATCAAAGTTA
ACTTCAAAATTAGACACAACATTGAAGATGGAAGCGTTCAACTAGCAGACCATTATCAACAAAATACTCCAATTGGCGATGGCC
CTGTCCTTTTACCAGACAACCATTACCTGTCCACACAATCTAAGCTTTCGAAAGATCCCAACGAAAAGAGAGACCACATGGTCC
TTCTTGAGTTTGTAACAGCTGCTGGGATTACACATGGCATGGATGAACTATACAAATAACCTAGGCTGCTGCCACCGCTGAGCA
ATAACTAGCATAACCCCTTGGGGCCTCTAAACGGGTCTTGAGGGGTTTTTTGAACGTCGTGACTGGGAAAACCCTGGCGTTACC
CAACTTAATCGCCTTGCAGCACATCCCCCTTTCGCCAGCTGGCGTAATAGCGAAGAGGCCCGCACCGATCGCCCTTCCCAACAG
TTGCGCAGCCTGAATGGCGAATGGCGCCTGATGCGGTATTTTCTCCTTACGCATCTGTGCGGTATTTCACACCGCATATGGTGC
ACTCTCAGTACAATCTGCTCTGATGCCGCATAGTTAAGCCAGCCCCGACACCCGCCAACACCCGCTGACGCGCCCTGACGGGCT
TGTCTGCTCCCGGCATCCGCTTACAGACAAGCTGTGACCGTCTCCGGGAGCTGCATGTGTCAGAGGTTTTCACCGTCATCACCG
AAACGCGCGAGACGAAAGGGCCTCGTGATACGCCTATTTTTATAGGTTAATGTCATGATAATAATGGTTTCTTAGACGTCAGGT
GGCACTTTTCGGGGAAATGTGCGCGGAACCCCTATTTGTTTATTTTTCTAAATACATTCAAATATGTATCCGCTCATGAGACAA
TAACCCTGATAAATGCTTCAATAATCCATCCTCCAAAGTTGGAGAGTGAGTTTTATGTCGCAAATATTAATGTTTCTGGTGAAC
CTTATCAAATTTTCGTTGATTTAATAGAAACATAGCGGTAAAATTAGCAGTAACTTAATAGAACGGAAATGAAAAAAGCCACTC
TCATATGCTATTGGCTACCAACCTTTAGCGAGAATGACTTAATCCTGTACAGCCATACAGGACTTCGACTTATAAGAGGCGCCA
ACTTCAAATAAGTTATTTGCCTTGTTTTCGCGAACAAGGCTTATTAGATACACCTATTGTACCGTTACTCTACGAATATTTCAA
GTAGTAATTACTAGCATTGTCCGTTACTCTACGAATATTTCAAGTAGTAATTACTAGCATTGTCCGTTACTCTACGAATATTTC
AAGTAGTAATTACTAGCATTGTCATATACATAATAAAACGGATATAAAAGGGCGTTTTCTATACCTAGAAGTCTTGTAAATGTA
CAGGGCGTTTAGATATAGAGAACGCCCTTTTTGTGTTCCGTTCCAGTGGAAGCTACCACTTTAAAAAGATGGTCTAGTGTAGCC
AATGCAGGAGAGTACACTCGGATATCAGTTGTCGTTGCATTCAACTGTCTGACGTAAGCGAGGTAAAGGACACAAGCCTTGCAT
AAAACAAGCCTACGGGATGTAAATCCTAATAATGATGATAACCAAGACGTTAGCGGCAAAAAGTGTTGGGGGTTCAAAATAAGA
CATGATTGTGCGACTGGAGTTAAACAGTTACTCGTAAGCGGCGATCATGACACTGATTCACGGCTATTCTTGTACAAGCTAGCT
TTATTACAAGGATATGCGGGTTATATAGCGAATCACCCGAAAGGGAACGGTGTTGGGCGTGAGAAACGCACCGTACGGCGCAAT
ACAATGCCAATAAGCTATATACGGACGGTATAGTAGTTTTGTAAGCTATAACCGTTTGTCGTCAATGCAACCAATCTCAATTCG
AGACCTCGGCATCTAAGCCAGTACGAATGAGTGGGCGTTTTAACCTCGTAAATTTTCAACAGGGGTTACTATGCCCAAAACTAC
ATTCAGATTTCCTAACAAACTCGCCAGTATGAAAACCTTAAGACCTTAAAGTCAAGGGATTTGAAGGATTTTAACCTCGATTAG
CAAAAAATGTAGAGTACTGAAGCAACTACCATTAACTAAGATAGTGGGGGATTGAGGAAGAATCCAGAGCTGTTTAAATCAAGT
GAAAGACAAGATGAAATTAAAAGAATAGTGAAAGATAGGGGAGTGGTTCTCTATGAGAAAGGAAATGGCTAGAGAACAAAGGCA
GCGGTTTATTGATCTATTGTTAGACTTTATGGTAAAGAATCCTCATTTATTTGTTAATGGTACAGAGGATGAAAGTAATAATGT
TGTTACAAAATGTAATAGTGATATTAAAGAGGTTGCGGAGTCATATTTAACTCTTTTATAGTGAGAGGGTTAAAACTAATTAAT
ATGTATTAAGGCCCAATGTTGGAATTATTGTATTTCACTAGGCAACCTACTTACTAAAAGTAAGATTATCCATTAGTGGATGTT
ATAATATTGGGTTTTTTAACACAATAATCATCGCCTTTCGGTGTCGTTTGATAGAAAAGTAACCATTAGCGATGAAAAAGTCAA
TATAAAAAGCCATCCGTAAAAAACGGATGGCTTACCGTACATAGGATCGTTGGTAGGGCGGCGTATCCTACATCTCTGGTAACT
TACCTAGCCAATCAAATGCTTGAGAACGGCGGTTAGATAAGCGCGTGGGGAACCTTTCCCACCTCAAAGATCCTATATCATTAT
TATGTTACTTTCTACAGGTAGTATACCATGTTCTTATATTTTAGTAAACTCCCCGTTAGCTTAACAGGTCTTTGTAAGCAATTA
AACGTCCACTATTCAATCGTCTTTGGATTTTCGCAGGACCGTTTTTTAGATCGAACATAGTTGATAAGAACAAATAACCGCTTG
GGTCCAACTTTATAGCAATTAGTATATGGTCATTTAAAATCTTTACCAATTCAACGCTATTAGGTTCTTTAGGATTTTGCCCGA
CATAGTCGGGGTGTTCAACGATATCTTTTATGTGCGATGAATATTTTTCATAAATACCAGGATGTTGTTTCTTTACGTGCTTTA
TAAATCCGGGAAACATTTTTACATCGTTAGAAGTGCAAGTCAAGTTATATGTATCTATAATGATTTGTGGAAGTTTTGCCACAA
CAGTTGGTTTATTTACAATCTTTTTTTTATTAGCCGTCAAATTTCTCCCTCATCTCGTCTCTTTATATCTTTATTTTATCATAA
```

```
AGGAGTATTTGAACCGTCGCGCGGGACAGGTTTATGATAGGGATATTTTATTGAATAATTGATGGTATAAGGGACTTTCATGCT
TGGAAAGTGGGGATTATGAATTAGATGCTTGTCCACAATATGTTCCAATGTAATTAAAATTTATGTTCCCACCTTGACCAAACA
TCACGTCCATACTTAAATCGTCCCTCCTTTAATAGGTAAAATATTAATTTACCTTAATAAAAAAATAATGGATAATAGTATTCG
TCTGAATTTATATAATCAGGGGGAACTATTGATGCTGGGGATACTATTTACAGCGGCGCCATCTACTGATGTCGTAAAGGATTT
GCAAGATAAAGTTATATCATTGCAGGATCATGAGGTAGCGTTTTTGAACACCACGATATCTAATATGTTGATCCCCGAAGCAAA
CTTAAGAGTGTGTTGATAGTGCAGTATCTTAAAATTTTGTGTATAATAGGAATTGAAGTTAAATTAGATGCTAAAAATTTGTAA
TTAAGAAGGAGGGATTCGTCATGTTGGTATTCCAAATGCGTAATGTAGATAAAACATCTACTGTTTTGAAACAGACTAAAAACA
GTGATTACGCAGATAAATAAATACGTTAGATTAATTCCTACCAGTGACTAATCTTATGACTTTTTAAACAGATAACTAAAATTA
CAAACAAATCGTTTAACTTCTGTATTTATTTACAGATGTAATCACTTCAGGAGTAATTACATGAACAAAAATATAAAATATTCT
CAAAACTTTTTAACGAGTGAAAAAGTACTCAACCAAATAATAAAACAATTGAATTTAAAAGAAACCGATACCGTTTACGAAATT
GGAACAGGTAAAGGGCATTTAACGACGAAACTGGCTAAAATAAGTAAACAGGTAACGTCTATTGAATTAGACAGTCATCTATTC
AACTTATCGTCAGAAAAATTAAAACTGAACATTCGTGTCACTTTAATTCACCAAGATATTCTACAGTTTCAATTCCCTAACAAA
CAGAGGTATAAAATTGTTGGGAGTATTCCTTACCATTTAAGCACACAAATTATTAAAAAAGTGGTTTTTGAAAGCCATGCGTCT
GACATCTATCTGATTGTTGAAGAAGGATTCTACAAGCGTACCTTGGATATTCACCGAACACTAGGGTTGCTCTTGCACACTCAA
GTCTCGATTCAGCAATTGCTTAAGCTGCCAGCGGAATGCTTTCATCCTAAACCAAAAGTAAACAGTGTCTTAATAAAACTTACC
CGCCATACCACAGATGTTCCAGATAAATATTGGAAGCTATATACGTACTTTGTTTCAAAATGGGTCAATCGAGAATATCGTCAA
CTGTTTACTAAAAATCAGTTTCATCAAGCAATGAAACACGCCAAAGTAAACAATTTAAGTACCATTACTTATGAGCAAGTATTG
TCTATTTTTAATAGTTATCTATTATTTAACGGGAGGAAATAATTCTATGAGTCGCTTTTTTAAATTTGGAAAGTTACACGTTAC
TAAAGGGAATGGAGATAAATTATTAGATATACTACTGACAGCTTCCAAGAAGCTAAAGAGGTCCCTAGCGCCTACGGGGAATTT
GGGGTACATTGAAAAAGGAAGAGTATGAGTATTCAACATTTCCGTGTCGCCCTTATTCCCTTTTTTGCGGCATTTTGCCTTCCT
GTTTTTGCTCACCCAGAAACGCTGGTGAAAGTAAAAGATGCTGAAGATCAGTTGGGTGCACGAGTGGGTTACATCGAACTGGAT
CTCAACAGCGGTAAGATCCTTGAGAGTTTTCGCCCCGAAGAACGTTTTCCAATGATGAGCACTTTTAAAGTTCTGCTATGTGGC
GCGGTATTATCCCGTATTGACGCCGGGCAAGAGCAACTCGGTCGCCGCATACACTATTCTCAGAATGACTTGGTTGAGTACTCA
CCAGTCACAGAAAAGCATCTTACGGATGGCATGACAGTAAGAGAATTATGCAGTGCTGCCATAACCATGAGTGATAACACTGCG
GCCAACTTACTTCTGACAACGATCGGAGGACCGAAGGAGCTAACCGCTTTTTTGCACAACATGGGGGATCATGTAACTCGCCTT
GATCGTTGGGAACCGGAGCTGAATGAAGCCATACCAAACGACGAGCGTGACACCACGATGCCTGTAGCAATGGCAACAACGTTG
CGCAAACTATTAACTGGCGAACTACTTACTCTAGCTTCCCGGCAACAATTAATAGACTGGATGGAGGCGGATAAAGTTGCAGGA
CCACTTCTGCGCTCGGCCCTTCCGGCTGGCTGGTTTATTGCTGATAAATCTGGAGCCGGTGAGCGTGGGTCTCGCGGTATCATT
GCAGCACTGGGGCCAGATGGTAAGCCCTCCCGTATCGTAGTTATCTACACGACGGGGAGTCAGGCAACTATGGATGAACGAAAT
AGACAGATCGCTGAGATAGGTGCCTCACTGATTAAGCATTGGTAACTGTCAGACCAAGTTTACTCATATATACTTTAGATTGAT
TTAAAACTTCATTTTTAATTTAAAAGGATCTAGGTGAAGATCCTTTTTGATAATCTCATGACCAAAATCCCTTAACGTGAGTTT
TCGTTCCACTGAGCGTCAGACCCCGTAGAAAAGATCAAAGGATCTTC--3’
```

pXyl-Lm-agrC-GFP

```
5’-
TTGAGATCCTTTTTTTCTGCGCGTAATCTGCTGCTTGCAAACAAAAAAACCACCGCTACCAGCGGTGGTTTGTTTGCCGGATCA
AGAGCTACCAACTCTTTTTCCGAAGGTAACTGGCTTCAGCAGAGCGCAGATACCAAATACTGTTCTTCTAGTGTAGCCGTAGTT
AGGCCACCACTTCAAGAACTCTGTAGCACCGCCTACATACCTCGCTCTGCTAATCCTGTTACCAGTGGCTGCTGCCAGTGGCGA
TAAGTCGTGTCTTACCGGGTTGGACTCAAGACGATAGTTACCGGATAAGGCGCAGCGGTCGGGCTGAACGGGGGGTTCGTGCAC
ACAGCCCAGCTTGGAGCGAACGACCTACACCGAACTGAGATACCTACAGCGTGAGCTATGAGAAAGCGCCACGCTTCCCGAAGG
GAGAAAGGCGGACAGGTATCCGGTAAGCGGCAGGGTCGGAACAGGAGAGCGCACGAGGGAGCTTCCAGGGGGAAACGCCTGGTA
TCTTTATAGTCCTGTCGGGTTTCGCCACCTCTGACTTGAGCGTCGATTTTTGTGATGCTCGTCAGGGGGGCGGAGCCTATGGAA
AAACGCCAGCAACGCGGCCTTTTTACGGTTCCTGGCCTTTTGCTGGCCTTTTGCTCACATGTTCTTTCCTGCGTTATCCCCTGA
TTCTGTGGATAACCGTATTACCGCCTTTGAGTGAGCTGATACCGCTCGCCGCAGCCGAACGACCGAGCGCAGCGAGTCAGTGAG
CGAGGAAGCGGAAGAGCGCCCAATACGCAAACCGCCTCTCCCCGCGCGTTGGCCGATTCATTAATGCAGCTGGCACGACAGGTT
TCCCGACTGGAAAGCGGGCAGTGAGCGCAACGCAATTAATGTGAGTTAGCTCACTCATTAGGCACCCCAGGCTTTACACTTTAT
GCTTCCGGCTCGTATGTTGTGTGAATTGTGAGCGGATAACAATTTCACACAGGAAACAGCTATGGCCATGATTACGCCAAGCTT
GCATGCCTGCACTACATAATTTCTAATTCTTGAATAACTTCTCTATCGGTCACTTTCGTATCTAAGGCAACGTGCGAATATTTC
TTCATAATTTCCCGCAAACTAGCAAGGCCTAATCCACGACCTTCTCCTTTTGTAGAGAAACCTTCTTCAAATATCTTATAAATC
GGTGGCATATTTACTGGTAAACTATTCGCAAATACAATAATTATGCTATCACCTTTTTTCACAAACGCGATTCGAATAACTGGA
TTTTCACAAGTTAACGCCGCTTCAACCGCATTATCTAGCAAAATACCGACAACTTTGCATAAATCTATACTATCCATCGAAATC
TTGTCAATCGGTTCAACTACTTCCAAAATTGCATCAATTTTCAACTCTTGAGCTCGTATTAATTTAACAGCTAGCAAGCCCTTT
AGTTCAATAACATGAATATTTTGAAGCAATGAAATCTTATAGTTGTTCGATTCAATTGTTTTATTTATAGGTACAATATTATTT
TCAAAGTAATACTTCAAACCCGGCATATCATTATTATCAATATATCCAACAAGTGTTGACAGGATATTCACATAATCATGACGA
AAAACGCGCATTTCTCTATGCAGGGACTCTAATGTAGTAACATAATCCTGTAACTGTTCTAGCTGTTCCTTCTGATTCTGTACT
TTAAGTTCGTTGGTTGCCGTATTAATAACAACCGTCACTATAACTATTAACAAAATGGTATATCCTGTAAAAATAAGCGTATTA
ATTTTCAAAACTGATCCATCAAAACCTGCGATTGACCCCGCGTATATGTTCATATAGAACGCTAAGACAGTAAGAGCAACGATA
GAAAAAATAATATACGCATATTTTCTATGTTCAACAAATCTAGAAATATTAACTTTTTCAATCAATTTTCTAAGAAGAAACGAA
AATACCAACAAATTCGCCAACATTCCCGCGCAGTAAATTAACGTTGGTAACAGTTCATTAAATATCTCATCATATTTAAAGTTC
AAGCCCGGTACTAAAATAAAACCTACTATAGAGTCACTAATAGTAAGTAAAATAATAACCACAAGCGTAATAGAAGCTGAAACT
ACTACATTTTTATTTTTCCAATATAAGGCACTTACAAAAACAATCAATACAAAAATCATCGACCAGTATTGAACTAAAGTAAAT
AAAGGGAAGGCTAGCATAGCAATTGCTATAGTAACCAATCCCTCTTTAATTGAAAAAACTTTGTTTGTTAAAATCTGGATTGCA
```

```
ATAAAAATACCCGTTATCTGTATAATTGCCATCAAAATACTAAACATAGTAGTTCCTCCTTATCTACAGATGCATTTTATTTCA
TATAGTAAGTACATCACCTATTAGTTTGTTGTTTAAACAAACTAACTTATTTTCATCTTATATAACCTCGTCAGTATTTTCAAT
ATTTTTTTTAGTTTTTTATGAACACATTAGATATAATAAAGGGAAGATTCGCTATGTACTATGTTGATACTTAATTTAAAGATT
AAACAAATGGAGTGGATGAAGTGGATATCGCTGATCAAACCTTTGTCAAAAAAGTAAATCAAAAGTTATTATTAAAAGAAATCC
TTAAAAATTCACCTATTTCAAGAGCAAAATTATCTGAAATGACTGGATTAAATAAATCAACTGTCTCATCACAGGTAAACACGT
TAATGAAAGAAAGTATGGTATTTGAAATAGGTCAAGGACAATCAAGTGGCGGAAGAAGACCTGTCATGCTTGTTTTTAATAAAA
AGGCAGGATACTCCGTTGGAATAGATGTTGGTGTGGATTATATTAATGGCATTTTAACAGACCTTGAAGGAACAATCGTTCTTG
ATCAATACCGCCATTTGGAATCCAATTCTCCAGAAATAACGAAAGACATTTTGATTGATATGATTCATCACTTTATTACGCAAA
TGCCCCAATCTCCGTACGGGCTTATTGGTATAGGTATTTGCGTGCCTGGACTCATTGATAAAGATCAAAAAATTGTTTTCACTC
CGAACTCCAACTGGAGAGATATTGACTTAAAATCTTCGATACAAGAGAAGTACAATGTGCCTGTTTTTATTGAAAATGAGGCAA
ATGCTGGCGCATATGGAGAAAAAGTATTTGGAGCTGCAAAAAATCACGATAACATTATTTACGTAAGTATCAGCACAGGAATAG
GGATCGGTGTTATTATCAACAATCATTTATATAGAGGAGTAAGCGGCTTCTCTGGAGAAATGGGACATATGACAATAGACTTTA
ATGGTCCTAAATGCAGTTGCGGAAACCGAGGATGCTGGGAATTGTATGCTTCAGAGAAGGCTTTATTAAAATCTCTTCAGACCA
AAGAGAAAAAACTGTCCTATCAAGATATCATAAACCTCGCCCATCTGAATGATATCGGAACCTTAAATGCATTACAAAATTTTG
GATTCTATTTAGGAATAGGCCTTACCAATATTCTAAATACTTTCAACCCACAAGCCGTAATTTTAAGAAATAGCATAATTGAAT
CGCATCCTATGGTTTTAAATTCAATGAGAAGTGAAGTATCATCAAGGGTTTATTCCCAATTAGGCAATAGCTATGAATTATTGC
CATCTTCCTTAGGACAGAATGCACCGGCATTAGGAATGTCCTCCATTGTGATTGATCATTTTCTGGACATGATTACAATGTAAT
TTTTTATGGAATGGACAGCTCATCTTTAAAGATGAGTTTTTTTATTCTAGGAGTATTTCTGAAGCAATAGTGACATGGCACCTT
CTCATATGAAAAAGGAGTTCTAAAATAGAAATCTCCTTTTTCATGTGCAAATTATTTTTCTTTATAACGAAAATATCTAAAGTC
GGCCCATTCACTGGCCGTCGTTTTACCGAAATACGGGCAGACATGGCCTGCCCGGTTAATGCCTGGCTCTTCAAACAGAACAAG
ACGATTAATAAAATGGAAAAGCCAACTGTGAACATTCTTTTGCAGTTGGTTTTTTTTATGAATAAACTCACTTAAATAAAATAT
TAATATGGGTTTTTAAGCCTATTATACAGTCCTTTTTACACGTTCGAATATTCTGAAATGTATCCAAAAAATCGGCAATTCTGC
TCTTTTTTGGTCGTTCATGACAAGAATCGGACATTTCATTACATTTTTGGTTATTATGGGTAAATTCGTTGTAAAATATTAGTG
GAGGTGAATTAGGATCACAGAGATGTGGTCAGTCAGTCTTAATTAACCAAGGAGGTGAAATGTACAATGAAAGGAGAAGAACTT
TTCACTGGAGTTGTCCCAATTCTTGTTGAATTAGATGGTGATGTTAATGGGCACAAATTTTCTGTCAGTGGAGAGGGTGAAGGT
GATGCAACATACGGAAAACTTACCCTTAAATTTATTTGCACTACTGGAAAACTACCTGTTCCATGGCCAACACTTGTCACTACT
TTCGCGTATGGTCTTCAATGCTTTGCGAGATACCCAGATCATATGAAACAGCATGACTTTTTCAAGAGTGCCATGCCCGAAGGT
TATGTACAGGAAAGAACTATATTTTTCAAAGATGACGGGAACTACAAGACACGTGCTGAAGTCAAGTTTGAAGGTGATACCCTT
GTTAATAGAATCGAGTTAAAAGGTATTGATTTTAAAGAAGATGGAAACATTCTTGGACACAAATTGGAATACAACTATAACTCA
CACAATGTATACATCATGGCAGACAAACAAAAGAATGGAATCAAAGTTAACTTCAAAATTAGACACAACATTGAAGATGGAAGC
GTTCAACTAGCAGACCATTATCAACAAAATACTCCAATTGGCGATGGCCCTGTCCTTTTACCAGACAACCATTACCTGTCCACA
CAATCTAAGCTTTCGAAAGATCCCAACGAAAAGAGAGACCACATGGTCCTTCTTGAGTTTGTAACAGCTGCTGGGATTACACAT
GGCATGGATGAACTATACAAATAACCTAGGCTGCTGCCACCGCTGAGCAATAACTAGCATAACCCCTTGGGGCCTCTAAACGGG
TCTTGAGGGGTTTTTTGAACGTCGTGACTGGGAAAACCCTGGCGTTACCCAACTTAATCGCCTTGCAGCACATCCCCCTTTCGC
CAGCTGGCGTAATAGCGAAGAGGCCCGCACCGATCGCCCTTCCCAACAGTTGCGCAGCCTGAATGGCGAATGGCGCCTGATGCG
ATATTTTCTCCTTACGCATCTGTGCGGTATTTCACACCGCATATGGTGCACTCTCAGTACAATCTGCTCTGATGCCGCATAGTT
AAGCCAGCCCCGACACCCGCCAACACCCGCTGACGCGCCCTGACGGGCTTGTCTGCTCCCGGCATCCGCTTACAGACAAGCTGT
GACCGTCTCCGGGAGCTGCATGTGTCAGAGGTTTTCACCGTCATCACCGAAACGCGCGAGACGAAAGGGCCTCGTGATACGCCT
ATTTTTATAGGTTAATGTCATGATAATAATGGTTTCTTAGACGTCAGGTGGCACTTTTCGGGGAAATGTGCGCGGAACCCCTAT
TTGTTTATTTTTCTAAATACATTCAAATATGTATCCGCTCATGAGACAATAACCCTGATAAATGCTTCAATAATCCATCCTCCA
AAGTTGGAGAGTGAGTTTTATGTCGCAAATATTAATGTTTCTGGTGAACCTTATCAAATTTTCGTTGATTTAATAGAAACATAG
CGGTAAAATTAGCAGTAACTTAATAGAACGGAAATGAAAAAAGCCACTCTCATATGCTATTGGCTACCAACCTTTAGCGAGAAT
GACTTAATCCTGTACAGCCATACAGGACTTCGACTTATAAGAGGCGCCAACTTCAAATAAGTTATTTGCCTTGTTTTCGCGAAC
AAGGCTTATTAGATACACCTATTGTACCGTTACTCTACGAATATTTCAAGTAGTAATTACTAGCATTGTCCGTTACTCTACGAA
TATTTCAAGTAGTAATTACTAGCATTGTCCGTTACTCTACGAATATTTCAAGTAGTAATTACTAGCATTGTCATATACATAATA
AAACGGATATAAAAGGGCGTTTTCTATACCTAGAAGTCTTGTAAATGTACAGGGCGTTTAGATATAGAGAACGCCCTTTTTGTG
TTCCGTTCCAGTGGAAGCTACCACTTTAAAAAGATGGTCTAGTGTAGCCAATGCAGGAGAGTACACTCGGATATCAGTTGTCGT
TGCATTCAACTGTCTGACGTAAGCGAGGTAAAGGACACAAGCCTTGCATAAAACAAGCCTACGGGATGTAAATCCTAATAATGA
TGATAACCAAGACGTTAGCGGCAAAAAGTGTTGGGGGTTCAAAATAAGACATGATTGTGCGACTGGAGTTAAACAGTTACTCGT
AAGCGGCGATCATGACACTGATTCACGGCTATTCTTGTACAAGCTAGCTTTATTACAAGGATATGCGGGTTATATAGCGAATCA
CCCGAAAGGGAACGGTGTTGGGCGTGAGAAACGCACCGTACGGCGCAATACAATGCCAATAAGCTATATACGGACGGTATAGTA
GTTTTGTAAGCTATAACCGTTTGTCGTCAATGCAACCAATCTCAATTCGAGACCTCGGCATCTAAGCCAGTACGAATGAGTGGG
CGTTTTAACCTCGTAAATTTTCAACAGGGGTTACTATGCCCAAAACTACATTCAGATTTCCTAACAAACTCGCCAGTATGAAAA
CCTTAAGACCTTAAAGTCAAGGGATTTGAAGGATTTTAACCTCGATTAGCAAAAAATGTAGAGTACTGAAGCAACTACCATTAA
CTAAGATAGTGGGGGATTGAGGAAGAATCCAGAGCTGTTTAAATCAAGTGAAAGACAAGATGAAATTAAAAGAATAGTGAAAGA
TAGGGGAGTGGTTCTCTATGAGAAAGGAAATGGCTAGAGAACAAAGGCAGCGGTTTATTGATCTATTGTTAGACTTTATGGTAA
AGAATCCTCATTTATTTGTTAATGGTACAGAGGATGAAAGTAATAATGTTGTTACAAAATGTAATAGTGATATTAAAGAGGTTG
CGGAGTCATATTTAACTCTTTTATAGTGAGAGGGTTAAAACTAATTAATATGTATTAAGGCCCAATGTTGGAATTATTGTATTT
CACTAGGCAACCTACTTACTAAAAGTAAGATTATCCATTAGTGGATGTTATAATATTGGGTTTTTTAACACAATAATCATCGCC
TTTCGGTGTCGTTTGATAGAAAAGTAACCATTAGCGATGAAAAAGTCAATATAAAAAGCCATCCGTAAAAAACGGATGGCTTAC
CGTACATAGGATCGTTGGTAGGGCGGCGTATCCTACATCTCTGGTAACTTACCTAGCCAATCAAATGCTTGAGAACGGCGGTTA
GATAAGCGCGTGGGGAACCTTTCCCACCTCAAAGATCCTATATCATTATTATGTTACTTTCTACAGGTAGTATACCATGTTCTT
ATATTTTAGTAAACTCCCCGTTAGCTTAACAGGTCTTTGTAAGCAATTAAACGTCCACTATTCAATCGTCTTTGGATTTTCGCA
```

```
GGACCGTTTTTTAGATCGAACATAGTTGATAAGAACAAATAACCGCTTGGGTCCAACTTTATAGCAATTAGTATATGGTCATTT
AAAATCTTTACCAATTCAACGCTATTAGGTTCTTTAGGATTTTGCCCGACATAGTCGGGGTGTTCAACGATATCTTTTATGTGC
GATGAATATTTTTCATAAATACCAGGATGTTGTTTCTTTACGTGCTTTATAAATCCGGGAAACATTTTTACATCGTTAGAAGTG
CAAGTCAAGTTATATGTATCTATAATGATTTGTGGAAGTTTTGCCACAACAGTTGGTTTATTTACAATCTTTTTTTTATTAGCC
GTCAAATTTCTCCCTCATCTCGTCTCTTTATATCTTTATTTTATCATAAAGGAGTATTTGAACCGTCGCGCGGGACAGGTTTAT
GATAGGGATATTTTATTGAATAATTGATGGTATAAGGGACTTTCATGCTTGGAAAGTGGGGATTATGAATTAGATGCTTGTCCA
CAATATGTTCCAATGTAATTAAAATTTATGTTCCCACCTTGACCAAACATCACGTCCATACTTAAATCGTCCCTCCTTTAATAG
GTAAAATATTAATTTACCTTAATAAAAAAATAATGGATAATAGTATTCGTCTGAATTTATATAATCAGGGGGAACTATTGATGC
TGGGGATACTATTTACAGCGGCGCCATCTACTGATGTCGTAAAGGATTTGCAAGATAAAGTTATATCATTGCAGGATCATGAGG
TAGCGTTTTTGAACACCACGATATCTAATATGTTGATCCCCGAAGCAAACTTAAGAGTGTGTTGATAGTGCAGTATCTTAAAAT
TTTGTGTATAATAGGAATTGAAGTTAAATTAGATGCTAAAAATTTGTAATTAAGAAGGAGGGATTCGTCATGTTGGTATTCCAA
ATGCGTAATGTAGATAAAACATCTACTGTTTTGAAACAGACTAAAAACAGTGATTACGCAGATAAATAAATACGTTAGATTAAT
TCCTACCAGTGACTAATCTTATGACTTTTTAAACAGATAACTAAAATTACAAACAAATCGTTTAACTTCTGTATTTATTTACAG
ATGTAATCACTTCAGGAGTAATTACATGAACAAAAATATAAAATATTCTCAAAACTTTTTAACGAGTGAAAAAGTACTCAACCA
AATAATAAAACAATTGAATTTAAAAGAAACCGATACCGTTTACGAAATTGGAACAGGTAAAGGGCATTTAACGACGAAACTGGC
TAAAATAAGTAAACAGGTAACGTCTATTGAATTAGACAGTCATCTATTCAACTTATCGTCAGAAAAATTAAAACTGAACATTCG
TGTCACTTTAATTCACCAAGATATTCTACAGTTTCAATTCCCTAACAAACAGAGGTATAAAATTGTTGGGAGTATTCCTTACCA
TTTAAGCACACAAATTATTAAAAAAGTGGTTTTTGAAAGCCATGCGTCTGACATCTATCTGATTGTTGAAGAAGGATTCTACAA
GCGTACCTTGGATATTCACCGAACACTAGGGTTGCTCTTGCACACTCAAGTCTCGATTCAGCAATTGCTTAAGCTGCCAGCGGA
ATGCTTTCATCCTAAACCAAAAGTAAACAGTGTCTTAATAAAACTTACCCGCCATACCACAGATGTTCCAGATAAATATTGGAA
GCTATATACGTACTTTGTTTCAAAATGGGTCAATCGAGAATATCGTCAACTGTTTACTAAAAATCAGTTTCATCAAGCAATGAA
ACACGCCAAAGTAAACAATTTAAGTACCATTACTTATGAGCAAGTATTGTCTATTTTTAATAGTTATCTATTATTTAACGGGAG
GAAATAATTCTATGAGTCGCTTTTTTAAATTTGGAAAGTTACACGTTACTAAAGGGAATGGAGATAAATTATTAGATATACTAC
TGACAGCTTCCAAGAAGCTAAAGAGGTCCCTAGCGCCTACGGGGAATTTGGGGTACATTGAAAAAGGAAGAGTATGAGTATTCA
ACATTTCCGTGTCGCCCTTATTCCCTTTTTTGCGGCATTTTGCCTTCCTGTTTTTGCTCACCCAGAAACGCTGGTGAAAGTAAA
AGATGCTGAAGATCAGTTGGGTGCACGAGTGGGTTACATCGAACTGGATCTCAACAGCGGTAAGATCCTTGAGAGTTTTCGCCC
CGAAGAACGTTTTCCAATGATGAGCACTTTTAAAGTTCTGCTATGTGGCGCGGTATTATCCCGTATTGACGCCGGGCAAGAGCA
ACTCGGTCGCCGCATACACTATTCTCAGAATGACTTGGTTGAGTACTCACCAGTCACAGAAAAGCATCTTACGGATGGCATGAC
AGTAAGAGAATTATGCAGTGCTGCCATAACCATGAGTGATAACACTGCGGCCAACTTACTTCTGACAACGATCGGAGGACCGAA
GGAGCTAACCGCTTTTTTGCACAACATGGGGGATCATGTAACTCGCCTTGATCGTTGGGAACCGGAGCTGAATGAAGCCATACC
AAACGACGAGCGTGACACCACGATGCCTGTAGCAATGGCAACAACGTTGCGCAAACTATTAACTGGCGAACTACTTACTCTAGC
TTCCCGGCAACAATTAATAGACTGGATGGAGGCGGATAAAGTTGCAGGACCACTTCTGCGCTCGGCCCTTCCGGCTGGCTGGTT
TATTGCTGATAAATCTGGAGCCGGTGAGCGTGGGTCTCGCGGTATCATTGCAGCACTGGGGCCAGATGGTAAGCCCTCCCGTAT
CGTAGTTATCTACACGACGGGGAGTCAGGCAACTATGGATGAACGAAATAGACAGATCGCTGAGATAGGTGCCTCACTGATTAA
GCATTGGTAACTGTCAGACCAAGTTTACTCATATATACTTTAGATTGATTTAAAACTTCATTTTTAATTTAAAAGGATCTAGGT
GAAGATCCTTTTTGATAATCTCATGACCAAAATCCCTTAACGTGAGTTTTCGTTCCACTGAGCGTCAGACCCCGTAGAAAAGAT
CAAAGGATCTTC-3'
```

pXyl-Lm-agrA-GFP

```
5'-
TTGAGATCCTTTTTTTCTGCGCGTAATCTGCTGCTTGCAAACAAAAAAACCACCGCTACCAGCGGTGGTTTGTTTGCCGGATCA
AGAGCTACCAACTCTTTTTCCGAAGGTAACTGGCTTCAGCAGAGCGCAGATACCAAATACTGTTCTTCTAGTGTAGCCGTAGTT
AGGCCACCACTTCAAGAACTCTGTAGCACCGCCTACATACCTCGCTCTGCTAATCCTGTTACCAGTGGCTGCTGCCAGTGGCGA
TAAGTCGTGTCTTACCGGGTTGGACTCAAGACGATAGTTACCGGATAAGGCGCAGCGGTCGGGCTGAACGGGGGGTTCGTGCAC
ACAGCCCAGCTTGGAGCGAACGACCTACACCGAACTGAGATACCTACAGCGTGAGCTATGAGAAAGCGCCACGCTTCCCGAAGG
GAGAAAGGCGGACAGGTATCCGGTAAGCGGCAGGGTCGGAACAGGAGAGCGCACGAGGGAGCTTCCAGGGGGAAACGCCTGGTA
TCTTTATAGTCCTGTCGGGTTTCGCCACCTCTGACTTGAGCGTCGATTTTTGTGATGCTCGTCAGGGGGGCGGAGCCTATGGAA
AAACGCCAGCAACGCGGCCTTTTTACGGTTCCTGGCCTTTTGCTGGCCTTTTGCTCACATGTTCTTTCCTGCGTTATCCCCTGA
TTCTGTGGATAACCGTATTACCGCCTTTGAGTGAGCTGATACCGCTCGCCGCAGCCGAACGACCGAGCGCAGCGAGTCAGTGAG
CGAGGAAGCGGAAGAGCGCCCAATACGCAAACCGCCTCTCCCCGCGCGTTGGCCGATTCATTAATGCAGCTGGCACGACAGGTT
TCCCGACTGGAAAGCGGGCAGTGAGCGCAACGCAATTAATGTGAGTTAGCTCACTCATTAGGCACCCCAGGCTTTACACTTTAT
GCTTCCGGCTCGTATGTTGTGTGGAATTGTGAGCGGATAACAATTTCACACAGGAAACAGCTATGGCCATGATTACGCCAAGCTT
GCATGCCTGCATTATAAACTCAAGCTTTTAATTAATTTCGATGATGCATAACAATTTTCACCGTTAGACATCTTCACCACACCT
TTTGTCGTATCTAGCTCATGAATATTTTTCTTATTCACAATATACGACCTGTGGCACCGATAAAATGATTCGTCCAACATTTTC
TCGATATTCTTCAACTTACCATAAAATTCCACTTGGCGATTTTTGCCATGTAAAATTACTTTATGAATAGTTGGCGCTGTCTCA
AAAAACAAGATATCGTCTAACAGTTCATGAATAATCTTCTTATCCGACACTTTAAACGTAAAATACTTCTGCATATCTTGATCA
TTGGAAATCCGTTCTTCTGCTTGCTTCATACAGGCAAGTACCCGGTCATGCAGCAAATCAATATCGTCTTTAATAATGTAATCA
AGCGCTTCCACCTTATACGTGAAAGTCATATAACTTAACTCCGCATGTGTTGTAATAAAAATAATGAAACCACGCGGATCAAAC
TTCCGAATTTCCTGAGCTAATTCAAAACCATTCATGTCCGGCTGCCCCAAATCAATGTCTAAAAAATAAAGCCCCATACCTTGA
TGTGTAGGCATTCGTGATACTAACTCAAACGGATCTCCTGTTGAAAGTTCTAACTTCATATCAAAATGTTCAACCATAATATAG
TCTTCAATATATTTCGTTAACCTTTCTCGCTGCATTCTGTTATCTTCACAAATAAAAACCGGTAGCATAGTAGTTCCTCCTTAT
CTACAGATGCATTTTATTTCATATAGTAAGTACATCACCTATTAGTTTGTTGTTTAAACAAACTAACTTATTTTCATCTTATAT
```

```
AACCTCGTCAGTATTTTCAATATTTTTTTTAGTTTTTTATGAACACATTAGATATAATAAAGGGAAGATTCGCTATGTACTATG
TTGATACTTAATTTAAAGATTAAACAAATGGAGTGGATGAAGTGGATATCGCTGATCAAACCTTTGTCAAAAAAGTAAATCAAA
AGTTATTATTAAAAGAAATCCTTAAAAATTCACCTATTTCAAGAGCAAAATTATCTGAAATGACTGGATTAAATAAATCAACTG
TCTCATCACAGGTAAACACGTTAATGAAAGAAAGTATGGTATTTGAAATAGGTCAAGGACAATCAAGTGGCGGAAGAAGACCTG
TCATGCTTGTTTTTAATAAAAAGGCAGGATACTCCGTTGGAATAGATGTTGGTGTGGATTATATTAATGGCATTTTAACAGACC
TTGAAGGAACAATCGTTCTTGATCAATACCGCCATTTGGAATCCAATTCTCCAGAAATAACGAAAGACATTTTGATTGATATGA
TTCATCACTTTATTACGCAAATGCCCCAATCTCCGTACGGGCTTATTGGTATAGGTATTTGCGTGCCTGGACTCATTGATAAAG
ATCAAAAAATTGTTTTCACTCCGAACTCCAACTGGAGAGATATTGACTTAAAATCTTCGATACAAGAGAAGTACAATGTGCCTG
TTTTTATTGAAAATGAGGCAAATGCTGGCGCATATGGAGAAAAAGTATTTGGAGCTGCAAAAAAATCACGATAACATTATTTACG
TAAGTATCAGCACAGGAATAGGGATCGGTGTTATTATCAACAATCATTTATATAGAGGAGTAAGCGGCTTCTCTGGAGAAATGG
GACATATGACAATAGACTTTAATGGTCCTAAATGCAGTTGCGGAAACCGAGGATGCTGGGAATTGTATGCTTCAGAGAAGGCTT
TATTAAAATCTCTTCAGACCAAAGAGAAAAAACTGTCCTATCAAGATATCATAAACCTCGCCCATCTGAATGATATCGGAACCT
TAAATGCATTACAAAATTTTGGATTCTATTTAGGAATAGGCCTTACCAATATTCTAAATACTTTCAACCCACAAGCCGTAATTT
TAAGAAATAGCATAATTGAATCGCATCCTATGGTTTTAAATTCAATGAGAAGTGAAGTATCATCAAGGGTTTATTCCCAATTAG
GCAATAGCTATGAATTATTGCCATCTTCCTTAGGACAGAATGCACCGGCATTAGGAATGTCCTCCATTGTGATTGATCATTTTC
TGGACATGATTACAATGTAATTTTTTATGGAATGGACAGCTCATCTTTAAAGATGAGTTTTTTTATTCTAGGAGTATTTCTGAA
GCAATAGTGACATGGCACCTTCTCATATGAAAAAGGAGTTCTAAAATAGAAATCTCCTTTTTCATGTGCAAATTATTTTTCTTT
ATAACGAAAATATCTAAAGTCGGCCCATTCACTGGCCGTCGTTTTACCGAAATACGGGCAGACATGGCCTGCCCGGTTAATGCC
TGGCTCTTCAAACAGAACAAGACGATTAATAAAATGGAAAAGCCAACTGTGAACATTCTTTTGCAGTTGGTTTTTTTTATGAAT
AAACTCACTTAAATAAAATATTAATATGGGTTTTTAAGCCTATTATACAGTCCTTTTTACACGTTCGAATATTCTGAAATGTAT
CCAAAAAATCGGCAATTCTGCTCTTTTTTGGTCGTTCATGACAAGAATCGGACATTTCATTACATTTTTGGTTATTATGGGTAA
ATTCGTTGTAAAATATTAGTGGAGGTGAATTAGGATCACAGAGATGTGGTCAGTCAGTCTTAATTAACCAAGGAGGTGAAATGT
ACAATGAAAGGAGAAGAACTTTTCACTGGAGTTGTCCCAATTCTTGTTGAATTAGATGGTGATGTTAATGGGCACAAATTTTCT
GTCAGTGGAGAGGGTGAAGGTGATGCAACATACGGAAAACTTACCCTTAAATTTATTTGCACTACTGGAAAACTACCTGTTCCA
TGGCCAACACTTGTCACTACTTTCGCGTATGGTCTTCAATGCTTTGCGAGATACCCAGATCATATGAAACAGCATGACTTTTTC
AAGAGTGCCATGCCCGAAGGTTATGTACAGGAAAGAACTATATTTTTCAAAGATGACGGGAACTACAAGACACGTGCTGAAGTC
AAGTTTGAAGGTGATACCCTTGTTAATAGAATCGAGTTAAAAGGTATTGATTTTAAAGAAGATGGAAACATTCTTGGACACAAA
TTGGAATACAACTATAACTCACACAATGTATACATCATGGCAGACAAACAAAAGAATGGAATCAAAGTTAACTTCAAAATTAGA
CACAACATTGAAGATGGAAGCGTTCAACTAGCAGACCATTATCAACAAAATACTCCAATTGGCGATGGCCCTGTCCTTTTACCA
GACAACCATTACCTGTCCACACAATCTAAGCTTTCGAAAGATCCCAACGAAAAGAGAGACCACATGGTCCTTCTTGAGTTTGTA
ACAGCTGCTGGGATTACACATGGCATGGATGAACTATACAAATAACCTAGGCTGCTGCCACCGCTGAGCAATAACTAGCATAAC
CCCTTGGGGCCTCTAAACGGGTCTTGAGGGGTTTTTTGAACGTCGTGACTGGGAAAACCCTGGCGTTACCCAACTTAATCGCCT
TGCAGCACATCCCCCTTTCGCCAGCTGGCGTAATAGCGAAGAGGCCCGCACCGATCGCCCTTCCCAACAGTTGCGCAGCCTGAA
TGGCGAATGGCGCCTGATGCGGTATTTTCTCCTTACGCATCTGTGCGGTATTTCACACCGCATATGGTGCACTCTCAGTACAAT
CTGCTCTGATGCCGCATAGTTAAGCCAGCCCCGACACCCGCCAACACCCGCTGACGCGCCCTGACGGGCTTGTCTGCTCCCGGC
ATCCGCTTACAGACAAGCTGTGACCGTCTCCGGGAGCTGCATGTGTCAGAGGTTTTCACCGTCATCACCGAAACGCGCGAGACG
AAAGGGCCTCGTGATACGCCTATTTTTATAGGTTAATGTCATGATAATAATGGTTTCTTAGACGTCAGGTGGCACTTTTCGGGG
AAATGTGCGCGGAACCCCTATTTGTTTATTTTTCTAAATACATTCAAATATGTATCCGCTCATGAGACAATAACCCTGATAAAT
GCTTCAATAATCCATCCTCCAAAGTTGGAGAGTGAGTTTTATGTCGCAAATATTAATGTTTCTGGTGAACCTTATCAAATTTTC
GTTGATTTAATAGAAACATAGCGGTAAAATTAGCAGTAACTTAATAGAACGGAAATGAAAAAAGCCACTCTCATATGCTATTGG
CTACCAACCTTTAGCGAGAATGACTTAATCCTGTACAGCCATACAGGACTTCGACTTATAAGAGGCGCCAACTTCAAATAAGTT
ATTTGCCTTGTTTTCGCGAACAAGGCTTATTAGATACACCTATTGTACCGTTACTCTACGAATATTTCAAGTAGTAATTACTAG
CATTGTCCGTTACTCTACGAATATTTCAAGTAGTAATTACTAGCATTGTCCGTTACTCTACGAATATTTCAAGTAGTAATTACT
AGCATTGTCATATACATAATAAAACGGATATAAAAGGGCGTTTTCTATACCTAGAAGTCTTGTAAATGTACAGGGCGTTTAGAT
ATAGAGAACGCCCTTTTTGTGTTCCGTTCCAGTGGAAGCTACCACTTTAAAAAGATGGTCTAGTGTAGCCAATGCAGGAGAGTA
CACTCGGATATCAGTTGTCGTTGCATTCAACTGTCTGACGTAAGCGAGGTAAAGGACACAAGCCTTGCATAAAACAAGCCTACG
GGATGTAAATCCTAATAATGATGATAACCAAGACGTTAGCGGCAAAAAGTGTTGGGGGTTCAAAATAAGACATGATTGTGCGAC
TGGAGTTAAACAGTTACTCGTAAGCGGCGATCATGACACTGATTCACGGCTATTCTTGTACAAGCTAGCTTTATTACAAGGATA
TGCGGGTTATATAGCGAATCACCCGAAAGGGAACGGTGTTGGGCGTGAGAAACGCACCGTACGGCGCAATACAATGCCAATAAG
CTATATACGGACGGTATAGTAGTTTTGTAAGCTATAACCGTTTGTCGTCAATGCAACCAATCTCAATTCGAGACCTCGGCATCT
AAGCCAGTACGAATGAGTGGGCGTTTTAACCTCGTAAATTTTCAACAGGGGTTACTATGCCCAAAACTACATTCAGATTTCCTA
ACAAACTCGCCAGTATGAAAACCTTAAGACCTTAAAGTCAAGGGATTTGAAGGATTTTAACCTCGATTAGCAAAAAATGTAGAG
TACTGAAGCAACTACCATTAACTAAGATAGTGGGGGATTGAGGAAGAATCCAGAGCTGTTTAAATCAAGTGAAAGACAAGATGA
AATTAAAAGAATAGTGAAAGATAGGGGAGTGGTTCTCTATGAGAAAGGAAATGGCTAGAGAACAAAGGCAGCGGTTTATTGATC
TATTGTTAGACTTTATGGTAAAGAATCCTCATTTATTTGTTAATGGTACAGAGGATGAAAGTAATAATGTTGTTACAAAATGTA
ATAGTGATATTAAAGAGGTTGCGGAGTCATATTTAACTCTTTTATAGTGAGAGGGTTAAAACTAATTAATATGTATTAAGGCCC
AATGTTGGAATTATTGTATTTCACTAGGCAACCTACTTACTAAAAGTAAGATTATCCATTAGTGGATGTTATAATATTGGGTTT
TTTAACACAATAATCATCGCCTTTCGGTGTCGTTTGATAGAAAAGTAACCATTAGCGATGAAAAAGTCAATATAAAAAGCCATC
CGTAAAAAACGGATGGCTTACCGTACATAGGATCGTTGGTAGGGCGGCGTATCCTACATCTCTGGTAACTTACCTAGCCAATCA
AATGCTTGAGAACGGCGGTTAGATAAGCGCGTGGGGAACCTTTCCCACCTCAAAGATCCTATATCATTATTATGTTACTTTCTA
CAGGTAGTATACCATGTTCTTATATTTTAGTAAACTCCCCGTTAGCTTAACAGGTCTTTGTAAGCAATTAAACGTCCACTATTC
AATCGTCTTTGGATTTTCGCAGGACCGTTTTTTAGATCGAACATAGTTGATAAGAACAAATAACCGCTTGGGTCCAACTTTATA
GCAATTAGTATATGGTCATTTAAAATCTTTACCAATTCAACGCTATTAGGTTCTTTAGGATTTTGCCCGACATAGTCGGGGTGT
```

```
TCAACGATATCTTTTATGTGCGATGAATATTTTTCATAAATACCAGGATGTTGTTTCTTTACGTGCTTTATAAATCCGGGAAAC
ATTTTTACATCGTTAGAAGTGCAAGTCAAGTTATATGTATCTATAATGATTTGTGGAAGTTTTGCCACAACAGTTGGTTTATTT
ACAATCTTTTTTTTATTAGCCGTCAAATTTCTCCCTCATCTCGTCTCTTTATATCTTTATTTTATCATAAAGGAGTATTTGAAC
CGTCGCGCGGGACAGGTTTATGATAGGGATATTTTATTGAATAATTGATGGTATAAGGGACTTTCATGCTTGGAAAGTGGGGAT
TATGAATTAGATGCTTGTCCACAATATGTTCCAATGTAATTAAAATTTATGTTCCCACCTTGACCAAACATCACGTCCATACTT
AAATCGTCCCTCCTTTAATAGGTAAAATATTAATTTACCTTAATAAAAAAATAATGGATAATAGTATTCGTCTGAATTTATATA
ATCAGGGGGAACTATTGATGCTGGGGATACTATTTACAGCGGCGCCATCTACTGATGTCGTAAAGGATTTGCAAGATAAAGTTA
TATCATTGCAGGATCATGAGGTAGCGTTTTTGAACACCACGATATCTAATATGTTGATCCCCGAAGCAAACTTAAGAGTGTGTT
GATAGTGCAGTATCTTAAAATTTTGTGTATAATAGGAATTGAAGTTAAATTAGATGCTAAAAATTTGTAATTAAGAAGGAGGGA
TTCGTCATGTTGGTATTCCAAATGCGTAATGTAGATAAAACATCTACTGTTTTGAAACAGACTAAAAACAGTGATTACGCAGAT
AAATAAATACGTTAGATTAATTCCTACCAGTGACTAATCTTATGACTTTTTAAACAGATAACTAAAATTACAAACAAATCGTTT
AACTTCTGTATTTATTTACAGATGTAATCACTTCAGGAGTAATTACATGAACAAAAATATAAAATATTCTCAAAACTTTTTAAC
GAGTGAAAAAGTACTCAACCAAATAATAAAACAATTGAATTTAAAAGAAACCGATACCGTTTACGAAATTGGAACAGGTAAAGG
GCATTTAACGACGAAACTGGCTAAAATAAGTAAACAGGTAACGTCTATTGAATTAGACAGTCATCTATTCAACTTATCGTCAGA
AAAATTAAAACTGAACATTCGTGTCACTTTAATTCACCAAGATATTCTACAGTTTCAATTCCCTAACAAACAGAGGTATAAAAT
TGTTGGGAGTATTCCTTACCATTTAAGCACACAAATTATTAAAAAAGTGGTTTTTGAAAGCCATGCGTCTGACATCTATCTGAT
TGTTGAAGAAGGATTCTACAAGCGTACCTTGGATATTCACCGAACACTAGGGTTGCTCTTGCACACTCAAGTCTCGATTCAGCA
ATTGCTTAAGCTGCCAGCGGAATGCTTTCATCCTAAACCAAAAGTAAACAGTGTCTTAATAAAACTTACCCGCCATACCACAGA
TGTTCCAGATAAATATTGGAAGCTATATACGTACTTTGTTTCAAAATGGGTCAATCGAGAATATCGTCAACTGTTTACTAAAAA
TCAGTTTCATCAAGCAATGAAACACGCCAAAGTAAACAATTTAAGTACCATTACTTATGAGCAAGTATTGTCTATTTTTAATAG
TTATCTATTATTTAACGGGAGGAAATAATTCTATGAGTCGCTTTTTTAAATTTGGAAAGTTACACGTTACTAAAGGGAATGGAG
ATAAATTATTAGATATACTACTGACAGCTTCCAAGAAGCTAAAGAGGTCCCTAGCGCCTACGGGGAATTTGGGGTACATTGAAA
AAGGAAGAGTATGAGTATTCAACATTTCCGTGTCGCCCTTATTCCCTTTTTTGCGGCATTTTGCCTTCCTGTTTTTGCTCACCC
AGAAACGCTGGTGAAAGTAAAAGATGCTGAAGATCAGTTGGGTGCACGAGTGGGTTACATCGAACTGGATCTCAACAGCGGTAA
GATCCTTGAGAGTTTTCGCCCCGAAGAACGTTTTCCAATGATGAGCACTTTTAAAGTTCTGCTATGTGGCGCGGTATTATCCCG
TATTGACGCCGGGCAAGAGCAACTCGGTCGCCGCATACACTATTCTCAGAATGACTTGGTTGAGTACTCACCAGTCACAGAAAA
GCATCTTACGGATGGCATGACAGTAAGAGAATTATGCAGTGCTGCCATAACCATGAGTGATAACACTGCGGCCAACTTACTTCT
GACAACGATCGGAGGACCGAAGGAGCTAACCGCTTTTTTGCACAACATGGGGGATCATGTAACTCGCCTTGATCGTTGGGAACC
GGAGCTGAATGAAGCCATACCAAACGACGAGCGTGACACCACGATGCCTGTAGCAATGGCAACAACGTTGCGCAAACTATTAAC
TGGCGAACTACTTACTCTAGCTTCCCGGCAACAATTAATAGACTGGATGGAGGCGGATAAAGTTGCAGGACCACTTCTGCGCTC
GGCCCTTCCGGCTGGCTGGTTTATTGCTGATAAATCTGGAGCCGGTGAGCGTGGGTCTCGCGGTATCATTGCAGCACTGGGGCC
AGATGGTAAGCCCTCCCGTATCGTAGTTATCTACACGACGGGGAGTCAGGCAACTATGGATGAACGAAATAGACAGATCGCTGA
GATAGGTGCCTCACTGATTAAGCATTGGTAACTGTCAGACCAAGTTTACTCATATATACTTTAGATTGATTTAAAACTTCATTT
TTAATTTAAAAGGATCTAGGTGAAGATCCTTTTTGATAATCTCATGACCAAAATCCCTTAACGTGAGTTTTCGTTCCACTGAGC
GTCAGACCCCGTAGAAAAGATCAAAGGATCTTC-3'
```

pXyl-Sa-agrCA-LysT

```
5'-
GAAGATCCTTTGATCTTTTCTACGGGGTCTGACGCTCAGTGGAACGAAAACTCACGTTAAGGGATTTTGGTCATGAGATTATCA
AAAAGGATCTTCACCTAGATCCTTTTAAATTAAAAATGAAGTTTTAAATCAATCTAAAGTATATATGAGTAAACTTGGTCTGAC
AGTTACCAATGCTTAATCAGTGAGGCACCTATCTCAGCGATCTGTCTATTTCGTTCATCCATAGTTGCCTGACTCCCCGTCGTG
TAGATAACTACGATACGGGAGGGCTTACCATCTGGCCCCAGTGCTGCAATGATACCGCGAGACCCACGCTCACCGGCTCCAGAT
TTATCAGCAATAAACCAGCCAGCCGGAAGGGCCGAGCGCAGAAGTGGTCCTGCAACTTTATCCGCCTCCATCCAGTCTATTAAT
TGTTGCCGGGAAGCTAGAGTAAGTAGTTCGCCAGTTAATAGTTTGCGCAACGTTGTTGCCATTGCTACAGGCATCGTGGTGTCA
CGCTCGTCGTTTGGTATGGCTTCATTCAGCTCCGGTTCCCAACGATCAAGGCGAGTTACATGATCCCCCATGTTGTGCAAAAAA
GCGGTTAGCTCCTTCGGTCCTCCGATCGTTGTCAGAAGTAAGTTGGCCGCAGTGTTATCACTCATGGTTATGGCAGCACTGCAT
AATTCTCTTACTGTCATGCCATCCGTAAGATGCTTTTCTGTGACTGGTGAGTACTCAACCAAGTCATTCTGAGAATAGTGTATG
CGGCGACCGAGTTGCTCTTGCCCGGCGTCAATACGGGATAATACCGCGCCACATAGCAGAACTTTAAAAGTGCTCATCATTGGA
AAACGTTCTTCGGGGCGAAAACTCTCAAGGATCTTACCGCTGTTGAGATCCAGTTCGATGTAACCCACTCGTGCACCCAACTGA
TCTTCAGCATCTTTTACTTTCACCAGCGTTTCTGGGTGAGCAAAAACAGGAAGGCAAAATGCCGCAAAAAAGGGAATAAGGGCG
ACACGGAAATGTTGAATACTCATACTCTTCCTTTTTCAATGTACCCCAAATTCCCCGTAGGCGCTAGGGACCTCTTTAGCTTCT
TGGAAGCTGTCAGTAGTATATCTAATAATTTATCTCCATTCCCTTTAGTAACGTGTAACTTTCCAAATTTAAAAAAGCGACTCA
TAGAATTATTTCCTCCCGTTAAATAATAGATAACTATTAAAAATAGACAATACTTGCTCATAAGTAATGGTACTTAAATTGTTT
ACTTTGGCGTGTTTCATTGCTTGATGAAACTGATTTTTAGTAAACAGTTGACGATATTCTCGATTGACCCATTTTGAAACAAAG
TACGTATATAGCTTCCAATATTTATCTGGAACATCTGTGGTATGGCGGGTAAGTTTTATTAAGACACTGTTTACTTTTGGTTTA
GGATGAAAGCATTCCGCTGGCAGCTTAAGCAATTGCTGAATCGAGACTTGAGTGTGCAAGAGCAACCCTAGTGTTCGGTGAATA
TCCAAGGTACGCTTGTAGAATCCTTCTTCAACAATCAGATAGATGTCAGACGCATGGCTTTCAAAAACCACTTTTTTAATAATT
TGTGTGCTTAAATGGTAAGGAATACTCCCAACAATTTTATACCTCTGTTTGTTAGGGAATTGAAACTGTAGAATATCTTGGTGA
ATTAAAGTGACACGAATGTTCAGTTTTAATTTTTCTGACGATAAGTTGAATAGATGACTGTCTAATTCAATAGACGTTACCTGT
TTACTTATTTTAGCCAGTTTCGTCGTTAAATGCCCTTTACCTGTTCCAATTTCGTAAACGGTATCGGTTTCTTTTAAATTCAAT
TGTTTTATTATTTGGTTGAGTACTTTTTCACTCGTTAAAAAGTTTTGAGAATATTTTATATTTTTGTTCATGTAATTACTCCTG
AAGTGATTACATCTGTAAATAAATACAGAAGTTAAACGATTTGTTTGTAATTTTAGTTATCTGTTTAAAAAGTCATAAGATTAG
```

```
TCACTGGTAGGAATTAATCTAACGTATTTATTTATCTGCGTAATCACTGTTTTTAGTCTGTTTCAAAACAGTAGATGTTTTATC
TACATTACGCATTTGGAATACCAACATGACGAATCCCTCCTTCTTAATTACAAATTTTTAGCATCTAATTTAACTTCAATTCCT
ATTATACACAAAATTTTAAGATACTGCACTATCAACACACTCTTAAGTTTGCTTCGGGGATCAACATATTAGATATCGTGGTGT
TCAAAAACGCTACCTCATGATCCTGCAATGATATAACTTTATCTTGCAAATCCTTTACGACATCAGTAGATGGCGCCGCTGTAA
ATAGTATCCCCAGCATCAATAGTTCCCCCTGATTATATAAATTCAGACGAATACTATTATCCATTATTTTTTTATTAAGGTAAA
TTAATATTTTACCTATTAAAGGAGGGACGATTTAAGTATGGACGTGATGTTTGGTCAAGGTGGGAACATAAATTTTAATTACAT
TGGAACATATTGTGGACAAGCATCTAATTCATAATCCCCACTTTCCAAGCATGAAAGTCCCTTATACCATCAATTATTCAATAA
AATATCCCTATCATAAACCTGTCCCGCGCGACGGTTCAAATACTCCTTTATGATAAAATAAAGATATAAAGAGACGAGATGAGG
GAGAAATTTGACGGCTAATAAAAAAAAGATTGTAAATAAACCAACTGTTGTGGCAAAACTTCCACAAATCATTATAGATACATA
TAACTTGACTTGCACTTCTAACGATGTAAAAATGTTTCCCGGATTTATAAAGCACGTAAAGAAACAACATCCTGGTATTTATGA
AAAATATTCATCGCACATAAAAGATATCGTTGAACACCCCGACTATGTCGGGCAAAATCCTAAAGAACCTAATAGCGTTGAATT
GGTAAAGATTTTAAATGACCATATACTAATTGCTATAAAGTTGGACCCAAGCGGTTATTTGTTCTTATCAACTATGTTCGATCT
AAAAAACGGTCCTGCGAAAATCCAAAGACGATTGAATAGTGGACGTTTAATTGCTTACAAAGACCTGTTAAGCTAACGGGGAGT
TTACTAAAATATAAGAACATGGTATACTACCTGTAGAAAGTAACATAATAATGATATAGGATCTTTGAGGTGGGAAAGGTTCCC
CACGCGCTTATCTAACCGCCGTTCTCAAGCATTTGATTGGCTAGGTAAGTTACCAGAGATGTAGGATACGCCGCCCTACCAACG
ATCCTATGTACGGTAAGCCATCCGTTTTTTACGGATGGCTTTTTATATTGACTTTTTCATCGCTAATGGTTACTTTTCTATCAA
ACGACACCGAAAGGCGATGATTATTGTGTTAAAAAACCCAATATTATAACATCCACTAATGGATAATCTTACTTTTAGTAAGTA
GGTTGCCTAGTGAAATACAATAATTCCAACATTGGGCCTTAATACATATTAATTAGTTTTAACCCTCTCACTATAAAAGAGTTA
AATATGACTCCGCAACCTCTTTAATATCACTATTACATTTTGTAACAACATTATTACTTTCATCCTCTGTACCATTAACAAATA
AATGAGGATTCTTTACCATAAAGTCTAACAATAGATCAATAAACCGCTGCCTTTGTTCTCTAGCCATTTCCTTTCTCATAGAGA
ACCACTCCCCTATCTTTCACTATTCTTTTAATTTCATCTTGTCTTTCACTTGATTTAAACAGCTCTGGATTCTTCCTCAATCCC
CCACTATCTTAGTTAATGGTAGTTGCTTCAGTACTCTACATTTTTTGCTAATCGAGGTTAAAATCCTTCAAATCCCTTGACTTT
AAGGTCTTAAGGTTTTCATACTGGCGAGTTTGTTAGGAAATCTGAATGTAGTTTTGGGCATAGTAACCCCTGTTGAAAATTTAC
GAGGTTAAAACGCCCACTCATTCGTACTGGCTTAGATGCCGAGGTCTCGAATTGAGATTGGTTGCATTGACGACAAACGGTTAT
AGCTTACAAAACTACTATACCGTCCGTATATAGCTTATTGGCATTGTATTGCGCCGTACGGTGCGTTTCTCACGCCCAACACCG
TTCCCTTTCGGGTGATTCGCTATATAACCCGCATATCCTTGTAATAAAGCTAGCTTGTACAAGAATAGCCGTGAATCAGTGTCA
TGATCGCCGCTTACGAGTAACTGTTTAACTCCAGTCGCACAATCATGTCTTATTTTGAACCCCCAACACTTTTTGCCGCTAACG
TCTTGGTTATCATCATTATTAGGATTTACATCCCGTAGGCTTGTTTTATGCAAGGCTTGTGTCCTTTACCTCGCTTACGTCAGA
CAGTTGAATGCAACGACAACTGATATCCGAGTGTACTCTCCTGCATTGGCTACACTAGACCATCTTTTTAAAGTGGTAGCTTCC
ACTGGAACGGAACACAAAAAGGGCGTTCTCTATATCTAAACGCCCTGTACATTTACAAGACTTCTAGGTATAGAAAACGCCCTT
TTATATCCGTTTTATTATGTATATGACAATGCTAGTAATTACTACTTGAAATATTCGTAGAGTAACGGACAATGCTAGTAATTA
CTACTTGAAATATTCGTAGAGTAACGGACAATGCTAGTAATTACTACTTGAAATATTCGTAGAGTAACGGTACAATAGGTGTAT
CTAATAAGCCTTGTTCGCGAAAACAAGGCAAATAACTTATTTGAAGTTGGCGCCTCTTATAAGTCGAAGTCCTGTATGGCTGTA
CAGGATTAAGTCATTCTCGCTAAAGGTTGGTAGCCAATAGCATATGAGAGTGGCTTTTTTCATTTCCGTTCTATTAAGTTACTG
CTAATTTTACCGCTATGTTTCTATTAAATCAACGAAAATTTGATAAGGTTCACCAGAAACATTAATATTTGCGACATAAAACTC
ACTCTCCAACTTTGGAGGATGGATTATTGAAGCATTTATCAGGGTTATTGTCTCATGAGCGGATACATATTTGAATGTATTTAG
AAAAATAAACAAATAGGGGTTCCGCGCACATTTCCCCGAAAAGTGCCACCTGACGTCTAAGAAACCATTATTATCATGACATTA
ACCTATAAAAATAGGCGTATCACGAGGCCCTTTCGTCTCGCGCGTTTCGGTGATGACGGTGAAAACCTCTGACACATGCAGCTC
CCGGAGACGGTCACAGCTTGTCTGTAAGCGGATGCCGGGAGCAGACAAGCCCGTCAGGGCGCGTCAGCGGGTGTTGGCGGGTGT
CGGGGCTGGCTTAACTATGCGGCATCAGAGCAGATTGTACTGAGAGTGCACCATATGCGGTGTGAAATACCGCACAGATGCGTA
AGGAGAAAATACCGCATCAGGCGCCATTCGCCATTCAGGCTGCGCAACTGTTGGGAAGGGCGATCGGTGCGGGCCTCTTCGCTA
TTACGCCAGCTGGCGAAAGGGGGATGTGCTGCAAGGCGATTAAGTTGGGTAACGCCAGGGTTTTCCCAGTCACGACGTTCAAAA
AACCCCTCAAGACCCGTTTAGAGGCCCCAAGGGGTTATGCTAGTTATTGCTCAGCGGTGGCAGCAGCCTAGGTTATTACTTTAT
AGTTCCCCAAAGAACACCTAAAGTATTAGTAGATTTATTCCATGTTCTTACAGGCAAGTAAATACGTTGGCCACTGTTACCTGT
ATAACCTACCCAAACATGACCGTCTTGTTTCATCACTTCATCATAATGAATTGTTTGACCTGCTTTTAAGACTCCTGACTGCGG
CATGCTTCTAAATGGACCAGTCGTTCTTGTTATTATATCTGTATTAGGTGTGAAGCTAGCTGACTCTGATTTATATAGTGTGCC
ATATTTGTTTGTTTTCCAACCTGTATTCGGCGTTGGAGTTACTGTACCACCTGCTTTTCCATATCCTGCGCTCTTTAAGAAAGG
CATTGGATCTTGGGCAGTTGAATTTGAAAATGAATTAACCATTCTTTGGAAGTGTAAATGTGGTGCTGTAGAATAACCAGTGCT
TCCAGACCAACCGATTATTTGACCAGCTTTGACATAATCTCCTACTTTAACATTATATTTACTTAGATGCATATACCATTGTCT
ATGCACTCCATCATTTTCAATAAGACCTATTTGATTACCTCCTCCGTAATTACTCCAACCAGCTTCAACTATTTTTCCGCTTGA
AATAGCTTTTACTGGTGTTCCAATATTCATAAAAAAATCAACTCCGTAGTGCATACCGCCATTTATACCTAATGGATAAGGACC
GTAACCATATCCTTTTTTGTAATTATTCAACCATTGTGCTGAATGTTCATGTGTGCTTCCAGCACTCGCAGCCGCCGGTCCTGC
CAGAACCAAATGAAACAGCAATAAAAATCCAGCGAATAACGGCAGTAAAGAGGTTTTGAATCGTTTTGCAAACATTGTACATTT
CACCTCCTTGGTTAATTAAGACTGACTGACCACATCTCTGTGATCTAGTTATATTAAAACATGCTAAAAGCATTTATTTTCCAA
TTTTTCTTAACTAGTCGTTTTTTATTCTTAACTGTAAATTTTTTTATGTTAAAATATTAAATACAAATTACATTTAACAGTTAA
GTATTTATTTCCTACAGTTAGGCAATATAATGATAAAAGATTGTACTAAATCGTATAATGACAGTGAAGCCAGGCATTAACCGG
GCAGGCCATGTCTGCCCGTATTTCGGTAAAACGACGGCCAGTGAATTGGCCGACTTTAGATATTTTCGTTATAAAGAAAAATAA
TTTGCACATGAAAAAGGAGATTTCTATTTTAGAACTCCTTTTTCATATGAGAAGGTGCCATGTCACTATTGCTTCAGAAATACT
CCTAGAATAAAAAAACTCATCTTTAAAGATGAGCTGTCCATTCCATAAAAAATTACATTGTAATCATGTCCAGAAAATGATCAA
TCACAATGGAGGACATTCCTAATGCCGGTGCATTCTGTCCTAAGGAAGATGGCAATAATTCATAGCTATTGCCTAATTGGGAAT
AAACCCTTGATGATACTTCACTTCTCATTGAATTTAAAACCATAGGATGCGATTCAATTATGCTATTTCTTAAAATTACGGCTT
GTGGGTTGAAAGTATTTAGAATATTGGTAAGGCCTATTCCTAAATAGAATCCAAAATTTTGTAATGCATTTAAGGTTCCGATAT
CATTCAGATGGGCGAGGTTTATGATATCTTGATAGGACAGTTTTTTCTCTTTGGTCTGAAGAGATTTTAATAAAGCCTTCTCTG
```

```
AAGCATACAATTCCCAGCATCCTCGGTTTCCGCAACTGCATTTAGGACCATTAAAGTCTATTGTCATATGTCCCATTTCTCCAG
AGAAGCCGCTTACTCCTCTATATAAATGATTGTTGATAATAACACCGATCCCTATTCCTGTGCTGATACTTACGTAAATAATGT
TATCGTGATTTTTTGCAGCTCCAAATACTTTTTCTCCATATGCGCCAGCATTTGCCTCATTTTCAATAAAAACAGGCACATTGT
ACTTCTCTTGTATCGAAGATTTTAAGTCAATATCTCTCCAGTTGGAGTTCGGAGTGAAAACAATTTTTTGATCTTTATCAATGA
GTCCAGGCACGCAAATACCTATACCAATAAGCCCGTACGGAGATTGGGGCATTTGCGTAATAAAGTGATGAATCATATCAATCA
AAATGTCTTTCGTTATTTCTGGAGAATTGGATTCCAAATGGCGGTATTGATCAAGAACGATTGTTCCTTCAAGGTCTGTTAAAA
TGCCATTAATATAATCCACACCAACATCTATTCCAACGGAGTATCCTGCCTTTTTATTAAAAACAAGCATGACAGGTCTTCTTC
CGCCACTTGATTGTCCTTGACCTATTTCAAATACCATACTTTCTTTCATTAACGTGTTTACCTGTGATGAGACAGTTGATTTAT
TTAATCCAGTCATTTCAGATAATTTTGCTCTTGAAATAGGTGAATTTTTAAGGATTTCTTTTAATAATAACTTTTGATTTACTT
TTTTGACAAAGGTTTGATCAGCGATATCCACTTCATCCACTCCATTTGTTTAATCTTTAAATTAAGTATCAACATAGTACATAG
CGAATCTTCCCTTTATTATATCTAATGTGTTCATAAAAAACTAAAAAAAATATTGAAAATACTGACGAGGTTATATAAGATGAA
AATAAGTTAGTTTGTTTAAACAACAAACTAATAGGTGATGTACTTACTATATGAAATAAAATGCATCTGTAGATAAGGAGGAAC
TACTATGGAATTATTAAATAGTTATAATTTTGTTTTATTCGTATTAACTCAAATGATATTAATGTTTACAATACCAGCTATAAT
TAGTGGTATTAAGTACAGTAAACTTGATTATTTTTTCATCATAGTAATTTCGACATTATCGTTATTTCTATTTAAAATGTTTGA
TAGCGCGTCCTTAATCATATTAACTTCATTTATTATTATAATGTATTTTGTCAAAATCAAATGGTATTCTATTTTGTTGATTAT
GACTTCGCAGATTATTCTATACTGTGCTAACTACATGTATATAGTTATATATGCATATATCACCAAAATTTCTGATAGTATATT
TGTAATATTCCCTAGCTTTTTTGTAGTTTATGTGACTATTAGTATACTATTCTCATATATAATAAATAGAGTTCTCAAAAAAAT
TAGCACACCATATCTAATACTAAACAAAGGATTTTTAATAGTTATTTCGACTATCTTACTGCTTACTTTTTCATTATTTTTCTT
TTATTCACAAATAAACTCGGATGAAGCTAAAGTAATAAGGCAGTATTCTTTTATTTTTATTGGTATCACTATATTTTTAAGTAT
ATTAACATTTGTTATTTCTCAATTTCTCCTTAAAGAGATGAAATATAAACGTAATCAAGAAGAAATTGAAACCTATTATGAATA
TACATTGAAGATTGAAGCTATCAACAACGAAATGCGCAAGTTCCGTCATGATTATGTCAATATCTTAACGACACTTTCAGAATA
CATTCGAGAAGATGACATGCCTGGCCTACGTGATTATTTCAATAAAAATATTGTACCTATGAAAGACAATTTACAAATGAATGC
TATAAAATTAAATGGTATCGAGAATCTTAAAGTACGTGAAATTAAAGGCTTAATTACTGCGAAAATTTTACGTGCACAAGAAAT
GAATATTCCGATTAGTATCGAAATACCCGATGAAGTAAGTAGCATTAACTTGAATATGATCGATTTAAGTCGCAGTATTGGTAT
TATTCTTGATAATGCAATTGAGGCATCAACTGAAATTGATGACCCTATCATTCGCGTTGCATTTATTGAAAGTGAAAATTCAGT
AACGTTTATTGTTATGAATAAATGCGCTGATGATATACCACGCATTCATGAATTGTTCCAAGAAAGTTTTTCTACTAAAGGTGA
AGGTCGTGGTTTAGGTCTATCAACTTTAAAAGAAATTGCTGATAATGCAGACAATGTCTTATTAGATACAATTATCGAAAATGG
TTTCTTTATTCAAAAAGTTGAAATTATTAACAACTAGCCATAAGGATGTGAATGTATGAAAATTTTCATTTGCGAAGACGATCC
AAAACAAAGAGAAAACATGGTTACCATTATTAAAAATTATATAATGATAGAAGAAAAGCCTATGGAAATTGCCCTCGCAACTGA
TAATCCTTATGAGGTGCTTGAGCAAGCTAAAAATATGAATGACATAGGCTGTTACTTTTTAGATATTCAACTTTCAACTGATAT
TAATGGTATCAAATTAGGCAGTGAAATTCGTAAGCATGACCCAGTTGGTAACATTATTTTCGTTACGAGTCACAGTGAACTTAC
CTATTTAACATTTGTCTACAAAGTTGCAGCGATGGATTTTATTTTTAAAGATGATCCAGCTGAATTAAGAACTCGAATTATAGA
CTGTTTAGAAACTGCACATACACGCTTACAATTGTTGTCTAAAGATAATAGCGTTGAAACGATTGAATTAAAACGTGGCAGTAA
TTCAGTGTATGTTCAATATGATGATATTATGTTTTTTGAATCATCAACAAAATCTCACAGACTCATTGCCCATTTAGATAACCG
TCAAATTGAATTTTATGGTAATTTAAAAGAACTGAGTCAATTAGATGATCGTTTCTTTAGATGTCATAATAGCTTTGTCGTCAA
TCGCCATAATATTGAATCTATAGATTCGAAAGAGCGAATTGTCTATTTTAAAAATAAAGAACACTGCTATGCATCGGTGAGAAA
CGTTAAAAAAATATAATGCAGGCATGCAAGCTTGGCGTAATCATGGTCATAGCTGTTTCCTGTGTGAAATTGTTATCCGCTCAC
AATTCCACACAACATACGAGCCGGAAGCATAAAGTGTAAAGCCTGGGGTGCCTAATGAGTGAGCTAACTCACATTAATTGCGTT
GCGCTCACTGCCCGCTTTCCAGTCGGGAAACCTGTCGTGCCAGCTGCATTAATGAATCGGCCAACGCGCGGGGAGAGGCGGTTT
GCGTATTGGGCGCTCTTCCGCTTCCTCGCTCACTGACTCGCTGCGCTCGGTCGTTCGGCTGCGGCGAGCGGTATCAGCTCACTC
AAAGGCGGTAATACGGTTATCCACAGAATCAGGGGATAACGCAGGAAAGAACATGTGAGCAAAAGGCCAGCAAAAGGCCAGGAA
CCGTAAAAAGGCCGCGTTGCTGGCGTTTTTCCATAGGCTCCGCCCCCCTGACGAGCATCACAAAAATCGACGCTCAAGTCAGAG
GTGGCGAAACCCGACAGGACTATAAAGATACCAGGCGTTTCCCCCTGGAAGCTCCCTCGTGCGCTCTCCTGTTCCGACCCTGCC
GCTTACCGGATACCTGTCCGCCTTTCTCCCTTCGGGAAGCGTGGCGCTTTCTCATAGCTCACGCTGTAGGTATCTCAGTTCGGT
GTAGGTCGTTCGCTCCAAGCTGGGCTGTGTGCACGAACCCCCCGTTCAGCCCGACCGCTGCGCCTTATCCGGTAACTATCGTCT
TGAGTCCAACCCGGTAAGACACGACTTATCGCCACTGGCAGCAGCCACTGGTAACAGGATTAGCAGAGCGAGGTATGTAGGCGG
TGCTACAGAGTTCTTGAAGTGGTGGCCTAACTACGGCTACACTAGAAGAACAGTATTTGGTATCTGCGCTCTGCTGAAGCCAGT
TACCTTCGGAAAAAGAGTTGGTAGCTCTTGATCCGGCAAACAAACCACCGCTGGTAGCGGTGGTTTTTTTGTTTGCAAGCAGCA
GATTACGCGCAGAAAAAAAGGATCTCAA-3’
```

Characterization data for _S. aureus_ AIP-III + N-terminal alanine standard.

RP-HPLC
Purity = 97.8%

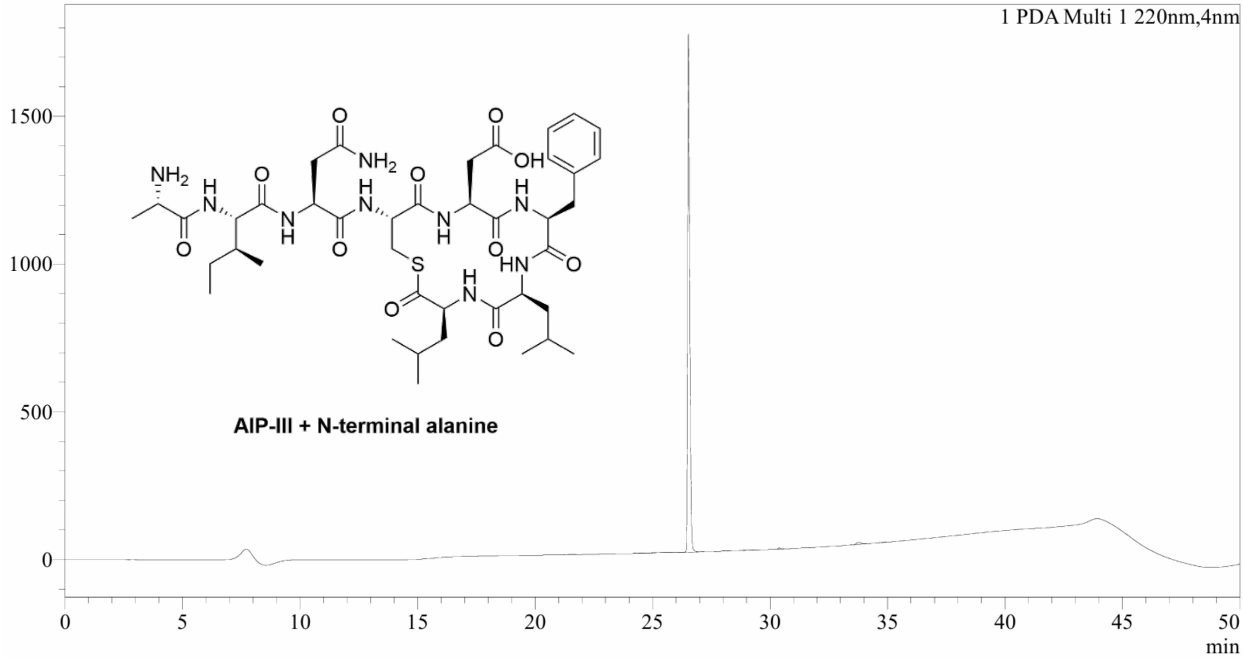

_MS_
$[M+H]^+$ calc. = 890.4441, meas. = 890.4442

See Experimental section for methods.

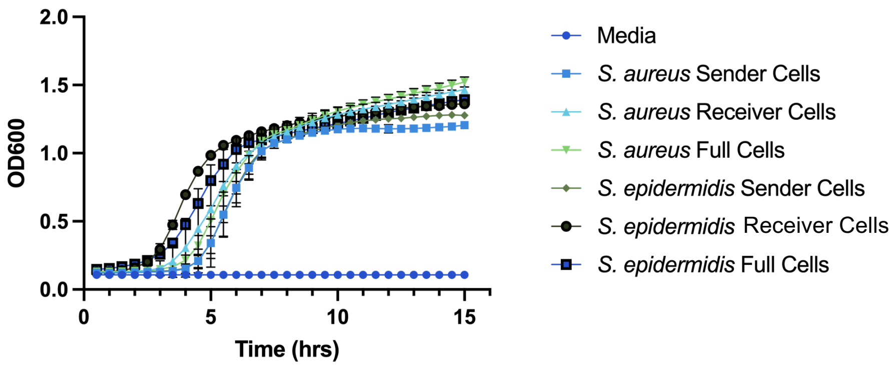

Figure S2. Growth curves for *S. aureus* and *S. epidermidis* sender, receiver, and full cells. Normal growth was generally observed for all the staphylococci derived constructs reported in this study; the *Sa* and *Se* sender cells had slightly lower growth at 15 h relative to the other constructs. Error bars represent the standard deviation of one biological replicate, tested in triplicate (n=3).

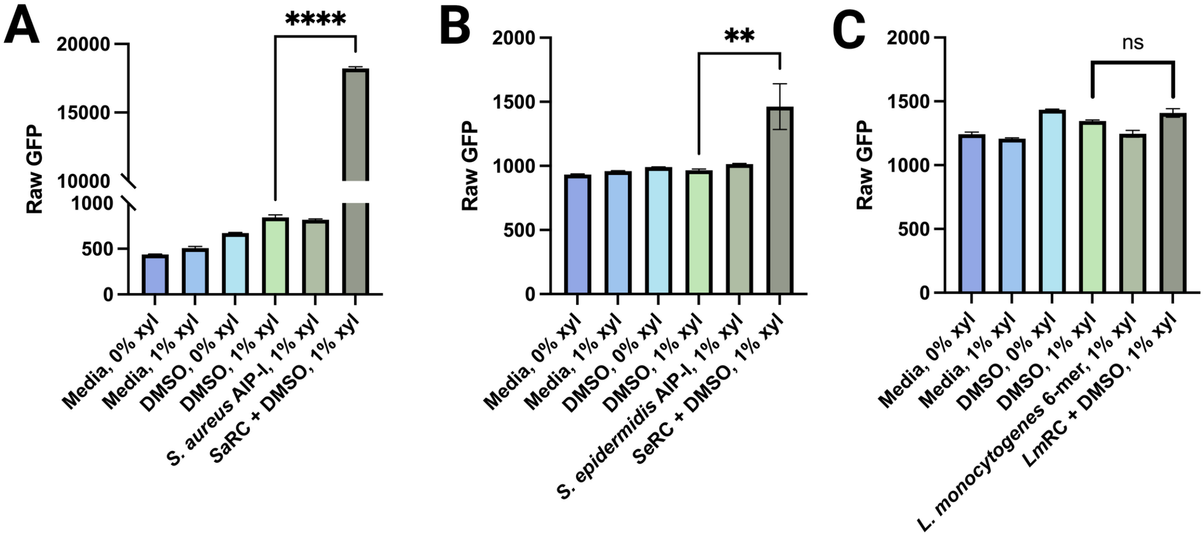

Figure S3. Fluorescence data for GFP-only control *B. subtilis* cells. **(**A) Fluorescence data for *B. subtilis* cells containing the pSa-control-GFP vector. No significant fluorescence was observed with or without xylose, or upon the addition of *S. aureus* AIP-I. A significant increase in fluorescence was observed when compared with background fluorescence of the *Sa* receiver cells (dark olive bar). (B) Fluorescence data for *B. subtilis* cells containing the pSe-control-GFP vector. No significant fluorescence was observed with or without xylose, or upon the addition of *S. epidermidis* AIP-I. A significant increase in fluorescence was observed when compared with background fluorescence of the *Se* receiver cells (dark olive bar). (C) Fluorescence data for *B. subtilis* cells containing the pLm-control-GFP vector. No significant fluorescence was observed with or without xylose, upon the addition of *L. monocytogenes* 6-mer AIP, or when compared to the background fluorescence of the *Lm* receiver cells. Error bars represent the standard error of the mean of three biological replicates tested in triplicate (n=3). Significance values were determined using an unpaired t-test with ** and **** deemed significant with $p < 0.01$ and $p < 0.0001$ respectively.

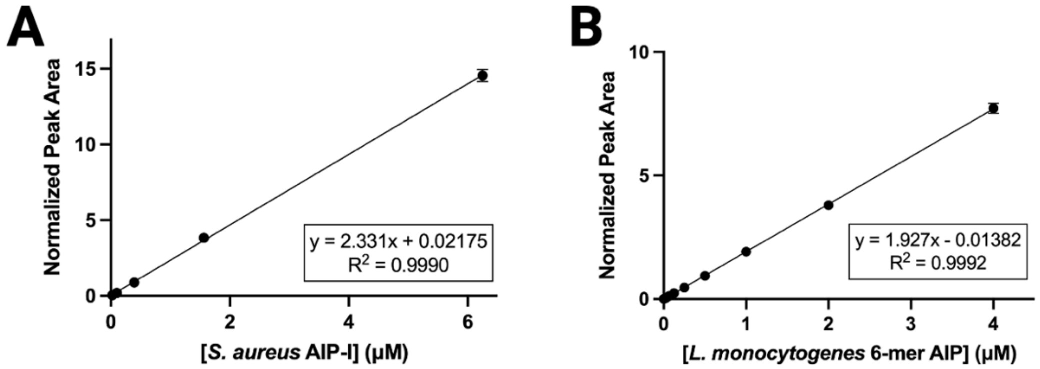

Figure S4. Standard curves for AIP quantification in sender cell supernatants. Curves were generated using synthetic standards of (A) *S. aureus* AIP-I and (B) *L. monocytogenes* 6-mer AIP. Each data point represents the peak area of the listed AIP divided by the peak area of the internal standard (AIN-(CDFLL); final concentration = 5 µM). Extracted ion chromatogram peaks (MS^1) were integrated for the calculated m/z of the peptide being quantified ± 5 ppm mass error. Standard curves were fit to a simple linear regression model. Data are shown as the mean ± standard deviation for three replicates.

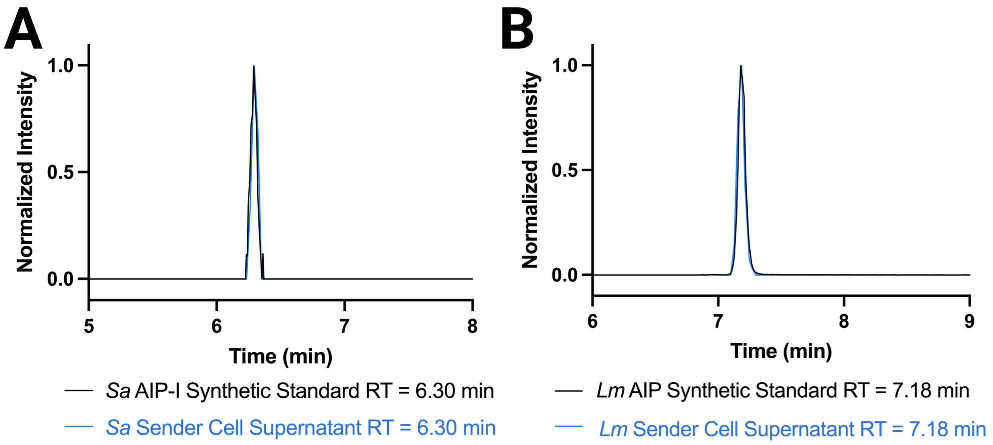

Figure S5. Extracted ion chromatograms (EICs) from MS^1 analyses of *S. aureus* and *L. monocytogenes* sender cell supernatants. For all parts, peak intensity was normalized such that the peak maximum was set to 1. (A) EIC for singly charged molecular ion peak with an m/z of 961.3794 ± 5 ppm mass error for the *Sa* sender cell supernatant (blue) and a *S. aureus* AIP-I synthetic standard (black), with retention times (RT) shown below the plot. (B) EIC for singly charged molecular ion peak with an m/z of 699.2993 ± 5 ppm mass error for the *Lm* sender cell supernatant (blue) and a *L. monocytogenes* 6-mer AIP synthetic standard (black), with retention times (RT) shown below the plot.

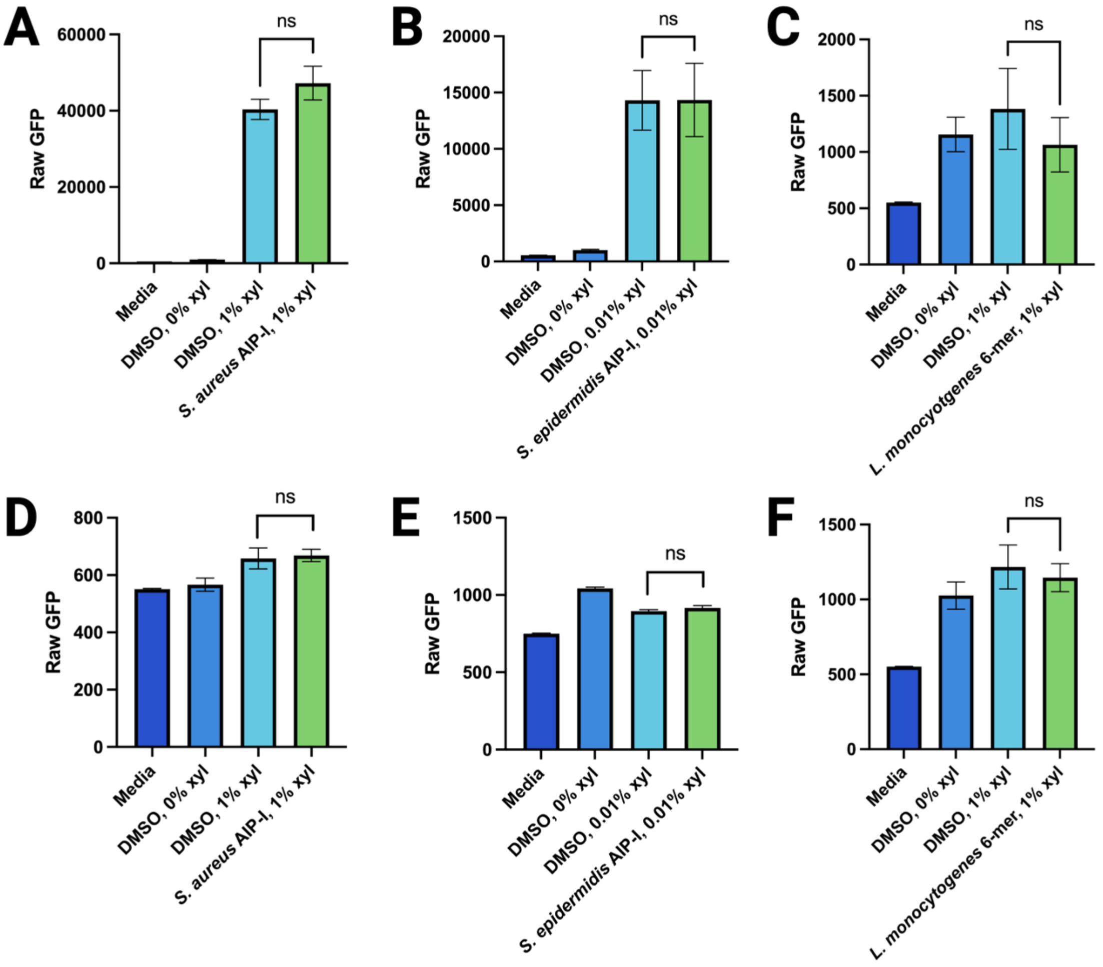

Figure S6. Fluorescence data for AgrA- and AgrC-only control *B. subtilis* cells. A media control (containing no cells, dark blue bar) is included on each plot as a measure of background fluorescence. (A-C) Fluorescence data for *B. subtilis* cells containing the (A) pSa-AgrA-GFP, (B) pSe-AgrA-GFP and (C) pLm-AgrA-GFP vectors. Significant fluorescence was observed in the *S. aureus* or *S. epidermidis* AgrA constructs when the constructs were induced with xylose, with no difference between DMSO and native AIP treatment (turquoise vs. green bars, respectively). No significant fluorescence differences were observed for the *L. monocytogenes* AgrA construct under all tested conditions. (D-F) Fluorescence data for *B. subtilis* cells containing the (D) pSa-AgrC-GFP, (E) pSe-AgrC-GFP and (F) pLm-AgrC-GFP vectors. No significant fluorescence was observed for these constructs under all tested conditions. Error bars represent the standard error of the mean of at least three biological replicates, each tested in duplicate (n=3). Significance values were determined using one-way ANOVA with Šídák's multiple comparisons test ($p_{adj} < 0.05$).

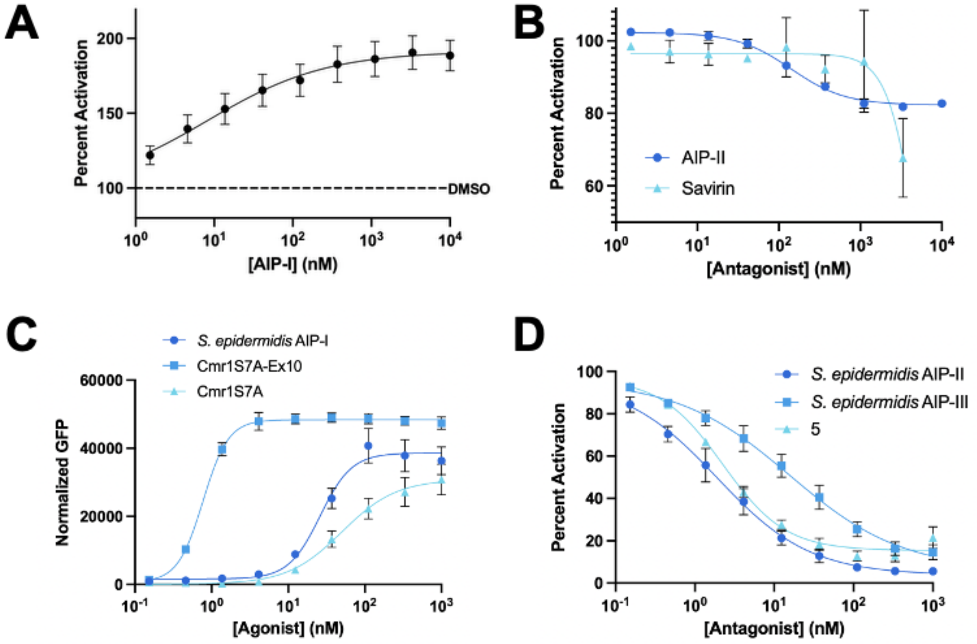

Figure S7. Representative dose-response data for certain QS modulators in *S. aureus* and *S. epidermidis* receiver and full cells. (A) Dose-response curve of *S. aureus* AIP-I in *Sa* receiver cells, showing a dose-dependent increase in fluorescence upon addition of the native AIP. Fluorescence normalized to media (0% activity) and DMSO controls (100% activity). (B) Dose-response curves of *S. aureus* AIP-II and the small molecule inhibitor savirin in *Sa* full cells, showing a dose-dependent decrease in fluorescence upon addition of both inhibitors. Fluorescence normalized to media (0% activity) and DMSO (100% activity) controls. (C) Dose-response curves of select agonists in *Se* receiver cells, showing dose-dependent increases in fluorescence upon addition of native and non-native agonists. Fluorescence normalized to growth and media controls. We note that activity is not reported as percent activation due to the lack of basal AIP production in the receiver cells that is usually standardized as "100% activity." (D) Dose-response curves of select antagonists in *Se* full cells, showing dose-dependent decreases in fluorescence upon addition of antagonists. Fluorescence normalized media (0% activity) and DMSO (100% activity) controls. Error bars represent the standard error of the mean of three biological replicates, each tested in triplicate (n=3).

Method for AgrC labeling experiments.

Separate cultures of *S. aureus* LAC, *B. subtilis* 168, and the *Sa* receiver cell lysostaphin (SaRC-LysT) construct were grown overnight for 20 h in LB medium. The following day, 1 mL aliquots of cells were pelleted via centrifugation at 13,200 rpm for 2 min. Supernatant was decanted, and cells were washed with 500 µL of PBS (pH 7.4) twice. Cells were resuspended in 250 µL of PBS, and OD_{600} was read on a Biotek Synergy 2 plate reader running Gen 5 software (version 1.05). Cells were split in half and moved to 0.6 mL Eppendorf tubes. In one tube, TAMRA-AIP-III D4A[4] (from a 1 mM DMSO stock solution; see below for structure) was added to one tube of cells to a final concentration of 1 µM (1% DMSO). DMSO (1%) was added to the other tube as a negative control. The samples were covered and incubated at room temperature with mild shaking (on an orbital shaker) for 40 min. Cells were pelleted and washed with 125 µL of PBS twice to remove any unbound TAMRA-AIP-III D4A. Final solutions were plated in a black 384-well microtiter plate (Corning), and fluorescence was read at 540/595 nm on the plate reader. Fluorescence was normalized to growth and DMSO controls. Fluorescence levels serve as an estimate for relative AgrC expression levels between the three strains. The results shown in **Figure S8** indicate that the amount of AgrC expressed upon induction with xylose is significantly higher than that observed in WT *S. aureus* LAC cells.

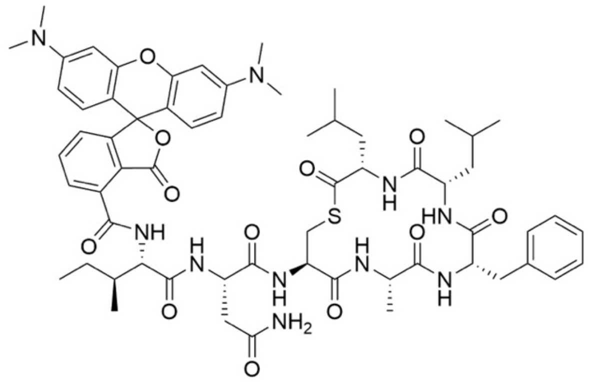

TAMRA-AIP-III D4A

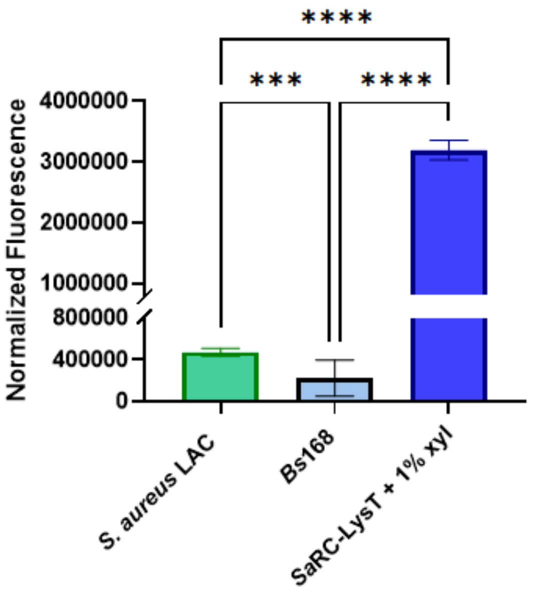

Figure S8. Examination of relative AgrC-I expression levels via fluorescent labeling. AgrC-I expression in *S. aureus* LAC versus *B. subtilis* SaRC-LysT cells induced with 1% xylose. Cultures of SaRC-LysT treated with xylose were grown in LB medium for 20 h. Cells were pelleted, washed with PBS twice, and incubated with 1 μM TAMRA-AIP-III D4A for 40 min. After incubation, cells were washed with PBS to remove unbound probe. Cells were resuspended in PBS, and fluorescence was measured at 540/595 nm. Data was corrected for background fluorescence observed from the DMSO control and further normalized to OD_{600}. Error bars represent the standard error of the mean of at least three biological replicates, each in technical duplicate (n=3). Significance values were determined using a one-way ANOVA with Šídák's multiple comparisons test where *** and **** represent $p_{adj} < 0.001$ and $p_{adj} < 0.0001$ respectively.

Table S3. QS modulator activity data in *S. aureus* and *S. epidermidis agr* reporter strains. These data are reproduced from our past studies (cited in Reference column) for comparisons in the current study. CI = confidence interval for IC_{50} or EC_{50} value.

Compound	EC_{50} (nM)	IC_{50} (nM)	EC_{50}/IC_{50} 95% CI (nM)	Reference
Agonism activity in S. aureus group-I (RN9222))				
S. aureus AIP-I	3.21	-	1.21 - 8.57	[5]
Antagonism activity in S. aureus group-I (AH1677)				
S. aureus AIP-II	-	1.62	0.927 - 2.82	[6]
S. aureus AIP-III	-	5.05	2.46 - 10.4	[6]
S. aureus AIP-III D4A	-	0.485	0.289 - 0.813	[6]
**AAA**	-	5.74	4.21 - 8.29	[7]
**12**	-	1.33	1.05 - 1.65	[7]
Savirin	-	9200	8700 - 9600	[4]
Agonism activity in S. epidermidis group- I (AH3408)				
S. epidermidis AIP-I	339	-	278 - 418	[7]
**AA**	10.3	-	6.18 - 17.2	[8]
**Cmr1**	>2000	-	-	[7]
**Cmr1S7A**	378	-	290 - 523	[7]
**Cmr1S7A-Ex10**	32	-	23.1 - 42.9	[7]
**Cmr7F9(3FPhe)**	168	-	68.9 - 2330	[7]
**8**	1620	-	915 - 5760	[7]
**10**	70.9	-	24.7 - 145	[7]
Antagonism activity in S. epidermidis group- I (AH3408)				
S. epidermidis AIP-II	-	7.77	5.90 - 11.2	[7]
S. epidermidis AIP-III	-	390	290 - 553	[7]
**AAA**	-	2.04	1.54 - 2.72	[7]
**5**	-	40.4	31.9 - 50.3	[7]
**6**	-	1.64	0.918 - 3.10	[7]
**12**	-	1.21	1.04 - 1.43	[7]
**Cmr3S6α**	[a]	[a]	-	[9]

[a]Compound previously found to be inactive in both agonism and antagonism style reporter assays.

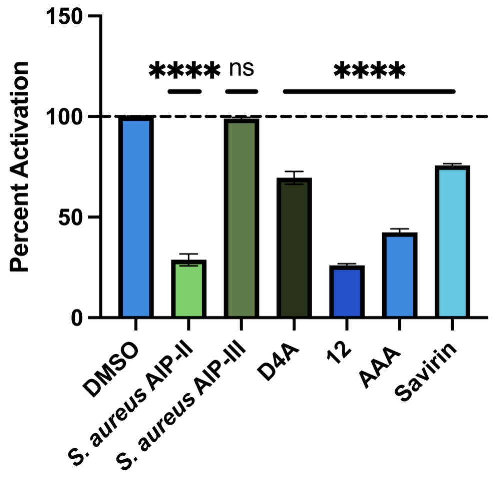

Figure S9. QS inhibitor activity in *S. aureus* full cells induced with 0.1% xylose. All compound concentrations are 10 μM. Dotted line represents vehicle (DMSO) fluorescence for ease of comparison. Fluorescence normalized to media (0% activity) and DMSO (100% activity) controls. Error bars represent the standard error of the mean of three biological replicates, each tested in duplicate (n=3). Significance values represent comparison to the vehicle control (DMSO) and were determined using a one-way ANOVA with Šídák's multiple comparisons test ($p_{adj} < 0.0001$).

```
Sa AgrB-I   1    MNYFDNKIDQFATYLQKRNNLDHIQFLQVRLGMQVLAKNIGKLIVMYTIAYILNIFLFTL  60
Se AgrB-I   1    MKIIDKKIEQFAQYLQRKNNLDHIQFLKIRLGMQVLAINIEKSIVVYGLAIIFHTFFYTL  60

Sa AgrB-I   61   ITNLTFYLIRRHAHGAHAPSSFWCYVESIILFILLPLVIVNFHINFLIMIILTVISLGVI  120
Se AgrB-I   61   LTHLSYFLIRRHAHGTHANSSLLCHIQNIIFFIIFPYLIIKLDINYFVLLSMALVGLIIT  120

Sa AgrB-I   121  SVYAPAATKKKPIPVRLIKRKKYYAIIVSLTLFIITLIIKEPFAQFIQLGIIIEAITLLP  180
Se AgrB-I   121  ILYAPAATKKQPIPRRLVKRKKILSIFLYCTIVVISLVTKEPVNKLILFGVILESLTLLP  180

Sa AgrB-I   181  IFFIKEDL  188
Se AgrB-I   181  IFFPKEDI  188


Sa AgrD-I   1    MNTLFNLFFDFITGILKNIGNIAAYSTCDFIMDEVEVPKELTQLHE*  47
Se AgrD-I   1    MENIFNLFIKFFTTILEFIGTVAGDSVCASYFDEPEVPEELTKLYE*  47
```

Figure S10. NCBI BLAST protein alignment of *S. aureus* and *S. epidermidis* (top) AgrB and (bottom) AgrD sequences used in *B. subtilis* sender and full cells.[10]

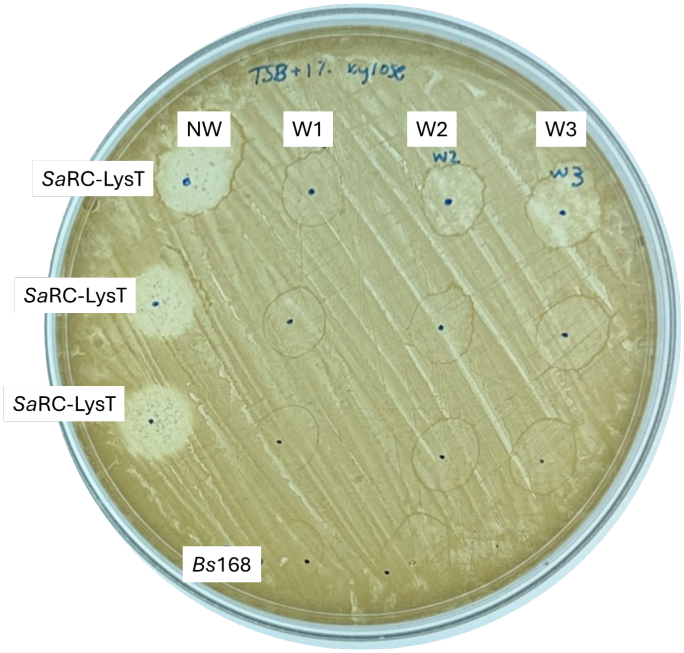

Figure S11. *S. aureus* receiver cell LysT zone of inhibition assay data for supernatant controls. Image of representative agar plate shown. *Sa* receiver cells LysT were grown with lincomycin (25 µg/mL) and erythromycin (1 µg/mL) in LB medium (w/o xylose) for 20 h. Cells were pelleted and supernatant was collected. Cells were then washed 3x in PBS, and aliquots of each of the three washes were spotted onto a lawn of *S. aureus* LAC grown on TSB + 1% xylose agar (labels W1-W3) alongside an aliquot of the original supernatant collected (label NW). The washes were sufficient to remove antibiotics from the cell culture, as observed by the lack of growth inhibition in the W1-W3 spots and the growth inhibition in the NW spot. Three replicates are shown (label *Sa*RC-LysT). *B. subtilis* 168 supernatant was also plated as a control (label *Bs*168), and no growth inhibition was observed.

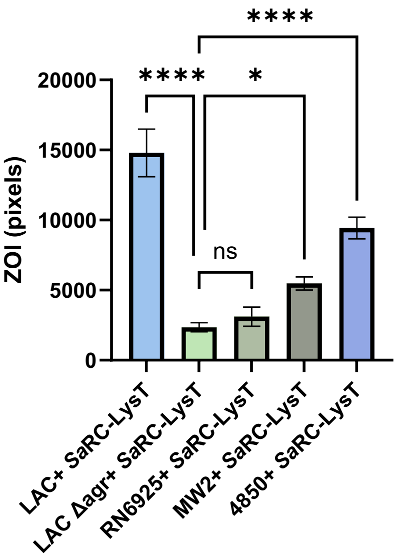

Figure S12. *S. aureus* receiver cell LysT versus *S. aureus* agr groups I-IV zone of inhibition quantification. ImageJ quantification of the zones produced by the *Sa*RC when grown on a lawn of each of the different agr groups from *S. aureus*. LAC, MW2, and 4850 (groups -I, -III, and -IV respectively) activate this system in line with reporter data discussed in the main text. While the *S. aureus* group-II strain used (RN6925) appears to have clear zones of inhibition in this assay, these are not statistically significant when compared to the LAC Δ*agr* strain at this xylose concentration, suggesting the observed activity likely reflects leaky expression rather than activation by AIP-II. Error bars represent standard deviation of three replicates. Significance testing was performed using one-way ANOVA with Šídák's multiple comparisons test. Differences with * and **** were deemed significant with $p_{adj} < 0.05$ and $p_{adj} < 0.0001$, respectively.

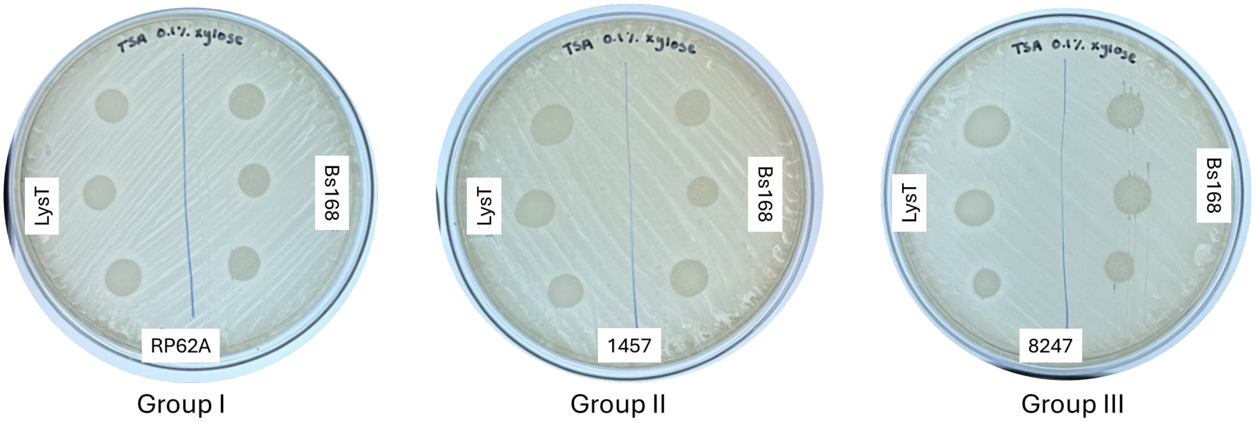

Figure S13. *S. aureus* receiver cell LysT versus *S. epidermidis agr* groups I–III zone of inhibition assay data. *From left to right:* lawns of *S. epidermidis* RP62A (group I), 1457 (group II), and 8247 (group III) grown on TSB + 0.1% xylose agar. Aliquots (5 µL) of SaRC-LysT or *B. subtilis* 168 (empty control) were spotted onto the lawns (left and right halves of the plate, respectively) and allowed to grow for 24 h prior to imaging.

MIC measurement method for lysostaphin in *S. aureus* LAC.

Overnight cultures of *S. aureus* LAC were grown in TSB medium and incubated at 37 °C with 200 rpm shaking for 20 h. A 1 mg/mL stock of purified lysostaphin (Fisher Scientific) was prepared in MilliQ water and diluted to a concentration of 64 µg/mL in TSB medium. Subsequent 2-fold dilutions were performed in TSB medium, and 50 µL aliquots of each dilution were plated in a clear 96-well microtiter plate (Costar). The overnight culture of *S. aureus* LAC was diluted 1:1000 in fresh TSB medium, and 50 µL aliquots were added to each well in the plate. A 100 µL *S. aureus* LAC culture grown in the absence of lysostaphin was used as a control. The plate was incubated statically at 37 °C for 24 h. The OD_{600} of each well was measured on a Biotek Synergy 2 plate reader running Gen 5 software (version 1.05). Compounds were tested in three biological replicates, each in technical duplicate. Measurements were background corrected and normalized to the *S. aureus* LAC control. Data were plotted using GraphPad Prism version 10.4.2 and are shown in **Figure S14**.

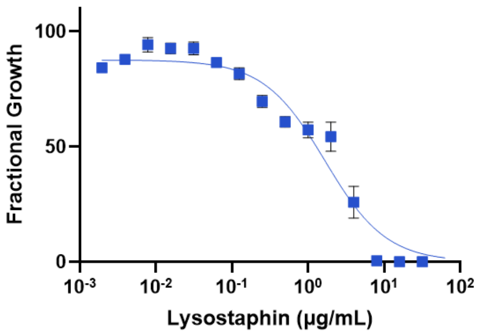

Figure S14. Growth inhibition of *S. aureus* LAC upon treatment with lysostaphin and MIC determination. Growth of *S. aureus* (LAC) was measured by monitoring OD_{600} after exposure to lysostaphin over a range of concentrations. A non-linear regression curve was generated in GraphPad Prism (version 10.4.2) using a four-parameter variable slope equation constraining the bottom to 0. Error bars indicate the standard error of the mean (SEM) across at least three biological replicates, each tested in duplicate (n=6). The MIC_{50} for lysostaphin in *S. aureus* LAC was calculated to be 1.67 µg/mL.

<u>Method to estimate lysostaphin concentration produced by *S. aureus* receiver cell LysT construct.</u>

We utilized the non-linear regression equation to back-calculate the concentration of lysostaphin released from the *Sa* receiver cell LysT construct. By rearranging the variables in Equation S1 to generate Equation S2, we can estimate the concentration of lysostaphin produced by comparing the growth inhibition of sample cultures to that observed for purified lysostaphin.

Variable	Definition	Value
**Y**	Growth	From experiment
**X**	Lysostaphin concentration in µg/mL	To be calculated
**T**	Top plateau in activity model	87.58
**H**	Hill slope	-1.079
**IC_{50}**	Inhibitory concentration at 50% activity	1.67

Equation S1:

$$y = \frac{T}{1+(\frac{IC_{50}}{x})^H}$$

Equation S2:

$$x = IC_{50}\,(\frac{T}{y} - 1)^{-\frac{1}{H}}$$

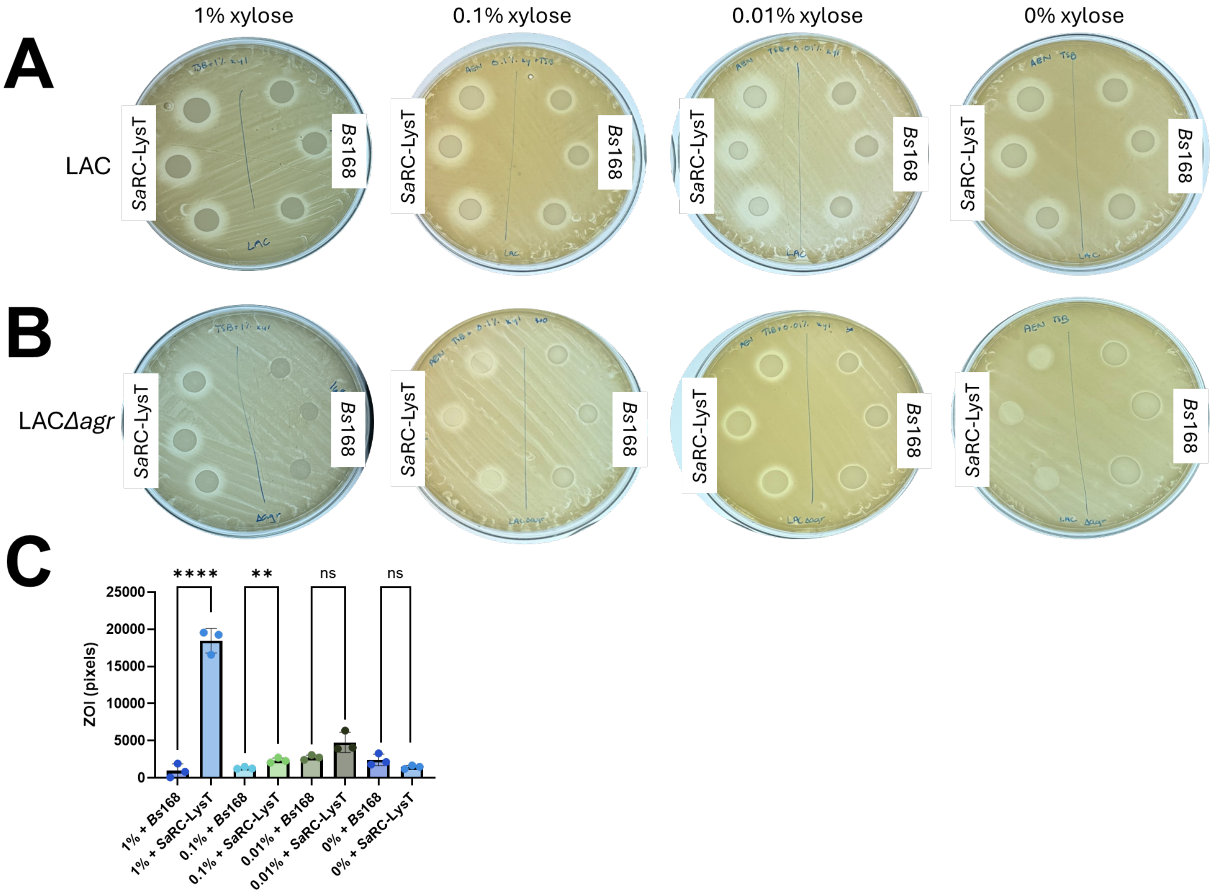

Figure S15. Effect of xylose percentage on zone of inhibition assay analysis. Lawns of either (A) *S. aureus* LAC or (B) *S. aureus* LAC *Δagr* were grown on TSB agar plates with varying xylose concentrations (0–1%, right to left). Aliquots (5 μL) of *Sa* receiver cell LysT or *B. subtilis* 168 were spotted onto the lawns (left and right halves of each plate, respectively). Zones of inhibition (larger than the Bs168 background effects) are observed in *S. aureus* LAC plates at 0% xylose and in *S. aureus* LAC *Δagr* plates at 1% and 0.1% xylose, suggestive of some background leaky expression of lysostaphin. (C) Plot of ImageJ analysis of zone of inhibition data for LAC *Δagr* in panel (B) showing leaky expression at 1% and 0.1% xylose. Statistical significance was determined using a t-test comparing the means of the zones from treatment with *B. subtilis* 168 or *S. aureus* RC-LysT at each of the xylose concentrations. ** and **** represent $p < 0.01$ and $p < 0.0001$, respectively.

References.

[1] Popp, P. F., Dotzler, M., Radeck, J., Bartels, J., and Mascher, T. (2017) The *Bacillus* BioBrick Box 2.0: expanding the genetic toolbox for the standardized work with *Bacillus subtilis*, *Sci. Rep. 7*, 15058. https://doi.org/10.1038/s41598-017-15107-z

[2] Gibson, D. G., Young, L., Chuang, R. Y., Venter, J. C., Hutchison, C. A., 3rd, and Smith, H. O. (2009) Enzymatic assembly of DNA molecules up to several hundred kilobases, *Nat. Methods 6*, 343-345. https://doi.org/10.1038/nmeth.1318

[3] Gonzalez, L. M., Mukhitov, N., and Voigt, C. A. (2020) Resilient living materials built by printing bacterial spores, *Nat. Chem. Biol. 16*, 126-133. https://doi.org/10.1038/s41589-019-0412-5

[4] Polaske, T. J., Vulpis, T. D., Nelson, A. E., Zhao, K., and Blackwell, H. E. (2026) A Small Molecule That Inhibits the Quorum Sensing Receptor AgrC in *Staphylococcus aureus*, *J. Am. Chem. Soc. 148*, 9199-9204. https://doi.org/10.1021/jacs.5c21051

[5] Tal-Gan, Y., Stacy, D. M., and Blackwell, H. E. (2014) N-Methyl and peptoid scans of an autoinducing peptide reveal new structural features required for inhibition and activation of AgrC quorum sensing receptors in *Staphylococcus aureus*, *Chem. Commun. (Camb.) 50*, 3000-3003. https://doi.org/10.1039/c4cc00117f

[6] Tal-Gan, Y., Stacy, D. M., Foegen, M. K., Koenig, D. W., and Blackwell, H. E. (2013) Highly potent inhibitors of quorum sensing in *Staphylococcus aureus* revealed through a systematic synthetic study of the group-III autoinducing peptide, *J. Am. Chem. Soc. 135*, 7869-7882. https://doi.org/10.1021/ja3112115

[7] Eisenbraun, E. L., Vulpis, T. D., Prosser, B. N., Horswill, A. R., and Blackwell, H. E. (2024) Synthetic Peptides Capable of Potent Multigroup Staphylococcal Quorum Sensing Activation and Inhibition in Both Cultures and Biofilm Communities, *J. Am. Chem. Soc. 146*, 15941-15954. https://doi.org/10.1021/jacs.4c02694

[8] Yang, T., Tal-Gan, Y., Paharik, A. E., Horswill, A. R., and Blackwell, H. E. (2016) Structure-Function Analyses of a *Staphylococcus epidermidis* Autoinducing Peptide Reveals Motifs Critical for AgrC-type Receptor Modulation, *ACS Chem. Biol. 11*, 1982-1991. https://doi.org/10.1021/acschembio.6b00120

[9] West, K. H. J., Shen, W., Eisenbraun, E. L., Yang, T., Vasquez, J. K., Horswill, A. R., and Blackwell, H. E. (2021) Non-Native Peptides Capable of Pan-Activating the agr Quorum Sensing System across Multiple Specificity Groups of *Staphylococcus epidermidis*, *ACS Chem. Biol. 16*, 1070-1078. https://doi.org/10.1021/acschembio.1c00240

[10] Altschul, S. F., Madden, T. L., Schaffer, A. A., Zhang, J., Zhang, Z., Miller, W., and Lipman, D. J. (1997) Gapped BLAST and PSI-BLAST: a new generation of protein database search programs, *Nucleic Acids Res. 25*, 3389-3402. https://doi.org/10.1093/nar/25.17.3389
